# A robust gene expression-based prognostic risk score predicts overall survival of lung adenocarcinoma patients

**DOI:** 10.18632/oncotarget.23490

**Published:** 2017-12-15

**Authors:** En-Guo Chen, Pin Wang, Haizhou Lou, Yunshan Wang, Hong Yan, Lei Bi, Liang Liu, Bin Li, Antoine M. Snijders, Jian-Hua Mao, Bo Hang

**Affiliations:** ^1^ Department of Pulmonary Medicine, Sir Run Run Shaw Hospital, College of Medicine, Zhejiang University, Hangzhou, China; ^2^ Biological Systems and Engineering Division, Lawrence Berkeley National Laboratory, Berkeley, CA, USA; ^3^ Department of Gastroenterology, Nanjing Drum Tower Hospital, Nanjing University Medical School, Nanjing, Jiangsu, China; ^4^ Department of Medical Oncology, Sir Run Run Shaw Hospital, School of Medicine, Zhejiang University, Hangzhou, China; ^5^ Department of Oncology, Fudan University Shanghai Cancer Center, Shanghai, China; ^6^ Nanjing KDRB Biotech Inc., Ltd, Jiangning District, Nanjing, Jiangsu, China

**Keywords:** lung adenocarcinoma, overall survival, prognosis, gene signature, bioinformatics

## Abstract

Identification of reliable predictive biomarkers and new therapeutic targets is a critical step for significant improvement in patient outcomes. Here, we developed a multi-step bioinformatics analytic strategy to mine large omics and clinical data to build a prognostic scoring system for predicting the overall survival (OS) of lung adenocarcinoma (LuADC) patients. In latter we first identified 1327 significantly and robustly deregulated genes, 600 of which were significantly associated with the OS of LuADC patients. Gene co-expression network analysis revealed the biological functions of these 600 genes in normal lung and LuADCs, which were found to be enriched for cell cycle-related processes, blood vessel development, cell-matrix adhesion and metabolic processes. Finally, we implemented a multiple resampling method combined with Cox regression analysis to identify a 27-gene signature associated with OS, and then created a prognostic scoring system based on this signature. This scoring system robustly predicted OS of LuADC patients in 100 sampling test sets and was further validated in four independent LuADC cohorts. In addition, in comparison to other existing prognostic gene signatures published in the literature, our signature was significantly superior in predicting OS of LuADC patients. In summary, our multi-omics and clinical data integration study created a 27-gene prognostic risk score that can predict OS of LuADC patients independent of age, gender and clinical stage. This score could guide therapeutic selection and allow stratification in clinical trials.

## INTRODUCTION

Lung cancer is the leading cause of cancer-related death worldwide [1], where non-small cell lung cancer (NSCLC) is the most common type of cancer affecting the lungs with adenocarcinoma being the most common subtype. Microarray and next generation sequencing technologies have become invaluable tools to deconvolute the genetic heterogeneity and complexity of NSCLC, providing tremendous information to define new biomarkers for diagnosis, prognosis and prediction of therapeutic response, and to identify new potential therapeutic targets. Despite the advances in our knowledge of the genetic factors underlying this disease, the five-year survival rate for NSCLC patients is approximately 21% [2]. Lung cancer treatment is therefore moving rapidly towards an era of personalized medicine, where the molecular characteristics of an individual patient's tumor will dictate the optimal treatment modalities. For example, NSCLC patients with *EGFR* mutations show significantly improved responses to treatment with tyrosine kinase inhibitors, *e.g*., gefitinib or erlotinib, that target this protein [3].

Patient stratification based on histopathological markers, immunohistochemistry and other molecular factors has been evaluated to improve treatment decisions in lung adenocarcinoma (LuADC) patients [4–6]. The availability of large cancer genomic data sets allows for unbiased approaches to identify multi-gene signatures important in tumor progression. Gene transcript based signatures that predict prognosis have successfully been developed for many different tumor types [7–10]. A number of gene signatures using microarray analysis show promise for prognosis or prediction of response to therapy in NSCLC [11–14]. However, these signatures were either based on incomplete genome annotation or were based solely on existing knowledge. Therefore, a new comprehensive and unbiased genome-wide screening for genes associated with lung cancer prognosis is warranted.

Here we developed a multi-step bioinformatics analytic strategy to mine large omics data together with clinical information to develop a gene expression-based prognostic risk score for LuADCs. We employed a resampling method by splitting the LuADCs TCGA dataset into training and testing sets and then used repeated cross-validation to identify critical genes for prognostic classification. Based on these analyses, we created a 27-gene expression prognostic scoring system and successfully applied it to predict overall survival (OS) in multiple validation datasets. Our study raises the prospect that the practicality of LuADC patient prognosis may be assessed by this prognostic scoring system.

## RESULTS

### Identification of consistently deregulated genes in human LuADCs

**Figure 1:**
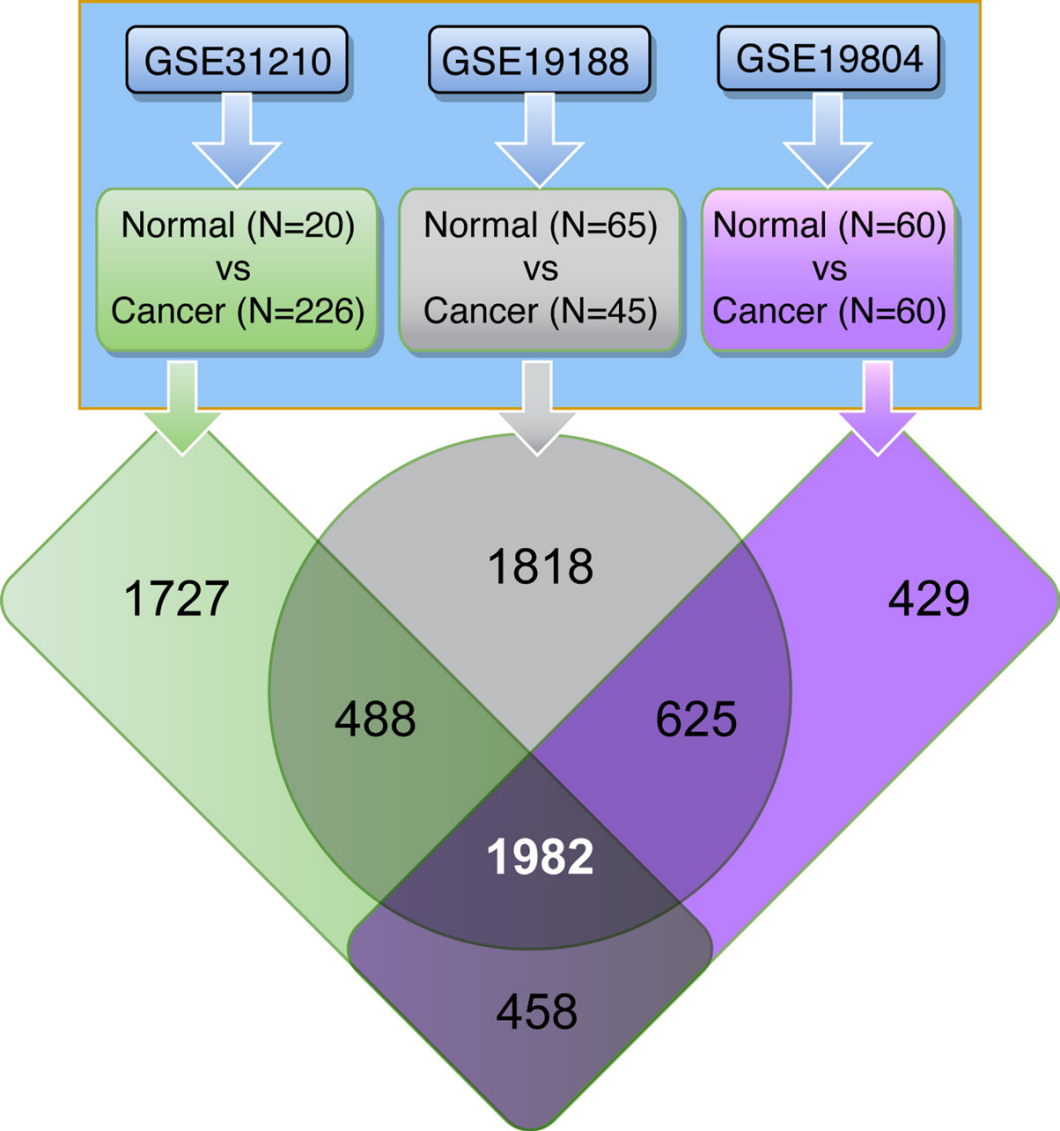
Human lung tissue data sets used in this study Three independent gene transcript data sets containing LuADC and normal lung tissue samples were used. Differential expression of tumor versus normal using a fold-change cut-off of 5.0 and adjusted *p*-value < 0.0001 identified the 1982 common probe IDs consistently deregulated in all three datasets.

A meta-analysis of three publically available LuADC transcriptome datasets (GSE31210, GSE19188 and GSE19804) was conducted to identify genes that are consistently deregulated in human LuADCs compared to normal lung tissues (Figure 1). The significant differential expression of genes was assessed by a fold change cut-off of 5 and adjusted *p*-value < 0.0001 (Supporing Information Supplementary Table 1). This resulted in a set of 1982 probe IDs (1374 down-regulated and 608 up-regulated) represented by 1327 unique genes (884 down-regulated and 543 up-regulated), which were consistently deregulated in all three datasets (Figure 1; Supporing Information Supplementary Table 1).

### Impact of the deregulated genes on overall survival in human LuADCs

**Figure 2:**
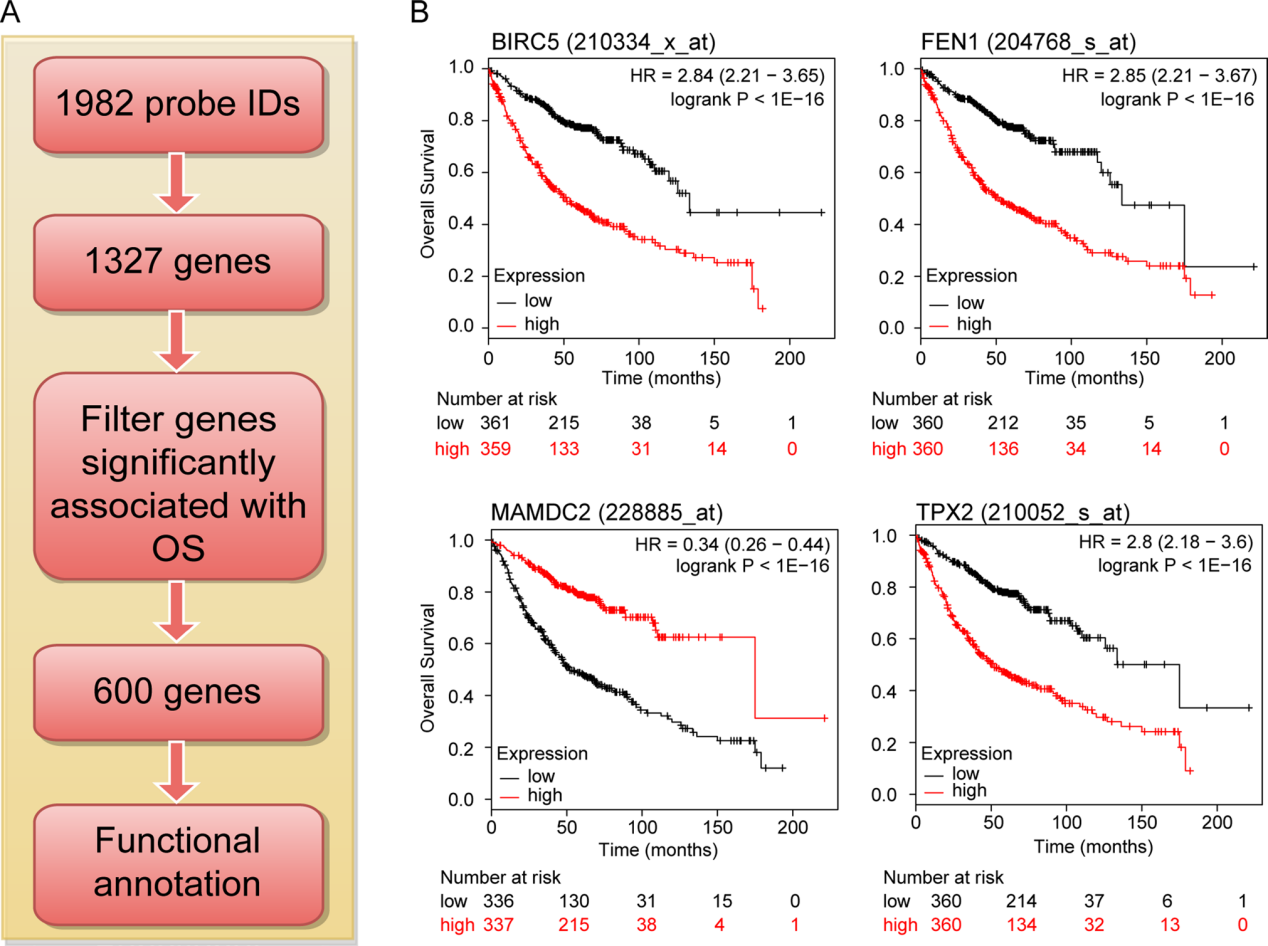
Flow diagram for identifying and validating a prognostic biomarker panel for LuADC (**A**) The 1982 robustly deregulated probe IDs represented 1327 genes of which 600 were significantly associated with LuADC overall survival used for functional analysis. (**B**) Kaplan-Meier survival curves for individual genes significantly associated with overall survival in LuADC patients. The LuADC patient cohort was divided into two equal groups based on median expression for each gene and compared by a Kaplan-Meier survival analysis. The estimate of the hazard ratio (HR) and log-rank *p*-value of the curve comparison between the groups is shown.

To assess the importance of the 1327 deregulated genes in LuADC development, we evaluated their prognostic value for LuADC patients in a large public database combining tumor gene expression and patient survival [17] (Figure 2A). The LuADC patient cohort was divided into two equal groups based on median expression for each gene. Subsequently, the effects of high or low expression levels on OS were examined using the Kaplan-Meier survival curve and log-rank test. This analysis identified 600 out of 1327 genes that were significantly associated with OS (adjusted *p*-value < 0.05; Figure 2B, Supporing Information Supplementary Table 2). 406 genes had a hazard ratio (HR) < 1 (higher gene expression associated with good prognosis) and 194 genes had a HR > 1 (higher gene expression associated with poor prognosis) (Supporing Information Supplementary Table 2).

**Figure 3:**
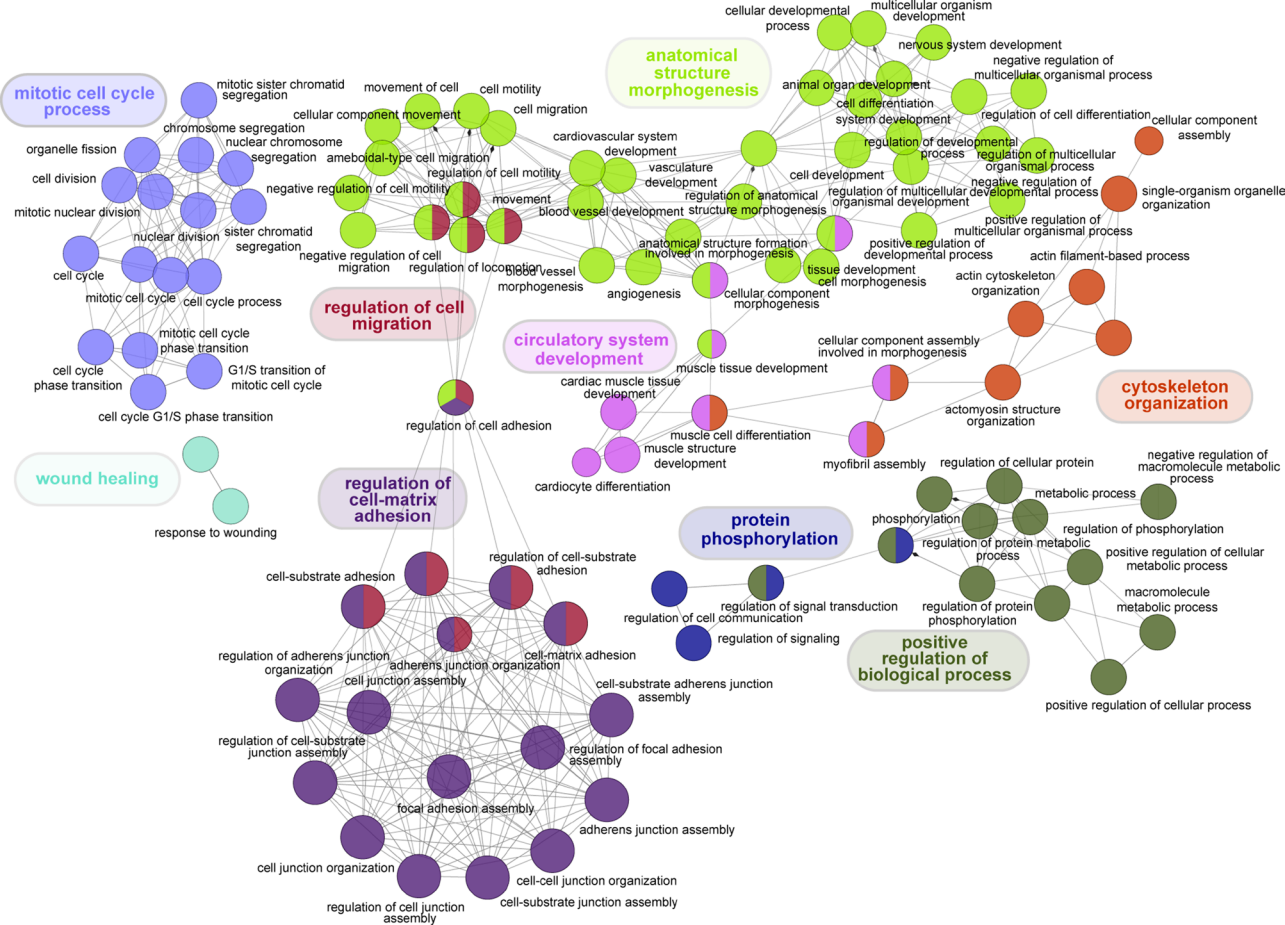
Visual representation of Gene Ontology enrichment analysis of genes significantly associated with OS in LuADC Functional enrichment analysis of the 600 genes significantly associated with OS was performed using ClueGo based on Gene Ontology categories (*p* < 0.001). Non-redundant biological terms for our 600-gene set were visualized in a functionally grouped network and related processes were colored by function.

To reveal the molecular mechanism underlying LuADC development, we determined which Gene Ontology (GO) categories are statistically overrepresented in the 600 gene set. ClueGo was used to integrate GO terms and create a functionally organized GO network (Figure 3) [18]. We observed significant enrichment for cell cycle, adhesion, cell death, angiogenesis, metabolism and kinase activity (Figure 3), all of which are hallmarks of cancer.

### Expression architecture of prognostic genes in normal lung and LuADCs

**Figure 4:**
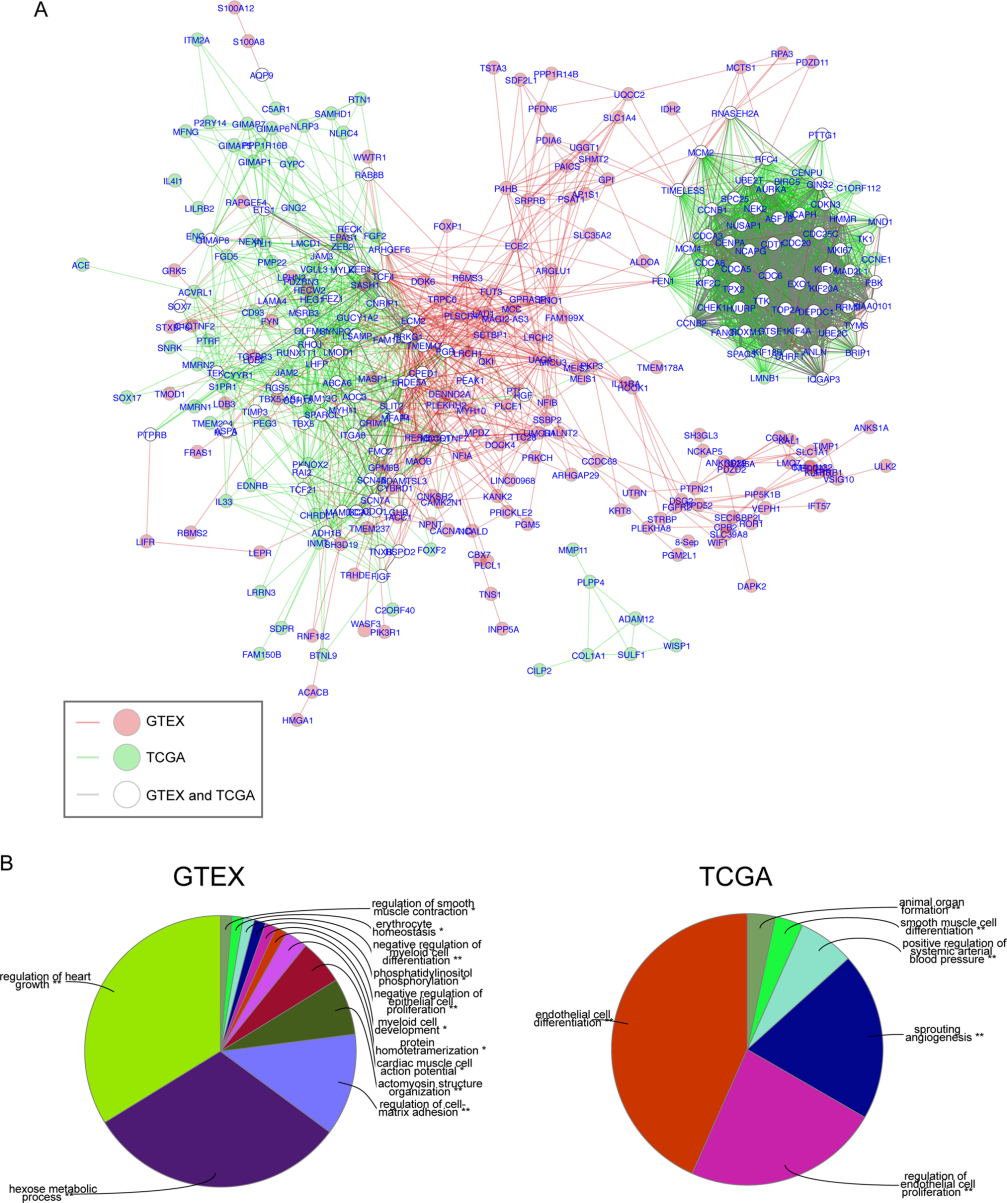
Comparison network of gene correlations in normal lung and LuADC (**A**) Gene co-expression correlation networks for normal lung and LuADC were generated based on correlation coefficients (R ≥| 0.7|; adjusted *p*-value < 0.001) among 600 genes in normal and LuADC tissue samples and then merged using DyNet. Differences between the two merged networks based on node and edge presence were highlighted. Nodes and/or edges present in both normal and tumor correlation networks are represented in white and gray, respectively. Nodes and/or edges present in either normal or tumor networks alone are represented in red and green, respectively. (**B**) Functional enrichment analysis of genes uniquely present in the normal lung correlation network (left) or the LuADC correlation network (red) based on Gene Ontology.

Co-expression network analysis has been used to identify clusters of genes with common biological functionality important in normal or tumor tissues. We used data obtained from the GTEx database of 320 normal human lung tissues and the TCGA database of 517 LuADC samples to reveal the expression architecture of 600 OS-associated genes in normal lung and LuADC tissues. We first calculated correlation coefficients among 600 genes in both normal and LuADC tissue samples, and then constructed a gene co-expression network where nodes represent individual genes and edges connecting genes represent a significant correlation in expression (R ≥ |0.7|; adjusted *p*-value < 0.001; Supporing Information Supplementary Figure 1). We then performed a comparison analysis between these two correlation networks by generating a composite network highlighting nodes and edges that were found exclusively in normal lung (red), exclusively in LuADC (green) or present in both (white) (Figure 4A). This analysis revealed a shared co-expression clique enriched for cell cycle and mitosis genes and a second, larger, clique containing a sub-clique of genes co-expressed in normal lung (red) and genes co-expressed in LuADC (green). Gene Ontology analysis of these subcliques revealed significant enrichment for muscle growth, metabolism and cell-matrix adhesion in normal lung and endothelial cell differentiation and angiogenesis in LuADC (Figure 4B).

### Development of a gene expression signature-based prognostic risk score in LuADC

**Figure 5:**
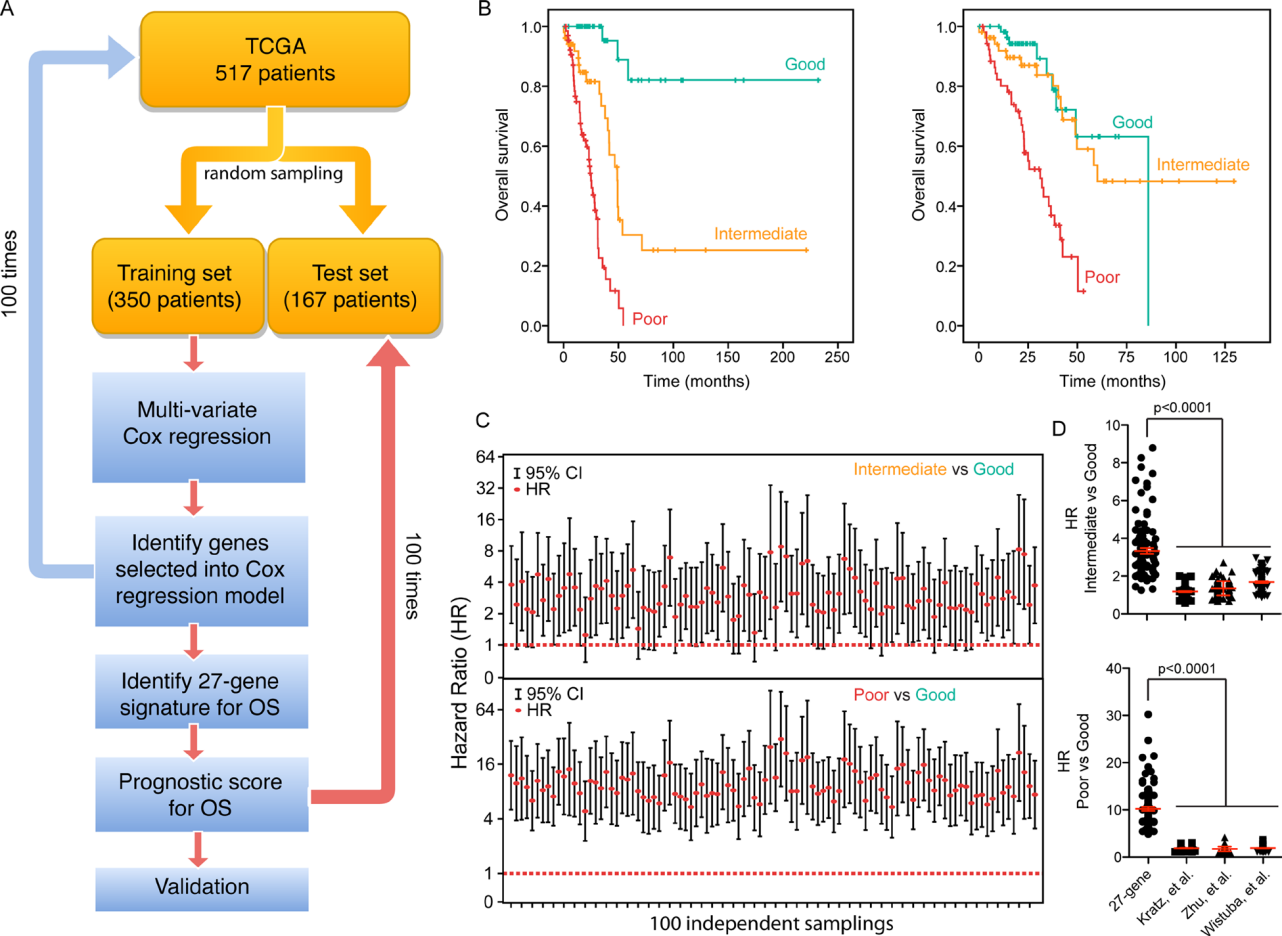
A 27-gene signature is associated with OS in LuADC patients (**A**) Cox regression was run on 100 random tumor samples for 600 genes significantly associated with OS to generate the 27-gene signature. The 27-gene signature was used to generate a prognostic scoring system, which was validated using 100 random test sets. (**B**) Kaplan-Meier overall survival curves for two representative test-cohorts separated into tertiles according to the prognostic score using the 27-gene signature. (**C**) For each of 100 test sets the HR and the 95% confidence interval was calculated using a Cox model based on the prognostic score with groups (good vs. poor: top; intermediate vs. poor: bottom). The red dotted line indicates a HR value of 1, or the null hypothesis. (**D**) Comparison of the HR for each of 100 test sets between the 27-gene signature and three existing gene signatures reported in the literature [12–14].

We designed a strategy to develop a prognostic scoring system (Figure 5A). We first used a resample method to split the TCGA dataset (total 517 patients) into 100 training (350 patients) and 100 testing (167 patients) datasets. We then performed a multivariate Cox regression analysis on all 100 training sets to discover statistically significant independent genes within the 600-gene set for predicting OS. The genes that recurred in at least 30% of 100 training sets were included in our final 27-gene signature (Supporing Information Supplementary Table 3). A prognostic score for a patient was used to assess a patient's risk of death and was defined as the linear combination of logarithmically transformed gene expression levels weighted by average Cox regression co-efficient obtained from 100 training data sets (Supporing Information Supplementary Table 4). The prognostic scores were assigned for all patients in both training and testing sets. In each training set, the patients were then divided into tertiles based on their prognostic score (good, intermediate and poor) and the prognostic score at the cut-points was recorded. Kaplan-Meier analysis was performed and a log-rank test was used to determine significant differences in OS among different groups for all training sets (Figure 5B). The hazard ratio (HR) was calculated for each testing set for the “intermediate” and “poor” groups in comparison to the “good” group (Figure 5C). In all test sets, patients in the “poor” group had a significant shorter OS than those in “good” group (HR confidence interval above “1”) (Figure 5C, bottom panel), where in more than 70% of the test sets, patients in the “intermediate” group had a significant shorter OS than those in “good” group (Figure 5C, top panel), indicating that this prognostic scoring system has discriminative ability to distinguish patients with good prognosis from patients with worse prognosis.

### 27-gene expression signature-based prognostic risk score independently predicts overall survival in LuADC patients

**Figure 6:**
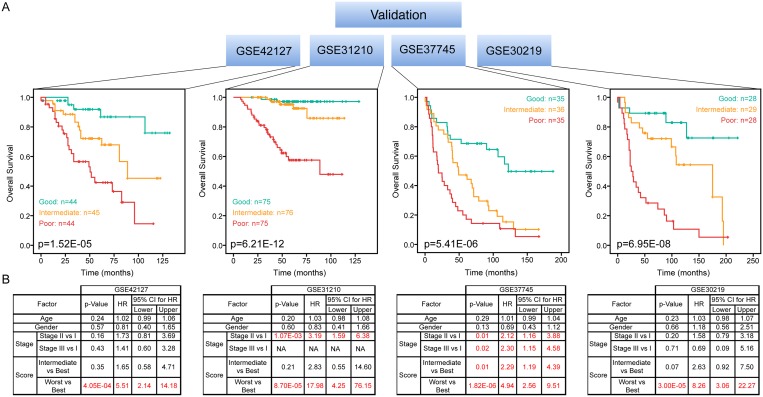
Independent validation of 27-gene signature Kaplan-Meier overall survival curves were generated for four independent LuADC patient cohorts according to the prognostic score using the 27-gene signature. The patient cohort was divided into tertiles based on the prognostic score and the log-rank *p*-value of the curve comparison between the groups is shown. The hazard ratio and the 95% confidence interval was calculated using a Cox model based on tumor stage (I–IV), gender, age at diagnosis and prognostic score as covariates. Significant factors are highlighted in red.

We then tested our 27-gene prognostic signature in four independent datasets of LuADC patients. Prognostic scores for all patients were calculated and patients were ranked based on their score and divided into three equal sized cohorts. Kaplan-Meier analysis revealed a significant difference among three patient cohorts. Patients with a high prognostic score had a significantly shorter OS compared to patients with a low prognostic score (*p* < 0.001) in all datasets (Figure 6A). Finally, we investigated whether our prognostic score was an independent prognostic factor over clinical information (age, gender and stage) using Cox regression. We conclude that our prognostic scores are independently and significantly associated with OS (Figure 6B).

### Comparison of 27-gene expression signature with existing prognostic signatures

There are a number of prognostic signatures for NSCLC prognosis in the literature. We compared the performance of three published signatures [12–14] with our 27-gene signature. For each of the published signatures, we performed a multivariate Cox regression analysis on the same 100 training sets, averaged the Cox regression co-efficient and calculated prognostic scores for all patients. For each signature, the patients were then divided into tertiles based on their prognostic scores and the prognostic scores at the cut-points were recorded. Finally, the HR was calculated for each testing set for the “intermediate” and “poor” groups in comparison to the “good” group (Supporing Information Supplementary Figure 2). The median HR of our 27-gene signature was on average 2.2-fold higher in the “intermediate” *vs.* “good” group and 5.0-fold higher in the “poor” vs “good” group compared to each of the three published signatures (Figure 5D). We conclude that our signature was significantly superior in predicting OS of the LuADC patients.

## DISCUSSION

Lung cancer is the most common cancer and the leading cause of cancer death among in men worldwide [1, 20]. NSCLC, like many other cancers, exhibits considerable complexity and heterogeneity in biology, drug response and survival [21], which represents a major obstacle to effective personalized treatment. This work aimed to identify reliable predictive biomarkers and build a prognostic scoring system for predicting OS of LuADC patients.

There are several prognostic signatures for NSCLC prognosis in the literature [12–14]. While these signatures have been shown to predict lung cancer survival, they were developed based on a subset of all genes in the genome or were assembled based on existing knowledge on the role of genes in cancer. With the availability of lung cancer transcriptome data sets covering many additional genes it seemed plausible that that novel gene signatures better able to predict LuADC patient survival could exist. To this end, we embarked on a comprehensive and unbiased genome-wide screen for genes associated with lung cancer prognosis. We show that our 27-gene scoring system has robust discriminative ability to distinguish patients with good versus bad prognosis in multiple datasets independent of clinical characteristics including age, gender and pathological stage. A direct performance comparison of our signature with the three published signatures mentioned above in terms of predicting patient survival showed that, while all signatures were able to predict survival, our 27-gene signature was much more robust. To translate such findings into clinical practice, a multigene assay should be developed for further validation of this gene signature in assessment of LuADC survival. Such information will assist treatment decision-making in a way similar to that used for the Oncotype DX breast cancer assay developed by Genomic Health [9] and Mammaprint 70-gene breast cancer recurrence assay by Agendia [7]. Randomized prospective clinical trials to further validate the accuracy and clinical value of this novel prognostic test for LuADC patients will need to be conducted.

In conclusion, lung cancer remains the leading cause of cancer-related disease burden. We developed a multi-step unbiased bioinformatics analytic approach to identify reliable predictive biomarkers and new therapeutic targets for LuADCs. We discovered that the expression of 600 genes are consistently altered in LUADCs and are significantly associated with OS of LuADC patients. Our study created a robust 27-gene prognostic signature that could predict patient overall survival independent of age, gender and clinical stage. This signature could guide adjuvant therapy for LuADC patients and include novel potential molecular targets for therapy.

## MATERIALS AND METHODS

### Data sets used in this study

Gene transcript data of normal and LuADC tissues was obtained from NCBI Gene Expression Omnibus (GEO) accession numbers: GSE31210, GSE19188 and GSE19804. Normal lung gene transcript data used for generating gene expression correlation networks were obtained from GTEx (http://www.gtexportal.org/home/datasets) using the RPKM normalized gene transcript counts table [15, 16].

### Statistical analysis

GEO2R was used to calculate the differential expression of tumor versus normal using a fold-change cut-off of 5 and adjusted *p*-value < 0.0001. Association of differentially expressed genes and OS in LuADC patients was assessed using Kaplan-Meier plotter (http://kmplot.com) including KM survival analysis, hazard ratio (HR) with 95% confidence intervals and logrank *p*-value for each gene [17]. The cytoscape plugin ClueGO was used to assess overrepresentation of Gene Ontology categories in biological networks (adjusted *p* < 0.001 was used as a threshold for significance) [18].

### Gene co-expression network construction

Gene expression Spearman correlation coefficients were calculated in “R” for 600 genes that were differentially expressed between LuADC and normal tissues samples and significantly associated with OS of LuADC patients. A gene network was generated where nodes represent individual genes and edges connecting nodes were drawn when the correlation coefficient exceeded R ≥ |0.7| (adjusted *p*-value ≤ 0.001). Gene co-expression networks were generated for normal lung gene expression data (GTEx) and lung adenocarcinoma (TCGA) and visualized using Cytoscape 3.4.0. (http://www.cytoscape.org). Dynet was used to highlight differences between two networks based on node and edge presence, ClueGO was used to identify significantly enriched biological pathways [18, 19].

### Gene expression signature-based prognostic risk score

100 random selections of 350 patients with LuADC were extracted from TCGA dataset and used as a training set to isolate a biomarker panel associated with OS. The remaining 167 patients for each selection were used as a test set to validate the prognostic significance of the biomarker panel. A forward-conditional Cox regression using all 600 genes as covariates was performed using SPSS on each of the training sets in order to isolate the biomarker panel. The results of each test were recorded and the genes that appeared in more than half of the training sets were included in our biomarker panel.

Cox regression was repeated on all 100 training sets using our 27-gene signature as covariates using the forced-entry (enter) method to obtain the co-efficient values for each biomarker. The resulting 100 co-efficient values of each biomarker were averaged to estimate the true co-efficient value of each gene. A prognostic scoring system was created based on this formula:

$$\sum_{i\;=\;1}^{27}(gene\;i\;co-efficient)\;x\;(gene\;i\;expression\;level)$$

The patients were ranked by their prognostic scores and divided into three equal sized cohorts. Kaplan-Meier plots were constructed and a long-rank test was used to determine differences in OS of LuADC patients.

Prognostic scores for each of the test set samples were then calculated using the same set of mean co-efficient values developed in the training set. Patients were ranked based on their prognostic scores and divided into three cohorts based on the average prognostic score at cut-point in the training sets. Kaplan-Meier plots were constructed and a long-rank test was used to determine differences among OS in all testing sets.

To further validate our biomarker panel, mRNA expression levels for the 27-gene signature were obtained from four additional datasets (GSE42127, GSE31210, GSE37745 and GSE30219). New coefficients for 27 genes were obtained from Cox regression. Prognostic scores for all patients were calculated and patients were ranked based on their scores and divided into three equal sized cohorts. Kaplan-Meier analysis and a long-rank test were used to determine differences in survival.

## SUPPLEMENTARY MATERIALS FIGURES AND TABLES









## Abbreviations

LuADC: lung adenocarcinoma
NSCLC: non-small cell lung cancer
OS: overall survival
TCGA: The Cancer Genome Atlas
GEO: Gene Expression Omnibus
GO: gene ontology
HR: hazard ratio.
