# Supplementary material for: A robust gene expression-based prognostic risk score predicts overall survival of lung adenocarcinoma patients
Source: Oncotarget. 2017 Dec 15;9(6):6862–71. doi: 10.18632/oncotarget.23490 (PMC5805521; doi:10.18632/oncotarget.23490)
Supplement: Supplementary file 1 [file oncotarget-09-6862-s001.pdf]

A robust gene expression-based prognostic risk score predicts overall survival of lung adenocarcinoma patients

SUPPLEMENTARY MATERIALS

Supplementary Table 1: List of genes that are deregulated in lung adenocarcinomas across three datasets using criteria: adjusted $p < 0.0001$ and fold change > 5. See Supplementary_Table_1

Supplementary Table 2: The impact of deregulated genes on overall survival (OS). See Supplementary_Table_2

Supplementary Table 3: Frequency of genes appeared in Cox regression model among 100 resampling test sets. See Supplementary_Table_3

Supplementary Table 4: The average Cox regression co-efficient for each gene is used to calculate prognostic score

Gene Name	Cox regression co-efficient
FAM83A	0.20995771
STK32A	−0.45049286
TRPC6	0.382016798
DEFA1B	0.298967835
TMEM47	0.220892566
CDC25C	0.338527972
PRKAR2B	0.035274941
TMEM100	0.101858155
CNTN4	0.120687495
HOOK1	0.079775222
INPP5A	−0.220656803
TRHDE	0.363592887
RSPO2	0.092585398
LDB3	0.127095987
SLC24A3	−0.336677565
VEPH1	0.164080783
SLC1A1	0.192834044
GPM6A	0.086279146
TMEM106B	0.105899244
FOXP1	0.249725361
NTN4	0.159188986
PALD1	0.167148577
F12	0.158275055
FHL1	−0.869024553
TIMP1	0.14597252
IGSF9	0.078902808
KLF9	0.32007008

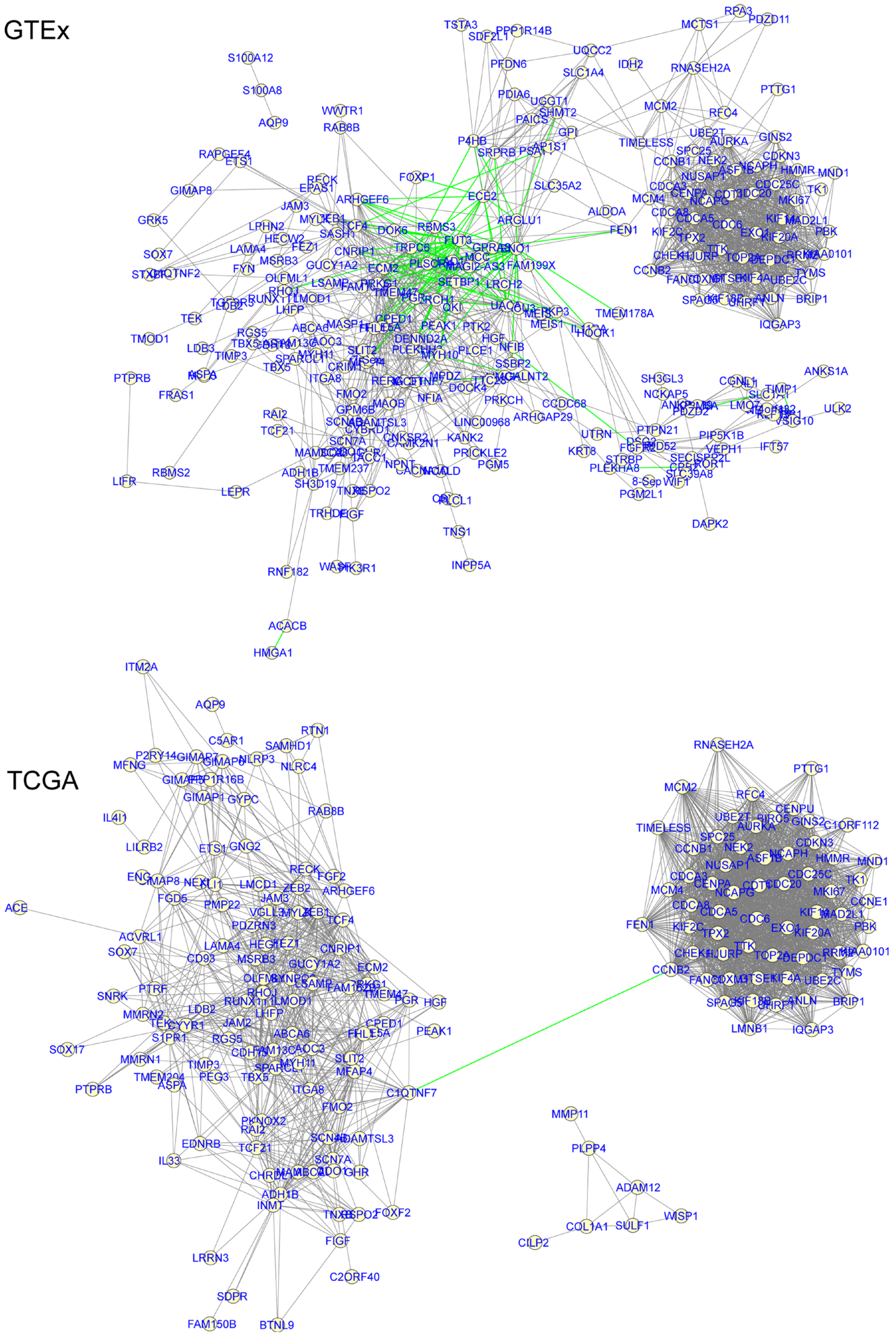

Supplementary Figure 1: Expression architectures of 600 genes in normal lung (top) and LuADCs (below) are revealed by gene correlation network analysis. Gray edges indicate positive correlations and green edges indicate negative correlations.

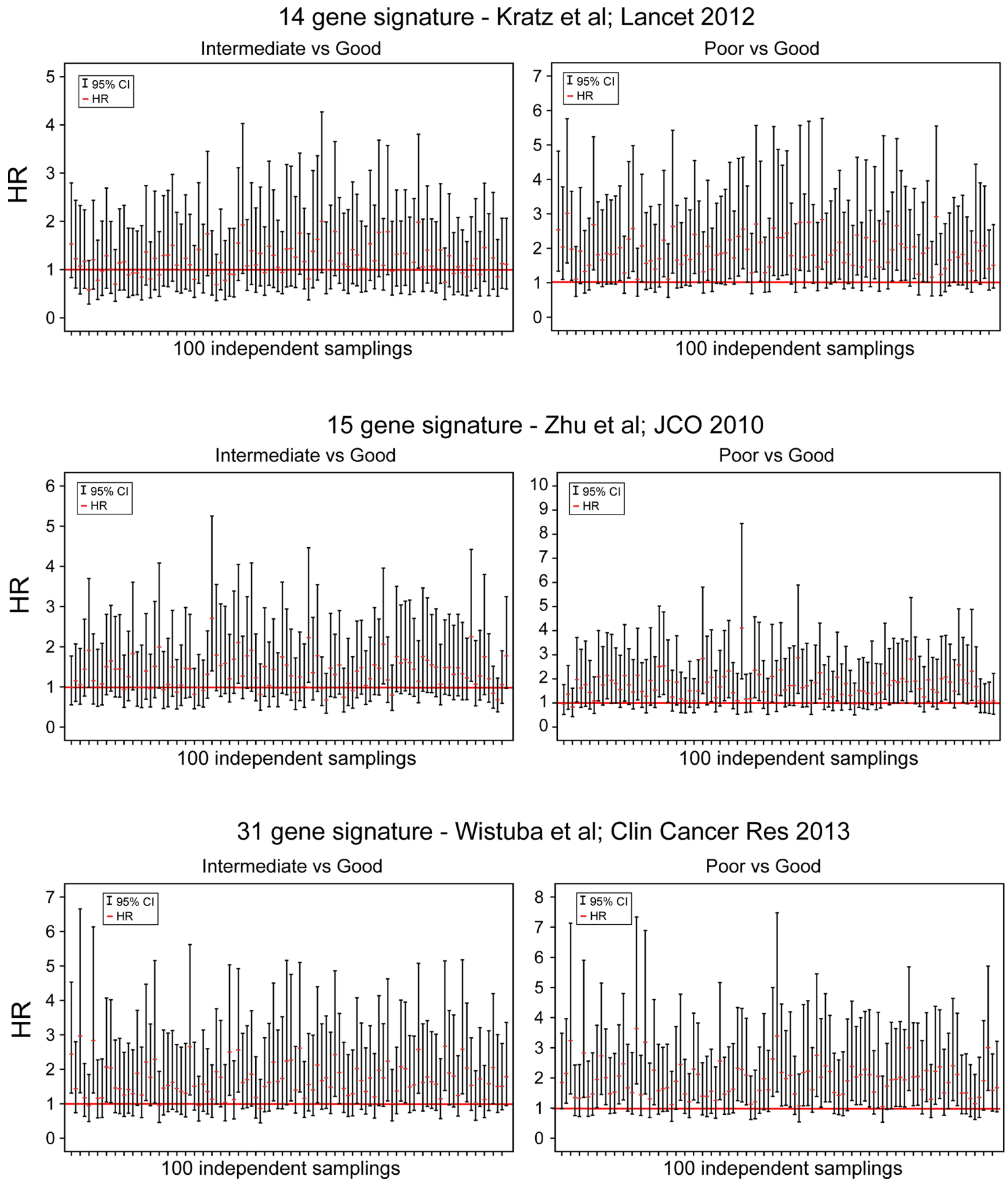

Supplementary Figure 2: For each of three existing gene signatures, the HR and the 95% confidence interval were calculated for each test set using a Cox model based on the prognostic score with groups (intermediate vs. good: left; poor vs. good: right). The red line indicates a HR value of 1, or the null hypothesis.
