# Supplementary material for: A robust gene expression-based prognostic risk score predicts overall survival of lung adenocarcinoma patients
Source: Oncotarget. 2017 Dec 15;9(6):6862–71. doi: 10.18632/oncotarget.23490 (PMC5805521; doi:10.18632/oncotarget.23490)
Supplement: Supplementary file 2 [file oncotarget-09-6862-s002.doc]

| **Supplementary Table 1:** List of genes that are deregulated in lung adenocarcinomas across three datasets using criteria: adjusted p<0.0001 and fold change >5 | | | | | | | | | | |
| --- | --- | --- | --- | --- | --- | --- | --- | --- | --- | --- |
| **Affymetrix probe ID** | **Gene symbol** | **GSE19188** | | | **GSE19804** | | | **GSE31210** | | |
|  |  | **adjusted P-Value** | **P-Value** | **logFC** | **adjusted P-Value** | **P-Value** | **logFC** | **adjusted P-Value** | **P-Value** | **logFC** |
| 210081_at | AGER | 2.35E-27 | 2.58E-31 | -4.10 | 1.15E-28 | 3.80E-32 | -4.40 | 1.20E-15 | 5.17E-18 | -4.62 |
| 203980_at | FABP4 | 1.14E-22 | 1.90E-25 | -3.97 | 3.04E-22 | 7.24E-25 | -3.75 | 3.04E-14 | 2.02E-16 | -3.87 |
| 232578_at | CLDN18 | 6.93E-22 | 1.69E-24 | -3.96 | 2.88E-16 | 3.30E-18 | -4.16 | 9.87E-07 | 6.88E-08 | -3.80 |
| 242009_at | SLC6A4 | 1.29E-18 | 1.19E-20 | -3.90 | 1.79E-19 | 8.85E-22 | -3.59 | 1.21E-12 | 1.25E-14 | -5.69 |
| 214135_at | CLDN18 | 4.54E-24 | 4.04E-27 | -3.87 | 3.43E-18 | 2.39E-20 | -3.23 | 3.23E-06 | 2.65E-07 | -3.54 |
| 209470_s_at | GPM6A | 2.51E-29 | 9.19E-34 | -3.86 | 3.52E-29 | 1.10E-32 | -4.20 | 1.20E-16 | 3.52E-19 | -3.71 |
| 219230_at | TMEM100 | 5.30E-21 | 1.98E-23 | -3.78 | 8.54E-18 | 6.70E-20 | -3.57 | 2.97E-12 | 3.54E-14 | -3.32 |
| 238222_at | GKN2 | 1.95E-26 | 5.35E-30 | -3.67 | 4.11E-17 | 3.82E-19 | -3.25 | 1.63E-09 | 4.86E-11 | -2.87 |
| 209613_s_at | ADH1B | 9.16E-18 | 1.09E-19 | -3.67 | 1.09E-14 | 1.80E-16 | -3.34 | 7.82E-05 | 9.93E-06 | -2.81 |
| 228885_at | MAMDC2 | 2.66E-25 | 1.32E-28 | -3.64 | 1.12E-12 | 2.87E-14 | -2.51 | 2.26E-07 | 1.27E-08 | -2.35 |
| 230560_at | STXBP6 | 9.49E-24 | 1.02E-26 | -3.63 | 2.51E-22 | 5.73E-25 | -3.63 | 2.26E-16 | 7.54E-19 | -3.31 |
| 205866_at | FCN3 | 4.54E-24 | 3.91E-27 | -3.59 | 3.00E-20 | 1.21E-22 | -3.41 | 1.83E-10 | 4.00E-12 | -2.84 |
| 209469_at | GPM6A | 2.51E-29 | 5.54E-34 | -3.51 | 4.16E-28 | 1.75E-31 | -3.62 | 1.21E-12 | 1.24E-14 | -3.71 |
| 206651_s_at | CPB2 | 1.75E-20 | 8.04E-23 | -3.50 | 1.44E-12 | 3.78E-14 | -2.80 | 1.60E-05 | 1.63E-06 | -3.30 |
| 204712_at | WIF1 | 2.25E-21 | 7.01E-24 | -3.48 | 1.73E-14 | 2.98E-16 | -3.77 | 4.22E-06 | 3.59E-07 | -3.26 |
| 206209_s_at | CA4 | 6.56E-25 | 4.20E-28 | -3.48 | 4.14E-31 | 6.82E-35 | -3.87 | 4.59E-19 | 5.29E-22 | -3.83 |
| 213317_at | CLIC5 | 4.79E-24 | 4.47E-27 | -3.43 | 1.92E-22 | 4.22E-25 | -3.46 | 7.52E-10 | 2.03E-11 | -2.60 |
| 229309_at | ADRB1 | 3.82E-20 | 2.07E-22 | -3.41 | 1.25E-17 | 1.03E-19 | -3.21 | 1.37E-10 | 2.91E-12 | -2.81 |
| 215454_x_at | SFTPC | 1.95E-24 | 1.39E-27 | -3.40 | 1.04E-17 | 8.39E-20 | -3.27 | 2.04E-09 | 6.29E-11 | -4.36 |
| 235568_at | MCEMP1 | 3.03E-22 | 6.59E-25 | -3.36 | 5.18E-19 | 2.92E-21 | -2.71 | 3.17E-10 | 7.57E-12 | -2.77 |
| 206742_at | FIGF | 6.40E-25 | 3.89E-28 | -3.34 | 1.35E-20 | 4.97E-23 | -2.86 | 1.77E-06 | 1.33E-07 | -2.65 |
| 209074_s_at | FAM107A | 1.79E-23 | 2.26E-26 | -3.32 | 6.01E-25 | 6.37E-28 | -3.44 | 1.67E-12 | 1.78E-14 | -3.30 |
| 235649_at | ADAMTS8 | 5.70E-27 | 8.33E-31 | -3.28 | 1.73E-21 | 5.21E-24 | -2.22 | 4.55E-07 | 2.84E-08 | -2.92 |
| 232122_s_at | VEPH1 | 2.35E-27 | 2.24E-31 | -3.26 | 5.19E-12 | 1.56E-13 | -1.95 | 9.49E-05 | 1.24E-05 | -1.69 |
| 204719_at | ABCA8 | 8.90E-23 | 1.43E-25 | -3.26 | 2.30E-16 | 2.60E-18 | -2.81 | 2.54E-08 | 1.07E-09 | -2.38 |
| 210299_s_at | FHL1 | 2.72E-22 | 5.72E-25 | -3.24 | 2.49E-18 | 1.69E-20 | -2.96 | 3.01E-12 | 3.58E-14 | -2.81 |
| 204273_at | EDNRB | 1.21E-22 | 2.05E-25 | -3.23 | 6.92E-22 | 1.87E-24 | -3.19 | 4.60E-17 | 1.18E-19 | -2.83 |
| 223678_s_at | SFTPA2///SFTPA1 | 6.31E-12 | 3.22E-13 | -3.21 | 6.70E-10 | 3.24E-11 | -3.20 | 1.95E-06 | 1.49E-07 | -3.38 |
| 228766_at | CD36 | 2.02E-21 | 6.18E-24 | -3.19 | 3.58E-20 | 1.47E-22 | -3.37 | 1.10E-13 | 8.51E-16 | -3.00 |
| 228434_at | BTNL9 | 1.85E-25 | 7.46E-29 | -3.17 | 2.79E-24 | 3.77E-27 | -2.92 | 1.20E-18 | 1.70E-21 | -3.61 |
| 209904_at | TNNC1 | 4.09E-25 | 2.24E-28 | -3.16 | 3.87E-27 | 2.34E-30 | -3.12 | 1.15E-08 | 4.37E-10 | -3.64 |
| 228504_at | SCN7A | 1.82E-23 | 2.36E-26 | -3.12 | 6.14E-12 | 1.87E-13 | -2.21 | 5.00E-06 | 4.35E-07 | -1.78 |
| 205200_at | CLEC3B | 9.73E-23 | 1.58E-25 | -3.06 | 2.47E-27 | 1.36E-30 | -2.88 | 4.62E-19 | 5.58E-22 | -2.96 |
| 217046_s_at | AGER | 1.67E-26 | 4.27E-30 | -3.03 | 6.23E-36 | 2.05E-40 | -3.46 | 2.44E-16 | 8.20E-19 | -5.06 |
| 227848_at | PEBP4 | 2.99E-20 | 1.53E-22 | -3.01 | 2.12E-13 | 4.59E-15 | -2.27 | 6.23E-05 | 7.64E-06 | -2.03 |
| 207519_at | SLC6A4 | 6.62E-17 | 1.00E-18 | -3.01 | 9.08E-18 | 7.19E-20 | -2.59 | 6.31E-13 | 6.01E-15 | -3.42 |
| 213456_at | SOSTDC1 | 7.18E-16 | 1.48E-17 | -2.99 | 6.92E-18 | 5.32E-20 | -3.39 | 9.99E-13 | 1.00E-14 | -5.42 |
| 211699_x_at | HBA2///HBA1 | 1.83E-18 | 1.79E-20 | -2.97 | 1.33E-16 | 1.41E-18 | -2.56 | 9.84E-15 | 5.45E-17 | -3.00 |
| 227874_at | EMCN | 1.29E-23 | 1.46E-26 | -2.95 | 2.22E-24 | 2.85E-27 | -2.92 | 2.22E-17 | 5.21E-20 | -2.47 |
| 219866_at | CLIC5 | 2.17E-25 | 9.52E-29 | -2.95 | 4.30E-19 | 2.35E-21 | -2.66 | 9.02E-11 | 1.81E-12 | -2.72 |
| 204931_at | TCF21 | 8.84E-23 | 1.41E-25 | -2.93 | 1.61E-21 | 4.81E-24 | -2.62 | 4.99E-11 | 9.19E-13 | -2.67 |
| 230469_at | RTKN2 | 1.86E-19 | 1.25E-21 | -2.92 | 8.87E-33 | 6.49E-37 | -3.41 | 2.32E-21 | 1.05E-24 | -3.70 |
| 228268_at | FMO2 | 1.07E-21 | 2.79E-24 | -2.86 | 4.25E-22 | 1.05E-24 | -2.54 | 2.35E-11 | 3.97E-13 | -1.99 |
| 209458_x_at | HBA2///HBA1 | 1.33E-18 | 1.24E-20 | -2.86 | 1.90E-16 | 2.07E-18 | -2.46 | 5.87E-13 | 5.56E-15 | -3.09 |
| 214146_s_at | PPBP | 3.70E-17 | 5.26E-19 | -2.85 | 3.57E-11 | 1.29E-12 | -2.43 | 5.39E-07 | 3.47E-08 | -2.70 |
| 222717_at | SDPR | 6.74E-21 | 2.66E-23 | -2.83 | 1.49E-21 | 4.37E-24 | -2.89 | 3.79E-13 | 3.43E-15 | -2.60 |
| 203571_s_at | ADIRF | 9.46E-22 | 2.42E-24 | -2.82 | 1.15E-23 | 1.86E-26 | -2.57 | 1.48E-09 | 4.36E-11 | -1.96 |
| 221133_s_at | CLDN18 | 1.95E-17 | 2.59E-19 | -2.82 | 3.11E-15 | 4.56E-17 | -2.90 | 2.60E-07 | 1.50E-08 | -3.33 |
| 209116_x_at | HBB | 2.33E-19 | 1.63E-21 | -2.81 | 3.98E-15 | 5.96E-17 | -2.60 | 6.88E-10 | 1.84E-11 | -2.84 |
| 230135_at | HHIP | 1.75E-20 | 8.05E-23 | -2.81 | 5.56E-14 | 1.07E-15 | -2.34 | 1.87E-07 | 1.02E-08 | -3.06 |
| 227198_at | AFF3 | 1.49E-19 | 9.66E-22 | -2.80 | 2.23E-16 | 2.50E-18 | -2.27 | 4.22E-09 | 1.44E-10 | -2.25 |
| 230360_at | GLDN | 9.83E-24 | 1.08E-26 | -2.79 | 1.23E-05 | 1.57E-06 | -1.05 | 1.24E-05 | 1.22E-06 | -1.44 |
| 201540_at | FHL1 | 1.89E-23 | 2.49E-26 | -2.78 | 1.55E-19 | 7.38E-22 | -2.35 | 5.44E-07 | 3.50E-08 | -1.50 |
| 217232_x_at | HBB | 6.18E-20 | 3.64E-22 | -2.77 | 3.35E-16 | 3.90E-18 | -2.47 | 6.04E-10 | 1.58E-11 | -2.63 |
| 205952_at | KCNK3 | 1.41E-23 | 1.63E-26 | -2.77 | 1.63E-19 | 7.91E-22 | -2.64 | 3.72E-15 | 1.82E-17 | -2.90 |
| 204018_x_at | HBA2///HBA1 | 1.20E-18 | 1.09E-20 | -2.76 | 5.70E-17 | 5.50E-19 | -2.41 | 1.46E-13 | 1.18E-15 | -2.94 |
| 224061_at | INMT | 1.78E-23 | 2.21E-26 | -2.75 | 3.83E-18 | 2.70E-20 | -2.53 | 1.19E-09 | 3.41E-11 | -2.30 |
| 217414_x_at | HBA2///HBA1 | 1.15E-18 | 1.04E-20 | -2.75 | 4.57E-17 | 4.31E-19 | -2.41 | 1.35E-13 | 1.09E-15 | -3.07 |
| 1556579_s_at | IGSF10 | 1.16E-25 | 4.34E-29 | -2.75 | 1.40E-26 | 9.72E-30 | -2.86 | 2.43E-14 | 1.55E-16 | -3.22 |
| 209763_at | CHRDL1 | 3.03E-22 | 6.57E-25 | -2.74 | 6.89E-13 | 1.68E-14 | -2.30 | 1.70E-05 | 1.74E-06 | -1.94 |
| 211745_x_at | HBA2///HBA1 | 9.55E-19 | 8.38E-21 | -2.73 | 1.17E-16 | 1.22E-18 | -2.40 | 1.45E-12 | 1.52E-14 | -2.95 |
| 229839_at | SCARA5 | 2.96E-18 | 3.11E-20 | -2.73 | 2.72E-14 | 4.90E-16 | -2.15 | 6.24E-08 | 2.97E-09 | -3.18 |
| 228698_at | SOX7 | 4.48E-17 | 6.48E-19 | -2.72 | 1.17E-18 | 7.33E-21 | -2.75 | 1.62E-12 | 1.72E-14 | -2.40 |
| 220677_s_at | ADAMTS8 | 5.04E-26 | 1.47E-29 | -2.71 | 6.70E-24 | 1.07E-26 | -1.72 | 9.33E-10 | 2.58E-11 | -2.25 |
| 217546_at | MT1M | 9.33E-14 | 3.17E-15 | -2.70 | 2.52E-15 | 3.62E-17 | -3.12 | 3.84E-16 | 1.41E-18 | -2.97 |
| 206701_x_at | EDNRB | 1.75E-22 | 3.23E-25 | -2.70 | 4.25E-22 | 1.06E-24 | -2.93 | 3.51E-19 | 3.96E-22 | -2.62 |
| 229584_at | LRRK2 | 4.83E-20 | 2.75E-22 | -2.69 | 8.48E-11 | 3.37E-12 | -2.10 | 6.45E-05 | 7.96E-06 | -1.70 |
| 209614_at | ADH1B | 1.50E-26 | 3.56E-30 | -2.67 | 1.02E-19 | 4.65E-22 | -2.23 | 8.59E-08 | 4.28E-09 | -2.90 |
| 238018_at | FAM150B | 2.42E-22 | 4.95E-25 | -2.67 | 6.88E-17 | 6.82E-19 | -2.62 | 1.05E-14 | 6.02E-17 | -2.95 |
| 239849_at |  | 4.10E-20 | 2.25E-22 | -2.66 | 1.28E-11 | 4.18E-13 | -1.48 | 1.25E-14 | 7.29E-17 | -2.74 |
| 207547_s_at | FAM107A | 2.26E-25 | 1.07E-28 | -2.65 | 1.18E-25 | 1.02E-28 | -2.65 | 1.70E-18 | 2.62E-21 | -2.61 |
| 219597_s_at | DUOX1 | 1.06E-24 | 7.15E-28 | -2.65 | 1.30E-15 | 1.73E-17 | -1.93 | 2.03E-06 | 1.56E-07 | -2.28 |
| 229308_at | ANKRD29 | 5.54E-21 | 2.11E-23 | -2.65 | 4.48E-22 | 1.14E-24 | -2.31 | 6.76E-10 | 1.80E-11 | -2.67 |
| 211726_s_at | FMO2 | 3.43E-21 | 1.20E-23 | -2.65 | 8.03E-17 | 8.03E-19 | -2.40 | 1.32E-10 | 2.77E-12 | -1.94 |
| 204731_at | TGFBR3 | 7.44E-24 | 7.48E-27 | -2.65 | 3.81E-16 | 4.52E-18 | -2.16 | 2.79E-13 | 2.40E-15 | -2.14 |
| 204271_s_at | EDNRB | 1.04E-21 | 2.69E-24 | -2.64 | 1.18E-25 | 9.93E-29 | -2.77 | 1.90E-20 | 1.18E-23 | -2.42 |
| 206069_s_at | ACADL | 3.20E-25 | 1.70E-28 | -2.64 | 1.33E-20 | 4.88E-23 | -2.05 | 8.55E-11 | 1.70E-12 | -3.04 |
| 223623_at | C2orf40 | 6.86E-15 | 1.80E-16 | -2.64 | 7.00E-14 | 1.37E-15 | -2.29 | 6.58E-05 | 8.14E-06 | -2.37 |
| 226625_at | TGFBR3 | 6.40E-25 | 3.95E-28 | -2.62 | 1.83E-17 | 1.59E-19 | -2.11 | 3.06E-13 | 2.66E-15 | -2.00 |
| 211696_x_at | HBB | 1.33E-19 | 8.48E-22 | -2.61 | 1.23E-14 | 2.04E-16 | -2.31 | 8.30E-09 | 3.03E-10 | -2.38 |
| 205433_at | BCHE | 3.56E-18 | 3.77E-20 | -2.61 | 1.28E-12 | 3.33E-14 | -2.46 | 2.74E-08 | 1.17E-09 | -2.51 |
| 222722_at | OGN | 3.56E-13 | 1.38E-14 | -2.59 | 3.91E-11 | 1.43E-12 | -2.19 | 2.23E-06 | 1.74E-07 | -2.26 |
| 206702_at | TEK | 2.59E-24 | 2.00E-27 | -2.57 | 2.85E-24 | 3.91E-27 | -2.60 | 4.78E-19 | 5.85E-22 | -2.31 |
| 206068_s_at | ACADL | 8.57E-24 | 8.94E-27 | -2.56 | 5.96E-18 | 4.47E-20 | -2.05 | 4.07E-09 | 1.38E-10 | -2.39 |
| 241811_x_at | SLC6A4 | 1.53E-12 | 6.77E-14 | -2.55 | 1.70E-17 | 1.45E-19 | -2.44 | 2.45E-12 | 2.81E-14 | -4.60 |
| 230250_at | PTPRB | 1.48E-20 | 6.67E-23 | -2.55 | 4.81E-21 | 1.57E-23 | -2.54 | 5.11E-17 | 1.33E-19 | -2.23 |
| 209292_at | ID4 | 6.05E-19 | 4.86E-21 | -2.55 | 6.82E-12 | 2.10E-13 | -1.86 | 4.13E-10 | 1.03E-11 | -2.06 |
| 220244_at | LINC00312 | 3.55E-23 | 5.13E-26 | -2.53 | 1.39E-16 | 1.47E-18 | -1.75 | 1.10E-14 | 6.30E-17 | -1.70 |
| 214414_x_at | HBA2///HBA1 | 2.71E-18 | 2.81E-20 | -2.53 | 1.78E-15 | 2.47E-17 | -2.11 | 3.68E-09 | 1.23E-10 | -2.32 |
| 230867_at | COL6A6 | 1.52E-19 | 9.97E-22 | -2.53 | 1.21E-17 | 9.96E-20 | -2.31 | 5.57E-08 | 2.63E-09 | -2.60 |
| 221132_at | CLDN18 | 1.11E-15 | 2.39E-17 | -2.51 | 3.64E-13 | 8.39E-15 | -2.74 | 3.30E-10 | 7.96E-12 | -3.60 |
| 235670_at | STX11 | 6.00E-26 | 1.87E-29 | -2.51 | 3.75E-25 | 3.66E-28 | -2.69 | 2.14E-17 | 4.93E-20 | -2.04 |
| 206159_at | GDF10 | 2.35E-21 | 7.43E-24 | -2.51 | 9.93E-19 | 6.07E-21 | -1.97 | 2.63E-10 | 6.07E-12 | -2.56 |
| 203323_at | CAV2 | 1.07E-20 | 4.56E-23 | -2.51 | 1.45E-17 | 1.23E-19 | -2.34 | 6.44E-15 | 3.43E-17 | -2.04 |
| 203065_s_at | CAV1 | 4.65E-20 | 2.64E-22 | -2.49 | 1.46E-22 | 3.07E-25 | -2.65 | 2.07E-15 | 9.62E-18 | -2.15 |
| 209840_s_at | LRRN3 | 3.23E-17 | 4.53E-19 | -2.46 | 1.20E-18 | 7.50E-21 | -2.27 | 2.25E-11 | 3.77E-13 | -2.65 |
| 209493_at | PDZD2 | 3.15E-21 | 1.08E-23 | -2.45 | 1.38E-14 | 2.34E-16 | -1.92 | 8.75E-11 | 1.75E-12 | -1.86 |
| 239349_at | C1QTNF7 | 2.72E-20 | 1.38E-22 | -2.45 | 6.37E-12 | 1.95E-13 | -1.66 | 2.78E-05 | 3.05E-06 | -1.49 |
| 1558444_at |  | 2.13E-18 | 2.14E-20 | -2.45 | 1.54E-11 | 5.12E-13 | -1.41 | 7.94E-11 | 1.55E-12 | -2.33 |
| 205357_s_at | AGTR1 | 3.85E-20 | 2.10E-22 | -2.44 | 6.38E-17 | 6.23E-19 | -2.10 | 8.56E-15 | 4.67E-17 | -2.83 |
| 236936_at |  | 3.33E-23 | 4.69E-26 | -2.44 | 5.17E-16 | 6.42E-18 | -2.40 | 2.87E-13 | 2.48E-15 | -2.43 |
| 213451_x_at | TNXB///TNXA | 4.93E-27 | 6.31E-31 | -2.44 | 3.25E-16 | 3.77E-18 | -1.72 | 6.64E-13 | 6.38E-15 | -2.56 |
| 215918_s_at | SPTBN1 | 1.50E-26 | 3.57E-30 | -2.42 | 6.23E-36 | 2.28E-40 | -2.51 | 1.70E-21 | 6.22E-25 | -3.05 |
| 206488_s_at | CD36 | 6.31E-19 | 5.10E-21 | -2.41 | 2.10E-17 | 1.83E-19 | -2.63 | 2.90E-13 | 2.50E-15 | -2.47 |
| 227826_s_at |  | 3.55E-20 | 1.89E-22 | -2.40 | 2.24E-13 | 4.89E-15 | -1.86 | 8.03E-09 | 2.92E-10 | -2.12 |
| 209555_s_at | CD36 | 1.23E-17 | 1.54E-19 | -2.40 | 4.38E-16 | 5.32E-18 | -2.65 | 2.00E-10 | 4.44E-12 | -2.35 |
| 203549_s_at | LPL | 1.02E-16 | 1.64E-18 | -2.40 | 8.51E-15 | 1.37E-16 | -1.70 | 3.30E-10 | 7.94E-12 | -1.90 |
| 214505_s_at | FHL1 | 4.67E-17 | 6.79E-19 | -2.39 | 2.16E-16 | 2.42E-18 | -2.48 | 1.86E-14 | 1.15E-16 | -1.97 |
| 1556037_s_at | HHIP | 2.01E-22 | 3.90E-25 | -2.39 | 6.56E-15 | 1.04E-16 | -1.97 | 4.85E-07 | 3.07E-08 | -2.54 |
| 218711_s_at | SDPR | 1.79E-17 | 2.35E-19 | -2.39 | 8.89E-15 | 1.44E-16 | -2.51 | 3.35E-16 | 1.20E-18 | -2.28 |
| 207526_s_at | IL1RL1 | 8.66E-10 | 6.84E-11 | -2.39 | 9.28E-12 | 2.95E-13 | -2.87 | 2.12E-07 | 1.18E-08 | -2.47 |
| 225078_at | EMP2 | 1.16E-21 | 3.24E-24 | -2.38 | 5.34E-22 | 1.40E-24 | -1.87 | 8.42E-10 | 2.31E-11 | -1.30 |
| 206167_s_at | ARHGAP6 | 8.29E-25 | 5.46E-28 | -2.37 | 2.16E-20 | 8.38E-23 | -2.10 | 1.50E-16 | 4.72E-19 | -1.98 |
| 216333_x_at | TNXB///TNXA | 9.11E-28 | 5.00E-32 | -2.37 | 3.68E-16 | 4.34E-18 | -1.71 | 4.17E-13 | 3.82E-15 | -2.54 |
| 205207_at | IL6 | 4.03E-09 | 3.69E-10 | -2.37 | 3.34E-12 | 9.54E-14 | -2.84 | 8.52E-10 | 2.34E-11 | -2.72 |
| 227662_at | SYNPO2 | 1.10E-17 | 1.35E-19 | -2.36 | 7.32E-10 | 3.57E-11 | -1.70 | 4.59E-06 | 3.95E-07 | -1.63 |
| 212097_at | CAV1 | 1.73E-22 | 3.14E-25 | -2.35 | 2.32E-20 | 9.09E-23 | -2.21 | 2.36E-11 | 3.99E-13 | -1.46 |
| 203548_s_at | LPL | 5.89E-15 | 1.52E-16 | -2.35 | 1.14E-11 | 3.68E-13 | -1.83 | 3.08E-10 | 7.29E-12 | -1.94 |
| 206170_at | ADRB2 | 2.05E-22 | 4.01E-25 | -2.35 | 3.24E-20 | 1.31E-22 | -1.96 | 3.86E-06 | 3.25E-07 | -1.24 |
| 244455_at | KCNT2 | 1.69E-23 | 2.07E-26 | -2.35 | 1.04E-22 | 2.09E-25 | -2.16 | 5.80E-15 | 3.04E-17 | -2.10 |
| 219295_s_at | PCOLCE2 | 1.37E-16 | 2.33E-18 | -2.35 | 6.88E-14 | 1.35E-15 | -2.02 | 4.46E-12 | 5.74E-14 | -2.76 |
| 205554_s_at | DNASE1L3 | 3.70E-17 | 5.24E-19 | -2.34 | 2.27E-18 | 1.52E-20 | -1.87 | 1.66E-09 | 4.95E-11 | -1.99 |
| 238178_at |  | 7.86E-21 | 3.21E-23 | -2.34 | 1.29E-16 | 1.36E-18 | -1.82 | 2.85E-17 | 6.89E-20 | -2.14 |
| 227827_at |  | 7.56E-19 | 6.39E-21 | -2.34 | 2.53E-13 | 5.61E-15 | -2.00 | 1.97E-07 | 1.09E-08 | -1.97 |
| 205819_at | MARCO | 2.06E-18 | 2.03E-20 | -2.33 | 3.20E-15 | 4.71E-17 | -1.96 | 3.14E-09 | 1.03E-10 | -2.58 |
| 243813_at | LINC00968 | 8.09E-21 | 3.33E-23 | -2.33 | 1.80E-23 | 3.17E-26 | -2.33 | 8.36E-12 | 1.20E-13 | -2.71 |
| 205019_s_at | VIPR1 | 2.60E-17 | 3.58E-19 | -2.33 | 3.30E-15 | 4.88E-17 | -1.90 | 3.22E-09 | 1.06E-10 | -2.11 |
| 227197_at | ARHGEF26 | 4.73E-21 | 1.74E-23 | -2.32 | 1.55E-21 | 4.57E-24 | -2.40 | 1.10E-16 | 3.15E-19 | -2.02 |
| 212713_at | MFAP4 | 1.79E-18 | 1.73E-20 | -2.32 | 3.69E-17 | 3.39E-19 | -2.02 | 7.07E-07 | 4.71E-08 | -2.29 |
| 235666_at | ITGA8 | 6.61E-21 | 2.60E-23 | -2.32 | 7.93E-16 | 1.02E-17 | -1.66 | 1.68E-09 | 5.05E-11 | -1.69 |
| 230130_at | SLIT2 | 1.02E-17 | 1.24E-19 | -2.32 | 1.37E-15 | 1.85E-17 | -2.01 | 2.47E-11 | 4.20E-13 | -1.67 |
| 205935_at | FOXF1 | 1.39E-21 | 4.04E-24 | -2.32 | 3.01E-21 | 9.53E-24 | -2.01 | 1.70E-21 | 6.61E-25 | -2.45 |
| 226028_at | ROBO4 | 4.84E-23 | 7.17E-26 | -2.32 | 3.38E-27 | 1.92E-30 | -2.39 | 3.38E-20 | 2.41E-23 | -2.60 |
| 206093_x_at | TNXB///TNXA | 2.35E-27 | 1.97E-31 | -2.32 | 2.53E-15 | 3.63E-17 | -1.63 | 2.10E-13 | 1.76E-15 | -2.56 |
| 213900_at | FAM189A2 | 2.42E-23 | 3.28E-26 | -2.31 | 3.47E-23 | 6.66E-26 | -1.80 | 1.17E-11 | 1.79E-13 | -2.02 |
| 205471_s_at | DACH1 | 3.43E-21 | 1.19E-23 | -2.31 | 3.66E-17 | 3.36E-19 | -2.02 | 9.04E-16 | 3.77E-18 | -1.94 |
| 228568_at | GCOM1 | 9.46E-22 | 2.41E-24 | -2.30 | 6.65E-25 | 7.18E-28 | -2.31 | 4.12E-14 | 2.83E-16 | -1.85 |
| 219436_s_at | EMCN | 1.34E-18 | 1.25E-20 | -2.30 | 1.74E-16 | 1.88E-18 | -2.30 | 5.53E-17 | 1.49E-19 | -2.16 |
| 219689_at | SEMA3G | 4.31E-20 | 2.42E-22 | -2.30 | 3.28E-17 | 2.96E-19 | -1.85 | 5.39E-19 | 6.90E-22 | -2.09 |
| 229641_at | CCBE1 | 5.63E-22 | 1.35E-24 | -2.30 | 2.15E-29 | 6.30E-33 | -2.33 | 8.13E-14 | 6.10E-16 | -3.00 |
| 201539_s_at | FHL1 | 1.46E-16 | 2.49E-18 | -2.30 | 2.19E-15 | 3.10E-17 | -2.47 | 2.36E-14 | 1.50E-16 | -2.50 |
| 224822_at | DLC1 | 1.02E-19 | 6.31E-22 | -2.30 | 1.58E-18 | 1.01E-20 | -1.90 | 1.01E-08 | 3.78E-10 | -1.47 |
| 210004_at | OLR1 | 2.62E-16 | 4.82E-18 | -2.30 | 4.35E-07 | 3.90E-08 | -1.46 | 2.41E-06 | 1.90E-07 | -1.20 |
| 228854_at |  | 2.31E-09 | 1.99E-10 | -2.29 | 3.57E-11 | 1.29E-12 | -2.52 | 9.26E-09 | 3.44E-10 | -2.59 |
| 230670_at | IGSF10 | 6.02E-26 | 1.98E-29 | -2.29 | 8.96E-25 | 1.02E-27 | -1.88 | 6.96E-14 | 5.08E-16 | -2.97 |
| 213974_at | ADAMTSL3 | 1.89E-22 | 3.60E-25 | -2.27 | 6.51E-19 | 3.80E-21 | -2.15 | 2.74E-16 | 9.36E-19 | -2.52 |
| 223395_at | ABI3BP | 4.66E-19 | 3.63E-21 | -2.27 | 1.25E-16 | 1.31E-18 | -2.09 | 1.60E-08 | 6.34E-10 | -1.76 |
| 210298_x_at | FHL1 | 6.42E-16 | 1.30E-17 | -2.27 | 8.00E-16 | 1.03E-17 | -2.48 | 1.35E-13 | 1.08E-15 | -2.48 |
| 226992_at | NOSTRIN | 5.93E-23 | 9.12E-26 | -2.27 | 7.41E-15 | 1.18E-16 | -1.81 | 2.60E-12 | 3.02E-14 | -2.02 |
| 229127_at | JAM2 | 7.72E-24 | 7.91E-27 | -2.27 | 1.79E-19 | 8.82E-22 | -2.20 | 2.33E-14 | 1.47E-16 | -2.01 |
| 205569_at | LAMP3 | 2.08E-22 | 4.12E-25 | -2.27 | 1.80E-17 | 1.55E-19 | -1.89 | 9.10E-08 | 4.57E-09 | -1.75 |
| 209841_s_at | LRRN3 | 1.58E-17 | 2.06E-19 | -2.26 | 2.10E-20 | 8.03E-23 | -2.11 | 3.99E-12 | 5.07E-14 | -2.26 |
| 1562921_at | EP300-AS1 | 2.95E-21 | 9.98E-24 | -2.25 | 1.45E-08 | 9.36E-10 | -1.41 | 4.32E-05 | 5.04E-06 | -1.40 |
| 238062_at | GPIHBP1 | 1.12E-20 | 4.86E-23 | -2.25 | 6.65E-27 | 4.26E-30 | -2.29 | 1.65E-10 | 3.58E-12 | -2.92 |
| 224013_s_at | SOX7 | 1.93E-15 | 4.46E-17 | -2.25 | 5.46E-17 | 5.25E-19 | -2.47 | 1.47E-13 | 1.19E-15 | -1.82 |
| 220327_at | VGLL3 | 1.83E-21 | 5.50E-24 | -2.25 | 1.71E-15 | 2.35E-17 | -1.93 | 2.53E-08 | 1.07E-09 | -1.65 |
| 210906_x_at | AQP4 | 5.22E-16 | 1.03E-17 | -2.24 | 6.30E-14 | 1.22E-15 | -2.37 | 5.63E-06 | 4.99E-07 | -2.12 |
| 229985_at |  | 2.47E-18 | 2.53E-20 | -2.24 | 3.59E-10 | 1.63E-11 | -1.35 | 1.37E-15 | 6.04E-18 | -3.40 |
| 204975_at | EMP2 | 5.77E-21 | 2.21E-23 | -2.23 | 1.78E-20 | 6.70E-23 | -1.70 | 5.15E-12 | 6.79E-14 | -1.75 |
| 236383_at |  | 1.40E-22 | 2.40E-25 | -2.23 | 2.57E-22 | 5.97E-25 | -1.96 | 3.14E-21 | 1.61E-24 | -2.43 |
| 226492_at | SEMA6D | 4.25E-18 | 4.58E-20 | -2.22 | 8.59E-17 | 8.63E-19 | -1.73 | 3.00E-14 | 1.99E-16 | -1.84 |
| 204154_at | CDO1 | 1.36E-20 | 6.11E-23 | -2.22 | 1.01E-17 | 8.09E-20 | -2.32 | 8.84E-14 | 6.74E-16 | -2.43 |
| 235108_at | KCNK3 | 6.40E-25 | 3.73E-28 | -2.22 | 1.27E-24 | 1.48E-27 | -2.00 | 4.65E-20 | 3.66E-23 | -2.82 |
| 203887_s_at | THBD | 9.35E-17 | 1.48E-18 | -2.22 | 1.43E-18 | 9.07E-21 | -2.00 | 3.84E-16 | 1.40E-18 | -1.98 |
| 205498_at | GHR | 3.59E-21 | 1.27E-23 | -2.21 | 2.77E-15 | 4.02E-17 | -1.86 | 3.17E-12 | 3.83E-14 | -1.94 |
| 206481_s_at | LDB2 | 3.55E-21 | 1.25E-23 | -2.21 | 5.21E-22 | 1.34E-24 | -2.14 | 1.04E-14 | 5.87E-17 | -1.88 |
| 226872_at | RFX2 | 2.07E-19 | 1.41E-21 | -2.19 | 3.67E-10 | 1.67E-11 | -1.13 | 5.64E-06 | 5.01E-07 | -0.93 |
| 228850_s_at | SLIT2 | 4.17E-22 | 9.46E-25 | -2.19 | 6.74E-17 | 6.67E-19 | -1.84 | 3.76E-12 | 4.70E-14 | -1.52 |
| 220170_at | FHL5 | 1.63E-22 | 2.93E-25 | -2.18 | 4.95E-17 | 4.70E-19 | -1.95 | 4.72E-16 | 1.77E-18 | -2.52 |
| 225895_at | SYNPO2 | 7.40E-21 | 2.97E-23 | -2.18 | 9.03E-10 | 4.49E-11 | -1.52 | 5.43E-08 | 2.55E-09 | -1.62 |
| 209220_at | GPC3 | 1.28E-16 | 2.17E-18 | -2.18 | 2.70E-15 | 3.91E-17 | -1.99 | 6.28E-10 | 1.65E-11 | -1.92 |
| 222885_at | EMCN | 1.67E-19 | 1.10E-21 | -2.18 | 9.29E-22 | 2.57E-24 | -2.22 | 1.15E-17 | 2.42E-20 | -1.88 |
| 209894_at | LEPR | 1.28E-13 | 4.48E-15 | -2.18 | 1.98E-16 | 2.18E-18 | -1.89 | 9.88E-13 | 9.88E-15 | -1.97 |
| 203888_at | THBD | 2.76E-18 | 2.87E-20 | -2.18 | 4.49E-20 | 1.88E-22 | -1.96 | 1.58E-16 | 5.04E-19 | -2.08 |
| 204343_at | ABCA3 | 7.34E-21 | 2.92E-23 | -2.17 | 2.65E-10 | 1.17E-11 | -1.29 | 6.95E-06 | 6.37E-07 | -1.68 |
| 228915_at | DACH1 | 8.75E-19 | 7.58E-21 | -2.17 | 1.01E-18 | 6.20E-21 | -2.24 | 1.12E-12 | 1.13E-14 | -2.86 |
| 204677_at | CDH5 | 1.36E-20 | 6.09E-23 | -2.17 | 1.29E-24 | 1.53E-27 | -2.20 | 7.79E-23 | 1.57E-26 | -2.20 |
| 225911_at | NPNT | 1.08E-18 | 9.68E-21 | -2.17 | 1.00E-12 | 2.55E-14 | -1.65 | 1.13E-07 | 5.83E-09 | -1.55 |
| 205326_at | RAMP3 | 5.42E-23 | 8.22E-26 | -2.16 | 9.71E-22 | 2.75E-24 | -2.01 | 2.33E-18 | 3.88E-21 | -2.60 |
| 227148_at | PLEKHH2 | 4.04E-19 | 3.06E-21 | -2.16 | 6.55E-14 | 1.28E-15 | -1.75 | 1.30E-08 | 5.04E-10 | -1.68 |
| 218876_at | TPPP3 | 1.25E-17 | 1.58E-19 | -2.15 | 2.10E-07 | 1.75E-08 | -1.31 | 1.00E-05 | 9.56E-07 | -1.94 |
| 202242_at | TSPAN7 | 3.27E-20 | 1.70E-22 | -2.15 | 3.97E-15 | 5.94E-17 | -1.94 | 3.65E-10 | 8.91E-12 | -2.07 |
| 204894_s_at | AOC3 | 3.00E-21 | 1.02E-23 | -2.15 | 1.26E-16 | 1.33E-18 | -1.96 | 1.26E-14 | 7.37E-17 | -2.56 |
| 225079_at | EMP2 | 3.21E-21 | 1.11E-23 | -2.15 | 7.02E-19 | 4.14E-21 | -1.83 | 8.70E-14 | 6.60E-16 | -1.62 |
| 205609_at | ANGPT1 | 8.87E-22 | 2.24E-24 | -2.15 | 9.32E-12 | 2.96E-13 | -1.74 | 5.13E-06 | 4.48E-07 | -1.55 |
| 220351_at | ACKR4 | 1.89E-21 | 5.74E-24 | -2.14 | 2.42E-15 | 3.45E-17 | -1.77 | 8.09E-12 | 1.15E-13 | -1.97 |
| 220994_s_at | STXBP6 | 1.88E-20 | 8.71E-23 | -2.14 | 1.42E-22 | 2.96E-25 | -2.14 | 9.96E-11 | 2.02E-12 | -2.83 |
| 219059_s_at | LYVE1 | 1.60E-18 | 1.54E-20 | -2.13 | 3.47E-23 | 6.65E-26 | -2.30 | 4.21E-16 | 1.56E-18 | -3.00 |
| 227419_x_at | PLAC9 | 6.36E-21 | 2.48E-23 | -2.13 | 5.35E-23 | 1.05E-25 | -2.07 | 1.17E-11 | 1.80E-13 | -1.87 |
| 223816_at | SLC46A2 | 8.18E-22 | 2.04E-24 | -2.13 | 2.05E-13 | 4.43E-15 | -1.87 | 7.61E-05 | 9.62E-06 | -2.15 |
| 205632_s_at | PIP5K1B | 2.42E-24 | 1.79E-27 | -2.13 | 9.00E-17 | 9.07E-19 | -2.13 | 1.02E-07 | 5.21E-09 | -1.41 |
| 235183_at |  | 1.17E-18 | 1.06E-20 | -2.13 | 1.89E-18 | 1.24E-20 | -2.34 | 4.33E-12 | 5.54E-14 | -1.91 |
| 1560359_at | PELO | 2.42E-22 | 4.91E-25 | -2.12 | 1.15E-13 | 2.35E-15 | -1.66 | 1.50E-13 | 1.23E-15 | -1.70 |
| 220003_at | LRRC36 | 2.26E-25 | 1.05E-28 | -2.12 | 3.20E-05 | 4.55E-06 | -0.96 | 1.07E-05 | 1.03E-06 | -2.09 |
| 206208_at | CA4 | 1.12E-21 | 3.05E-24 | -2.12 | 9.29E-32 | 1.36E-35 | -2.50 | 3.59E-18 | 6.24E-21 | -3.70 |
| 213664_at | SLC1A1 | 3.93E-13 | 1.54E-14 | -2.12 | 4.45E-11 | 1.65E-12 | -1.84 | 2.25E-06 | 1.75E-07 | -1.42 |
| 219478_at | WFDC1 | 3.79E-19 | 2.85E-21 | -2.11 | 9.12E-07 | 8.85E-08 | -0.98 | 1.86E-07 | 1.01E-08 | -1.74 |
| 205382_s_at | CFD | 1.13E-20 | 4.93E-23 | -2.11 | 6.43E-19 | 3.74E-21 | -2.14 | 1.22E-11 | 1.89E-13 | -2.19 |
| 209616_s_at | CES1 | 2.28E-15 | 5.36E-17 | -2.11 | 2.69E-12 | 7.48E-14 | -1.61 | 4.86E-08 | 2.25E-09 | -2.01 |
| 210762_s_at | DLC1 | 4.68E-18 | 5.09E-20 | -2.11 | 5.65E-18 | 4.21E-20 | -1.88 | 7.05E-07 | 4.70E-08 | -1.37 |
| 225720_at | SYNPO2 | 7.79E-21 | 3.15E-23 | -2.11 | 3.41E-11 | 1.23E-12 | -1.55 | 3.22E-08 | 1.41E-09 | -1.81 |
| 209897_s_at | SLIT2 | 8.44E-21 | 3.54E-23 | -2.10 | 4.12E-15 | 6.21E-17 | -1.68 | 2.87E-15 | 1.36E-17 | -1.88 |
| 218730_s_at | OGN | 1.66E-10 | 1.13E-11 | -2.09 | 3.87E-10 | 1.77E-11 | -1.96 | 2.26E-05 | 2.42E-06 | -2.41 |
| 237466_s_at | HHIP | 1.44E-19 | 9.28E-22 | -2.09 | 6.12E-12 | 1.86E-13 | -1.44 | 3.63E-06 | 3.02E-07 | -2.96 |
| 213316_at | KIAA1462 | 1.28E-18 | 1.18E-20 | -2.09 | 2.40E-23 | 4.35E-26 | -2.18 | 2.91E-19 | 3.14E-22 | -1.76 |
| 226380_at | PTPN21 | 3.07E-19 | 2.24E-21 | -2.09 | 1.94E-20 | 7.40E-23 | -1.88 | 1.25E-17 | 2.67E-20 | -1.64 |
| 221667_s_at | HSPB8 | 1.80E-20 | 8.32E-23 | -2.08 | 3.30E-13 | 7.52E-15 | -1.43 | 1.29E-06 | 9.30E-08 | -1.19 |
| 236359_at | SCN4B | 9.30E-18 | 1.11E-19 | -2.08 | 1.89E-19 | 9.59E-22 | -1.98 | 8.02E-09 | 2.91E-10 | -2.77 |
| 236029_at | FAT3 | 1.20E-15 | 2.62E-17 | -2.08 | 1.23E-16 | 1.29E-18 | -1.92 | 4.48E-13 | 4.14E-15 | -2.68 |
| 238332_at | ANKRD29 | 5.67E-16 | 1.12E-17 | -2.08 | 7.18E-22 | 1.96E-24 | -2.52 | 8.04E-08 | 3.96E-09 | -2.01 |
| 219737_s_at | PCDH9 | 2.84E-20 | 1.45E-22 | -2.08 | 8.07E-14 | 1.61E-15 | -1.39 | 1.11E-09 | 3.14E-11 | -1.85 |
| 226145_s_at | FRAS1 | 5.95E-13 | 2.43E-14 | -2.07 | 7.93E-13 | 1.96E-14 | -1.95 | 4.37E-06 | 3.73E-07 | -1.54 |
| 229011_at |  | 1.87E-19 | 1.27E-21 | -2.07 | 2.52E-13 | 5.60E-15 | -1.74 | 2.78E-13 | 2.38E-15 | -1.55 |
| 214091_s_at | GPX3 | 1.85E-16 | 3.27E-18 | -2.06 | 4.02E-13 | 9.40E-15 | -1.72 | 1.30E-09 | 3.77E-11 | -1.68 |
| 201348_at | GPX3 | 3.76E-19 | 2.82E-21 | -2.06 | 9.22E-14 | 1.86E-15 | -1.58 | 1.28E-08 | 4.93E-10 | -1.53 |
| 234996_at | CALCRL | 1.31E-19 | 8.35E-22 | -2.06 | 1.88E-19 | 9.48E-22 | -2.16 | 1.76E-17 | 3.84E-20 | -2.24 |
| 235489_at | RHOJ | 2.37E-19 | 1.66E-21 | -2.06 | 3.97E-19 | 2.13E-21 | -2.14 | 2.04E-13 | 1.70E-15 | -1.83 |
| 239650_at | NCKAP5 | 6.03E-17 | 9.05E-19 | -2.05 | 2.66E-32 | 2.92E-36 | -3.11 | 4.80E-15 | 2.42E-17 | -3.22 |
| 242500_at |  | 1.92E-18 | 1.89E-20 | -2.05 | 5.69E-16 | 7.15E-18 | -1.66 | 1.20E-12 | 1.22E-14 | -2.28 |
| 203865_s_at | ADARB1 | 4.07E-21 | 1.47E-23 | -2.05 | 2.02E-19 | 1.03E-21 | -1.73 | 7.73E-12 | 1.09E-13 | -1.70 |
| 1556325_at | FILIP1 | 2.08E-18 | 2.08E-20 | -2.05 | 3.36E-20 | 1.37E-22 | -2.44 | 4.02E-15 | 1.99E-17 | -1.90 |
| 222520_s_at | IFT57 | 4.20E-20 | 2.34E-22 | -2.05 | 2.86E-08 | 1.97E-09 | -1.22 | 2.14E-05 | 2.27E-06 | -1.20 |
| 235306_at | GIMAP8 | 4.80E-19 | 3.76E-21 | -2.05 | 4.38E-22 | 1.11E-24 | -2.20 | 1.12E-13 | 8.78E-16 | -1.70 |
| 202524_s_at | SPOCK2 | 1.77E-22 | 3.29E-25 | -2.04 | 5.57E-34 | 3.06E-38 | -2.47 | 3.17E-17 | 7.71E-20 | -1.79 |
| 205206_at | KAL1 | 1.75E-16 | 3.08E-18 | -2.04 | 6.93E-20 | 3.09E-22 | -2.42 | 7.80E-13 | 7.60E-15 | -1.63 |
| 203372_s_at | SOCS2 | 3.04E-11 | 1.78E-12 | -2.04 | 1.88E-10 | 8.06E-12 | -2.33 | 1.12E-06 | 7.96E-08 | -1.45 |
| 219869_s_at | SLC39A8 | 1.41E-16 | 2.40E-18 | -2.04 | 4.80E-08 | 3.46E-09 | -1.58 | 2.69E-07 | 1.55E-08 | -1.26 |
| 207069_s_at | SMAD6 | 1.21E-14 | 3.35E-16 | -2.04 | 3.67E-14 | 6.78E-16 | -1.49 | 2.92E-11 | 5.06E-13 | -1.66 |
| 225817_at | CGNL1 | 2.46E-23 | 3.37E-26 | -2.04 | 3.00E-14 | 5.43E-16 | -1.40 | 2.83E-06 | 2.29E-07 | -1.05 |
| 223836_at | FGFBP2 | 7.14E-16 | 1.46E-17 | -2.04 | 6.56E-17 | 6.47E-19 | -2.52 | 8.20E-12 | 1.17E-13 | -2.25 |
| 209267_s_at | SLC39A8 | 5.40E-22 | 1.28E-24 | -2.04 | 1.74E-14 | 3.01E-16 | -1.46 | 3.13E-07 | 1.86E-08 | -0.99 |
| 209170_s_at | GPM6B | 6.41E-19 | 5.22E-21 | -2.04 | 1.33E-17 | 1.11E-19 | -2.20 | 2.17E-12 | 2.42E-14 | -1.90 |
| 239262_at |  | 8.26E-18 | 9.67E-20 | -2.03 | 2.89E-19 | 1.51E-21 | -2.11 | 7.06E-12 | 9.75E-14 | -2.17 |
| 242868_at |  | 2.90E-17 | 4.04E-19 | -2.02 | 5.47E-06 | 6.46E-07 | -1.59 | 2.30E-08 | 9.61E-10 | -1.58 |
| 226950_at | ACVRL1 | 4.11E-22 | 9.26E-25 | -2.02 | 2.03E-26 | 1.52E-29 | -1.99 | 1.85E-18 | 2.91E-21 | -1.96 |
| 229529_at | TCF21 | 5.69E-18 | 6.38E-20 | -2.02 | 1.02E-20 | 3.61E-23 | -1.65 | 5.05E-07 | 3.22E-08 | -2.40 |
| 222738_at | WWC2 | 2.76E-22 | 5.85E-25 | -2.02 | 3.75E-25 | 3.57E-28 | -1.91 | 2.33E-18 | 3.90E-21 | -1.57 |
| 1556989_at |  | 1.52E-13 | 5.40E-15 | -2.02 | 6.62E-06 | 7.98E-07 | -1.37 | 8.33E-10 | 2.28E-11 | -2.25 |
| 212327_at | LIMCH1 | 3.87E-19 | 2.92E-21 | -2.02 | 3.45E-14 | 6.33E-16 | -1.66 | 4.37E-08 | 1.99E-09 | -1.14 |
| 213541_s_at | ERG | 2.42E-20 | 1.18E-22 | -2.02 | 1.39E-16 | 1.48E-18 | -1.80 | 2.55E-16 | 8.62E-19 | -1.84 |
| 218736_s_at | PALMD | 2.42E-20 | 1.18E-22 | -2.01 | 3.38E-14 | 6.19E-16 | -1.54 | 1.32E-13 | 1.05E-15 | -1.71 |
| 222043_at | CLU | 5.82E-15 | 1.50E-16 | -2.01 | 4.08E-12 | 1.19E-13 | -1.63 | 8.36E-05 | 1.07E-05 | -1.44 |
| 210002_at | GATA6 | 7.07E-17 | 1.08E-18 | -2.00 | 6.74E-09 | 4.07E-10 | -1.49 | 1.30E-05 | 1.29E-06 | -1.27 |
| 236262_at | MMRN2 | 2.45E-22 | 5.11E-25 | -2.00 | 1.68E-20 | 6.29E-23 | -1.95 | 4.65E-20 | 3.75E-23 | -2.03 |
| 243509_at |  | 4.10E-20 | 2.25E-22 | -1.99 | 4.31E-07 | 3.86E-08 | -1.46 | 3.23E-15 | 1.55E-17 | -2.01 |
| 241782_at | NEBL | 3.25E-20 | 1.69E-22 | -1.99 | 1.82E-23 | 3.24E-26 | -1.96 | 3.37E-14 | 2.24E-16 | -2.25 |
| 203821_at | HBEGF | 8.99E-17 | 1.42E-18 | -1.99 | 2.18E-16 | 2.44E-18 | -2.02 | 1.03E-08 | 3.88E-10 | -1.37 |
| 227006_at | PPP1R14A | 1.05E-18 | 9.31E-21 | -1.99 | 4.29E-16 | 5.19E-18 | -1.70 | 1.38E-09 | 4.07E-11 | -1.75 |
| 213895_at | EMP1 | 4.19E-13 | 1.65E-14 | -1.99 | 3.37E-11 | 1.21E-12 | -1.94 | 1.13E-10 | 2.34E-12 | -1.50 |
| 205608_s_at | ANGPT1 | 5.59E-18 | 6.25E-20 | -1.99 | 5.11E-14 | 9.78E-16 | -1.63 | 9.71E-07 | 6.76E-08 | -1.46 |
| 241672_at | SERTM1 | 7.80E-15 | 2.06E-16 | -1.98 | 8.13E-28 | 3.87E-31 | -2.72 | 1.12E-21 | 3.91E-25 | -3.96 |
| 219064_at | ITIH5 | 5.32E-22 | 1.26E-24 | -1.98 | 1.99E-16 | 2.20E-18 | -1.40 | 1.48E-07 | 7.88E-09 | -1.82 |
| 205863_at | S100A12 | 5.21E-09 | 4.86E-10 | -1.98 | 3.33E-11 | 1.20E-12 | -2.65 | 1.07E-09 | 3.03E-11 | -1.78 |
| 226303_at | PGM5 | 1.79E-16 | 3.17E-18 | -1.97 | 2.07E-16 | 2.30E-18 | -1.80 | 2.47E-09 | 7.82E-11 | -1.79 |
| 218723_s_at | RGCC | 1.25E-21 | 3.50E-24 | -1.97 | 3.20E-22 | 7.72E-25 | -2.05 | 7.30E-10 | 1.96E-11 | -1.52 |
| 202068_s_at | LDLR | 2.21E-19 | 1.53E-21 | -1.97 | 3.25E-13 | 7.39E-15 | -1.55 | 7.67E-06 | 7.11E-07 | -1.05 |
| 235570_at | RBMS3 | 2.82E-21 | 9.22E-24 | -1.97 | 1.05E-13 | 2.12E-15 | -1.31 | 5.71E-08 | 2.70E-09 | -1.18 |
| 235228_at | CCDC85A | 9.03E-21 | 3.80E-23 | -1.96 | 1.18E-26 | 7.95E-30 | -2.58 | 1.62E-15 | 7.18E-18 | -2.58 |
| 224358_s_at | MS4A7 | 8.67E-18 | 1.03E-19 | -1.96 | 3.05E-07 | 2.64E-08 | -1.23 | 6.31E-08 | 3.01E-09 | -1.31 |
| 218820_at | C14orf132 | 7.01E-19 | 5.86E-21 | -1.95 | 2.47E-14 | 4.41E-16 | -1.61 | 2.40E-10 | 5.47E-12 | -1.70 |
| 227088_at | PDE5A | 2.66E-18 | 2.74E-20 | -1.94 | 8.19E-14 | 1.64E-15 | -1.69 | 1.36E-10 | 2.87E-12 | -1.74 |
| 226490_at | NHSL1 | 4.31E-20 | 2.42E-22 | -1.94 | 2.82E-13 | 6.33E-15 | -1.44 | 1.14E-06 | 8.15E-08 | -1.22 |
| 229070_at | ADTRP | 5.69E-14 | 1.83E-15 | -1.94 | 1.50E-07 | 1.21E-08 | -1.24 | 2.70E-05 | 2.95E-06 | -1.20 |
| 232267_at | GPR133 | 1.81E-15 | 4.15E-17 | -1.93 | 2.40E-11 | 8.36E-13 | -1.55 | 6.49E-07 | 4.26E-08 | -1.61 |
| 220037_s_at | LYVE1 | 2.50E-14 | 7.47E-16 | -1.93 | 3.39E-17 | 3.06E-19 | -2.53 | 3.77E-14 | 2.55E-16 | -3.16 |
| 203373_at | SOCS2 | 1.51E-16 | 2.60E-18 | -1.93 | 2.38E-16 | 2.69E-18 | -2.01 | 3.10E-09 | 1.02E-10 | -1.54 |
| 209167_at | GPM6B | 1.59E-18 | 1.52E-20 | -1.93 | 4.24E-16 | 5.10E-18 | -2.11 | 1.04E-10 | 2.13E-12 | -1.82 |
| 205846_at | PTPRB | 1.00E-17 | 1.22E-19 | -1.93 | 6.62E-20 | 2.93E-22 | -1.92 | 1.76E-18 | 2.74E-21 | -1.95 |
| 208609_s_at | TNXB | 4.54E-24 | 4.06E-27 | -1.93 | 8.45E-17 | 8.47E-19 | -1.47 | 1.79E-14 | 1.10E-16 | -2.28 |
| 219093_at | PID1 | 5.59E-20 | 3.27E-22 | -1.93 | 2.30E-11 | 7.98E-13 | -1.29 | 2.08E-06 | 1.61E-07 | -1.13 |
| 230132_at | PCAT19 | 1.47E-23 | 1.73E-26 | -1.92 | 2.71E-28 | 1.04E-31 | -1.99 | 2.52E-14 | 1.63E-16 | -1.74 |
| 226974_at | NEDD4L | 1.16E-14 | 3.18E-16 | -1.92 | 3.16E-16 | 3.65E-18 | -1.97 | 5.99E-10 | 1.56E-11 | -1.75 |
| 236154_at | QKI | 2.17E-25 | 9.32E-29 | -1.92 | 2.93E-24 | 4.09E-27 | -1.61 | 2.82E-21 | 1.34E-24 | -1.84 |
| 220266_s_at | KLF4 | 1.21E-14 | 3.34E-16 | -1.91 | 8.12E-15 | 1.30E-16 | -2.32 | 3.87E-10 | 9.56E-12 | -2.28 |
| 242809_at | IL1RL1 | 4.04E-15 | 9.99E-17 | -1.91 | 1.84E-11 | 6.23E-13 | -1.72 | 2.71E-08 | 1.16E-09 | -1.56 |
| 204072_s_at | FRY | 9.67E-18 | 1.16E-19 | -1.91 | 1.49E-13 | 3.10E-15 | -1.56 | 3.78E-12 | 4.73E-14 | -1.34 |
| 1559277_at | FLJ35700 | 4.70E-08 | 5.29E-09 | -1.91 | 3.54E-10 | 1.61E-11 | -1.92 | 2.10E-08 | 8.64E-10 | -2.36 |
| 238919_at |  | 1.32E-16 | 2.23E-18 | -1.91 | 6.54E-10 | 3.16E-11 | -1.15 | 9.12E-09 | 3.38E-10 | -1.97 |
| 206658_at | UPK3B | 2.99E-11 | 1.75E-12 | -1.90 | 4.10E-19 | 2.22E-21 | -2.78 | 4.26E-08 | 1.94E-09 | -1.97 |
| 233947_s_at | TBX5-AS1 | 3.19E-18 | 3.36E-20 | -1.90 | 2.07E-12 | 5.63E-14 | -1.32 | 1.60E-08 | 6.35E-10 | -1.64 |
| 226397_s_at |  | 1.50E-26 | 3.17E-30 | -1.90 | 4.77E-21 | 1.54E-23 | -1.34 | 1.80E-08 | 7.29E-10 | -1.04 |
| 221841_s_at | KLF4 | 1.17E-18 | 1.07E-20 | -1.89 | 9.87E-17 | 1.01E-18 | -2.29 | 3.39E-08 | 1.49E-09 | -1.72 |
| 1553645_at | CCDC141 | 3.44E-15 | 8.39E-17 | -1.89 | 5.72E-18 | 4.27E-20 | -1.56 | 3.13E-10 | 7.43E-12 | -2.14 |
| 1562275_at |  | 1.24E-09 | 1.01E-10 | -1.89 | 3.28E-06 | 3.63E-07 | -1.75 | 3.27E-11 | 5.73E-13 | -2.40 |
| 209959_at | NR4A3 | 1.11E-11 | 5.96E-13 | -1.89 | 4.09E-10 | 1.89E-11 | -1.82 | 6.00E-06 | 5.37E-07 | -2.21 |
| 201843_s_at | EFEMP1 | 1.09E-16 | 1.76E-18 | -1.89 | 1.38E-14 | 2.35E-16 | -1.72 | 1.53E-06 | 1.13E-07 | -1.25 |
| 238909_at | S100A10 | 2.00E-12 | 9.08E-14 | -1.88 | 1.53E-10 | 6.45E-12 | -1.58 | 7.52E-06 | 6.96E-07 | -1.25 |
| 210619_s_at | HYAL1 | 2.57E-14 | 7.70E-16 | -1.88 | 3.26E-14 | 5.93E-16 | -1.64 | 2.77E-09 | 8.92E-11 | -2.39 |
| 229824_at | SHC3 | 1.93E-16 | 3.44E-18 | -1.88 | 4.10E-11 | 1.51E-12 | -1.39 | 8.14E-09 | 2.97E-10 | -1.40 |
| 208335_s_at | ACKR1 | 1.19E-13 | 4.16E-15 | -1.88 | 6.89E-13 | 1.68E-14 | -2.18 | 1.91E-05 | 2.00E-06 | -2.24 |
| 230104_s_at | TPPP | 2.33E-19 | 1.62E-21 | -1.87 | 3.19E-10 | 1.43E-11 | -1.29 | 4.82E-07 | 3.05E-08 | -1.45 |
| 236335_at | GUCY1A2 | 1.84E-14 | 5.31E-16 | -1.87 | 5.28E-12 | 1.59E-13 | -1.67 | 7.62E-12 | 1.07E-13 | -1.82 |
| 211276_at | TCEAL2 | 1.10E-14 | 3.00E-16 | -1.87 | 8.06E-14 | 1.61E-15 | -1.87 | 1.04E-06 | 7.29E-08 | -1.87 |
| 243172_at |  | 3.49E-20 | 1.85E-22 | -1.87 | 6.47E-22 | 1.74E-24 | -1.64 | 2.29E-14 | 1.44E-16 | -2.24 |
| 210067_at | AQP4 | 8.81E-11 | 5.63E-12 | -1.87 | 9.73E-08 | 7.56E-09 | -2.32 | 6.98E-05 | 8.71E-06 | -2.34 |
| 210066_s_at | AQP4 | 3.12E-14 | 9.54E-16 | -1.87 | 3.40E-11 | 1.23E-12 | -2.55 | 2.27E-06 | 1.78E-07 | -2.05 |
| 202112_at | VWF | 3.78E-20 | 2.04E-22 | -1.87 | 3.73E-19 | 1.98E-21 | -1.81 | 1.93E-11 | 3.16E-13 | -1.90 |
| 213240_s_at | KRT4 | 2.88E-15 | 6.91E-17 | -1.86 | 2.10E-10 | 9.11E-12 | -1.46 | 3.26E-06 | 2.68E-07 | -2.15 |
| 219440_at | RAI2 | 5.26E-23 | 7.88E-26 | -1.86 | 9.74E-16 | 1.27E-17 | -1.43 | 3.33E-10 | 8.04E-12 | -1.00 |
| 221747_at | TNS1 | 1.15E-21 | 3.18E-24 | -1.86 | 5.13E-19 | 2.86E-21 | -1.47 | 1.80E-12 | 1.96E-14 | -1.32 |
| 226769_at | FIBIN | 3.80E-12 | 1.84E-13 | -1.85 | 3.78E-16 | 4.48E-18 | -2.04 | 3.44E-13 | 3.03E-15 | -2.00 |
| 203296_s_at | ATP1A2 | 4.99E-18 | 5.51E-20 | -1.85 | 6.27E-10 | 3.02E-11 | -1.22 | 4.77E-06 | 4.12E-07 | -2.04 |
| 227417_at | MARC2 | 1.10E-23 | 1.22E-26 | -1.85 | 3.86E-09 | 2.22E-10 | -1.04 | 1.62E-05 | 1.66E-06 | -0.91 |
| 221204_s_at | CRTAC1 | 1.44E-18 | 1.36E-20 | -1.85 | 9.32E-19 | 5.65E-21 | -1.52 | 7.43E-08 | 3.62E-09 | -2.15 |
| 222162_s_at | ADAMTS1 | 1.63E-12 | 7.30E-14 | -1.84 | 2.44E-14 | 4.36E-16 | -2.04 | 2.74E-11 | 4.71E-13 | -2.21 |
| 204422_s_at | FGF2 | 5.25E-20 | 3.02E-22 | -1.84 | 1.50E-15 | 2.05E-17 | -1.83 | 3.51E-13 | 3.12E-15 | -1.77 |
| 226101_at | PRKCE | 1.32E-21 | 3.75E-24 | -1.84 | 2.62E-20 | 1.04E-22 | -1.45 | 2.75E-17 | 6.60E-20 | -1.18 |
| 204438_at | MRC1 | 1.73E-16 | 3.05E-18 | -1.84 | 6.07E-07 | 5.65E-08 | -1.00 | 5.08E-06 | 4.44E-07 | -1.18 |
| 1557422_at |  | 7.72E-16 | 1.60E-17 | -1.84 | 6.87E-07 | 6.50E-08 | -1.13 | 7.44E-07 | 4.99E-08 | -1.50 |
| 226627_at | SEPT8 | 1.50E-26 | 3.35E-30 | -1.84 | 1.02E-20 | 3.65E-23 | -1.07 | 2.56E-14 | 1.67E-16 | -1.55 |
| 225660_at | SEMA6A | 3.96E-12 | 1.92E-13 | -1.84 | 1.90E-23 | 3.40E-26 | -1.82 | 1.72E-12 | 1.85E-14 | -2.46 |
| 207302_at | SGCG | 1.53E-19 | 1.00E-21 | -1.84 | 1.05E-30 | 2.31E-34 | -2.08 | 1.51E-11 | 2.41E-13 | -3.63 |
| 213715_s_at | KANK3 | 2.08E-20 | 9.91E-23 | -1.83 | 1.68E-30 | 4.31E-34 | -2.01 | 2.91E-16 | 1.02E-18 | -2.20 |
| 229613_at |  | 6.06E-16 | 1.22E-17 | -1.83 | 2.18E-11 | 7.52E-13 | -1.16 | 3.02E-09 | 9.86E-11 | -1.92 |
| 209821_at | IL33 | 4.67E-14 | 1.47E-15 | -1.83 | 1.51E-12 | 3.99E-14 | -2.04 | 2.66E-07 | 1.54E-08 | -1.95 |
| 38037_at | HBEGF | 5.81E-16 | 1.16E-17 | -1.83 | 3.65E-16 | 4.30E-18 | -1.80 | 8.57E-10 | 2.36E-11 | -1.56 |
| 214770_at | MSR1 | 1.57E-15 | 3.54E-17 | -1.83 | 3.69E-07 | 3.25E-08 | -1.11 | 5.75E-08 | 2.72E-09 | -1.21 |
| 206030_at | ASPA | 9.65E-21 | 4.10E-23 | -1.82 | 5.75E-20 | 2.50E-22 | -1.65 | 1.05E-12 | 1.06E-14 | -2.38 |
| 209291_at | ID4 | 5.48E-15 | 1.40E-16 | -1.82 | 1.87E-10 | 8.03E-12 | -1.45 | 5.54E-08 | 2.60E-09 | -1.48 |
| 204446_s_at | ALOX5 | 4.83E-16 | 9.38E-18 | -1.82 | 8.71E-11 | 3.47E-12 | -1.09 | 1.16E-06 | 8.29E-08 | -0.86 |
| 223315_at | NTN4 | 1.89E-19 | 1.29E-21 | -1.82 | 4.70E-08 | 3.38E-09 | -1.18 | 2.73E-06 | 2.19E-07 | -1.03 |
| 225369_at | ESAM | 5.24E-19 | 4.15E-21 | -1.82 | 5.25E-22 | 1.36E-24 | -1.53 | 1.13E-12 | 1.14E-14 | -1.67 |
| 206283_s_at | TAL1 | 1.50E-21 | 4.40E-24 | -1.81 | 4.01E-25 | 4.03E-28 | -2.21 | 3.51E-19 | 3.98E-22 | -2.33 |
| 217628_at | CLIC5 | 7.94E-19 | 6.80E-21 | -1.81 | 5.74E-21 | 1.89E-23 | -2.31 | 9.12E-09 | 3.38E-10 | -1.84 |
| 212328_at | LIMCH1 | 4.34E-17 | 6.26E-19 | -1.81 | 1.12E-12 | 2.88E-14 | -1.55 | 4.64E-09 | 1.59E-10 | -1.31 |
| 219836_at | ZBED2 | 7.65E-12 | 3.97E-13 | -1.81 | 4.55E-20 | 1.92E-22 | -2.34 | 2.78E-10 | 6.46E-12 | -2.09 |
| 231947_at | MYCT1 | 6.81E-22 | 1.64E-24 | -1.81 | 1.70E-22 | 3.67E-25 | -1.87 | 4.07E-16 | 1.51E-18 | -2.37 |
| 204579_at | FGFR4 | 7.76E-18 | 9.04E-20 | -1.80 | 2.82E-21 | 8.81E-24 | -1.75 | 2.18E-11 | 3.64E-13 | -2.60 |
| 1552509_a_at | CD300LG | 2.97E-15 | 7.13E-17 | -1.80 | 1.57E-21 | 4.66E-24 | -2.27 | 5.45E-16 | 2.15E-18 | -3.76 |
| 227929_at |  | 4.52E-20 | 2.54E-22 | -1.80 | 2.79E-25 | 2.55E-28 | -2.23 | 1.04E-17 | 2.13E-20 | -1.86 |
| 227178_at | CELF2 | 2.72E-24 | 2.19E-27 | -1.80 | 1.67E-23 | 2.81E-26 | -1.36 | 8.37E-16 | 3.47E-18 | -1.59 |
| 203812_at | SLIT3 | 6.40E-25 | 3.98E-28 | -1.80 | 8.51E-21 | 3.00E-23 | -1.17 | 3.76E-11 | 6.71E-13 | -1.78 |
| 204955_at | SRPX | 1.68E-13 | 6.03E-15 | -1.80 | 1.56E-13 | 3.27E-15 | -1.59 | 6.49E-12 | 8.82E-14 | -1.74 |
| 223775_at | HHIP | 1.57E-18 | 1.49E-20 | -1.80 | 7.59E-11 | 2.97E-12 | -1.56 | 7.32E-06 | 6.75E-07 | -2.62 |
| 204468_s_at | TIE1 | 9.97E-17 | 1.60E-18 | -1.79 | 3.08E-22 | 7.38E-25 | -1.83 | 5.10E-14 | 3.58E-16 | -1.54 |
| 225575_at | LIFR | 2.28E-14 | 6.73E-16 | -1.79 | 1.57E-14 | 2.70E-16 | -1.62 | 4.18E-09 | 1.42E-10 | -1.66 |
| 205407_at | RECK | 3.45E-20 | 1.82E-22 | -1.79 | 8.46E-15 | 1.36E-16 | -1.54 | 5.09E-14 | 3.57E-16 | -1.50 |
| 204796_at | EML1 | 1.46E-16 | 2.49E-18 | -1.79 | 3.64E-14 | 6.71E-16 | -1.50 | 4.62E-19 | 5.40E-22 | -1.74 |
| 228480_at | VAPA | 2.85E-17 | 3.96E-19 | -1.79 | 2.89E-12 | 8.15E-14 | -1.42 | 5.64E-15 | 2.94E-17 | -1.86 |
| 203435_s_at | MME | 1.17E-16 | 1.94E-18 | -1.79 | 4.30E-28 | 1.89E-31 | -2.19 | 5.03E-10 | 1.29E-11 | -3.20 |
| 209793_at | GRIA1 | 1.26E-17 | 1.58E-19 | -1.79 | 1.23E-25 | 1.08E-28 | -1.99 | 1.20E-11 | 1.84E-13 | -3.26 |
| 225207_at | PDK4 | 2.67E-09 | 2.34E-10 | -1.79 | 9.10E-13 | 2.29E-14 | -2.73 | 2.11E-12 | 2.35E-14 | -2.39 |
| 202759_s_at | PALM2-AKAP2///AKAP2 | 1.23E-20 | 5.41E-23 | -1.79 | 8.04E-23 | 1.59E-25 | -2.08 | 1.70E-21 | 6.82E-25 | -1.73 |
| 214265_at | ITGA8 | 3.10E-16 | 5.78E-18 | -1.79 | 3.27E-12 | 9.32E-14 | -1.45 | 2.60E-08 | 1.10E-09 | -1.62 |
| 223343_at | MS4A7 | 2.50E-17 | 3.44E-19 | -1.79 | 1.05E-08 | 6.61E-10 | -1.06 | 7.25E-10 | 1.95E-11 | -1.22 |
| 205792_at | WISP2 | 2.10E-13 | 7.72E-15 | -1.78 | 1.25E-14 | 2.09E-16 | -1.60 | 2.12E-11 | 3.52E-13 | -1.97 |
| 227448_at | ARGLU1 | 3.65E-18 | 3.87E-20 | -1.78 | 2.04E-11 | 6.98E-13 | -1.20 | 2.42E-12 | 2.78E-14 | -1.33 |
| 204396_s_at | GRK5 | 2.59E-21 | 8.40E-24 | -1.78 | 1.06E-29 | 2.91E-33 | -2.52 | 5.89E-18 | 1.09E-20 | -2.01 |
| 204368_at | SLCO2A1 | 1.54E-14 | 4.35E-16 | -1.78 | 7.50E-12 | 2.34E-13 | -1.39 | 1.03E-11 | 1.53E-13 | -1.54 |
| 222802_at | EDN1 | 5.03E-11 | 3.08E-12 | -1.78 | 6.74E-17 | 6.66E-19 | -1.91 | 2.29E-10 | 5.17E-12 | -1.36 |
| 207294_at | AGTR2 | 2.54E-08 | 2.71E-09 | -1.77 | 5.20E-08 | 3.78E-09 | -1.24 | 7.71E-06 | 7.16E-07 | -2.25 |
| 235849_at | SCARA5 | 5.72E-15 | 1.47E-16 | -1.77 | 9.99E-13 | 2.53E-14 | -1.64 | 2.42E-07 | 1.38E-08 | -2.81 |
| 209169_at | GPM6B | 9.41E-18 | 1.13E-19 | -1.77 | 1.85E-19 | 9.25E-22 | -1.94 | 2.29E-12 | 2.59E-14 | -2.23 |
| 216504_s_at | SLC39A8 | 1.52E-11 | 8.37E-13 | -1.77 | 1.44E-12 | 3.79E-14 | -1.14 | 4.00E-08 | 1.80E-09 | -1.06 |
| 226188_at | LGALSL | 2.44E-20 | 1.20E-22 | -1.77 | 5.06E-12 | 1.51E-13 | -1.02 | 6.46E-07 | 4.23E-08 | -1.07 |
| 221748_s_at | TNS1 | 3.22E-19 | 2.38E-21 | -1.77 | 6.30E-14 | 1.22E-15 | -1.26 | 4.72E-07 | 2.98E-08 | -0.76 |
| 213169_at | SEMA5A | 9.38E-19 | 8.21E-21 | -1.77 | 8.67E-19 | 5.18E-21 | -1.78 | 2.82E-12 | 3.33E-14 | -1.73 |
| 227762_at |  | 6.50E-08 | 7.55E-09 | -1.76 | 5.18E-12 | 1.55E-13 | -2.12 | 2.14E-11 | 3.56E-13 | -2.54 |
| 204041_at | MAOB | 9.65E-19 | 8.53E-21 | -1.76 | 8.82E-12 | 2.78E-13 | -1.06 | 2.98E-06 | 2.42E-07 | -1.45 |
| 219091_s_at | MMRN2 | 1.09E-21 | 2.92E-24 | -1.76 | 1.16E-20 | 4.18E-23 | -1.76 | 1.00E-21 | 3.31E-25 | -1.91 |
| 228737_at | TOX2 | 6.42E-19 | 5.24E-21 | -1.75 | 2.66E-17 | 2.35E-19 | -1.76 | 2.42E-12 | 2.77E-14 | -1.45 |
| 219682_s_at | TBX3 | 5.76E-15 | 1.48E-16 | -1.75 | 7.14E-14 | 1.40E-15 | -1.91 | 3.82E-22 | 9.78E-26 | -1.96 |
| 226462_at | STXBP6 | 1.79E-18 | 1.73E-20 | -1.75 | 1.02E-18 | 6.25E-21 | -2.02 | 1.73E-11 | 2.81E-13 | -2.45 |
| 208195_at | TTN | 1.59E-17 | 2.08E-19 | -1.75 | 4.88E-13 | 1.16E-14 | -1.10 | 1.85E-08 | 7.51E-10 | -1.49 |
| 228875_at | FAM162B | 1.36E-14 | 3.82E-16 | -1.75 | 2.75E-18 | 1.89E-20 | -1.72 | 8.54E-15 | 4.64E-17 | -2.07 |
| 210258_at | RGS13 | 1.66E-14 | 4.73E-16 | -1.74 | 2.25E-07 | 1.89E-08 | -1.37 | 5.05E-06 | 4.41E-07 | -1.38 |
| 206953_s_at | LPHN2 | 8.92E-17 | 1.40E-18 | -1.74 | 4.82E-18 | 3.51E-20 | -2.08 | 2.22E-17 | 5.16E-20 | -1.94 |
| 228731_at | GUCY1A2 | 1.08E-13 | 3.73E-15 | -1.74 | 6.63E-11 | 2.56E-12 | -1.65 | 3.22E-10 | 7.72E-12 | -1.82 |
| 213006_at | CEBPD | 4.16E-13 | 1.64E-14 | -1.73 | 8.25E-12 | 2.59E-13 | -1.92 | 1.46E-08 | 5.73E-10 | -1.78 |
| 219452_at | DPEP2 | 1.47E-23 | 1.75E-26 | -1.73 | 1.63E-16 | 1.75E-18 | -1.28 | 2.34E-08 | 9.80E-10 | -1.15 |
| 219213_at | JAM2 | 1.01E-19 | 6.21E-22 | -1.73 | 2.32E-22 | 5.22E-25 | -1.65 | 9.70E-14 | 7.44E-16 | -1.90 |
| 235591_at | SSTR1 | 2.67E-13 | 1.00E-14 | -1.73 | 1.12E-12 | 2.88E-14 | -1.76 | 6.71E-10 | 1.78E-11 | -2.21 |
| 210383_at | SCN1A | 2.08E-18 | 2.06E-20 | -1.73 | 9.52E-08 | 7.38E-09 | -0.93 | 8.54E-06 | 8.01E-07 | -2.48 |
| 215028_at | SEMA6A | 1.74E-09 | 1.46E-10 | -1.73 | 4.58E-12 | 1.35E-13 | -1.30 | 6.33E-13 | 6.06E-15 | -2.70 |
| 205384_at | FXYD1 | 2.69E-19 | 1.95E-21 | -1.73 | 1.68E-17 | 1.42E-19 | -1.63 | 7.04E-07 | 4.69E-08 | -1.85 |
| 204787_at | VSIG4 | 1.14E-16 | 1.88E-18 | -1.73 | 6.52E-09 | 3.92E-10 | -1.17 | 7.28E-08 | 3.54E-09 | -1.47 |
| 238447_at | RBMS3 | 9.80E-17 | 1.57E-18 | -1.72 | 5.35E-07 | 4.91E-08 | -1.11 | 2.80E-07 | 1.63E-08 | -1.14 |
| 228770_at | GPR146 | 2.25E-21 | 7.03E-24 | -1.72 | 6.28E-21 | 2.09E-23 | -1.64 | 1.20E-13 | 9.43E-16 | -1.85 |
| 202878_s_at | CD93 | 1.05E-17 | 1.29E-19 | -1.72 | 4.82E-18 | 3.52E-20 | -1.72 | 4.66E-17 | 1.20E-19 | -1.48 |
| 203835_at | LRRC32 | 8.89E-16 | 1.87E-17 | -1.72 | 5.38E-17 | 5.16E-19 | -1.53 | 7.96E-12 | 1.13E-13 | -1.45 |
| 235301_at | KIAA1324L | 4.79E-24 | 4.42E-27 | -1.72 | 1.37E-09 | 7.14E-11 | -1.27 | 1.79E-06 | 1.36E-07 | -1.41 |
| 231773_at | ANGPTL1 | 2.51E-19 | 1.79E-21 | -1.72 | 8.91E-20 | 4.04E-22 | -1.87 | 5.73E-14 | 4.11E-16 | -2.70 |
| 1558397_at | PECAM1 | 2.09E-17 | 2.81E-19 | -1.72 | 4.46E-21 | 1.43E-23 | -1.88 | 2.51E-17 | 5.93E-20 | -2.02 |
| 228325_at | SPIDR | 2.31E-13 | 8.58E-15 | -1.72 | 2.82E-13 | 6.33E-15 | -1.78 | 5.99E-08 | 2.84E-09 | -1.36 |
| 227780_s_at | ECSCR | 1.32E-21 | 3.71E-24 | -1.72 | 7.55E-26 | 5.94E-29 | -1.81 | 4.39E-20 | 3.29E-23 | -1.91 |
| 228665_at | CYYR1 | 1.54E-18 | 1.47E-20 | -1.71 | 1.80E-23 | 3.15E-26 | -1.76 | 9.85E-16 | 4.13E-18 | -1.90 |
| 232060_at | ROR1 | 8.47E-16 | 1.77E-17 | -1.71 | 8.58E-09 | 5.29E-10 | -1.06 | 4.80E-05 | 5.69E-06 | -0.88 |
| 228548_at | RAP1A | 7.77E-22 | 1.90E-24 | -1.71 | 3.59E-18 | 2.53E-20 | -1.30 | 3.52E-13 | 3.14E-15 | -1.31 |
| 203562_at | FEZ1 | 2.89E-18 | 3.03E-20 | -1.71 | 4.46E-15 | 6.81E-17 | -1.61 | 5.06E-11 | 9.34E-13 | -1.59 |
| 214696_at | MIR22HG | 7.11E-18 | 8.21E-20 | -1.71 | 1.33E-13 | 2.75E-15 | -1.43 | 7.59E-12 | 1.06E-13 | -1.42 |
| 203766_s_at | LMOD1 | 1.05E-17 | 1.29E-19 | -1.71 | 2.76E-08 | 1.90E-09 | -0.98 | 3.30E-06 | 2.71E-07 | -1.41 |
| 213103_at | STARD13 | 2.59E-24 | 2.04E-27 | -1.71 | 1.08E-22 | 2.19E-25 | -1.35 | 4.65E-20 | 3.69E-23 | -1.47 |
| 201496_x_at | MYH11 | 6.21E-16 | 1.25E-17 | -1.70 | 1.05E-06 | 1.04E-07 | -1.21 | 1.11E-05 | 1.07E-06 | -1.46 |
| 217177_s_at | PTPRB | 7.79E-21 | 3.14E-23 | -1.70 | 1.73E-27 | 9.15E-31 | -1.93 | 2.97E-20 | 2.06E-23 | -2.38 |
| 203074_at | ANXA8L1///ANXA8 | 1.62E-09 | 1.36E-10 | -1.70 | 1.74E-19 | 8.57E-22 | -1.85 | 3.99E-12 | 5.09E-14 | -2.60 |
| 219993_at | SOX17 | 6.26E-14 | 2.04E-15 | -1.70 | 9.31E-19 | 5.62E-21 | -1.91 | 2.86E-21 | 1.41E-24 | -1.82 |
| 226056_at | ARHGAP31 | 5.23E-24 | 5.07E-27 | -1.70 | 2.98E-20 | 1.19E-22 | -1.12 | 5.38E-11 | 1.00E-12 | -1.02 |
| 229172_at | HSPA12B | 2.47E-20 | 1.22E-22 | -1.70 | 2.80E-23 | 5.27E-26 | -1.53 | 5.59E-23 | 9.20E-27 | -2.08 |
| 32625_at | NPR1 | 4.84E-22 | 1.13E-24 | -1.70 | 4.41E-15 | 6.70E-17 | -1.13 | 7.09E-11 | 1.37E-12 | -1.83 |
| 205237_at | FCN1 | 1.09E-14 | 2.96E-16 | -1.70 | 4.27E-13 | 1.00E-14 | -1.83 | 1.18E-07 | 6.13E-09 | -1.40 |
| 208438_s_at | FGR | 2.17E-21 | 6.69E-24 | -1.69 | 1.54E-13 | 3.22E-15 | -1.42 | 2.95E-10 | 6.93E-12 | -1.25 |
| 237252_at | THBD | 1.54E-16 | 2.66E-18 | -1.69 | 7.79E-16 | 1.00E-17 | -1.80 | 1.58E-18 | 2.37E-21 | -1.99 |
| 1569608_x_at | ANKRD20A9P | 1.01E-16 | 1.62E-18 | -1.69 | 7.23E-09 | 4.39E-10 | -1.03 | 2.91E-08 | 1.26E-09 | -2.04 |
| 203088_at | FBLN5 | 5.83E-14 | 1.89E-15 | -1.69 | 4.65E-12 | 1.38E-13 | -1.35 | 8.91E-07 | 6.13E-08 | -1.38 |
| 228339_at | ECSCR | 3.93E-21 | 1.40E-23 | -1.69 | 1.17E-22 | 2.40E-25 | -1.80 | 5.10E-20 | 4.20E-23 | -2.02 |
| 213247_at | SVEP1 | 8.00E-22 | 1.97E-24 | -1.69 | 9.10E-17 | 9.22E-19 | -1.50 | 1.32E-08 | 5.10E-10 | -1.91 |
| 228174_at | SCAI | 5.33E-17 | 7.90E-19 | -1.68 | 1.85E-15 | 2.58E-17 | -1.45 | 1.48E-09 | 4.36E-11 | -1.05 |
| 225571_at | LIFR | 6.39E-10 | 4.93E-11 | -1.68 | 3.53E-12 | 1.02E-13 | -1.82 | 9.55E-10 | 2.65E-11 | -1.78 |
| 211343_s_at | COL13A1 | 3.37E-15 | 8.21E-17 | -1.68 | 1.64E-13 | 3.46E-15 | -1.32 | 3.32E-10 | 8.00E-12 | -1.40 |
| 222453_at | CYBRD1 | 1.31E-18 | 1.22E-20 | -1.68 | 9.08E-13 | 2.28E-14 | -1.26 | 3.60E-05 | 4.11E-06 | -0.73 |
| 231991_at | CCM2L | 3.12E-21 | 1.07E-23 | -1.68 | 1.75E-28 | 6.07E-32 | -1.78 | 1.88E-20 | 1.13E-23 | -2.21 |
| 236089_at |  | 3.80E-15 | 9.36E-17 | -1.68 | 1.83E-15 | 2.54E-17 | -1.69 | 4.19E-13 | 3.84E-15 | -1.78 |
| 1554547_at | FAM13C | 5.29E-17 | 7.82E-19 | -1.68 | 3.56E-13 | 8.18E-15 | -1.74 | 3.30E-11 | 5.81E-13 | -1.89 |
| 211998_at | H3F3B | 9.13E-18 | 1.09E-19 | -1.68 | 2.73E-18 | 1.86E-20 | -1.25 | 3.73E-12 | 4.64E-14 | -1.42 |
| 229518_at | FAM46B | 1.28E-20 | 5.64E-23 | -1.67 | 8.31E-22 | 2.28E-24 | -1.73 | 6.18E-09 | 2.19E-10 | -1.46 |
| 235427_at | CFLAR | 1.94E-20 | 9.13E-23 | -1.67 | 2.34E-11 | 8.14E-13 | -1.32 | 1.49E-09 | 4.41E-11 | -0.90 |
| 1564796_at | EMP1 | 9.02E-11 | 5.78E-12 | -1.67 | 1.63E-09 | 8.61E-11 | -1.69 | 4.94E-06 | 4.29E-07 | -1.81 |
| 1555216_a_at | LOC645722 | 1.22E-16 | 2.04E-18 | -1.67 | 2.49E-19 | 1.29E-21 | -1.83 | 2.74E-11 | 4.70E-13 | -1.52 |
| 226225_at | MCC | 1.77E-14 | 5.11E-16 | -1.67 | 1.27E-08 | 8.14E-10 | -1.18 | 1.17E-11 | 1.81E-13 | -1.14 |
| 236712_at |  | 1.28E-12 | 5.60E-14 | -1.67 | 1.58E-12 | 4.18E-14 | -1.34 | 1.00E-14 | 5.60E-17 | -1.92 |
| 1552398_a_at | CLEC12A | 1.88E-13 | 6.83E-15 | -1.67 | 1.19E-10 | 4.92E-12 | -1.35 | 8.19E-11 | 1.61E-12 | -1.48 |
| 238206_at | RXFP1 | 7.50E-20 | 4.54E-22 | -1.67 | 1.70E-24 | 2.11E-27 | -2.01 | 2.10E-12 | 2.31E-14 | -2.90 |
| 1561135_at |  | 9.53E-18 | 1.14E-19 | -1.67 | 5.32E-07 | 4.88E-08 | -0.89 | 1.80E-14 | 1.11E-16 | -1.87 |
| 204642_at | S1PR1 | 1.86E-19 | 1.25E-21 | -1.67 | 2.57E-23 | 4.75E-26 | -1.99 | 1.43E-19 | 1.46E-22 | -2.21 |
| 239942_at |  | 2.01E-12 | 9.12E-14 | -1.66 | 8.96E-25 | 9.99E-28 | -2.24 | 1.09E-19 | 1.03E-22 | -2.74 |
| 230943_at | SOX17 | 1.53E-12 | 6.76E-14 | -1.66 | 2.01E-16 | 2.22E-18 | -1.77 | 2.77E-13 | 2.36E-15 | -1.85 |
| 226856_at | MUSTN1///TMEM110 | 2.78E-18 | 2.90E-20 | -1.66 | 4.22E-14 | 7.93E-16 | -1.22 | 2.98E-08 | 1.29E-09 | -1.34 |
| 227529_s_at | AKAP12 | 7.81E-11 | 4.95E-12 | -1.66 | 1.18E-13 | 2.42E-15 | -2.34 | 1.78E-10 | 3.90E-12 | -1.87 |
| 40560_at | TBX2 | 2.77E-15 | 6.62E-17 | -1.66 | 9.46E-13 | 2.39E-14 | -1.28 | 5.77E-16 | 2.33E-18 | -1.60 |
| 235561_at | TXNL1 | 8.40E-19 | 7.25E-21 | -1.66 | 4.83E-15 | 7.47E-17 | -1.43 | 8.34E-11 | 1.65E-12 | -1.72 |
| 203324_s_at | CAV2 | 1.50E-14 | 4.23E-16 | -1.66 | 1.77E-12 | 4.72E-14 | -1.82 | 1.77E-09 | 5.36E-11 | -1.38 |
| 222321_at | AGTR2 | 8.92E-09 | 8.68E-10 | -1.65 | 1.63E-08 | 1.06E-09 | -0.93 | 2.43E-07 | 1.39E-08 | -1.98 |
| 236583_at | GIMAP1 | 4.43E-19 | 3.42E-21 | -1.65 | 4.25E-19 | 2.31E-21 | -1.34 | 8.50E-13 | 8.31E-15 | -1.73 |
| 208510_s_at | PPARG | 2.33E-15 | 5.48E-17 | -1.65 | 3.86E-10 | 1.77E-11 | -1.07 | 2.23E-10 | 5.01E-12 | -1.94 |
| 230645_at | FRMD3 | 1.58E-16 | 2.74E-18 | -1.65 | 4.09E-22 | 1.00E-24 | -2.29 | 3.45E-15 | 1.68E-17 | -1.96 |
| 206898_at | CDH19 | 2.44E-20 | 1.20E-22 | -1.65 | 1.43E-20 | 5.27E-23 | -1.94 | 1.39E-14 | 8.25E-17 | -2.81 |
| 205083_at | AOX1 | 4.44E-15 | 1.12E-16 | -1.65 | 1.12E-13 | 2.29E-15 | -1.76 | 8.24E-12 | 1.18E-13 | -1.97 |
| 235581_at |  | 6.79E-20 | 4.07E-22 | -1.65 | 8.31E-10 | 4.11E-11 | -1.00 | 1.19E-07 | 6.21E-09 | -0.92 |
| 1553243_at | ITIH5 | 2.42E-24 | 1.81E-27 | -1.64 | 3.04E-21 | 9.68E-24 | -1.24 | 1.92E-11 | 3.15E-13 | -1.96 |
| 217437_s_at | TACC1 | 4.58E-22 | 1.06E-24 | -1.64 | 9.42E-18 | 7.48E-20 | -1.40 | 1.96E-17 | 4.41E-20 | -1.69 |
| 219719_at | HIGD1B | 2.14E-20 | 1.03E-22 | -1.64 | 3.56E-26 | 2.73E-29 | -1.90 | 1.88E-09 | 5.74E-11 | -2.05 |
| 205003_at | DOCK4 | 3.88E-21 | 1.38E-23 | -1.63 | 2.22E-21 | 6.78E-24 | -1.45 | 3.65E-14 | 2.46E-16 | -1.15 |
| 219529_at | CLIC3 | 4.68E-13 | 1.88E-14 | -1.63 | 6.37E-16 | 8.08E-18 | -1.88 | 1.49E-06 | 1.10E-07 | -1.13 |
| 221272_s_at | C1orf21 | 7.43E-23 | 1.15E-25 | -1.63 | 3.41E-14 | 6.25E-16 | -1.02 | 1.49E-10 | 3.19E-12 | -1.54 |
| 212730_at | SYNM | 1.02E-19 | 6.28E-22 | -1.63 | 1.45E-18 | 9.26E-21 | -1.74 | 3.85E-16 | 1.42E-18 | -1.99 |
| 241789_at | RBMS3 | 4.89E-17 | 7.15E-19 | -1.63 | 1.32E-06 | 1.33E-07 | -1.13 | 3.30E-06 | 2.72E-07 | -1.20 |
| 202674_s_at | LMO7 | 1.53E-17 | 1.98E-19 | -1.63 | 4.60E-17 | 4.36E-19 | -1.81 | 9.81E-08 | 4.97E-09 | -1.27 |
| 204364_s_at | REEP1 | 2.25E-12 | 1.03E-13 | -1.63 | 1.29E-11 | 4.25E-13 | -1.35 | 6.07E-10 | 1.59E-11 | -1.80 |
| 201150_s_at | TIMP3 | 1.22E-14 | 3.37E-16 | -1.63 | 2.24E-12 | 6.13E-14 | -1.23 | 5.11E-08 | 2.37E-09 | -0.99 |
| 220027_s_at | RASIP1 | 2.46E-18 | 2.52E-20 | -1.63 | 8.16E-28 | 4.03E-31 | -1.95 | 5.82E-21 | 3.41E-24 | -2.18 |
| 207008_at | CXCR2 | 2.49E-13 | 9.31E-15 | -1.62 | 8.44E-14 | 1.69E-15 | -2.40 | 6.41E-12 | 8.70E-14 | -1.76 |
| 228728_at | CPED1 | 2.57E-20 | 1.29E-22 | -1.62 | 1.76E-13 | 3.74E-15 | -1.22 | 3.90E-07 | 2.38E-08 | -1.00 |
| 226985_at | FGD5 | 1.13E-19 | 7.16E-22 | -1.62 | 2.11E-22 | 4.70E-25 | -1.50 | 8.99E-20 | 7.89E-23 | -1.68 |
| 226103_at | NEXN | 2.69E-16 | 4.95E-18 | -1.62 | 5.14E-08 | 3.74E-09 | -1.39 | 2.24E-09 | 6.99E-11 | -1.36 |
| 204249_s_at | LMO2 | 2.73E-19 | 1.98E-21 | -1.62 | 1.35E-17 | 1.13E-19 | -1.33 | 5.10E-15 | 2.60E-17 | -1.21 |
| 205392_s_at | CCL15-CCL14///CCL14 | 1.94E-10 | 1.35E-11 | -1.62 | 4.11E-12 | 1.20E-13 | -1.94 | 2.09E-06 | 1.61E-07 | -1.80 |
| 239832_at |  | 8.19E-20 | 5.00E-22 | -1.61 | 5.73E-06 | 6.80E-07 | -0.71 | 1.12E-05 | 1.08E-06 | -1.36 |
| 209369_at | ANXA3 | 1.52E-11 | 8.35E-13 | -1.61 | 5.73E-11 | 2.18E-12 | -1.78 | 1.78E-07 | 9.66E-09 | -1.40 |
| 205779_at | RAMP2 | 8.91E-16 | 1.88E-17 | -1.61 | 3.59E-18 | 2.53E-20 | -1.71 | 5.31E-13 | 5.01E-15 | -1.28 |
| 218995_s_at | EDN1 | 1.40E-11 | 7.67E-13 | -1.61 | 1.17E-14 | 1.93E-16 | -1.62 | 1.21E-12 | 1.25E-14 | -1.59 |
| 236982_at |  | 1.94E-07 | 2.47E-08 | -1.60 | 8.44E-08 | 6.46E-09 | -1.42 | 5.55E-08 | 2.61E-09 | -0.90 |
| 200878_at | EPAS1 | 1.24E-18 | 1.13E-20 | -1.60 | 6.88E-25 | 7.56E-28 | -1.63 | 6.54E-12 | 8.91E-14 | -0.90 |
| 230711_at |  | 1.67E-15 | 3.79E-17 | -1.60 | 1.21E-09 | 6.20E-11 | -1.48 | 1.35E-11 | 2.11E-13 | -1.88 |
| 239108_at | FAR2 | 2.87E-15 | 6.87E-17 | -1.60 | 1.62E-08 | 1.06E-09 | -1.11 | 1.31E-09 | 3.80E-11 | -1.36 |
| 230959_at |  | 5.67E-15 | 1.45E-16 | -1.60 | 3.79E-08 | 2.67E-09 | -0.81 | 2.72E-12 | 3.17E-14 | -2.29 |
| 223344_s_at | MS4A7 | 5.41E-15 | 1.38E-16 | -1.60 | 3.08E-09 | 1.73E-10 | -1.12 | 1.40E-07 | 7.38E-09 | -1.12 |
| 210815_s_at | CALCRL | 1.71E-09 | 1.43E-10 | -1.60 | 1.22E-08 | 7.73E-10 | -2.04 | 5.07E-14 | 3.54E-16 | -1.68 |
| 237737_at | LOC100289026 | 6.08E-15 | 1.57E-16 | -1.59 | 3.66E-06 | 4.12E-07 | -0.73 | 3.76E-05 | 4.31E-06 | -1.56 |
| 231067_s_at | AKAP12 | 1.76E-08 | 1.82E-09 | -1.59 | 1.09E-14 | 1.80E-16 | -2.04 | 9.27E-11 | 1.87E-12 | -2.12 |
| 213417_at | TBX2 | 5.80E-14 | 1.88E-15 | -1.59 | 7.08E-14 | 1.39E-15 | -1.24 | 7.03E-10 | 1.89E-11 | -1.59 |
| 229367_s_at | GIMAP6 | 6.88E-17 | 1.05E-18 | -1.59 | 1.42E-17 | 1.19E-19 | -1.95 | 1.94E-15 | 8.90E-18 | -1.67 |
| 238151_at |  | 7.05E-21 | 2.80E-23 | -1.59 | 6.74E-21 | 2.26E-23 | -1.48 | 1.63E-16 | 5.24E-19 | -1.68 |
| 228528_at | LOC100286909 | 5.43E-14 | 1.74E-15 | -1.59 | 3.98E-05 | 5.78E-06 | -0.89 | 4.74E-06 | 4.09E-07 | -1.37 |
| 206331_at | CALCRL | 1.51E-12 | 6.68E-14 | -1.59 | 8.16E-12 | 2.56E-13 | -1.80 | 2.55E-14 | 1.65E-16 | -1.52 |
| 228692_at | PREX2 | 2.56E-20 | 1.28E-22 | -1.58 | 1.44E-10 | 6.02E-12 | -1.40 | 2.35E-09 | 7.39E-11 | -1.91 |
| 226071_at | ADAMTSL4 | 4.88E-14 | 1.55E-15 | -1.58 | 5.77E-09 | 3.43E-10 | -1.27 | 1.22E-06 | 8.77E-08 | -0.84 |
| 228967_at | EIF1 | 1.28E-20 | 5.68E-23 | -1.58 | 4.69E-18 | 3.40E-20 | -1.29 | 6.52E-10 | 1.73E-11 | -1.33 |
| 205883_at | ZBTB16 | 9.80E-07 | 1.43E-07 | -1.58 | 6.72E-09 | 4.05E-10 | -2.17 | 2.09E-11 | 3.46E-13 | -2.52 |
| 225673_at | MYADM | 3.16E-19 | 2.32E-21 | -1.57 | 1.70E-15 | 2.35E-17 | -1.31 | 2.61E-10 | 6.02E-12 | -0.89 |
| 226322_at | TMTC1 | 8.75E-12 | 4.60E-13 | -1.57 | 1.83E-13 | 3.90E-15 | -1.69 | 1.27E-12 | 1.31E-14 | -2.02 |
| 227554_at | MAGI2-AS3 | 2.37E-19 | 1.66E-21 | -1.57 | 5.31E-13 | 1.26E-14 | -1.02 | 4.98E-09 | 1.73E-10 | -1.05 |
| 1556314_a_at |  | 1.62E-15 | 3.66E-17 | -1.57 | 3.74E-15 | 5.58E-17 | -1.53 | 1.40E-14 | 8.36E-17 | -1.63 |
| 225855_at | EPB41L5 | 7.50E-18 | 8.70E-20 | -1.57 | 8.05E-14 | 1.60E-15 | -1.34 | 3.76E-08 | 1.68E-09 | -1.27 |
| 228554_at | PGR | 5.12E-18 | 5.69E-20 | -1.56 | 1.27E-08 | 8.12E-10 | -0.90 | 2.46E-05 | 2.66E-06 | -1.22 |
| 219563_at | LINC00341 | 5.41E-21 | 2.03E-23 | -1.56 | 9.96E-16 | 1.31E-17 | -1.42 | 3.97E-15 | 1.96E-17 | -1.72 |
| 204959_at | MNDA | 1.32E-13 | 4.65E-15 | -1.56 | 7.03E-10 | 3.42E-11 | -1.50 | 6.08E-06 | 5.46E-07 | -0.90 |
| 203961_at | NEBL | 1.17E-15 | 2.55E-17 | -1.56 | 1.20E-13 | 2.46E-15 | -1.50 | 3.90E-08 | 1.75E-09 | -1.23 |
| 231001_at | FIBIN | 1.77E-10 | 1.21E-11 | -1.56 | 4.39E-20 | 1.82E-22 | -2.01 | 1.34E-12 | 1.40E-14 | -2.11 |
| 1556321_a_at |  | 3.27E-16 | 6.14E-18 | -1.56 | 2.84E-12 | 8.00E-14 | -0.96 | 3.92E-13 | 3.55E-15 | -1.18 |
| 212636_at | QKI | 2.41E-19 | 1.70E-21 | -1.56 | 2.48E-23 | 4.54E-26 | -1.72 | 5.11E-16 | 1.95E-18 | -1.19 |
| 236313_at | CDKN2B | 6.60E-13 | 2.72E-14 | -1.56 | 7.64E-11 | 3.00E-12 | -1.55 | 1.03E-05 | 9.91E-07 | -1.31 |
| 229151_at | SLC14A1 | 4.43E-12 | 2.18E-13 | -1.55 | 6.27E-11 | 2.41E-12 | -1.72 | 1.93E-08 | 7.89E-10 | -2.35 |
| 1554503_a_at | OSCAR | 1.07E-16 | 1.73E-18 | -1.55 | 2.40E-11 | 8.37E-13 | -1.01 | 1.59E-07 | 8.50E-09 | -0.96 |
| 212448_at | NEDD4L | 4.55E-15 | 1.15E-16 | -1.55 | 9.71E-15 | 1.58E-16 | -1.51 | 2.33E-08 | 9.79E-10 | -1.45 |
| 215184_at | DAPK2 | 6.15E-20 | 3.61E-22 | -1.55 | 2.38E-15 | 3.38E-17 | -1.26 | 2.86E-08 | 1.24E-09 | -1.19 |
| 226694_at | PALM2-AKAP2///AKAP2 | 3.05E-23 | 4.24E-26 | -1.55 | 5.68E-24 | 8.62E-27 | -1.74 | 1.03E-16 | 2.93E-19 | -1.19 |
| 225782_at | MSRB3 | 4.63E-15 | 1.17E-16 | -1.55 | 2.72E-09 | 1.51E-10 | -1.32 | 1.09E-09 | 3.08E-11 | -1.30 |
| 220088_at | C5AR1 | 3.63E-15 | 8.90E-17 | -1.55 | 2.33E-10 | 1.02E-11 | -1.52 | 1.44E-09 | 4.23E-11 | -1.35 |
| 1552309_a_at | NEXN | 1.94E-16 | 3.46E-18 | -1.55 | 1.98E-08 | 1.32E-09 | -1.34 | 6.03E-09 | 2.14E-10 | -1.26 |
| 34210_at | CD52 | 2.11E-14 | 6.18E-16 | -1.55 | 1.49E-09 | 7.81E-11 | -1.26 | 2.33E-05 | 2.49E-06 | -1.02 |
| 204042_at | WASF3 | 7.34E-16 | 1.52E-17 | -1.55 | 9.02E-14 | 1.81E-15 | -1.55 | 3.38E-10 | 8.18E-12 | -1.45 |
| 232027_at | SYNE1 | 2.44E-19 | 1.73E-21 | -1.55 | 1.29E-20 | 4.66E-23 | -1.48 | 5.68E-09 | 2.00E-10 | -1.66 |
| 202222_s_at | DES | 4.43E-19 | 3.43E-21 | -1.54 | 7.44E-17 | 7.41E-19 | -1.45 | 6.39E-08 | 3.05E-09 | -1.43 |
| 1554079_at | GALNT18 | 2.41E-18 | 2.46E-20 | -1.54 | 9.47E-16 | 1.23E-17 | -1.18 | 5.80E-07 | 3.76E-08 | -0.97 |
| 49452_at | ACACB | 5.85E-18 | 6.58E-20 | -1.54 | 6.70E-12 | 2.07E-13 | -1.24 | 1.39E-11 | 2.19E-13 | -1.39 |
| 242051_at |  | 3.79E-18 | 4.03E-20 | -1.54 | 4.27E-11 | 1.58E-12 | -1.33 | 2.69E-14 | 1.76E-16 | -1.50 |
| 204482_at | CLDN5 | 8.32E-16 | 1.74E-17 | -1.54 | 3.91E-22 | 9.52E-25 | -2.11 | 2.07E-18 | 3.29E-21 | -2.52 |
| 226022_at | SASH1 | 4.65E-20 | 2.64E-22 | -1.54 | 1.30E-19 | 6.11E-22 | -1.61 | 2.32E-21 | 1.06E-24 | -1.54 |
| 213706_at | GPD1 | 4.32E-18 | 4.67E-20 | -1.54 | 2.25E-18 | 1.50E-20 | -0.91 | 1.09E-11 | 1.65E-13 | -2.81 |
| 220570_at | RETN | 7.45E-13 | 3.12E-14 | -1.54 | 5.30E-06 | 6.22E-07 | -1.00 | 8.96E-06 | 8.45E-07 | -1.50 |
| 231841_s_at | KIAA1462 | 9.15E-14 | 3.10E-15 | -1.54 | 2.15E-16 | 2.39E-18 | -2.02 | 1.80E-11 | 2.94E-13 | -1.44 |
| 214043_at | PTPRD | 2.02E-13 | 7.39E-15 | -1.53 | 2.01E-06 | 2.12E-07 | -1.05 | 1.83E-07 | 1.00E-08 | -1.35 |
| 212254_s_at | DST | 1.33E-18 | 1.24E-20 | -1.53 | 1.02E-13 | 2.05E-15 | -1.15 | 5.75E-10 | 1.49E-11 | -1.09 |
| 213438_at | NFASC | 2.95E-19 | 2.15E-21 | -1.53 | 4.02E-15 | 6.04E-17 | -1.46 | 1.47E-08 | 5.76E-10 | -1.87 |
| 205978_at | KL | 1.76E-13 | 6.34E-15 | -1.53 | 5.69E-24 | 8.74E-27 | -2.27 | 1.92E-16 | 6.26E-19 | -2.40 |
| 204570_at | COX7A1 | 3.74E-19 | 2.79E-21 | -1.53 | 2.89E-17 | 2.58E-19 | -1.36 | 5.66E-11 | 1.06E-12 | -1.41 |
| 226731_at | ITGA1 | 1.38E-17 | 1.77E-19 | -1.53 | 5.33E-12 | 1.60E-13 | -1.13 | 2.70E-11 | 4.63E-13 | -1.22 |
| 230720_at | RNF182 | 8.89E-16 | 1.87E-17 | -1.53 | 3.56E-16 | 4.15E-18 | -1.59 | 5.79E-15 | 3.03E-17 | -2.26 |
| 243041_s_at |  | 9.28E-14 | 3.15E-15 | -1.53 | 3.89E-08 | 2.75E-09 | -1.08 | 3.58E-08 | 1.59E-09 | -1.06 |
| 228335_at | CLDN11 | 3.99E-12 | 1.94E-13 | -1.53 | 2.76E-13 | 6.17E-15 | -1.76 | 1.13E-06 | 8.10E-08 | -1.49 |
| 218625_at | NRN1 | 9.20E-14 | 3.12E-15 | -1.53 | 1.48E-15 | 2.03E-17 | -1.82 | 1.37E-12 | 1.44E-14 | -1.81 |
| 229004_at | ADAMTS15 | 1.42E-14 | 4.02E-16 | -1.53 | 6.08E-14 | 1.18E-15 | -1.17 | 1.93E-10 | 4.25E-12 | -1.91 |
| 219777_at | GIMAP6 | 7.02E-17 | 1.07E-18 | -1.53 | 1.28E-19 | 6.01E-22 | -1.96 | 1.41E-12 | 1.48E-14 | -1.55 |
| 219371_s_at | KLF2 | 7.83E-17 | 1.22E-18 | -1.52 | 2.32E-17 | 2.04E-19 | -1.67 | 5.01E-06 | 4.36E-07 | -1.07 |
| 233289_at |  | 2.43E-14 | 7.23E-16 | -1.52 | 9.93E-07 | 9.74E-08 | -0.83 | 1.99E-17 | 4.52E-20 | -2.41 |
| 207574_s_at | GADD45B | 1.10E-11 | 5.88E-13 | -1.52 | 1.15E-12 | 2.95E-14 | -1.50 | 1.79E-05 | 1.85E-06 | -1.05 |
| 205856_at | SLC14A1 | 8.55E-10 | 6.75E-11 | -1.52 | 4.86E-10 | 2.28E-11 | -1.67 | 8.01E-06 | 7.46E-07 | -1.46 |
| 228532_at | C1orf162 | 1.05E-14 | 2.84E-16 | -1.52 | 5.87E-11 | 2.24E-12 | -1.09 | 2.00E-07 | 1.10E-08 | -0.94 |
| 213515_x_at | HBG2///HBG1 | 4.47E-08 | 5.00E-09 | -1.52 | 4.50E-15 | 6.89E-17 | -1.82 | 9.09E-12 | 1.33E-13 | -2.04 |
| 207496_at | MS4A2 | 2.18E-14 | 6.42E-16 | -1.52 | 7.63E-08 | 5.77E-09 | -1.20 | 2.25E-05 | 2.41E-06 | -1.42 |
| 1552553_a_at | NLRC4 | 3.85E-18 | 4.11E-20 | -1.52 | 2.38E-15 | 3.38E-17 | -1.15 | 3.53E-09 | 1.18E-10 | -1.15 |
| 214366_s_at | ALOX5 | 6.36E-14 | 2.08E-15 | -1.52 | 1.91E-09 | 1.02E-10 | -1.05 | 5.71E-08 | 2.70E-09 | -1.08 |
| 208982_at | PECAM1 | 1.35E-20 | 6.02E-23 | -1.52 | 2.73E-23 | 5.09E-26 | -1.50 | 1.25E-15 | 5.44E-18 | -1.18 |
| 225968_at | PRICKLE2 | 1.28E-18 | 1.18E-20 | -1.52 | 7.35E-13 | 1.80E-14 | -1.10 | 4.75E-12 | 6.21E-14 | -1.09 |
| 209168_at | GPM6B | 5.79E-17 | 8.67E-19 | -1.52 | 3.69E-19 | 1.96E-21 | -1.99 | 6.01E-11 | 1.14E-12 | -1.67 |
| 207317_s_at | CASQ2 | 3.39E-18 | 3.58E-20 | -1.51 | 1.71E-09 | 9.07E-11 | -0.79 | 1.07E-05 | 1.04E-06 | -2.04 |
| 215016_x_at | DST | 6.71E-19 | 5.52E-21 | -1.51 | 2.40E-13 | 5.31E-15 | -1.14 | 1.47E-09 | 4.32E-11 | -1.03 |
| 219054_at |  | 8.82E-15 | 2.35E-16 | -1.51 | 9.60E-10 | 4.82E-11 | -1.15 | 1.84E-05 | 1.91E-06 | -1.17 |
| 229302_at | TMEM178A | 2.55E-10 | 1.81E-11 | -1.51 | 4.95E-16 | 6.11E-18 | -1.87 | 2.77E-10 | 6.43E-12 | -1.77 |
| 201328_at | ETS2 | 1.23E-18 | 1.12E-20 | -1.51 | 1.76E-09 | 9.35E-11 | -0.93 | 5.11E-06 | 4.47E-07 | -0.96 |
| 225602_at | GLIPR2 | 1.09E-20 | 4.70E-23 | -1.51 | 1.34E-14 | 2.28E-16 | -1.09 | 1.62E-16 | 5.18E-19 | -1.31 |
| 217897_at | FXYD6 | 3.41E-19 | 2.53E-21 | -1.51 | 3.00E-21 | 9.43E-24 | -1.42 | 1.62E-11 | 2.60E-13 | -1.43 |
| 205027_s_at | MAP3K8 | 4.54E-12 | 2.24E-13 | -1.51 | 1.66E-13 | 3.50E-15 | -1.63 | 9.53E-10 | 2.64E-11 | -1.33 |
| 228218_at | LSAMP | 8.28E-19 | 7.11E-21 | -1.51 | 8.07E-11 | 3.19E-12 | -1.14 | 7.83E-05 | 9.94E-06 | -1.07 |
| 220287_at | ADAMTS9 | 3.98E-11 | 2.38E-12 | -1.51 | 2.19E-05 | 2.98E-06 | -1.12 | 1.05E-11 | 1.56E-13 | -1.96 |
| 203485_at | RTN1 | 3.37E-13 | 1.30E-14 | -1.51 | 1.37E-10 | 5.72E-12 | -1.26 | 1.81E-06 | 1.37E-07 | -0.98 |
| 229893_at | FRMD3 | 3.67E-19 | 2.74E-21 | -1.51 | 8.51E-31 | 1.71E-34 | -1.88 | 9.33E-14 | 7.13E-16 | -1.54 |
| 225557_at | CSRNP1 | 4.05E-15 | 1.00E-16 | -1.50 | 5.52E-19 | 3.14E-21 | -1.73 | 1.10E-09 | 3.11E-11 | -1.48 |
| 223449_at | SEMA6A | 1.38E-12 | 6.06E-14 | -1.50 | 2.63E-24 | 3.51E-27 | -1.62 | 5.38E-15 | 2.76E-17 | -2.12 |
| 205111_s_at | PLCE1 | 2.32E-17 | 3.16E-19 | -1.50 | 6.32E-11 | 2.43E-12 | -0.99 | 7.16E-08 | 3.48E-09 | -1.09 |
| 226673_at | SH2D3C | 1.30E-18 | 1.20E-20 | -1.50 | 4.93E-24 | 7.39E-27 | -1.55 | 3.42E-17 | 8.46E-20 | -1.61 |
| 219167_at | RASL12 | 2.46E-18 | 2.51E-20 | -1.50 | 3.61E-15 | 5.37E-17 | -1.13 | 1.34E-12 | 1.39E-14 | -1.41 |
| 232298_at | MBNL1-AS1 | 6.45E-19 | 5.27E-21 | -1.50 | 5.53E-14 | 1.06E-15 | -1.27 | 3.46E-08 | 1.53E-09 | -1.34 |
| 200795_at | SPARCL1 | 2.43E-16 | 4.45E-18 | -1.50 | 3.63E-13 | 8.35E-15 | -1.27 | 1.10E-07 | 5.69E-09 | -0.93 |
| 226244_at | CLEC14A | 2.15E-18 | 2.17E-20 | -1.50 | 2.22E-25 | 1.99E-28 | -1.57 | 7.78E-18 | 1.57E-20 | -2.18 |
| 219908_at | DKK2 | 6.72E-12 | 3.45E-13 | -1.50 | 1.16E-13 | 2.38E-15 | -1.85 | 5.05E-11 | 9.32E-13 | -1.92 |
| 1552318_at | GIMAP1 | 3.68E-17 | 5.20E-19 | -1.50 | 4.37E-17 | 4.10E-19 | -1.79 | 2.64E-13 | 2.24E-15 | -1.77 |
| 226931_at | TMTC1 | 1.67E-10 | 1.13E-11 | -1.49 | 7.01E-09 | 4.25E-10 | -1.53 | 2.85E-10 | 6.64E-12 | -1.80 |
| 228094_at | AMICA1 | 8.52E-18 | 1.00E-19 | -1.49 | 9.33E-10 | 4.66E-11 | -0.93 | 8.60E-05 | 1.11E-05 | -0.89 |
| 207442_at | CSF3 | 3.54E-08 | 3.89E-09 | -1.49 | 2.29E-08 | 1.54E-09 | -1.41 | 3.02E-08 | 1.31E-09 | -2.24 |
| 224339_s_at | ANGPTL1 | 2.80E-16 | 5.17E-18 | -1.49 | 3.02E-18 | 2.08E-20 | -1.58 | 5.54E-15 | 2.86E-17 | -2.99 |
| 231925_at |  | 1.91E-15 | 4.40E-17 | -1.49 | 1.31E-14 | 2.21E-16 | -1.52 | 5.43E-17 | 1.43E-19 | -1.90 |
| 242340_at |  | 2.27E-16 | 4.11E-18 | -1.49 | 1.24E-21 | 3.62E-24 | -1.82 | 9.83E-13 | 9.82E-15 | -2.96 |
| 222486_s_at | ADAMTS1 | 7.22E-08 | 8.47E-09 | -1.49 | 2.17E-10 | 9.45E-12 | -1.87 | 3.26E-12 | 3.97E-14 | -2.12 |
| 203434_s_at | MME | 4.32E-08 | 4.80E-09 | -1.49 | 4.24E-18 | 3.03E-20 | -2.40 | 2.72E-12 | 3.17E-14 | -2.62 |
| 237390_at |  | 1.78E-15 | 4.07E-17 | -1.49 | 4.33E-23 | 8.40E-26 | -1.89 | 3.14E-10 | 7.47E-12 | -4.74 |
| 203638_s_at | FGFR2 | 1.05E-11 | 5.60E-13 | -1.49 | 3.16E-08 | 2.19E-09 | -0.91 | 2.06E-06 | 1.59E-07 | -1.22 |
| 218775_s_at | WWC2 | 3.95E-15 | 9.74E-17 | -1.48 | 2.84E-12 | 7.99E-14 | -1.43 | 1.47E-16 | 4.56E-19 | -1.38 |
| 202920_at | ANK2 | 2.86E-11 | 1.66E-12 | -1.48 | 2.97E-09 | 1.66E-10 | -1.39 | 8.34E-05 | 1.07E-05 | -1.04 |
| 228540_at | QKI | 3.44E-23 | 4.90E-26 | -1.48 | 9.94E-33 | 9.09E-37 | -1.22 | 1.39E-18 | 2.06E-21 | -1.20 |
| 209735_at | ABCG2 | 4.17E-15 | 1.04E-16 | -1.48 | 5.76E-10 | 2.75E-11 | -1.08 | 2.71E-08 | 1.16E-09 | -1.19 |
| 209656_s_at | TMEM47 | 6.59E-15 | 1.72E-16 | -1.48 | 4.34E-13 | 1.02E-14 | -1.46 | 1.18E-10 | 2.45E-12 | -1.27 |
| 227779_at | ECSCR | 2.92E-21 | 9.72E-24 | -1.48 | 3.75E-25 | 3.71E-28 | -1.72 | 2.27E-18 | 3.65E-21 | -2.35 |
| 241722_x_at |  | 2.89E-14 | 8.75E-16 | -1.47 | 5.71E-05 | 8.59E-06 | -1.23 | 7.11E-07 | 4.75E-08 | -1.50 |
| 222304_x_at | OR7E47P | 5.75E-21 | 2.20E-23 | -1.47 | 1.82E-18 | 1.19E-20 | -1.23 | 3.14E-09 | 1.03E-10 | -1.12 |
| 202014_at | PPP1R15A | 6.19E-13 | 2.54E-14 | -1.47 | 1.45E-17 | 1.22E-19 | -1.61 | 2.77E-05 | 3.03E-06 | -0.90 |
| 205033_s_at | DEFA1B///DEFA3///DEFA1 | 2.45E-08 | 2.60E-09 | -1.47 | 1.01E-14 | 1.65E-16 | -2.29 | 1.85E-09 | 5.60E-11 | -1.47 |
| 242277_at |  | 2.85E-07 | 3.75E-08 | -1.47 | 7.70E-05 | 1.20E-05 | -1.27 | 3.26E-12 | 3.97E-14 | -1.22 |
| 232224_at | MASP1 | 5.31E-20 | 3.08E-22 | -1.47 | 1.09E-27 | 5.60E-31 | -1.51 | 6.08E-18 | 1.13E-20 | -2.62 |
| 232629_at | PROK2 | 2.41E-07 | 3.13E-08 | -1.47 | 7.42E-10 | 3.63E-11 | -1.98 | 2.96E-11 | 5.13E-13 | -2.49 |
| 229797_at | MCOLN3 | 4.08E-11 | 2.44E-12 | -1.46 | 5.71E-09 | 3.39E-10 | -1.11 | 2.91E-06 | 2.36E-07 | -1.34 |
| 232404_at | SHROOM4 | 7.16E-20 | 4.32E-22 | -1.46 | 5.24E-17 | 5.01E-19 | -1.00 | 1.30E-10 | 2.74E-12 | -1.89 |
| 227289_at | PCDH17 | 4.74E-12 | 2.35E-13 | -1.46 | 2.44E-16 | 2.76E-18 | -1.72 | 8.79E-12 | 1.27E-13 | -1.61 |
| 231035_s_at | OTUD1 | 2.01E-14 | 5.86E-16 | -1.46 | 1.01E-12 | 2.57E-14 | -1.05 | 4.91E-10 | 1.25E-11 | -0.76 |
| 228333_at | ZEB2 | 5.45E-18 | 6.07E-20 | -1.46 | 3.89E-13 | 9.03E-15 | -1.29 | 3.18E-12 | 3.86E-14 | -1.42 |
| 221276_s_at | SYNC | 1.57E-14 | 4.48E-16 | -1.46 | 4.28E-08 | 3.05E-09 | -1.03 | 2.56E-07 | 1.47E-08 | -0.73 |
| 37028_at | PPP1R15A | 2.64E-14 | 7.95E-16 | -1.46 | 4.26E-18 | 3.05E-20 | -1.57 | 3.84E-06 | 3.23E-07 | -0.91 |
| 204777_s_at | MAL | 4.01E-15 | 9.89E-17 | -1.46 | 4.70E-16 | 5.76E-18 | -1.35 | 3.64E-06 | 3.03E-07 | -1.39 |
| 205112_at | PLCE1 | 5.31E-17 | 7.86E-19 | -1.46 | 7.26E-13 | 1.78E-14 | -1.12 | 1.48E-08 | 5.79E-10 | -1.12 |
| 1555579_s_at | PTPRM | 4.31E-17 | 6.20E-19 | -1.45 | 5.39E-20 | 2.33E-22 | -1.38 | 2.41E-11 | 4.09E-13 | -0.98 |
| 235044_at | CYYR1 | 8.14E-15 | 2.16E-16 | -1.45 | 7.29E-17 | 7.24E-19 | -1.71 | 1.18E-15 | 5.06E-18 | -1.72 |
| 230179_at | LOC285812 | 2.62E-14 | 7.86E-16 | -1.45 | 1.27E-14 | 2.13E-16 | -1.53 | 3.33E-15 | 1.61E-17 | -1.71 |
| 205507_at | ARHGEF15 | 2.02E-20 | 9.53E-23 | -1.45 | 6.18E-20 | 2.72E-22 | -1.19 | 8.58E-20 | 7.38E-23 | -1.47 |
| 213844_at | HOXA5 | 1.64E-11 | 9.12E-13 | -1.45 | 6.84E-12 | 2.11E-13 | -1.41 | 9.92E-12 | 1.47E-13 | -1.63 |
| 217525_at | OLFML1 | 1.08E-16 | 1.74E-18 | -1.45 | 1.26E-12 | 3.26E-14 | -1.33 | 2.18E-10 | 4.90E-12 | -1.33 |
| 213364_s_at | SNX1 | 2.01E-22 | 3.89E-25 | -1.45 | 2.21E-16 | 2.48E-18 | -1.15 | 1.48E-10 | 3.15E-12 | -0.92 |
| 243481_at | RHOJ | 5.01E-18 | 5.54E-20 | -1.45 | 1.40E-18 | 8.80E-21 | -1.33 | 8.12E-14 | 6.07E-16 | -1.87 |
| 219937_at | TRHDE | 3.36E-20 | 1.76E-22 | -1.44 | 2.66E-17 | 2.36E-19 | -1.58 | 4.52E-11 | 8.19E-13 | -2.32 |
| 206049_at | SELP | 2.26E-17 | 3.06E-19 | -1.44 | 3.47E-17 | 3.15E-19 | -1.43 | 4.14E-10 | 1.04E-11 | -1.43 |
| 200911_s_at | TACC1 | 8.28E-26 | 2.88E-29 | -1.44 | 9.66E-20 | 4.40E-22 | -1.11 | 7.23E-12 | 1.00E-13 | -0.87 |
| 202760_s_at | PALM2-AKAP2///AKAP2 | 1.12E-16 | 1.84E-18 | -1.44 | 9.56E-18 | 7.63E-20 | -1.98 | 1.96E-17 | 4.40E-20 | -1.51 |
| 237400_at | ATP5S | 1.12E-15 | 2.42E-17 | -1.43 | 1.54E-07 | 1.25E-08 | -1.02 | 1.11E-05 | 1.08E-06 | -0.96 |
| 231859_at | C14orf132 | 7.41E-17 | 1.15E-18 | -1.43 | 4.70E-14 | 8.92E-16 | -1.25 | 8.42E-10 | 2.31E-11 | -1.80 |
| 213817_at | IRAK3 | 5.99E-14 | 1.94E-15 | -1.43 | 1.13E-05 | 1.44E-06 | -1.13 | 1.87E-06 | 1.42E-07 | -1.14 |
| 205637_s_at | SH3GL3 | 1.13E-16 | 1.86E-18 | -1.43 | 2.07E-28 | 7.58E-32 | -1.59 | 1.39E-14 | 8.27E-17 | -2.15 |
| 213069_at | HEG1 | 6.78E-19 | 5.60E-21 | -1.43 | 2.34E-17 | 2.06E-19 | -1.42 | 1.93E-14 | 1.20E-16 | -1.23 |
| 227923_at | SHANK3 | 1.09E-16 | 1.77E-18 | -1.43 | 5.03E-18 | 3.71E-20 | -1.60 | 5.11E-14 | 3.60E-16 | -1.98 |
| 227618_at |  | 5.45E-21 | 2.05E-23 | -1.42 | 3.98E-11 | 1.46E-12 | -0.90 | 4.21E-09 | 1.44E-10 | -1.24 |
| 234998_at | RAB11A | 2.51E-19 | 1.79E-21 | -1.42 | 3.92E-13 | 9.13E-15 | -1.24 | 2.49E-13 | 2.11E-15 | -1.08 |
| 219165_at | PDLIM2 | 1.29E-16 | 2.19E-18 | -1.42 | 3.84E-19 | 2.05E-21 | -1.13 | 7.68E-14 | 5.70E-16 | -1.06 |
| 218656_s_at | LHFP | 1.00E-16 | 1.61E-18 | -1.42 | 9.07E-17 | 9.17E-19 | -1.39 | 1.44E-13 | 1.16E-15 | -1.42 |
| 1557094_at |  | 2.23E-15 | 5.22E-17 | -1.42 | 6.26E-22 | 1.67E-24 | -1.60 | 2.12E-10 | 4.74E-12 | -2.81 |
| 211986_at | AHNAK | 1.91E-20 | 8.92E-23 | -1.42 | 2.69E-18 | 1.83E-20 | -1.23 | 8.92E-12 | 1.30E-13 | -1.13 |
| 209683_at | FAM49A | 4.83E-18 | 5.32E-20 | -1.42 | 6.35E-16 | 8.04E-18 | -1.30 | 1.38E-08 | 5.37E-10 | -1.09 |
| 225721_at | SYNPO2 | 3.70E-17 | 5.25E-19 | -1.42 | 4.45E-09 | 2.58E-10 | -1.03 | 6.87E-07 | 4.55E-08 | -1.38 |
| 228653_at | SAMD5 | 6.66E-17 | 1.01E-18 | -1.42 | 5.78E-10 | 2.76E-11 | -1.04 | 1.01E-07 | 5.15E-09 | -1.52 |
| 228618_at | PEAR1 | 4.93E-18 | 5.44E-20 | -1.41 | 7.02E-21 | 2.40E-23 | -1.53 | 5.11E-16 | 1.95E-18 | -1.62 |
| 210190_at | STX11 | 3.24E-15 | 7.84E-17 | -1.41 | 1.23E-16 | 1.28E-18 | -1.64 | 2.05E-08 | 8.42E-10 | -2.19 |
| 220646_s_at | KLRF1 | 8.52E-12 | 4.47E-13 | -1.41 | 1.07E-14 | 1.76E-16 | -1.94 | 1.09E-07 | 5.59E-09 | -1.56 |
| 206637_at | P2RY14 | 4.99E-16 | 9.76E-18 | -1.41 | 3.17E-16 | 3.66E-18 | -1.66 | 1.50E-13 | 1.23E-15 | -1.35 |
| 200671_s_at | SPTBN1 | 5.30E-15 | 1.35E-16 | -1.41 | 7.08E-14 | 1.39E-15 | -1.96 | 4.08E-11 | 7.33E-13 | -1.37 |
| 230661_at | LOC286191 | 1.39E-17 | 1.78E-19 | -1.41 | 1.27E-08 | 8.14E-10 | -1.03 | 2.16E-11 | 3.60E-13 | -3.02 |
| 202995_s_at | FBLN1 | 4.67E-08 | 5.24E-09 | -1.41 | 2.49E-08 | 1.69E-09 | -1.39 | 3.34E-08 | 1.47E-09 | -1.62 |
| 209242_at | PEG3 | 6.29E-11 | 3.92E-12 | -1.41 | 2.35E-05 | 3.23E-06 | -0.95 | 2.71E-05 | 2.96E-06 | -1.17 |
| 226140_s_at | OTUD1 | 1.07E-21 | 2.81E-24 | -1.41 | 1.79E-23 | 3.08E-26 | -1.30 | 1.86E-09 | 5.64E-11 | -0.72 |
| 238061_at | LGI3 | 1.50E-23 | 1.82E-26 | -1.41 | 6.39E-18 | 4.86E-20 | -1.28 | 4.62E-19 | 5.54E-22 | -3.30 |
| 208790_s_at | PTRF | 4.38E-12 | 2.15E-13 | -1.41 | 2.48E-15 | 3.53E-17 | -1.57 | 3.02E-10 | 7.13E-12 | -0.84 |
| 228071_at | GIMAP7 | 3.02E-13 | 1.16E-14 | -1.40 | 3.06E-13 | 6.89E-15 | -1.64 | 1.75E-09 | 5.28E-11 | -1.34 |
| 201842_s_at | EFEMP1 | 5.34E-13 | 2.17E-14 | -1.40 | 5.54E-13 | 1.33E-14 | -1.39 | 2.01E-06 | 1.55E-07 | -1.02 |
| 212372_at | MYH10 | 5.01E-17 | 7.35E-19 | -1.40 | 1.68E-11 | 5.64E-13 | -1.17 | 5.08E-07 | 3.24E-08 | -0.84 |
| 202796_at | SYNPO | 1.83E-19 | 1.22E-21 | -1.40 | 4.90E-11 | 1.84E-12 | -0.93 | 1.64E-07 | 8.84E-09 | -0.94 |
| 225915_at | CAB39L | 3.09E-11 | 1.81E-12 | -1.40 | 1.07E-10 | 4.33E-12 | -1.49 | 6.46E-14 | 4.68E-16 | -1.54 |
| 1554690_a_at | TACC1 | 1.02E-19 | 6.34E-22 | -1.40 | 1.09E-11 | 3.51E-13 | -1.21 | 1.24E-16 | 3.65E-19 | -1.45 |
| 243584_at |  | 4.59E-14 | 1.45E-15 | -1.40 | 6.20E-07 | 5.81E-08 | -1.20 | 4.43E-10 | 1.12E-11 | -1.56 |
| 206618_at | IL18R1 | 9.92E-13 | 4.25E-14 | -1.40 | 1.31E-12 | 3.41E-14 | -1.48 | 9.47E-11 | 1.91E-12 | -1.49 |
| 63305_at | PKNOX2 | 2.71E-21 | 8.83E-24 | -1.40 | 6.70E-24 | 1.06E-26 | -1.23 | 3.96E-12 | 5.01E-14 | -1.54 |
| 239952_at | ZEB1 | 7.45E-17 | 1.16E-18 | -1.39 | 8.01E-21 | 2.80E-23 | -1.40 | 2.61E-17 | 6.20E-20 | -1.36 |
| 219282_s_at | TRPV2 | 1.07E-18 | 9.56E-21 | -1.39 | 3.68E-14 | 6.80E-16 | -0.90 | 1.32E-09 | 3.81E-11 | -1.02 |
| 238009_at |  | 1.49E-22 | 2.62E-25 | -1.39 | 2.16E-09 | 1.17E-10 | -1.05 | 7.54E-11 | 1.47E-12 | -1.36 |
| 204797_s_at | EML1 | 1.02E-14 | 2.76E-16 | -1.39 | 3.25E-12 | 9.26E-14 | -1.30 | 7.04E-16 | 2.88E-18 | -1.24 |
| 61297_at | CASKIN2 | 2.76E-17 | 3.82E-19 | -1.39 | 2.90E-22 | 6.84E-25 | -1.29 | 1.21E-17 | 2.56E-20 | -1.45 |
| 212915_at | PDZRN3 | 1.28E-14 | 3.58E-16 | -1.38 | 8.82E-07 | 8.53E-08 | -0.91 | 3.33E-05 | 3.74E-06 | -0.96 |
| 240715_at | TBX5 | 5.67E-15 | 1.45E-16 | -1.38 | 5.34E-09 | 3.14E-10 | -1.03 | 5.93E-12 | 7.96E-14 | -1.70 |
| 204073_s_at | MYRF | 4.34E-11 | 2.62E-12 | -1.38 | 5.01E-17 | 4.77E-19 | -1.56 | 2.84E-07 | 1.66E-08 | -1.59 |
| 225177_at | RAB11FIP1 | 8.04E-14 | 2.69E-15 | -1.38 | 3.77E-16 | 4.46E-18 | -1.29 | 3.65E-06 | 3.05E-07 | -1.00 |
| 229844_at | FOXP1 | 3.73E-20 | 2.01E-22 | -1.38 | 4.15E-15 | 6.27E-17 | -0.95 | 3.65E-12 | 4.53E-14 | -1.02 |
| 206377_at | FOXF2 | 5.69E-12 | 2.88E-13 | -1.38 | 9.11E-08 | 7.03E-09 | -1.00 | 2.09E-08 | 8.58E-10 | -1.26 |
| 205651_x_at | RAPGEF4 | 1.00E-16 | 1.61E-18 | -1.38 | 1.00E-17 | 8.00E-20 | -1.77 | 3.68E-21 | 1.95E-24 | -1.95 |
| 227719_at | SMAD9 | 1.88E-08 | 1.95E-09 | -1.38 | 4.86E-12 | 1.45E-13 | -1.63 | 2.66E-11 | 4.53E-13 | -1.51 |
| 227463_at | ACE | 1.11E-14 | 3.03E-16 | -1.38 | 1.32E-24 | 1.59E-27 | -1.33 | 3.95E-07 | 2.42E-08 | -1.27 |
| 210146_x_at | LILRB2 | 1.79E-11 | 9.99E-13 | -1.38 | 1.16E-12 | 2.98E-14 | -1.54 | 3.16E-09 | 1.04E-10 | -1.35 |
| 1570515_a_at | FILIP1 | 2.28E-10 | 1.60E-11 | -1.37 | 1.09E-08 | 6.88E-10 | -1.05 | 2.45E-09 | 7.75E-11 | -1.68 |
| 230595_at | PGM5-AS1 | 5.71E-15 | 1.46E-16 | -1.37 | 3.94E-18 | 2.80E-20 | -0.89 | 2.86E-07 | 1.67E-08 | -2.33 |
| 239218_at | PDE1C | 2.51E-19 | 1.80E-21 | -1.37 | 1.33E-14 | 2.25E-16 | -1.26 | 1.64E-08 | 6.51E-10 | -1.50 |
| 202149_at | NEDD9 | 1.71E-11 | 9.54E-13 | -1.37 | 2.25E-12 | 6.17E-14 | -1.35 | 1.56E-07 | 8.33E-09 | -0.98 |
| 229560_at | TLR8 | 4.79E-10 | 3.61E-11 | -1.37 | 1.49E-08 | 9.66E-10 | -1.42 | 8.96E-05 | 1.16E-05 | -0.97 |
| 203813_s_at | SLIT3 | 2.25E-15 | 5.29E-17 | -1.37 | 3.27E-13 | 7.44E-15 | -1.01 | 8.90E-08 | 4.45E-09 | -1.49 |
| 205414_s_at | ARHGAP44 | 4.53E-19 | 3.52E-21 | -1.37 | 9.07E-11 | 3.62E-12 | -0.90 | 1.77E-05 | 1.82E-06 | -1.08 |
| 229222_at | ACSS3 | 4.27E-15 | 1.06E-16 | -1.37 | 1.11E-09 | 5.67E-11 | -1.15 | 8.87E-07 | 6.09E-08 | -1.08 |
| 240890_at | LOC643733 | 5.87E-17 | 8.79E-19 | -1.37 | 1.91E-16 | 2.09E-18 | -1.36 | 5.13E-11 | 9.51E-13 | -1.27 |
| 239919_at | TBX5-AS1 | 4.06E-16 | 7.76E-18 | -1.37 | 2.87E-10 | 1.28E-11 | -1.20 | 5.10E-10 | 1.31E-11 | -1.21 |
| 219315_s_at | TMEM204 | 2.93E-14 | 8.92E-16 | -1.37 | 5.07E-14 | 9.68E-16 | -1.31 | 1.83E-17 | 4.05E-20 | -1.39 |
| 202994_s_at | FBLN1 | 2.18E-13 | 8.05E-15 | -1.37 | 6.89E-09 | 4.17E-10 | -1.11 | 1.18E-07 | 6.14E-09 | -1.54 |
| 232080_at | HECW2 | 3.41E-11 | 2.02E-12 | -1.36 | 7.89E-16 | 1.01E-17 | -1.36 | 5.65E-11 | 1.05E-12 | -1.27 |
| 226713_at | CCDC50 | 4.19E-20 | 2.31E-22 | -1.36 | 4.41E-20 | 1.84E-22 | -1.33 | 2.80E-15 | 1.32E-17 | -1.52 |
| 205405_at | SEMA5A | 1.14E-16 | 1.88E-18 | -1.36 | 1.91E-20 | 7.21E-23 | -1.44 | 1.50E-13 | 1.23E-15 | -1.78 |
| 205612_at | MMRN1 | 2.70E-10 | 1.93E-11 | -1.36 | 1.69E-11 | 5.70E-13 | -2.00 | 7.58E-09 | 2.74E-10 | -2.20 |
| 213236_at | SASH1 | 2.09E-21 | 6.41E-24 | -1.36 | 1.34E-17 | 1.12E-19 | -1.31 | 6.51E-25 | 3.57E-29 | -1.73 |
| 235771_at |  | 5.00E-16 | 9.81E-18 | -1.36 | 2.80E-11 | 9.90E-13 | -1.09 | 1.73E-12 | 1.87E-14 | -1.77 |
| 1568768_s_at | BRE-AS1 | 1.68E-09 | 1.40E-10 | -1.36 | 9.48E-10 | 4.74E-11 | -1.45 | 4.02E-07 | 2.46E-08 | -2.00 |
| 243023_at |  | 2.20E-15 | 5.15E-17 | -1.36 | 1.26E-07 | 1.00E-08 | -0.81 | 1.02E-07 | 5.20E-09 | -1.18 |
| 209283_at | CRYAB | 3.25E-15 | 7.87E-17 | -1.36 | 2.53E-18 | 1.72E-20 | -1.56 | 2.09E-10 | 4.65E-12 | -1.15 |
| 227947_at | PHACTR2 | 2.11E-22 | 4.25E-25 | -1.36 | 6.12E-15 | 9.60E-17 | -1.17 | 3.49E-13 | 3.08E-15 | -1.01 |
| 203186_s_at | S100A4 | 2.96E-13 | 1.13E-14 | -1.36 | 6.90E-16 | 8.79E-18 | -1.09 | 1.63E-06 | 1.22E-07 | -1.12 |
| 219355_at | CXorf57 | 2.04E-16 | 3.66E-18 | -1.36 | 7.70E-11 | 3.03E-12 | -1.04 | 9.45E-07 | 6.55E-08 | -1.16 |
| 244745_at | RERG | 5.98E-12 | 3.03E-13 | -1.36 | 8.73E-06 | 1.08E-06 | -1.11 | 3.00E-06 | 2.44E-07 | -1.35 |
| 211685_s_at | NCALD | 9.65E-19 | 8.49E-21 | -1.36 | 5.43E-10 | 2.58E-11 | -0.83 | 4.14E-05 | 4.81E-06 | -0.89 |
| 203060_s_at | PAPSS2 | 2.14E-15 | 5.00E-17 | -1.35 | 5.31E-20 | 2.26E-22 | -1.21 | 6.62E-08 | 3.19E-09 | -0.92 |
| 202340_x_at | NR4A1 | 1.56E-10 | 1.05E-11 | -1.35 | 1.16E-16 | 1.21E-18 | -1.60 | 1.19E-05 | 1.17E-06 | -2.05 |
| 205656_at | PCDH17 | 2.12E-11 | 1.20E-12 | -1.35 | 1.92E-14 | 3.33E-16 | -1.49 | 1.10E-10 | 2.28E-12 | -1.37 |
| 244447_at |  | 4.81E-14 | 1.53E-15 | -1.35 | 2.60E-06 | 2.82E-07 | -0.96 | 2.00E-07 | 1.10E-08 | -1.74 |
| 227530_at | AKAP12 | 2.35E-09 | 2.03E-10 | -1.35 | 2.93E-14 | 5.29E-16 | -1.97 | 4.58E-11 | 8.32E-13 | -1.74 |
| 226751_at | CNRIP1 | 3.44E-16 | 6.47E-18 | -1.35 | 4.08E-16 | 4.88E-18 | -1.15 | 1.37E-10 | 2.91E-12 | -1.21 |
| 218974_at | SOBP | 2.92E-15 | 7.01E-17 | -1.35 | 3.28E-05 | 4.68E-06 | -0.77 | 1.28E-06 | 9.22E-08 | -1.18 |
| 226682_at | RORA | 3.32E-15 | 8.06E-17 | -1.35 | 3.46E-11 | 1.25E-12 | -1.22 | 1.70E-07 | 9.20E-09 | -0.85 |
| 204115_at | GNG11 | 8.76E-10 | 6.93E-11 | -1.35 | 2.62E-20 | 1.05E-22 | -1.81 | 1.33E-15 | 5.83E-18 | -1.88 |
| 226806_s_at | NFIA | 1.66E-13 | 5.95E-15 | -1.34 | 5.46E-07 | 5.03E-08 | -0.72 | 2.39E-07 | 1.35E-08 | -1.19 |
| 213058_at | TTC28 | 1.93E-17 | 2.57E-19 | -1.34 | 1.31E-14 | 2.22E-16 | -1.03 | 8.17E-12 | 1.17E-13 | -1.04 |
| 222121_at | ARHGEF26 | 1.13E-17 | 1.40E-19 | -1.34 | 2.28E-18 | 1.53E-20 | -1.93 | 1.23E-05 | 1.20E-06 | -1.46 |
| 202345_s_at | FABP5 | 3.81E-16 | 7.23E-18 | -1.34 | 7.43E-06 | 9.04E-07 | -0.85 | 3.12E-07 | 1.85E-08 | -1.19 |
| 244787_at |  | 2.41E-11 | 1.38E-12 | -1.34 | 1.93E-13 | 4.15E-15 | -1.19 | 1.13E-18 | 1.59E-21 | -2.52 |
| 208981_at | PECAM1 | 1.52E-19 | 9.90E-22 | -1.34 | 5.90E-24 | 9.17E-27 | -1.56 | 8.35E-20 | 7.02E-23 | -1.70 |
| 210078_s_at | KCNAB1 | 1.31E-12 | 5.75E-14 | -1.34 | 4.99E-09 | 2.93E-10 | -1.10 | 3.57E-10 | 8.70E-12 | -1.35 |
| 205247_at | NOTCH4 | 3.71E-17 | 5.29E-19 | -1.34 | 1.56E-19 | 7.52E-22 | -1.12 | 3.48E-19 | 3.82E-22 | -1.49 |
| 226933_s_at | ID4 | 4.17E-12 | 2.04E-13 | -1.34 | 2.32E-08 | 1.56E-09 | -1.07 | 2.76E-06 | 2.22E-07 | -1.42 |
| 209304_x_at | GADD45B | 1.21E-10 | 7.95E-12 | -1.34 | 2.00E-12 | 5.43E-14 | -1.34 | 6.61E-05 | 8.19E-06 | -1.03 |
| 235199_at | RNF125 | 2.16E-17 | 2.91E-19 | -1.34 | 6.33E-12 | 1.93E-13 | -1.14 | 3.59E-09 | 1.20E-10 | -1.01 |
| 235155_at | BDH2 | 4.94E-20 | 2.83E-22 | -1.34 | 1.89E-08 | 1.25E-09 | -0.95 | 9.15E-07 | 6.31E-08 | -0.97 |
| 238868_at | UACA | 5.98E-14 | 1.94E-15 | -1.33 | 8.32E-12 | 2.62E-13 | -1.43 | 7.62E-12 | 1.07E-13 | -1.30 |
| 43511_s_at | ARRB1 | 1.57E-14 | 4.47E-16 | -1.33 | 4.74E-25 | 4.86E-28 | -1.39 | 7.65E-14 | 5.67E-16 | -1.68 |
| 1552667_a_at | SH2D3C | 8.23E-14 | 2.76E-15 | -1.33 | 1.05E-17 | 8.50E-20 | -1.58 | 2.10E-12 | 2.32E-14 | -1.75 |
| 219761_at | CLEC1A | 2.14E-18 | 2.15E-20 | -1.33 | 2.54E-22 | 5.85E-25 | -1.46 | 6.00E-15 | 3.17E-17 | -1.68 |
| 212914_at | CBX7 | 1.39E-21 | 4.02E-24 | -1.33 | 5.96E-15 | 9.32E-17 | -1.07 | 1.74E-08 | 6.99E-10 | -0.83 |
| 206101_at | ECM2 | 5.99E-11 | 3.72E-12 | -1.33 | 8.07E-07 | 7.73E-08 | -1.07 | 1.67E-08 | 6.67E-10 | -1.37 |
| 43427_at | ACACB | 5.77E-18 | 6.48E-20 | -1.33 | 4.58E-12 | 1.35E-13 | -1.06 | 2.01E-12 | 2.20E-14 | -1.45 |
| 1555191_a_at | FHL5 | 7.60E-15 | 2.01E-16 | -1.33 | 3.62E-14 | 6.67E-16 | -1.39 | 1.34E-11 | 2.10E-13 | -2.31 |
| 208016_s_at | AGTR1 | 2.58E-14 | 7.72E-16 | -1.33 | 4.95E-14 | 9.43E-16 | -1.33 | 1.13E-14 | 6.52E-17 | -2.04 |
| 212975_at | DENND3 | 9.95E-23 | 1.64E-25 | -1.32 | 1.26E-23 | 2.07E-26 | -1.38 | 1.13E-19 | 1.09E-22 | -1.36 |
| 204236_at | FLI1 | 2.75E-16 | 5.07E-18 | -1.32 | 2.40E-13 | 5.31E-15 | -1.42 | 4.63E-13 | 4.31E-15 | -1.52 |
| 228153_at | RNF144B | 9.03E-15 | 2.42E-16 | -1.32 | 1.47E-15 | 2.01E-17 | -1.11 | 4.64E-07 | 2.91E-08 | -0.78 |
| 204069_at | MEIS1 | 1.58E-14 | 4.50E-16 | -1.32 | 1.64E-11 | 5.48E-13 | -1.02 | 3.50E-08 | 1.55E-09 | -1.08 |
| 202877_s_at | CD93 | 5.07E-14 | 1.61E-15 | -1.32 | 6.31E-17 | 6.15E-19 | -1.56 | 1.06E-17 | 2.19E-20 | -1.66 |
| 211922_s_at | CAT | 1.29E-16 | 2.18E-18 | -1.32 | 3.32E-07 | 2.89E-08 | -1.06 | 8.95E-09 | 3.30E-10 | -1.01 |
| 228748_at | CD59 | 8.41E-16 | 1.76E-17 | -1.32 | 1.74E-06 | 1.81E-07 | -0.79 | 1.67E-07 | 9.02E-09 | -0.92 |
| 205442_at | MFAP3L | 1.98E-08 | 2.07E-09 | -1.31 | 2.54E-11 | 8.92E-13 | -1.54 | 1.19E-05 | 1.16E-06 | -1.19 |
| 230482_at | ST6GALNAC5 | 2.52E-12 | 1.17E-13 | -1.31 | 3.01E-24 | 4.30E-27 | -1.95 | 1.57E-14 | 9.58E-17 | -1.92 |
| 241929_at |  | 1.45E-10 | 9.70E-12 | -1.31 | 2.93E-08 | 2.02E-09 | -1.10 | 3.13E-15 | 1.50E-17 | -2.72 |
| 226018_at | MTURN | 1.42E-09 | 1.18E-10 | -1.31 | 1.44E-13 | 2.98E-15 | -1.46 | 3.73E-06 | 3.12E-07 | -0.96 |
| 213618_at | ARAP2 | 3.84E-17 | 5.48E-19 | -1.31 | 6.21E-16 | 7.83E-18 | -1.14 | 4.84E-08 | 2.23E-09 | -0.77 |
| 228863_at | PCDH17 | 3.70E-10 | 2.71E-11 | -1.31 | 1.59E-16 | 1.71E-18 | -1.75 | 1.35E-11 | 2.11E-13 | -1.53 |
| 228396_at | PRKG1 | 1.68E-16 | 2.95E-18 | -1.31 | 1.94E-07 | 1.60E-08 | -0.82 | 4.57E-06 | 3.92E-07 | -0.82 |
| 218418_s_at | KANK2 | 4.78E-18 | 5.24E-20 | -1.31 | 2.08E-14 | 3.65E-16 | -1.09 | 2.00E-09 | 6.15E-11 | -0.98 |
| 239847_at |  | 1.88E-11 | 1.05E-12 | -1.31 | 2.08E-07 | 1.73E-08 | -0.94 | 4.48E-05 | 5.26E-06 | -0.98 |
| 228827_at | RUNX1T1 | 5.24E-12 | 2.62E-13 | -1.31 | 1.82E-10 | 7.80E-12 | -1.17 | 6.43E-08 | 3.07E-09 | -1.24 |
| 218901_at | PLSCR4 | 8.31E-17 | 1.30E-18 | -1.31 | 2.47E-07 | 2.08E-08 | -0.92 | 1.40E-06 | 1.02E-07 | -0.80 |
| 212230_at | PPAP2B | 5.03E-18 | 5.58E-20 | -1.31 | 3.23E-12 | 9.20E-14 | -1.19 | 4.80E-13 | 4.48E-15 | -1.20 |
| 236600_at | SPG20 | 4.14E-24 | 3.40E-27 | -1.31 | 4.55E-09 | 2.65E-10 | -1.04 | 2.78E-10 | 6.45E-12 | -0.90 |
| 225913_at | PEAK1 | 9.45E-21 | 3.99E-23 | -1.30 | 2.35E-18 | 1.58E-20 | -1.06 | 8.46E-12 | 1.22E-13 | -1.09 |
| 203543_s_at | KLF9 | 8.50E-09 | 8.23E-10 | -1.30 | 5.30E-11 | 2.00E-12 | -1.67 | 1.70E-15 | 7.67E-18 | -1.64 |
| 224861_at | GNAQ | 4.16E-24 | 3.50E-27 | -1.30 | 2.36E-18 | 1.59E-20 | -0.93 | 2.27E-12 | 2.56E-14 | -0.94 |
| 213067_at | MYH10 | 2.34E-10 | 1.64E-11 | -1.30 | 4.71E-08 | 3.39E-09 | -0.91 | 2.65E-08 | 1.13E-09 | -1.17 |
| 213056_at | FRMD4B | 1.97E-18 | 1.94E-20 | -1.30 | 2.21E-11 | 7.62E-13 | -0.80 | 4.52E-07 | 2.82E-08 | -0.79 |
| 203603_s_at | ZEB2 | 9.58E-14 | 3.27E-15 | -1.30 | 1.82E-12 | 4.89E-14 | -1.21 | 7.52E-12 | 1.05E-13 | -1.20 |
| 202274_at | ACTG2 | 4.26E-08 | 4.74E-09 | -1.30 | 8.54E-06 | 1.05E-06 | -0.87 | 9.05E-06 | 8.54E-07 | -1.43 |
| 207761_s_at | METTL7A | 1.13E-15 | 2.43E-17 | -1.30 | 4.19E-15 | 6.33E-17 | -1.21 | 4.08E-05 | 4.73E-06 | -0.81 |
| 231181_at |  | 7.59E-11 | 4.79E-12 | -1.29 | 8.95E-12 | 2.83E-13 | -1.17 | 4.75E-09 | 1.64E-10 | -2.19 |
| 230958_s_at |  | 9.24E-11 | 5.93E-12 | -1.29 | 2.57E-05 | 3.57E-06 | -0.80 | 7.02E-07 | 4.67E-08 | -1.01 |
| 209710_at | GATA2 | 2.48E-15 | 5.87E-17 | -1.29 | 1.45E-16 | 1.55E-18 | -1.33 | 1.34E-16 | 4.03E-19 | -1.76 |
| 215322_at |  | 1.27E-17 | 1.60E-19 | -1.29 | 4.34E-05 | 6.36E-06 | -0.95 | 9.61E-13 | 9.58E-15 | -1.55 |
| 208707_at | EIF5 | 1.47E-15 | 3.28E-17 | -1.29 | 3.87E-12 | 1.12E-13 | -0.94 | 2.32E-20 | 1.54E-23 | -1.64 |
| 1559696_at |  | 9.73E-14 | 3.33E-15 | -1.29 | 1.48E-11 | 4.93E-13 | -0.93 | 8.68E-10 | 2.39E-11 | -3.15 |
| 1557729_at | GRK5 | 1.71E-16 | 3.01E-18 | -1.29 | 6.01E-32 | 7.70E-36 | -1.77 | 7.70E-22 | 2.39E-25 | -1.84 |
| 231842_at | KIAA1462 | 1.85E-13 | 6.67E-15 | -1.29 | 4.97E-18 | 3.65E-20 | -1.67 | 4.41E-13 | 4.06E-15 | -1.38 |
| 210423_s_at | SLC11A1 | 1.34E-11 | 7.30E-13 | -1.29 | 1.35E-09 | 6.97E-11 | -0.87 | 2.86E-08 | 1.23E-09 | -1.26 |
| 227399_at | VGLL3 | 8.88E-12 | 4.68E-13 | -1.29 | 2.93E-10 | 1.31E-11 | -1.43 | 2.07E-06 | 1.60E-07 | -1.23 |
| 1559605_a_at | LOC285043 | 5.66E-14 | 1.82E-15 | -1.29 | 1.68E-11 | 5.66E-13 | -1.03 | 8.83E-07 | 6.06E-08 | -1.68 |
| 210549_s_at | CCL23 | 4.00E-13 | 1.57E-14 | -1.29 | 5.79E-15 | 9.04E-17 | -1.31 | 9.16E-06 | 8.67E-07 | -1.72 |
| 208789_at | PTRF | 1.21E-15 | 2.65E-17 | -1.28 | 2.58E-22 | 6.03E-25 | -1.21 | 4.84E-19 | 6.02E-22 | -1.45 |
| 203662_s_at | TMOD1 | 1.38E-19 | 8.83E-22 | -1.28 | 1.58E-19 | 7.66E-22 | -1.05 | 6.21E-11 | 1.18E-12 | -1.73 |
| 219654_at | PTPLA | 7.38E-11 | 4.65E-12 | -1.28 | 1.03E-10 | 4.17E-12 | -1.32 | 1.50E-07 | 8.00E-09 | -1.29 |
| 228193_s_at | RGCC | 1.53E-15 | 3.44E-17 | -1.28 | 1.22E-12 | 3.17E-14 | -1.25 | 3.82E-07 | 2.32E-08 | -1.09 |
| 212071_s_at | SPTBN1 | 8.33E-21 | 3.47E-23 | -1.28 | 2.58E-20 | 1.02E-22 | -1.06 | 8.20E-15 | 4.41E-17 | -1.13 |
| 207808_s_at | PROS1 | 6.92E-16 | 1.41E-17 | -1.28 | 1.33E-11 | 4.37E-13 | -1.22 | 7.67E-07 | 5.17E-08 | -0.88 |
| 204622_x_at | NR4A2 | 1.24E-06 | 1.85E-07 | -1.28 | 5.81E-11 | 2.21E-12 | -1.87 | 1.47E-05 | 1.48E-06 | -1.69 |
| 209159_s_at | NDRG4 | 8.86E-13 | 3.76E-14 | -1.28 | 1.08E-12 | 2.76E-14 | -1.41 | 2.61E-09 | 8.35E-11 | -1.84 |
| 231804_at | RXFP1 | 7.68E-14 | 2.56E-15 | -1.28 | 7.94E-19 | 4.72E-21 | -1.69 | 2.91E-10 | 6.82E-12 | -2.11 |
| 202910_s_at | CD97 | 6.84E-19 | 5.67E-21 | -1.28 | 8.83E-14 | 1.77E-15 | -1.05 | 5.10E-06 | 4.45E-07 | -0.90 |
| 225166_at | ARHGAP18 | 7.25E-16 | 1.49E-17 | -1.28 | 7.92E-07 | 7.58E-08 | -0.71 | 8.72E-11 | 1.74E-12 | -0.79 |
| 218309_at | CAMK2N1 | 1.69E-10 | 1.15E-11 | -1.28 | 1.19E-11 | 3.85E-13 | -1.43 | 8.63E-07 | 5.91E-08 | -1.32 |
| 230305_at | LINC00400 | 1.80E-16 | 3.18E-18 | -1.28 | 2.03E-14 | 3.54E-16 | -1.05 | 5.37E-12 | 7.14E-14 | -1.04 |
| 202908_at | WFS1 | 8.25E-21 | 3.41E-23 | -1.27 | 4.26E-19 | 2.32E-21 | -1.09 | 3.82E-10 | 9.41E-12 | -0.91 |
| 240410_at |  | 1.26E-11 | 6.81E-13 | -1.27 | 1.20E-06 | 1.20E-07 | -1.00 | 1.33E-13 | 1.06E-15 | -1.83 |
| 201578_at | PODXL | 5.41E-17 | 8.03E-19 | -1.27 | 1.72E-16 | 1.85E-18 | -1.07 | 4.62E-18 | 8.29E-21 | -1.21 |
| 209355_s_at | PPAP2B | 4.84E-16 | 9.43E-18 | -1.27 | 5.68E-06 | 6.73E-07 | -1.34 | 1.11E-11 | 1.69E-13 | -1.36 |
| 235334_at | ST6GALNAC3 | 7.93E-13 | 3.34E-14 | -1.27 | 2.46E-16 | 2.79E-18 | -1.56 | 2.08E-16 | 6.86E-19 | -1.65 |
| 201325_s_at | EMP1 | 9.56E-09 | 9.34E-10 | -1.27 | 1.15E-06 | 1.15E-07 | -1.20 | 2.57E-07 | 1.48E-08 | -1.05 |
| 201432_at | CAT | 3.94E-22 | 8.73E-25 | -1.27 | 2.32E-09 | 1.26E-10 | -0.96 | 4.11E-07 | 2.53E-08 | -0.72 |
| 228372_at | C10orf128 | 8.02E-18 | 9.36E-20 | -1.27 | 1.29E-11 | 4.23E-13 | -0.92 | 7.45E-07 | 5.01E-08 | -0.81 |
| 208983_s_at | PECAM1 | 2.41E-11 | 1.38E-12 | -1.27 | 4.34E-15 | 6.59E-17 | -1.74 | 1.37E-16 | 4.12E-19 | -1.62 |
| 225544_at | TBX3 | 1.37E-14 | 3.85E-16 | -1.27 | 3.77E-16 | 4.46E-18 | -1.04 | 2.29E-18 | 3.73E-21 | -1.82 |
| 201809_s_at | ENG | 9.53E-17 | 1.51E-18 | -1.27 | 2.85E-11 | 1.01E-12 | -0.80 | 4.15E-13 | 3.79E-15 | -1.11 |
| 228045_at |  | 6.50E-13 | 2.68E-14 | -1.27 | 3.24E-07 | 2.81E-08 | -0.89 | 2.50E-09 | 7.95E-11 | -0.98 |
| 209335_at | DCN | 1.85E-11 | 1.03E-12 | -1.27 | 3.88E-07 | 3.43E-08 | -0.93 | 1.33E-06 | 9.67E-08 | -1.04 |
| 220979_s_at | ST6GALNAC5 | 1.90E-10 | 1.31E-11 | -1.27 | 1.61E-18 | 1.04E-20 | -2.23 | 3.68E-09 | 1.23E-10 | -1.59 |
| 202177_at | GAS6 | 9.94E-15 | 2.68E-16 | -1.26 | 2.17E-12 | 5.92E-14 | -0.97 | 2.37E-07 | 1.34E-08 | -0.90 |
| 242496_at |  | 1.62E-12 | 7.24E-14 | -1.26 | 2.07E-11 | 7.10E-13 | -1.22 | 5.68E-06 | 5.04E-07 | -1.94 |
| 212822_at | HEG1 | 4.30E-15 | 1.07E-16 | -1.26 | 2.00E-22 | 4.42E-25 | -1.44 | 1.27E-18 | 1.85E-21 | -1.55 |
| 231262_at |  | 8.71E-07 | 1.26E-07 | -1.26 | 1.28E-13 | 2.64E-15 | -1.76 | 3.51E-13 | 3.12E-15 | -2.04 |
| 223492_s_at | LRRFIP1 | 5.72E-15 | 1.47E-16 | -1.26 | 1.02E-18 | 6.32E-21 | -1.54 | 5.53E-16 | 2.20E-18 | -1.36 |
| 227126_at | PTPRG | 1.68E-13 | 6.03E-15 | -1.26 | 2.68E-07 | 2.28E-08 | -0.88 | 2.80E-10 | 6.52E-12 | -1.20 |
| 203962_s_at | NEBL | 1.52E-11 | 8.37E-13 | -1.26 | 5.54E-10 | 2.64E-11 | -1.43 | 4.04E-08 | 1.83E-09 | -1.19 |
| 220180_at | CCDC68 | 3.71E-10 | 2.73E-11 | -1.26 | 3.52E-10 | 1.60E-11 | -1.23 | 2.69E-07 | 1.56E-08 | -1.37 |
| 207857_at | LILRA2 | 2.05E-12 | 9.36E-14 | -1.25 | 6.14E-12 | 1.87E-13 | -1.13 | 1.14E-06 | 8.15E-08 | -1.12 |
| 212886_at | CCDC69 | 6.90E-19 | 5.73E-21 | -1.25 | 1.26E-11 | 4.11E-13 | -1.00 | 1.07E-07 | 5.50E-09 | -1.14 |
| 1559477_s_at | MEIS1 | 2.92E-14 | 8.88E-16 | -1.25 | 8.14E-13 | 2.02E-14 | -1.05 | 4.57E-07 | 2.86E-08 | -0.73 |
| 226955_at | AFAP1L1 | 1.17E-13 | 4.08E-15 | -1.25 | 6.56E-17 | 6.45E-19 | -1.59 | 5.53E-16 | 2.21E-18 | -1.68 |
| 206382_s_at | BDNF | 1.46E-10 | 9.78E-12 | -1.25 | 9.71E-22 | 2.75E-24 | -1.64 | 5.83E-11 | 1.10E-12 | -2.83 |
| 210548_at | CCL23 | 7.17E-12 | 3.70E-13 | -1.25 | 4.45E-13 | 1.05E-14 | -1.37 | 1.13E-05 | 1.10E-06 | -2.01 |
| 225450_at | AMOTL1 | 2.14E-18 | 2.15E-20 | -1.25 | 4.60E-17 | 4.36E-19 | -1.25 | 1.12E-07 | 5.79E-09 | -0.78 |
| 227458_at | CD274 | 9.42E-07 | 1.37E-07 | -1.25 | 2.82E-12 | 7.90E-14 | -1.57 | 8.39E-05 | 1.08E-05 | -0.86 |
| 41644_at | SASH1 | 2.12E-20 | 1.01E-22 | -1.25 | 1.39E-15 | 1.88E-17 | -1.20 | 1.78E-21 | 7.48E-25 | -1.71 |
| 203934_at | KDR | 2.17E-09 | 1.86E-10 | -1.25 | 4.34E-13 | 1.02E-14 | -1.35 | 3.52E-05 | 4.00E-06 | -0.97 |
| 202917_s_at | S100A8 | 4.95E-06 | 8.38E-07 | -1.25 | 6.79E-10 | 3.29E-11 | -2.04 | 1.67E-06 | 1.26E-07 | -1.92 |
| 202468_s_at | CTNNAL1 | 4.60E-16 | 8.88E-18 | -1.24 | 8.81E-16 | 1.15E-17 | -1.18 | 1.93E-14 | 1.20E-16 | -1.31 |
| 203058_s_at | PAPSS2 | 5.43E-13 | 2.21E-14 | -1.24 | 2.63E-14 | 4.72E-16 | -1.32 | 6.23E-08 | 2.96E-09 | -0.92 |
| 219656_at | PCDH12 | 2.69E-16 | 4.96E-18 | -1.24 | 1.03E-17 | 8.33E-20 | -0.92 | 2.45E-14 | 1.57E-16 | -1.39 |
| 209960_at | HGF | 4.46E-14 | 1.40E-15 | -1.24 | 4.88E-07 | 4.42E-08 | -0.91 | 2.13E-05 | 2.26E-06 | -1.07 |
| 231899_at | ZC3H12C | 8.59E-15 | 2.29E-16 | -1.24 | 1.82E-11 | 6.16E-13 | -1.12 | 1.65E-08 | 6.58E-10 | -0.88 |
| 230636_s_at | KLF9 | 4.62E-12 | 2.28E-13 | -1.24 | 1.19E-11 | 3.87E-13 | -1.09 | 3.63E-16 | 1.31E-18 | -1.32 |
| 220765_s_at | LIMS2 | 1.17E-17 | 1.46E-19 | -1.24 | 1.67E-20 | 6.20E-23 | -1.22 | 6.75E-18 | 1.30E-20 | -1.43 |
| 218805_at | GIMAP5 | 2.86E-15 | 6.84E-17 | -1.24 | 4.80E-19 | 2.64E-21 | -1.41 | 4.41E-10 | 1.11E-11 | -1.29 |
| 204365_s_at | REEP1 | 1.64E-12 | 7.36E-14 | -1.24 | 1.30E-13 | 2.69E-15 | -1.00 | 2.70E-08 | 1.15E-09 | -1.50 |
| 225604_s_at | GLIPR2 | 3.40E-10 | 2.47E-11 | -1.24 | 7.32E-12 | 2.27E-13 | -1.31 | 3.58E-13 | 3.21E-15 | -1.11 |
| 224352_s_at | CFL2 | 1.22E-16 | 2.04E-18 | -1.24 | 3.17E-18 | 2.19E-20 | -1.35 | 4.49E-18 | 7.97E-21 | -1.23 |
| 227307_at | TSPAN18 | 8.61E-17 | 1.35E-18 | -1.23 | 1.10E-21 | 3.18E-24 | -1.36 | 6.15E-12 | 8.29E-14 | -1.02 |
| 219892_at | TM6SF1 | 2.78E-13 | 1.05E-14 | -1.23 | 6.60E-06 | 7.94E-07 | -1.02 | 5.62E-05 | 6.78E-06 | -0.72 |
| 226402_at | CYP2U1 | 2.99E-17 | 4.16E-19 | -1.23 | 4.35E-06 | 5.00E-07 | -0.76 | 1.40E-07 | 7.38E-09 | -0.82 |
| 200904_at | HLA-E | 2.33E-18 | 2.36E-20 | -1.23 | 6.53E-17 | 6.41E-19 | -0.88 | 9.95E-08 | 5.05E-09 | -0.73 |
| 203895_at | PLCB4 | 6.82E-09 | 6.51E-10 | -1.23 | 9.37E-10 | 4.68E-11 | -1.50 | 1.67E-06 | 1.25E-07 | -1.62 |
| 210873_x_at | APOBEC3A | 2.75E-06 | 4.40E-07 | -1.23 | 1.03E-07 | 8.06E-09 | -1.47 | 4.34E-07 | 2.69E-08 | -1.49 |
| 208228_s_at | FGFR2 | 6.34E-10 | 4.88E-11 | -1.23 | 1.20E-06 | 1.20E-07 | -0.97 | 1.25E-05 | 1.23E-06 | -1.14 |
| 226864_at | PKIA | 1.67E-15 | 3.80E-17 | -1.23 | 1.12E-08 | 7.09E-10 | -0.97 | 1.62E-06 | 1.21E-07 | -1.45 |
| 216248_s_at | NR4A2 | 5.91E-06 | 1.02E-06 | -1.23 | 1.58E-10 | 6.65E-12 | -1.93 | 1.82E-05 | 1.88E-06 | -1.78 |
| 223374_s_at | B3GALNT1 | 1.24E-10 | 8.15E-12 | -1.23 | 9.44E-10 | 4.72E-11 | -1.52 | 5.68E-11 | 1.06E-12 | -1.20 |
| 217504_at | ABCA6 | 6.60E-14 | 2.16E-15 | -1.22 | 3.18E-18 | 2.21E-20 | -1.54 | 2.25E-07 | 1.26E-08 | -1.16 |
| 221861_at | ARRB1 | 7.88E-16 | 1.64E-17 | -1.22 | 6.30E-27 | 3.92E-30 | -1.21 | 6.17E-17 | 1.68E-19 | -1.72 |
| 235978_at | FABP4 | 7.07E-14 | 2.33E-15 | -1.22 | 4.81E-19 | 2.66E-21 | -1.34 | 7.73E-12 | 1.09E-13 | -2.88 |
| 202555_s_at | MYLK | 3.32E-15 | 8.08E-17 | -1.22 | 2.83E-06 | 3.08E-07 | -0.84 | 6.97E-06 | 6.40E-07 | -0.96 |
| 222312_s_at |  | 2.10E-13 | 7.70E-15 | -1.22 | 7.59E-09 | 4.63E-10 | -0.75 | 1.93E-05 | 2.02E-06 | -1.02 |
| 229427_at | SEMA5A | 4.69E-13 | 1.88E-14 | -1.22 | 1.03E-19 | 4.75E-22 | -1.47 | 3.46E-13 | 3.05E-15 | -1.37 |
| 201324_at | EMP1 | 1.66E-10 | 1.13E-11 | -1.22 | 5.72E-08 | 4.21E-09 | -0.93 | 3.67E-07 | 2.22E-08 | -0.82 |
| 209305_s_at | GADD45B | 3.68E-07 | 4.95E-08 | -1.22 | 1.93E-10 | 8.35E-12 | -1.41 | 6.97E-06 | 6.39E-07 | -1.18 |
| 225776_at | RBMS2 | 3.74E-14 | 1.16E-15 | -1.21 | 5.96E-15 | 9.33E-17 | -1.09 | 3.53E-11 | 6.27E-13 | -1.06 |
| 227771_at | LIFR | 2.40E-09 | 2.08E-10 | -1.21 | 3.96E-10 | 1.82E-11 | -1.21 | 3.84E-10 | 9.49E-12 | -1.40 |
| 202552_s_at | CRIM1 | 2.08E-16 | 3.74E-18 | -1.21 | 9.91E-15 | 1.61E-16 | -1.09 | 8.74E-11 | 1.74E-12 | -1.24 |
| 1553102_a_at | CCDC69 | 9.11E-18 | 1.08E-19 | -1.21 | 7.79E-12 | 2.44E-13 | -1.14 | 3.97E-07 | 2.43E-08 | -1.22 |
| 224341_x_at | TLR4 | 5.11E-15 | 1.30E-16 | -1.21 | 2.03E-09 | 1.09E-10 | -1.27 | 1.22E-08 | 4.68E-10 | -1.11 |
| 235494_at | LSAMP | 1.78E-19 | 1.18E-21 | -1.21 | 1.72E-09 | 9.13E-11 | -0.92 | 4.55E-06 | 3.90E-07 | -1.39 |
| 222912_at | ARRB1 | 3.18E-10 | 2.30E-11 | -1.21 | 2.34E-14 | 4.15E-16 | -1.12 | 1.91E-06 | 1.46E-07 | -0.86 |
| 1552316_a_at | GIMAP1 | 1.39E-13 | 4.90E-15 | -1.21 | 5.39E-13 | 1.29E-14 | -1.34 | 4.34E-08 | 1.98E-09 | -1.04 |
| 202551_s_at | CRIM1 | 3.68E-14 | 1.15E-15 | -1.21 | 1.13E-15 | 1.49E-17 | -1.21 | 4.47E-12 | 5.76E-14 | -1.08 |
| 204466_s_at | SNCA | 1.90E-13 | 6.90E-15 | -1.21 | 7.09E-11 | 2.76E-12 | -1.19 | 6.32E-09 | 2.25E-10 | -1.25 |
| 214438_at | HLX | 2.15E-16 | 3.88E-18 | -1.21 | 1.82E-12 | 4.89E-14 | -0.90 | 6.73E-10 | 1.79E-11 | -0.93 |
| 212494_at | TENC1 | 1.28E-14 | 3.57E-16 | -1.21 | 1.14E-10 | 4.65E-12 | -0.84 | 8.84E-12 | 1.28E-13 | -1.36 |
| 225894_at | SYNPO2 | 2.17E-15 | 5.07E-17 | -1.21 | 2.27E-09 | 1.24E-10 | -1.15 | 5.16E-07 | 3.30E-08 | -1.31 |
| 221846_s_at | CASKIN2 | 8.26E-16 | 1.72E-17 | -1.20 | 1.58E-19 | 7.64E-22 | -1.08 | 2.78E-09 | 8.95E-11 | -1.23 |
| 204442_x_at | LTBP4 | 5.37E-14 | 1.71E-15 | -1.20 | 8.98E-19 | 5.40E-21 | -1.43 | 3.23E-15 | 1.56E-17 | -1.77 |
| 206115_at | EGR3 | 4.19E-07 | 5.70E-08 | -1.20 | 5.24E-09 | 3.08E-10 | -1.56 | 4.62E-06 | 3.97E-07 | -1.50 |
| 207195_at | CNTN6 | 7.85E-19 | 6.68E-21 | -1.20 | 8.06E-21 | 2.83E-23 | -1.35 | 9.09E-06 | 8.59E-07 | -1.71 |
| 219134_at | ELTD1 | 7.18E-12 | 3.71E-13 | -1.20 | 3.49E-13 | 8.01E-15 | -1.30 | 1.62E-10 | 3.49E-12 | -1.36 |
| 228302_x_at | CAMK2N1 | 1.42E-07 | 1.75E-08 | -1.20 | 1.33E-09 | 6.86E-11 | -1.18 | 3.01E-07 | 1.78E-08 | -2.20 |
| 227401_at | IL17D | 4.53E-15 | 1.14E-16 | -1.20 | 3.87E-14 | 7.18E-16 | -1.03 | 4.28E-09 | 1.46E-10 | -1.24 |
| 233903_s_at | ARHGEF26 | 3.76E-11 | 2.24E-12 | -1.20 | 2.25E-24 | 2.92E-27 | -2.22 | 2.03E-20 | 1.30E-23 | -2.01 |
| 227108_at | STARD9 | 6.80E-17 | 1.03E-18 | -1.20 | 9.58E-15 | 1.56E-16 | -0.98 | 5.12E-15 | 2.62E-17 | -1.24 |
| 202746_at | ITM2A | 3.69E-11 | 2.19E-12 | -1.20 | 1.33E-15 | 1.78E-17 | -1.78 | 5.79E-12 | 7.76E-14 | -1.63 |
| 225372_at | C10orf54 | 1.89E-21 | 5.71E-24 | -1.19 | 8.36E-23 | 1.67E-25 | -1.18 | 2.55E-14 | 1.65E-16 | -1.45 |
| 229319_at |  | 8.78E-19 | 7.63E-21 | -1.19 | 8.95E-07 | 8.67E-08 | -0.73 | 6.80E-07 | 4.49E-08 | -0.90 |
| 208740_at | SAP18 | 2.92E-21 | 9.82E-24 | -1.19 | 4.30E-12 | 1.26E-13 | -0.87 | 1.64E-08 | 6.54E-10 | -0.78 |
| 49111_at | ARRB1 | 8.14E-15 | 2.16E-16 | -1.19 | 6.95E-27 | 4.57E-30 | -1.32 | 1.35E-13 | 1.08E-15 | -1.77 |
| 1552701_a_at | CARD16 | 2.56E-14 | 7.64E-16 | -1.19 | 6.64E-13 | 1.61E-14 | -1.01 | 1.59E-05 | 1.62E-06 | -0.72 |
| 204621_s_at | NR4A2 | 2.40E-06 | 3.80E-07 | -1.19 | 5.55E-11 | 2.10E-12 | -1.76 | 7.88E-06 | 7.33E-07 | -1.80 |
| 218574_s_at | LMCD1 | 2.65E-14 | 7.99E-16 | -1.19 | 4.14E-14 | 7.76E-16 | -1.06 | 1.03E-07 | 5.24E-09 | -1.03 |
| 242332_at | FENDRR | 2.84E-15 | 6.79E-17 | -1.19 | 1.70E-20 | 6.37E-23 | -1.16 | 2.39E-19 | 2.54E-22 | -2.16 |
| 214761_at | ZNF423 | 2.39E-10 | 1.68E-11 | -1.19 | 2.22E-09 | 1.21E-10 | -1.24 | 1.31E-06 | 9.51E-08 | -1.35 |
| 212226_s_at | PPAP2B | 7.58E-19 | 6.42E-21 | -1.19 | 6.45E-06 | 7.75E-07 | -1.07 | 2.75E-11 | 4.74E-13 | -1.10 |
| 217781_s_at | ZNF106 | 7.34E-24 | 7.25E-27 | -1.18 | 1.71E-17 | 1.46E-19 | -0.97 | 1.32E-09 | 3.85E-11 | -0.80 |
| 203951_at | CNN1 | 8.17E-11 | 5.19E-12 | -1.18 | 5.93E-09 | 3.54E-10 | -0.90 | 2.52E-06 | 2.00E-07 | -1.37 |
| 36711_at | MAFF | 5.29E-08 | 6.01E-09 | -1.18 | 3.11E-14 | 5.64E-16 | -1.62 | 2.49E-08 | 1.05E-09 | -1.10 |
| 225144_at | BMPR2 | 2.75E-17 | 3.80E-19 | -1.18 | 1.85E-10 | 7.96E-12 | -1.10 | 5.26E-17 | 1.38E-19 | -0.86 |
| 238512_at |  | 7.65E-14 | 2.54E-15 | -1.17 | 5.74E-12 | 1.74E-13 | -1.06 | 4.89E-15 | 2.48E-17 | -1.70 |
| 236401_at | GIMAP1 | 6.47E-16 | 1.31E-17 | -1.17 | 3.02E-16 | 3.47E-18 | -1.28 | 3.77E-10 | 9.26E-12 | -1.19 |
| 1556209_at | CLEC2B | 1.81E-10 | 1.24E-11 | -1.17 | 2.54E-08 | 1.73E-09 | -1.10 | 1.49E-05 | 1.50E-06 | -1.05 |
| 214318_s_at | FRY | 1.00E-12 | 4.31E-14 | -1.17 | 1.30E-09 | 6.72E-11 | -1.23 | 6.55E-06 | 5.96E-07 | -0.98 |
| 208961_s_at | KLF6 | 8.90E-13 | 3.77E-14 | -1.17 | 4.08E-19 | 2.20E-21 | -1.49 | 1.26E-07 | 6.57E-09 | -0.98 |
| 225688_s_at | PHLDB2 | 2.08E-09 | 1.77E-10 | -1.17 | 9.66E-13 | 2.45E-14 | -1.50 | 2.94E-09 | 9.52E-11 | -1.11 |
| 239183_at | ANGPTL1 | 3.71E-16 | 7.04E-18 | -1.17 | 1.64E-18 | 1.06E-20 | -1.60 | 5.73E-11 | 1.07E-12 | -2.70 |
| 204848_x_at | HBG2///HBG1 | 3.24E-05 | 6.55E-06 | -1.17 | 5.38E-12 | 1.62E-13 | -1.60 | 3.85E-08 | 1.73E-09 | -2.32 |
| 235210_s_at | SBSPON | 9.08E-12 | 4.80E-13 | -1.17 | 4.33E-09 | 2.50E-10 | -1.41 | 1.11E-08 | 4.22E-10 | -1.47 |
| 204419_x_at | HBG2///HBG1 | 3.05E-05 | 6.13E-06 | -1.17 | 3.46E-12 | 9.92E-14 | -1.72 | 2.66E-08 | 1.13E-09 | -2.02 |
| 231873_at | BMPR2 | 4.63E-10 | 3.47E-11 | -1.17 | 1.35E-08 | 8.69E-10 | -0.93 | 9.09E-20 | 8.48E-23 | -1.05 |
| 227727_at | MRGPRF | 8.77E-12 | 4.62E-13 | -1.17 | 5.25E-08 | 3.82E-09 | -0.87 | 3.35E-07 | 2.00E-08 | -1.15 |
| 204726_at | CDH13 | 1.47E-13 | 5.18E-15 | -1.16 | 9.93E-12 | 3.18E-13 | -1.00 | 3.15E-10 | 7.52E-12 | -1.81 |
| 226280_at | BNIP2 | 1.86E-18 | 1.82E-20 | -1.16 | 3.96E-16 | 4.72E-18 | -1.28 | 5.02E-16 | 1.90E-18 | -1.07 |
| 225162_at | SH3D19 | 2.03E-16 | 3.64E-18 | -1.16 | 5.58E-08 | 4.10E-09 | -0.90 | 2.16E-09 | 6.74E-11 | -0.87 |
| 218546_at | C1orf115 | 7.27E-13 | 3.03E-14 | -1.16 | 4.78E-15 | 7.39E-17 | -1.25 | 7.89E-12 | 1.11E-13 | -1.43 |
| 226614_s_at | FAM167A | 5.41E-09 | 5.06E-10 | -1.15 | 1.14E-19 | 5.30E-22 | -1.34 | 5.86E-07 | 3.80E-08 | -1.35 |
| 207072_at | IL18RAP | 1.70E-11 | 9.44E-13 | -1.15 | 1.63E-10 | 6.88E-12 | -1.14 | 4.75E-07 | 3.00E-08 | -1.16 |
| 205730_s_at | ABLIM3 | 9.31E-15 | 2.50E-16 | -1.15 | 2.77E-19 | 1.44E-21 | -1.12 | 6.87E-10 | 1.84E-11 | -1.50 |
| 215306_at |  | 3.30E-13 | 1.27E-14 | -1.15 | 1.26E-10 | 5.20E-12 | -1.07 | 6.19E-08 | 2.94E-09 | -1.30 |
| 203591_s_at | CSF3R | 2.85E-10 | 2.04E-11 | -1.15 | 4.73E-12 | 1.40E-13 | -1.25 | 7.00E-05 | 8.74E-06 | -0.95 |
| 209539_at | ARHGEF6 | 7.31E-17 | 1.13E-18 | -1.15 | 2.74E-14 | 4.94E-16 | -0.91 | 5.13E-08 | 2.38E-09 | -0.86 |
| 203143_s_at | KIAA0040 | 3.17E-19 | 2.33E-21 | -1.15 | 6.02E-14 | 1.17E-15 | -1.03 | 1.74E-15 | 7.88E-18 | -1.22 |
| 203718_at | PNPLA6 | 1.34E-13 | 4.72E-15 | -1.15 | 3.57E-07 | 3.13E-08 | -0.77 | 2.74E-11 | 4.72E-13 | -0.79 |
| 206027_at | S100A3 | 9.35E-11 | 6.01E-12 | -1.15 | 5.72E-18 | 4.28E-20 | -1.57 | 1.51E-09 | 4.48E-11 | -2.04 |
| 207002_s_at | PLAGL1 | 8.19E-14 | 2.75E-15 | -1.14 | 7.79E-09 | 4.76E-10 | -0.93 | 9.00E-09 | 3.32E-10 | -1.17 |
| 236123_at | ST7L | 1.12E-14 | 3.05E-16 | -1.14 | 9.16E-12 | 2.90E-13 | -0.78 | 6.25E-17 | 1.72E-19 | -1.38 |
| 210139_s_at | PMP22 | 2.02E-15 | 4.67E-17 | -1.14 | 1.43E-12 | 3.74E-14 | -1.02 | 7.31E-07 | 4.91E-08 | -0.71 |
| 219195_at | PPARGC1A | 4.64E-09 | 4.29E-10 | -1.14 | 3.36E-15 | 4.98E-17 | -1.24 | 2.30E-08 | 9.61E-10 | -2.01 |
| 1559697_a_at |  | 1.11E-10 | 7.23E-12 | -1.14 | 1.44E-08 | 9.27E-10 | -0.83 | 1.47E-13 | 1.19E-15 | -2.63 |
| 201058_s_at | MYL9 | 3.86E-10 | 2.84E-11 | -1.14 | 1.94E-11 | 6.64E-13 | -1.24 | 2.46E-08 | 1.03E-09 | -1.24 |
| 221886_at | DENND2A | 1.36E-15 | 3.02E-17 | -1.14 | 2.29E-21 | 7.11E-24 | -1.23 | 7.66E-18 | 1.53E-20 | -2.07 |
| 228724_at | TTLL7 | 9.95E-10 | 7.98E-11 | -1.14 | 1.49E-13 | 3.11E-15 | -1.09 | 5.48E-14 | 3.92E-16 | -2.38 |
| 209543_s_at | CD34 | 7.68E-14 | 2.56E-15 | -1.14 | 9.76E-12 | 3.11E-13 | -0.96 | 6.14E-12 | 8.27E-14 | -1.19 |
| 215617_at | SPATS2L | 9.22E-14 | 3.13E-15 | -1.14 | 1.12E-10 | 4.59E-12 | -0.95 | 2.31E-07 | 1.30E-08 | -0.98 |
| 218346_s_at | SESN1 | 2.76E-14 | 8.34E-16 | -1.14 | 2.69E-08 | 1.84E-09 | -0.84 | 5.50E-08 | 2.58E-09 | -0.92 |
| 203329_at | PTPRM | 3.35E-16 | 6.30E-18 | -1.14 | 6.75E-21 | 2.27E-23 | -0.94 | 9.33E-12 | 1.37E-13 | -1.22 |
| 227688_at | LRCH2 | 2.49E-13 | 9.30E-15 | -1.14 | 2.18E-07 | 1.82E-08 | -1.10 | 4.57E-05 | 5.38E-06 | -1.11 |
| 235642_at |  | 2.15E-17 | 2.89E-19 | -1.14 | 6.16E-22 | 1.63E-24 | -1.48 | 1.41E-11 | 2.24E-13 | -3.08 |
| 231411_at | LHFP | 4.90E-12 | 2.44E-13 | -1.14 | 1.64E-10 | 6.95E-12 | -0.92 | 9.01E-11 | 1.80E-12 | -0.79 |
| 209655_s_at | TMEM47 | 5.95E-12 | 3.01E-13 | -1.13 | 1.72E-12 | 4.58E-14 | -1.35 | 9.34E-10 | 2.59E-11 | -1.44 |
| 238893_at | LINC00936 | 1.35E-15 | 2.99E-17 | -1.13 | 1.01E-07 | 7.87E-09 | -0.76 | 1.05E-10 | 2.16E-12 | -1.07 |
| 206453_s_at | NDRG2 | 6.03E-12 | 3.06E-13 | -1.13 | 7.52E-18 | 5.86E-20 | -1.07 | 1.59E-09 | 4.73E-11 | -1.20 |
| 1555270_a_at | WFS1 | 4.48E-17 | 6.48E-19 | -1.13 | 2.23E-18 | 1.47E-20 | -1.14 | 1.07E-06 | 7.50E-08 | -0.78 |
| 209318_x_at | PLAGL1 | 1.60E-12 | 7.13E-14 | -1.13 | 9.67E-09 | 6.02E-10 | -0.96 | 1.68E-07 | 9.05E-09 | -1.00 |
| 204220_at | GMFG | 1.63E-16 | 2.84E-18 | -1.13 | 4.05E-15 | 6.09E-17 | -1.15 | 3.23E-08 | 1.42E-09 | -0.84 |
| 226905_at | FAM101B | 4.50E-13 | 1.80E-14 | -1.13 | 4.32E-10 | 2.00E-11 | -1.06 | 2.67E-11 | 4.55E-13 | -1.16 |
| 242836_at |  | 2.36E-07 | 3.05E-08 | -1.13 | 3.72E-06 | 4.19E-07 | -1.48 | 3.89E-11 | 6.96E-13 | -2.41 |
| 232181_at | PPARGC1B | 1.04E-17 | 1.28E-19 | -1.13 | 2.48E-11 | 8.71E-13 | -1.16 | 7.01E-11 | 1.35E-12 | -1.36 |
| 203185_at | RASSF2 | 3.17E-12 | 1.51E-13 | -1.13 | 1.29E-14 | 2.17E-16 | -1.17 | 4.39E-11 | 7.93E-13 | -1.12 |
| 227646_at | EBF1 | 5.52E-12 | 2.78E-13 | -1.13 | 1.12E-10 | 4.60E-12 | -1.24 | 1.65E-10 | 3.57E-12 | -1.61 |
| 239848_at |  | 5.97E-16 | 1.19E-17 | -1.13 | 7.03E-11 | 2.73E-12 | -0.78 | 1.91E-11 | 3.13E-13 | -1.29 |
| 204049_s_at | PHACTR2 | 3.96E-16 | 7.55E-18 | -1.13 | 2.94E-08 | 2.03E-09 | -0.96 | 4.90E-11 | 8.99E-13 | -0.92 |
| 210838_s_at | ACVRL1 | 1.19E-13 | 4.12E-15 | -1.12 | 2.07E-24 | 2.62E-27 | -1.13 | 1.02E-06 | 7.17E-08 | -1.14 |
| 218678_at | NES | 6.22E-12 | 3.17E-13 | -1.12 | 4.03E-16 | 4.81E-18 | -1.23 | 3.61E-20 | 2.64E-23 | -1.70 |
| 221935_s_at | EOGT | 4.83E-14 | 1.53E-15 | -1.12 | 8.73E-10 | 4.33E-11 | -1.08 | 1.37E-14 | 8.09E-17 | -1.16 |
| 215146_s_at | TTC28 | 1.93E-15 | 4.45E-17 | -1.12 | 3.49E-13 | 8.01E-15 | -1.07 | 1.19E-13 | 9.38E-16 | -1.11 |
| 219407_s_at | LAMC3 | 4.53E-12 | 2.23E-13 | -1.12 | 9.37E-17 | 9.54E-19 | -0.92 | 1.22E-13 | 9.62E-16 | -1.65 |
| 225540_at | MAP2 | 1.76E-05 | 3.35E-06 | -1.12 | 5.88E-11 | 2.24E-12 | -1.33 | 4.13E-05 | 4.80E-06 | -1.17 |
| 236088_at | NTNG1 | 1.05E-13 | 3.59E-15 | -1.12 | 1.28E-22 | 2.64E-25 | -1.38 | 2.49E-09 | 7.90E-11 | -2.28 |
| 228370_at | LOC100506948 | 4.42E-13 | 1.76E-14 | -1.12 | 7.77E-09 | 4.74E-10 | -1.00 | 4.47E-06 | 3.83E-07 | -1.00 |
| 64064_at | GIMAP5 | 1.53E-16 | 2.64E-18 | -1.12 | 2.27E-20 | 8.85E-23 | -1.20 | 5.63E-10 | 1.46E-11 | -1.16 |
| 1552703_s_at | CARD16///CASP1 | 3.91E-12 | 1.90E-13 | -1.12 | 2.55E-11 | 8.98E-13 | -0.97 | 1.12E-07 | 5.78E-09 | -0.82 |
| 229969_at | SEC63 | 4.04E-18 | 4.33E-20 | -1.12 | 2.11E-07 | 1.76E-08 | -0.74 | 1.44E-07 | 7.67E-09 | -0.78 |
| 1552388_at | FLJ30901 | 5.56E-18 | 6.20E-20 | -1.11 | 2.93E-24 | 4.12E-27 | -0.92 | 4.42E-15 | 2.23E-17 | -3.35 |
| 220024_s_at | PRX | 3.18E-15 | 7.68E-17 | -1.11 | 1.74E-16 | 1.88E-18 | -0.96 | 5.61E-15 | 2.91E-17 | -1.64 |
| 213071_at | DPT | 1.98E-06 | 3.08E-07 | -1.11 | 1.63E-13 | 3.43E-15 | -1.55 | 3.29E-05 | 3.69E-06 | -1.45 |
| 218950_at | ARAP3 | 2.57E-14 | 7.71E-16 | -1.11 | 5.91E-19 | 3.42E-21 | -1.11 | 3.19E-17 | 7.83E-20 | -1.20 |
| 220110_s_at | NXF3 | 1.86E-11 | 1.04E-12 | -1.11 | 5.65E-10 | 2.70E-11 | -1.04 | 4.12E-10 | 1.03E-11 | -2.96 |
| 205472_s_at | DACH1 | 1.18E-09 | 9.60E-11 | -1.11 | 5.28E-11 | 1.99E-12 | -1.05 | 5.08E-14 | 3.55E-16 | -1.99 |
| 228603_at | ACTR3 | 5.51E-19 | 4.38E-21 | -1.11 | 1.99E-13 | 4.29E-15 | -1.04 | 7.43E-12 | 1.03E-13 | -0.79 |
| 213620_s_at | ICAM2 | 9.01E-16 | 1.90E-17 | -1.11 | 5.31E-20 | 2.27E-22 | -1.39 | 3.25E-13 | 2.85E-15 | -1.35 |
| 204681_s_at | RAPGEF5 | 3.68E-12 | 1.77E-13 | -1.11 | 2.17E-13 | 4.73E-15 | -0.92 | 9.32E-09 | 3.46E-10 | -0.91 |
| 228255_at | TMEM237 | 2.01E-13 | 7.34E-15 | -1.11 | 2.79E-09 | 1.55E-10 | -0.98 | 2.30E-07 | 1.29E-08 | -0.99 |
| 212813_at | JAM3 | 7.50E-13 | 3.14E-14 | -1.11 | 2.30E-11 | 7.99E-13 | -1.04 | 6.87E-10 | 1.84E-11 | -0.95 |
| 225163_at | FRMD4A | 5.42E-17 | 8.05E-19 | -1.10 | 5.69E-11 | 2.16E-12 | -0.99 | 6.81E-14 | 4.96E-16 | -1.19 |
| 1555832_s_at | KLF6 | 1.03E-13 | 3.54E-15 | -1.10 | 1.97E-18 | 1.30E-20 | -1.39 | 1.87E-06 | 1.43E-07 | -0.78 |
| 228062_at | NAP1L5 | 3.59E-12 | 1.72E-13 | -1.10 | 1.43E-07 | 1.15E-08 | -1.11 | 3.53E-09 | 1.18E-10 | -1.08 |
| 224606_at | KLF6 | 1.34E-15 | 2.95E-17 | -1.10 | 1.33E-16 | 1.40E-18 | -1.23 | 2.69E-06 | 2.15E-07 | -0.77 |
| 204445_s_at | ALOX5 | 1.89E-09 | 1.61E-10 | -1.10 | 1.32E-05 | 1.70E-06 | -0.77 | 2.39E-06 | 1.89E-07 | -1.00 |
| 201719_s_at | EPB41L2 | 1.29E-16 | 2.17E-18 | -1.10 | 2.62E-12 | 7.25E-14 | -1.01 | 1.46E-09 | 4.30E-11 | -1.04 |
| 225474_at | MAGI1 | 7.94E-14 | 2.65E-15 | -1.10 | 1.06E-07 | 8.28E-09 | -0.73 | 4.84E-06 | 4.19E-07 | -0.87 |
| 1557938_s_at | PTRF | 8.17E-10 | 6.43E-11 | -1.10 | 1.65E-10 | 6.97E-12 | -1.32 | 5.19E-16 | 2.01E-18 | -1.06 |
| 206380_s_at | CFP | 1.13E-16 | 1.86E-18 | -1.10 | 4.27E-16 | 5.15E-18 | -1.29 | 6.08E-09 | 2.15E-10 | -2.33 |
| 228042_at | ADPRH | 1.70E-21 | 5.07E-24 | -1.10 | 5.36E-13 | 1.28E-14 | -0.96 | 2.10E-13 | 1.77E-15 | -1.07 |
| 217721_at | SEPT7 | 2.43E-17 | 3.33E-19 | -1.10 | 6.68E-14 | 1.30E-15 | -0.86 | 2.78E-09 | 8.97E-11 | -0.89 |
| 230083_at | USP53 | 5.63E-12 | 2.84E-13 | -1.10 | 1.78E-09 | 9.46E-11 | -0.93 | 1.08E-07 | 5.51E-09 | -0.80 |
| 209883_at | COLGALT2 | 5.52E-15 | 1.41E-16 | -1.10 | 2.48E-10 | 1.09E-11 | -0.82 | 1.02E-09 | 2.87E-11 | -1.54 |
| 204793_at | GPRASP1 | 2.72E-13 | 1.03E-14 | -1.10 | 1.46E-06 | 1.49E-07 | -0.98 | 3.85E-05 | 4.43E-06 | -0.81 |
| 227856_at | C4orf32 | 1.83E-14 | 5.29E-16 | -1.10 | 1.01E-07 | 7.87E-09 | -0.99 | 2.25E-07 | 1.26E-08 | -0.93 |
| 230479_at |  | 3.17E-14 | 9.75E-16 | -1.10 | 3.29E-10 | 1.48E-11 | -0.88 | 2.32E-09 | 7.27E-11 | -0.86 |
| 212764_at | ZEB1 | 1.56E-13 | 5.56E-15 | -1.10 | 7.72E-08 | 5.85E-09 | -0.95 | 9.34E-06 | 8.84E-07 | -0.71 |
| 236656_s_at | LOC100288911 | 2.81E-10 | 2.01E-11 | -1.09 | 1.52E-08 | 9.84E-10 | -0.84 | 4.22E-06 | 3.59E-07 | -0.88 |
| 235173_at | MBNL1-AS1 | 3.94E-22 | 8.65E-25 | -1.09 | 4.56E-16 | 5.56E-18 | -0.77 | 4.10E-06 | 3.47E-07 | -0.84 |
| 232068_s_at | TLR4 | 6.39E-13 | 2.63E-14 | -1.09 | 2.71E-10 | 1.20E-11 | -1.38 | 2.22E-10 | 4.99E-12 | -1.29 |
| 228890_at | ATOH8 | 1.03E-07 | 1.24E-08 | -1.09 | 1.24E-14 | 2.06E-16 | -1.54 | 3.01E-09 | 9.80E-11 | -1.99 |
| 213675_at | PARVA | 2.16E-14 | 6.37E-16 | -1.09 | 6.02E-10 | 2.89E-11 | -0.80 | 5.34E-09 | 1.86E-10 | -0.78 |
| 205933_at | SETBP1 | 1.45E-12 | 6.42E-14 | -1.09 | 5.92E-09 | 3.53E-10 | -0.93 | 8.89E-06 | 8.37E-07 | -1.00 |
| 228399_at | OSR1 | 2.01E-08 | 2.10E-09 | -1.09 | 3.22E-09 | 1.82E-10 | -1.08 | 6.85E-05 | 8.53E-06 | -1.10 |
| 242641_at |  | 5.59E-14 | 1.79E-15 | -1.09 | 4.80E-16 | 5.91E-18 | -1.25 | 8.68E-09 | 3.19E-10 | -2.18 |
| 225093_at | UTRN | 1.20E-19 | 7.60E-22 | -1.09 | 1.75E-15 | 2.42E-17 | -0.84 | 5.28E-10 | 1.36E-11 | -0.74 |
| 235593_at | ZEB2 | 2.02E-13 | 7.39E-15 | -1.09 | 9.72E-10 | 4.88E-11 | -1.01 | 1.34E-07 | 7.05E-09 | -0.86 |
| 235421_at | MAP3K8 | 4.31E-11 | 2.60E-12 | -1.09 | 7.66E-05 | 1.19E-05 | -0.87 | 6.84E-05 | 8.52E-06 | -0.83 |
| 219821_s_at | GFOD1 | 2.60E-13 | 9.79E-15 | -1.08 | 4.04E-14 | 7.54E-16 | -1.15 | 1.69E-18 | 2.57E-21 | -1.34 |
| 214724_at | DIXDC1 | 3.00E-13 | 1.15E-14 | -1.08 | 2.20E-07 | 1.84E-08 | -1.03 | 2.00E-09 | 6.16E-11 | -0.97 |
| 200672_x_at | SPTBN1 | 2.07E-07 | 2.65E-08 | -1.08 | 1.30E-08 | 8.31E-10 | -1.39 | 2.81E-16 | 9.77E-19 | -1.28 |
| 227478_at | SETBP1 | 1.83E-12 | 8.25E-14 | -1.08 | 1.56E-08 | 1.02E-09 | -1.03 | 2.69E-05 | 2.94E-06 | -0.95 |
| 234066_at | IL1RL1 | 5.63E-08 | 6.45E-09 | -1.08 | 8.71E-11 | 3.47E-12 | -1.33 | 3.92E-07 | 2.40E-08 | -1.37 |
| 212486_s_at | FYN | 7.00E-15 | 1.83E-16 | -1.08 | 1.51E-16 | 1.62E-18 | -1.46 | 1.78E-12 | 1.93E-14 | -1.24 |
| 238891_at |  | 1.22E-16 | 2.03E-18 | -1.08 | 5.40E-20 | 2.34E-22 | -1.56 | 6.56E-10 | 1.74E-11 | -2.30 |
| 203910_at | ARHGAP29 | 3.60E-13 | 1.40E-14 | -1.08 | 3.56E-16 | 4.17E-18 | -1.31 | 7.61E-09 | 2.75E-10 | -0.92 |
| 222725_s_at | PALMD | 1.89E-11 | 1.06E-12 | -1.08 | 7.40E-09 | 4.50E-10 | -1.17 | 1.83E-07 | 9.99E-09 | -1.59 |
| 213228_at | PDE8B | 7.45E-08 | 8.75E-09 | -1.08 | 2.65E-09 | 1.47E-10 | -1.10 | 8.44E-09 | 3.09E-10 | -1.34 |
| 205529_s_at | RUNX1T1 | 3.58E-09 | 3.24E-10 | -1.08 | 2.65E-06 | 2.87E-07 | -0.89 | 8.20E-07 | 5.59E-08 | -1.18 |
| 225299_at | MYO5B | 2.07E-09 | 1.77E-10 | -1.08 | 2.75E-07 | 2.35E-08 | -1.00 | 2.00E-05 | 2.11E-06 | -0.92 |
| 204467_s_at | SNCA | 4.83E-17 | 7.05E-19 | -1.07 | 5.73E-19 | 3.28E-21 | -0.92 | 4.10E-14 | 2.79E-16 | -1.72 |
| 224862_at | GNAQ | 1.02E-19 | 6.26E-22 | -1.07 | 6.70E-15 | 1.06E-16 | -0.90 | 7.13E-12 | 9.86E-14 | -0.79 |
| 230288_at | FGF14 | 5.95E-10 | 4.55E-11 | -1.07 | 8.91E-13 | 2.22E-14 | -0.97 | 1.82E-06 | 1.38E-07 | -1.97 |
| 220615_s_at | FAR2 | 1.53E-09 | 1.27E-10 | -1.07 | 3.05E-06 | 3.35E-07 | -0.82 | 8.32E-06 | 7.80E-07 | -0.89 |
| 212239_at | PIK3R1 | 2.38E-14 | 7.07E-16 | -1.07 | 5.95E-10 | 2.85E-11 | -0.77 | 1.72E-12 | 1.84E-14 | -1.20 |
| 1558280_s_at | ARHGAP29 | 1.61E-10 | 1.09E-11 | -1.07 | 1.31E-07 | 1.04E-08 | -0.80 | 6.70E-16 | 2.73E-18 | -1.89 |
| 216620_s_at | ARHGEF10 | 2.07E-12 | 9.47E-14 | -1.07 | 1.32E-23 | 2.19E-26 | -1.11 | 2.98E-16 | 1.05E-18 | -1.20 |
| 202156_s_at | CELF2 | 6.70E-15 | 1.75E-16 | -1.07 | 1.03E-06 | 1.01E-07 | -0.83 | 1.41E-07 | 7.45E-09 | -0.82 |
| 238458_at | MICU3 | 1.29E-10 | 8.59E-12 | -1.06 | 1.86E-08 | 1.23E-09 | -1.13 | 6.55E-07 | 4.30E-08 | -1.16 |
| 205568_at | AQP9 | 4.89E-08 | 5.52E-09 | -1.06 | 1.27E-08 | 8.13E-10 | -1.36 | 5.53E-06 | 4.89E-07 | -1.39 |
| 228496_s_at | CRIM1 | 5.03E-10 | 3.80E-11 | -1.06 | 6.07E-11 | 2.33E-12 | -0.95 | 4.30E-08 | 1.96E-09 | -0.84 |
| 204204_at | SLC31A2 | 8.72E-12 | 4.58E-13 | -1.06 | 1.20E-07 | 9.51E-09 | -0.88 | 6.49E-07 | 4.26E-08 | -0.82 |
| 204048_s_at | PHACTR2 | 1.75E-14 | 5.02E-16 | -1.06 | 3.40E-07 | 2.97E-08 | -0.98 | 5.66E-11 | 1.06E-12 | -0.96 |
| 220301_at | CCDC102B | 2.36E-10 | 1.66E-11 | -1.06 | 1.31E-15 | 1.75E-17 | -1.57 | 2.34E-08 | 9.81E-10 | -1.38 |
| 242324_x_at | CCBE1 | 2.59E-13 | 9.73E-15 | -1.06 | 3.87E-27 | 2.32E-30 | -1.80 | 5.80E-14 | 4.17E-16 | -2.24 |
| 211985_s_at | CALM1 | 1.69E-13 | 6.05E-15 | -1.06 | 9.18E-05 | 1.47E-05 | -0.73 | 1.74E-11 | 2.82E-13 | -0.74 |
| 212993_at | NACC2 | 7.05E-16 | 1.44E-17 | -1.06 | 1.12E-12 | 2.86E-14 | -0.81 | 8.35E-10 | 2.28E-11 | -0.83 |
| 206874_s_at | SLK | 5.41E-16 | 1.07E-17 | -1.05 | 2.59E-15 | 3.73E-17 | -0.89 | 2.04E-07 | 1.12E-08 | -0.73 |
| 226701_at | GJA5 | 8.45E-07 | 1.22E-07 | -1.05 | 2.55E-07 | 2.17E-08 | -1.13 | 6.25E-06 | 5.63E-07 | -1.16 |
| 213371_at | LDB3 | 1.35E-12 | 5.93E-14 | -1.05 | 3.55E-06 | 3.98E-07 | -0.82 | 2.97E-08 | 1.29E-09 | -2.20 |
| 209191_at | TUBB6 | 2.00E-11 | 1.13E-12 | -1.05 | 8.02E-12 | 2.52E-13 | -1.19 | 3.81E-11 | 6.81E-13 | -1.29 |
| 228905_at | PCM1 | 7.37E-11 | 4.64E-12 | -1.05 | 4.95E-09 | 2.90E-10 | -0.97 | 3.92E-09 | 1.33E-10 | -1.13 |
| 229504_at |  | 1.19E-13 | 4.13E-15 | -1.05 | 1.13E-10 | 4.64E-12 | -1.05 | 6.66E-12 | 9.13E-14 | -1.09 |
| 1566482_at |  | 5.96E-10 | 4.55E-11 | -1.05 | 1.45E-05 | 1.89E-06 | -0.72 | 2.81E-12 | 3.31E-14 | -1.88 |
| 209866_s_at | LPHN3 | 3.15E-09 | 2.81E-10 | -1.05 | 4.75E-15 | 7.30E-17 | -1.33 | 4.97E-11 | 9.14E-13 | -2.31 |
| 210517_s_at | AKAP12 | 5.46E-07 | 7.59E-08 | -1.04 | 3.05E-13 | 6.87E-15 | -2.00 | 5.17E-08 | 2.41E-09 | -1.28 |
| 225442_at | DDR2 | 5.24E-12 | 2.63E-13 | -1.04 | 2.44E-08 | 1.65E-09 | -0.87 | 5.94E-06 | 5.31E-07 | -0.89 |
| 206522_at | MGAM | 2.54E-07 | 3.31E-08 | -1.04 | 3.10E-07 | 2.69E-08 | -1.37 | 1.91E-05 | 1.99E-06 | -1.67 |
| 211896_s_at | DCN | 1.80E-08 | 1.86E-09 | -1.04 | 3.67E-06 | 4.13E-07 | -0.92 | 4.45E-05 | 5.22E-06 | -0.90 |
| 232539_at |  | 4.16E-11 | 2.50E-12 | -1.04 | 1.14E-08 | 7.24E-10 | -0.95 | 4.40E-08 | 2.01E-09 | -1.75 |
| 227620_at | SLC44A1 | 2.03E-15 | 4.70E-17 | -1.04 | 6.75E-11 | 2.61E-12 | -0.88 | 2.52E-09 | 8.00E-11 | -0.86 |
| 208056_s_at | CBFA2T3 | 2.68E-19 | 1.94E-21 | -1.04 | 6.76E-21 | 2.29E-23 | -1.16 | 1.20E-12 | 1.22E-14 | -1.36 |
| 201147_s_at | TIMP3 | 1.39E-07 | 1.71E-08 | -1.04 | 5.44E-10 | 2.59E-11 | -1.18 | 1.93E-05 | 2.02E-06 | -0.92 |
| 230690_at | TUBB1 | 1.70E-10 | 1.16E-11 | -1.04 | 1.89E-11 | 6.43E-13 | -1.30 | 2.07E-10 | 4.59E-12 | -1.51 |
| 1598_g_at | GAS6 | 2.39E-16 | 4.35E-18 | -1.04 | 1.73E-11 | 5.83E-13 | -0.77 | 5.31E-07 | 3.41E-08 | -0.86 |
| 238905_at | RHOJ | 7.93E-14 | 2.65E-15 | -1.04 | 3.39E-19 | 1.79E-21 | -1.16 | 9.85E-11 | 2.00E-12 | -1.37 |
| 244317_at | KIAA1324L | 2.39E-09 | 2.07E-10 | -1.04 | 2.67E-05 | 3.72E-06 | -0.90 | 7.84E-06 | 7.29E-07 | -0.95 |
| 1553639_a_at | PPARGC1B | 6.78E-14 | 2.23E-15 | -1.04 | 8.95E-13 | 2.24E-14 | -0.88 | 9.24E-07 | 6.38E-08 | -0.88 |
| 205528_s_at | RUNX1T1 | 2.09E-10 | 1.46E-11 | -1.03 | 8.36E-07 | 8.04E-08 | -0.88 | 1.33E-08 | 5.16E-10 | -1.38 |
| 232081_at | ABCG1 | 2.25E-10 | 1.58E-11 | -1.03 | 1.20E-09 | 6.13E-11 | -0.86 | 1.84E-07 | 1.00E-08 | -1.01 |
| 242342_at | GUCY1A2 | 3.17E-13 | 1.22E-14 | -1.03 | 3.15E-10 | 1.41E-11 | -0.85 | 2.31E-11 | 3.88E-13 | -1.70 |
| 208958_at | ERP44 | 3.39E-15 | 8.27E-17 | -1.03 | 1.18E-13 | 2.42E-15 | -0.91 | 3.00E-10 | 7.07E-12 | -1.03 |
| 52975_at | MVB12B | 3.19E-16 | 5.98E-18 | -1.03 | 3.87E-12 | 1.12E-13 | -0.84 | 1.87E-12 | 2.04E-14 | -1.08 |
| 221031_s_at | APOLD1 | 4.20E-05 | 8.72E-06 | -1.03 | 9.85E-11 | 3.96E-12 | -1.76 | 3.83E-15 | 1.88E-17 | -2.30 |
| 212681_at | EPB41L3 | 2.42E-10 | 1.71E-11 | -1.03 | 1.79E-11 | 6.06E-13 | -1.27 | 1.26E-09 | 3.63E-11 | -1.11 |
| 239725_at | PGAP1 | 6.17E-13 | 2.53E-14 | -1.03 | 2.19E-06 | 2.33E-07 | -0.85 | 1.17E-14 | 6.76E-17 | -1.15 |
| 228359_at | UBASH3B | 3.52E-09 | 3.18E-10 | -1.03 | 9.57E-14 | 1.93E-15 | -0.96 | 8.49E-06 | 7.96E-07 | -1.01 |
| 202350_s_at | MATN2 | 5.86E-05 | 1.26E-05 | -1.03 | 4.78E-08 | 3.44E-09 | -0.93 | 4.35E-06 | 3.71E-07 | -1.27 |
| 235175_at | GBP4 | 9.93E-09 | 9.75E-10 | -1.03 | 4.30E-17 | 4.04E-19 | -1.48 | 4.60E-05 | 5.42E-06 | -0.98 |
| 239229_at | PHEX | 1.99E-08 | 2.08E-09 | -1.03 | 2.15E-11 | 7.41E-13 | -1.35 | 1.05E-09 | 2.97E-11 | -1.76 |
| 212609_s_at | AKT3 | 1.78E-13 | 6.40E-15 | -1.02 | 3.36E-13 | 7.68E-15 | -1.05 | 5.48E-14 | 3.92E-16 | -1.42 |
| 222317_at | PDE3B | 2.56E-13 | 9.61E-15 | -1.02 | 1.32E-14 | 2.23E-16 | -1.18 | 1.27E-09 | 3.65E-11 | -1.41 |
| 237058_x_at | SLC6A13 | 8.67E-11 | 5.54E-12 | -1.02 | 3.11E-12 | 8.80E-14 | -0.73 | 1.07E-06 | 7.52E-08 | -1.38 |
| 204790_at | SMAD7 | 7.95E-12 | 4.16E-13 | -1.02 | 2.44E-08 | 1.65E-09 | -0.84 | 2.67E-08 | 1.14E-09 | -0.72 |
| 206390_x_at | PF4 | 1.86E-08 | 1.93E-09 | -1.02 | 1.25E-08 | 7.98E-10 | -0.92 | 7.43E-07 | 4.99E-08 | -1.47 |
| 218029_at | FAM65A | 4.66E-16 | 9.03E-18 | -1.02 | 1.14E-18 | 7.05E-21 | -0.92 | 2.33E-10 | 5.28E-12 | -1.16 |
| 228857_at | GNL1 | 1.83E-17 | 2.41E-19 | -1.02 | 2.08E-13 | 4.50E-15 | -0.81 | 7.62E-12 | 1.07E-13 | -1.06 |
| 212538_at | DOCK9 | 1.48E-11 | 8.13E-13 | -1.02 | 2.72E-07 | 2.32E-08 | -0.81 | 5.81E-08 | 2.75E-09 | -0.83 |
| 236698_at |  | 5.22E-09 | 4.88E-10 | -1.02 | 3.38E-11 | 1.22E-12 | -1.13 | 1.80E-08 | 7.26E-10 | -1.28 |
| 212450_at | SECISBP2L | 1.66E-13 | 5.92E-15 | -1.02 | 4.41E-14 | 8.33E-16 | -1.03 | 1.47E-12 | 1.56E-14 | -0.92 |
| 242033_at | RNF180 | 1.24E-11 | 6.71E-13 | -1.02 | 2.73E-06 | 2.97E-07 | -0.83 | 6.81E-05 | 8.48E-06 | -0.99 |
| 232235_at | DSEL | 5.08E-08 | 5.75E-09 | -1.02 | 5.93E-07 | 5.51E-08 | -0.98 | 1.32E-05 | 1.31E-06 | -0.92 |
| 234987_at | SAMHD1 | 1.07E-11 | 5.70E-13 | -1.01 | 9.63E-08 | 7.47E-09 | -0.70 | 6.54E-08 | 3.14E-09 | -0.85 |
| 202947_s_at | GYPC | 1.70E-15 | 3.88E-17 | -1.01 | 1.19E-17 | 9.78E-20 | -1.06 | 3.73E-11 | 6.64E-13 | -1.05 |
| 202202_s_at | LAMA4 | 1.18E-12 | 5.13E-14 | -1.01 | 4.33E-11 | 1.60E-12 | -1.04 | 3.85E-09 | 1.30E-10 | -1.08 |
| 227613_at | ZNF331 | 4.42E-09 | 4.07E-10 | -1.01 | 2.53E-14 | 4.54E-16 | -1.66 | 2.49E-13 | 2.11E-15 | -1.78 |
| 230913_at | ABCG1 | 4.32E-07 | 5.90E-08 | -1.01 | 3.43E-07 | 3.00E-08 | -0.90 | 4.67E-08 | 2.15E-09 | -1.12 |
| 232227_at | LOC100505976 | 2.62E-13 | 9.86E-15 | -1.01 | 1.88E-15 | 2.62E-17 | -1.08 | 2.43E-10 | 5.56E-12 | -1.37 |
| 206710_s_at | EPB41L3 | 1.85E-07 | 2.34E-08 | -1.01 | 2.49E-08 | 1.69E-09 | -1.36 | 5.01E-08 | 2.32E-09 | -1.09 |
| 223843_at | SCARA3 | 1.60E-12 | 7.11E-14 | -1.01 | 8.83E-12 | 2.79E-13 | -1.08 | 8.63E-05 | 1.11E-05 | -1.36 |
| 209210_s_at | FERMT2 | 1.02E-14 | 2.75E-16 | -1.01 | 4.54E-15 | 6.94E-17 | -1.19 | 1.44E-11 | 2.28E-13 | -0.78 |
| 206528_at | TRPC6 | 6.74E-09 | 6.42E-10 | -1.01 | 1.05E-13 | 2.12E-15 | -1.09 | 1.42E-07 | 7.54E-09 | -1.07 |
| 1554762_a_at | WWC2 | 6.09E-12 | 3.09E-13 | -1.01 | 6.74E-09 | 4.07E-10 | -0.86 | 1.65E-09 | 4.94E-11 | -0.74 |
| 204063_s_at | ULK2 | 2.38E-13 | 8.85E-15 | -1.01 | 7.33E-11 | 2.86E-12 | -0.79 | 2.31E-09 | 7.23E-11 | -0.84 |
| 40687_at | GJA4 | 4.68E-13 | 1.88E-14 | -1.00 | 6.25E-15 | 9.87E-17 | -1.05 | 3.87E-17 | 9.77E-20 | -1.41 |
| 211105_s_at | NFATC1 | 2.40E-12 | 1.11E-13 | -1.00 | 6.40E-08 | 4.77E-09 | -0.78 | 7.36E-08 | 3.58E-09 | -0.74 |
| 203006_at | INPP5A | 6.61E-14 | 2.17E-15 | -1.00 | 2.23E-16 | 2.52E-18 | -0.89 | 5.53E-17 | 1.48E-19 | -1.08 |
| 201148_s_at | TIMP3 | 1.22E-08 | 1.22E-09 | -1.00 | 3.29E-14 | 6.01E-16 | -1.09 | 1.09E-11 | 1.63E-13 | -1.63 |
| 219243_at | GIMAP4 | 1.88E-10 | 1.30E-11 | -1.00 | 3.32E-15 | 4.92E-17 | -1.32 | 5.77E-06 | 5.14E-07 | -0.92 |
| 206868_at | STARD8 | 4.73E-21 | 1.75E-23 | -1.00 | 1.65E-16 | 1.78E-18 | -0.97 | 7.72E-23 | 1.41E-26 | -1.48 |
| 205119_s_at | FPR1 | 3.47E-07 | 4.65E-08 | -1.00 | 2.52E-12 | 6.97E-14 | -1.36 | 5.34E-09 | 1.86E-10 | -1.57 |
| 239132_at | NOS1 | 3.44E-10 | 2.50E-11 | -1.00 | 6.09E-16 | 7.67E-18 | -1.34 | 4.86E-12 | 6.39E-14 | -3.34 |
| 228063_s_at | NAP1L5 | 4.01E-11 | 2.40E-12 | -1.00 | 1.06E-07 | 8.30E-09 | -0.98 | 3.01E-09 | 9.83E-11 | -0.97 |
| 229687_s_at | LOC100287017 | 6.08E-12 | 3.09E-13 | -1.00 | 4.88E-14 | 9.28E-16 | -1.03 | 1.01E-14 | 5.64E-17 | -1.57 |
| 229705_at | PIK3C3 | 4.71E-13 | 1.89E-14 | -1.00 | 2.50E-11 | 8.78E-13 | -1.00 | 2.10E-12 | 2.32E-14 | -1.09 |
| 227705_at | TCEAL7 | 2.62E-09 | 2.29E-10 | -1.00 | 6.28E-08 | 4.67E-09 | -1.18 | 8.12E-08 | 4.01E-09 | -1.21 |
| 208960_s_at | KLF6 | 6.04E-08 | 6.97E-09 | -1.00 | 3.64E-13 | 8.38E-15 | -1.61 | 1.22E-07 | 6.36E-09 | -1.01 |
| 201626_at | INSIG1 | 4.50E-10 | 3.37E-11 | -1.00 | 4.79E-10 | 2.25E-11 | -0.77 | 1.38E-09 | 4.03E-11 | -0.91 |
| 205193_at | MAFF | 2.94E-07 | 3.87E-08 | -1.00 | 9.34E-13 | 2.36E-14 | -1.35 | 1.62E-09 | 4.82E-11 | -1.16 |
| 238906_s_at | RHOJ | 2.36E-10 | 1.66E-11 | -1.00 | 8.43E-15 | 1.35E-16 | -1.77 | 1.17E-11 | 1.80E-13 | -1.50 |
| 236304_at |  | 1.23E-14 | 3.41E-16 | -1.00 | 4.61E-11 | 1.72E-12 | -0.97 | 2.07E-11 | 3.42E-13 | -1.70 |
| 219477_s_at | THSD1P1///THSD1 | 5.34E-08 | 6.08E-09 | -1.00 | 6.25E-15 | 9.85E-17 | -1.05 | 2.10E-13 | 1.76E-15 | -1.10 |
| 204153_s_at | MFNG | 2.30E-14 | 6.80E-16 | -1.00 | 5.46E-19 | 3.08E-21 | -1.00 | 3.03E-12 | 3.64E-14 | -1.10 |
| 225790_at | MSRB3 | 7.72E-10 | 6.03E-11 | -0.99 | 7.13E-11 | 2.77E-12 | -1.36 | 6.79E-10 | 1.81E-11 | -1.27 |
| 241310_at |  | 2.33E-05 | 4.58E-06 | -0.99 | 3.54E-06 | 3.96E-07 | -1.01 | 1.11E-06 | 7.90E-08 | -1.51 |
| 202747_s_at | ITM2A | 1.85E-10 | 1.27E-11 | -0.99 | 6.16E-21 | 2.04E-23 | -1.52 | 2.47E-09 | 7.84E-11 | -1.33 |
| 228456_s_at | CDS2 | 6.53E-20 | 3.86E-22 | -0.99 | 1.68E-13 | 3.55E-15 | -0.88 | 3.70E-09 | 1.24E-10 | -0.87 |
| 212859_x_at | MT1E | 5.68E-07 | 7.92E-08 | -0.99 | 1.08E-05 | 1.36E-06 | -0.77 | 1.02E-06 | 7.18E-08 | -1.17 |
| 239845_at |  | 1.30E-15 | 2.87E-17 | -0.99 | 4.32E-10 | 2.00E-11 | -0.81 | 9.08E-20 | 8.14E-23 | -1.59 |
| 200986_at | SERPING1 | 3.25E-13 | 1.25E-14 | -0.99 | 8.61E-13 | 2.14E-14 | -1.06 | 4.87E-08 | 2.25E-09 | -0.88 |
| 239270_at | PLCXD3 | 1.23E-09 | 1.00E-10 | -0.99 | 3.41E-09 | 1.93E-10 | -0.93 | 4.24E-06 | 3.61E-07 | -2.01 |
| 235658_at |  | 1.49E-16 | 2.55E-18 | -0.98 | 2.95E-10 | 1.32E-11 | -0.77 | 5.71E-07 | 3.69E-08 | -0.92 |
| 204683_at | ICAM2 | 1.74E-13 | 6.25E-15 | -0.98 | 3.57E-20 | 1.46E-22 | -1.28 | 1.64E-10 | 3.55E-12 | -0.98 |
| 227859_at | DNAJC27 | 1.11E-16 | 1.80E-18 | -0.98 | 1.00E-10 | 4.05E-12 | -0.92 | 2.70E-11 | 4.61E-13 | -0.99 |
| 207624_s_at | RPGR | 3.39E-15 | 8.28E-17 | -0.98 | 3.26E-09 | 1.84E-10 | -0.92 | 2.87E-10 | 6.70E-12 | -1.07 |
| 200965_s_at | ABLIM1 | 6.44E-13 | 2.65E-14 | -0.98 | 6.03E-18 | 4.54E-20 | -1.23 | 1.43E-07 | 7.57E-09 | -0.99 |
| 230183_at | EXT1 | 3.16E-10 | 2.28E-11 | -0.98 | 3.79E-08 | 2.67E-09 | -0.70 | 1.09E-10 | 2.24E-12 | -0.89 |
| 207111_at | EMR1 | 1.03E-08 | 1.02E-09 | -0.98 | 5.05E-16 | 6.26E-18 | -1.21 | 2.59E-09 | 8.27E-11 | -1.81 |
| 209993_at | ABCB1 | 1.44E-07 | 1.78E-08 | -0.98 | 8.02E-10 | 3.94E-11 | -1.26 | 8.74E-11 | 1.75E-12 | -2.01 |
| 228444_at | ARRB1 | 1.87E-11 | 1.05E-12 | -0.98 | 3.59E-17 | 3.28E-19 | -0.97 | 1.02E-09 | 2.86E-11 | -1.19 |
| 226267_at | JDP2 | 6.19E-14 | 2.01E-15 | -0.98 | 3.38E-13 | 7.74E-15 | -0.88 | 3.85E-06 | 3.24E-07 | -0.71 |
| 1559777_at | LOC731424 | 1.13E-08 | 1.12E-09 | -0.98 | 1.08E-11 | 3.47E-13 | -1.14 | 2.46E-07 | 1.40E-08 | -1.18 |
| 205638_at | BAI3 | 2.72E-14 | 8.21E-16 | -0.97 | 2.31E-18 | 1.55E-20 | -1.17 | 1.65E-08 | 6.59E-10 | -2.10 |
| 207358_x_at | MACF1 | 1.02E-16 | 1.65E-18 | -0.97 | 3.28E-16 | 3.81E-18 | -0.79 | 1.60E-10 | 3.44E-12 | -0.74 |
| 212308_at | CLASP2 | 2.75E-14 | 8.30E-16 | -0.97 | 1.34E-06 | 1.35E-07 | -0.75 | 2.58E-10 | 5.94E-12 | -0.83 |
| 228311_at | BCL6B | 4.71E-15 | 1.19E-16 | -0.97 | 1.43E-18 | 9.07E-21 | -1.01 | 7.07E-18 | 1.38E-20 | -1.33 |
| 212253_x_at | DST | 5.41E-09 | 5.06E-10 | -0.97 | 1.84E-10 | 7.88E-12 | -0.80 | 3.64E-08 | 1.62E-09 | -1.15 |
| 221087_s_at | APOL3 | 2.62E-10 | 1.86E-11 | -0.97 | 2.53E-16 | 2.89E-18 | -1.06 | 2.95E-09 | 9.61E-11 | -1.13 |
| 228201_at | ARL13B | 1.58E-11 | 8.70E-13 | -0.97 | 6.44E-10 | 3.10E-11 | -1.04 | 1.46E-11 | 2.33E-13 | -0.75 |
| 228097_at | MYLIP | 3.09E-08 | 3.36E-09 | -0.97 | 1.61E-07 | 1.31E-08 | -0.95 | 2.74E-05 | 3.01E-06 | -0.95 |
| 205752_s_at | GSTM5 | 9.76E-15 | 2.62E-16 | -0.97 | 7.64E-13 | 1.88E-14 | -0.78 | 5.06E-11 | 9.35E-13 | -1.48 |
| 230048_at | IFRD1 | 9.04E-10 | 7.18E-11 | -0.97 | 1.16E-09 | 5.94E-11 | -1.18 | 1.12E-11 | 1.70E-13 | -1.04 |
| 224925_at | PREX1 | 4.73E-16 | 9.19E-18 | -0.97 | 1.84E-16 | 2.00E-18 | -1.00 | 3.26E-10 | 7.83E-12 | -1.16 |
| 225390_s_at | KLF13 | 3.83E-14 | 1.19E-15 | -0.97 | 2.42E-12 | 6.65E-14 | -1.02 | 6.53E-12 | 8.89E-14 | -1.00 |
| 238484_s_at | SSBP2 | 3.11E-15 | 7.49E-17 | -0.97 | 1.58E-06 | 1.63E-07 | -0.71 | 9.06E-06 | 8.56E-07 | -1.00 |
| 242436_at |  | 7.43E-09 | 7.12E-10 | -0.96 | 1.79E-12 | 4.79E-14 | -1.19 | 3.89E-07 | 2.37E-08 | -1.55 |
| 236922_at | NCK1 | 6.63E-14 | 2.18E-15 | -0.96 | 3.86E-09 | 2.22E-10 | -0.87 | 1.44E-08 | 5.65E-10 | -0.75 |
| 230708_at | PRICKLE1 | 9.82E-09 | 9.62E-10 | -0.96 | 2.06E-05 | 2.79E-06 | -0.76 | 8.18E-06 | 7.64E-07 | -0.98 |
| 208131_s_at | PTGIS | 7.71E-07 | 1.10E-07 | -0.96 | 9.49E-08 | 7.35E-09 | -1.15 | 2.56E-05 | 2.78E-06 | -1.12 |
| 206236_at | GPR4 | 4.22E-09 | 3.87E-10 | -0.96 | 1.74E-10 | 7.40E-12 | -0.91 | 1.08E-13 | 8.37E-16 | -1.05 |
| 1553133_at | C9orf72 | 2.91E-12 | 1.37E-13 | -0.96 | 1.85E-08 | 1.23E-09 | -1.01 | 8.31E-06 | 7.78E-07 | -1.03 |
| 242607_at |  | 8.16E-14 | 2.74E-15 | -0.96 | 4.53E-08 | 3.25E-09 | -0.84 | 7.77E-12 | 1.10E-13 | -1.05 |
| 226485_at | VSIG10 | 1.66E-08 | 1.70E-09 | -0.95 | 5.19E-07 | 4.74E-08 | -0.81 | 9.92E-05 | 1.30E-05 | -0.73 |
| 204500_s_at | AGTPBP1 | 9.13E-15 | 2.45E-16 | -0.95 | 4.58E-24 | 6.79E-27 | -1.07 | 1.41E-14 | 8.48E-17 | -0.82 |
| 229452_at | TMEM88 | 3.15E-14 | 9.67E-16 | -0.95 | 8.89E-19 | 5.33E-21 | -1.07 | 9.61E-13 | 9.54E-15 | -1.91 |
| 220006_at | EFCC1 | 8.00E-16 | 1.66E-17 | -0.95 | 9.32E-19 | 5.66E-21 | -0.99 | 2.84E-07 | 1.66E-08 | -1.96 |
| 210174_at | NR5A2 | 1.65E-09 | 1.38E-10 | -0.95 | 5.54E-08 | 4.06E-09 | -1.00 | 8.42E-08 | 4.18E-09 | -1.21 |
| 235320_at | ARL6 | 1.30E-10 | 8.60E-12 | -0.95 | 2.36E-08 | 1.59E-09 | -0.91 | 1.75E-12 | 1.89E-14 | -0.85 |
| 205929_at | GPA33 | 7.28E-07 | 1.04E-07 | -0.95 | 3.74E-10 | 1.71E-11 | -0.98 | 1.91E-11 | 3.13E-13 | -2.50 |
| 237465_at | USP53 | 1.19E-07 | 1.45E-08 | -0.95 | 1.62E-09 | 8.54E-11 | -1.05 | 1.68E-07 | 9.05E-09 | -0.93 |
| 217287_s_at | TRPC6 | 2.22E-07 | 2.86E-08 | -0.95 | 3.45E-10 | 1.56E-11 | -1.08 | 6.56E-12 | 8.95E-14 | -1.60 |
| 207943_x_at | PLAGL1 | 6.17E-12 | 3.14E-13 | -0.95 | 1.09E-07 | 8.54E-09 | -0.77 | 6.44E-09 | 2.30E-10 | -0.90 |
| 204134_at | PDE2A | 4.57E-13 | 1.83E-14 | -0.95 | 1.81E-17 | 1.56E-19 | -1.31 | 2.12E-09 | 6.59E-11 | -1.42 |
| 202388_at | RGS2 | 8.80E-09 | 8.54E-10 | -0.94 | 1.06E-12 | 2.70E-14 | -1.43 | 3.05E-06 | 2.49E-07 | -1.07 |
| 220751_s_at | FAXDC2 | 1.88E-05 | 3.62E-06 | -0.94 | 5.39E-12 | 1.63E-13 | -1.15 | 6.86E-06 | 6.27E-07 | -1.19 |
| 37384_at | PPM1F | 4.53E-14 | 1.43E-15 | -0.94 | 7.45E-18 | 5.79E-20 | -1.09 | 2.74E-18 | 4.66E-21 | -1.33 |
| 227410_at | FAM43A | 2.21E-06 | 3.47E-07 | -0.94 | 9.70E-11 | 3.89E-12 | -1.30 | 5.08E-11 | 9.39E-13 | -1.25 |
| 1555233_at | RHOJ | 4.07E-07 | 5.53E-08 | -0.94 | 2.56E-11 | 9.02E-13 | -1.58 | 4.10E-14 | 2.79E-16 | -1.64 |
| 45297_at | EHD2 | 1.43E-10 | 9.57E-12 | -0.94 | 6.42E-12 | 1.97E-13 | -0.79 | 8.30E-12 | 1.19E-13 | -1.03 |
| 224964_s_at | GNG2 | 1.15E-10 | 7.54E-12 | -0.94 | 2.20E-13 | 4.80E-15 | -1.13 | 2.56E-06 | 2.04E-07 | -0.80 |
| 229544_at |  | 7.05E-13 | 2.93E-14 | -0.94 | 2.41E-08 | 1.63E-09 | -0.99 | 6.32E-09 | 2.25E-10 | -1.11 |
| 220936_s_at | H2AFJ | 6.34E-12 | 3.24E-13 | -0.94 | 8.25E-10 | 4.06E-11 | -0.87 | 9.55E-12 | 1.41E-13 | -1.06 |
| 239300_at | PIK3C3 | 1.25E-11 | 6.75E-13 | -0.94 | 1.59E-12 | 4.23E-14 | -0.98 | 8.06E-12 | 1.15E-13 | -1.19 |
| 201810_s_at | SH3BP5 | 6.26E-14 | 2.04E-15 | -0.93 | 2.25E-13 | 4.92E-15 | -1.17 | 2.10E-12 | 2.30E-14 | -1.23 |
| 202499_s_at | SLC2A3 | 1.10E-05 | 2.01E-06 | -0.93 | 1.20E-08 | 7.59E-10 | -1.46 | 7.30E-05 | 9.17E-06 | -1.11 |
| 241926_s_at | ERG | 1.83E-09 | 1.55E-10 | -0.93 | 3.64E-13 | 8.40E-15 | -1.51 | 6.31E-17 | 1.74E-19 | -1.39 |
| 207480_s_at | MEIS2 | 8.08E-10 | 6.34E-11 | -0.93 | 8.16E-09 | 5.01E-10 | -0.87 | 2.85E-10 | 6.63E-12 | -1.06 |
| 200953_s_at | CCND2 | 4.06E-11 | 2.43E-12 | -0.93 | 2.68E-08 | 1.83E-09 | -0.87 | 1.51E-05 | 1.52E-06 | -0.78 |
| 230151_at | SPRYD7 | 3.00E-12 | 1.42E-13 | -0.93 | 1.33E-15 | 1.78E-17 | -1.16 | 7.16E-18 | 1.41E-20 | -1.21 |
| 205303_at | KCNJ8 | 2.03E-10 | 1.41E-11 | -0.93 | 2.47E-16 | 2.82E-18 | -1.29 | 3.23E-11 | 5.65E-13 | -1.43 |
| 229934_at |  | 9.52E-11 | 6.13E-12 | -0.93 | 6.66E-09 | 4.02E-10 | -1.07 | 2.39E-05 | 2.57E-06 | -1.71 |
| 218665_at | FZD4 | 3.41E-10 | 2.47E-11 | -0.93 | 6.27E-18 | 4.75E-20 | -1.24 | 4.10E-24 | 3.00E-28 | -1.69 |
| 225582_at | ITPRIP | 1.78E-11 | 9.95E-13 | -0.93 | 3.25E-13 | 7.39E-15 | -1.05 | 1.21E-10 | 2.53E-12 | -1.15 |
| 213469_at | PGAP1 | 5.64E-11 | 3.49E-12 | -0.93 | 9.86E-09 | 6.16E-10 | -1.04 | 1.22E-12 | 1.26E-14 | -1.09 |
| 226152_at | TTC7B | 4.37E-14 | 1.37E-15 | -0.93 | 8.59E-10 | 4.26E-11 | -0.76 | 4.58E-11 | 8.34E-13 | -0.85 |
| 225373_at | C10orf54 | 2.20E-13 | 8.13E-15 | -0.93 | 9.33E-22 | 2.61E-24 | -1.06 | 2.37E-09 | 7.47E-11 | -0.72 |
| 235497_at | LINC01128 | 3.45E-13 | 1.34E-14 | -0.93 | 1.42E-06 | 1.45E-07 | -0.71 | 2.38E-07 | 1.35E-08 | -0.89 |
| 1555993_at | CACNA1D | 1.74E-07 | 2.20E-08 | -0.93 | 3.61E-07 | 3.17E-08 | -0.75 | 1.48E-08 | 5.83E-10 | -1.15 |
| 236264_at | LPHN3 | 9.32E-10 | 7.42E-11 | -0.92 | 1.34E-14 | 2.28E-16 | -1.03 | 4.84E-11 | 8.86E-13 | -2.51 |
| 219958_at | TMEM74B | 2.54E-13 | 9.52E-15 | -0.92 | 2.47E-19 | 1.27E-21 | -0.99 | 1.39E-16 | 4.21E-19 | -1.36 |
| 230917_at |  | 3.91E-10 | 2.88E-11 | -0.92 | 7.40E-11 | 2.89E-12 | -0.93 | 8.75E-05 | 1.13E-05 | -0.95 |
| 226633_at | RAB8B | 2.38E-19 | 1.68E-21 | -0.92 | 6.67E-16 | 8.49E-18 | -1.01 | 3.46E-09 | 1.15E-10 | -0.71 |
| 202150_s_at | NEDD9 | 6.29E-07 | 8.83E-08 | -0.92 | 5.65E-13 | 1.36E-14 | -1.29 | 1.24E-05 | 1.22E-06 | -0.98 |
| 204499_at | AGTPBP1 | 6.67E-17 | 1.01E-18 | -0.92 | 1.86E-18 | 1.22E-20 | -0.92 | 1.67E-07 | 9.01E-09 | -1.06 |
| 211626_x_at | ERG | 4.04E-17 | 5.79E-19 | -0.92 | 9.29E-22 | 2.58E-24 | -1.10 | 3.65E-07 | 2.20E-08 | -1.04 |
| 230933_at | DSTN | 2.67E-18 | 2.76E-20 | -0.92 | 2.42E-08 | 1.64E-09 | -0.79 | 1.27E-05 | 1.25E-06 | -1.01 |
| 229822_at |  | 2.24E-16 | 4.06E-18 | -0.92 | 4.29E-17 | 4.01E-19 | -0.90 | 8.42E-12 | 1.21E-13 | -1.14 |
| 225914_s_at | CAB39L | 5.36E-12 | 2.69E-13 | -0.92 | 6.94E-14 | 1.36E-15 | -1.00 | 1.37E-09 | 4.00E-11 | -0.96 |
| 229781_at | LOC100506725 | 1.60E-12 | 7.14E-14 | -0.92 | 3.53E-17 | 3.21E-19 | -1.13 | 2.44E-05 | 2.63E-06 | -1.19 |
| 209481_at | SNRK | 2.41E-18 | 2.45E-20 | -0.92 | 4.20E-17 | 3.92E-19 | -1.14 | 2.80E-16 | 9.67E-19 | -1.10 |
| 222171_s_at | PKNOX2 | 1.58E-14 | 4.49E-16 | -0.91 | 2.17E-19 | 1.11E-21 | -1.00 | 1.11E-10 | 2.29E-12 | -1.92 |
| 53991_at | DENND2A | 2.73E-14 | 8.24E-16 | -0.91 | 1.56E-19 | 7.51E-22 | -1.04 | 5.11E-14 | 3.62E-16 | -1.39 |
| 229967_at | CMTM2 | 1.57E-07 | 1.96E-08 | -0.91 | 1.38E-10 | 5.78E-12 | -1.71 | 2.08E-08 | 8.54E-10 | -1.44 |
| 216944_s_at | ITPR1 | 2.43E-11 | 1.40E-12 | -0.91 | 2.21E-08 | 1.48E-09 | -0.80 | 2.71E-07 | 1.57E-08 | -0.86 |
| 225504_at | HMBOX1 | 7.09E-17 | 1.09E-18 | -0.91 | 4.37E-16 | 5.30E-18 | -0.75 | 2.45E-14 | 1.57E-16 | -1.09 |
| 211356_x_at | LEPR | 9.72E-05 | 2.19E-05 | -0.91 | 9.95E-08 | 7.75E-09 | -1.39 | 1.96E-09 | 6.00E-11 | -1.29 |
| 222409_at | CORO1C | 5.65E-13 | 2.30E-14 | -0.91 | 2.43E-12 | 6.69E-14 | -0.75 | 6.02E-10 | 1.57E-11 | -0.73 |
| 221035_s_at | TEX14 | 4.09E-11 | 2.45E-12 | -0.91 | 1.47E-13 | 3.06E-15 | -0.74 | 4.02E-05 | 4.65E-06 | -1.45 |
| 225939_at | EIF4E3 | 4.29E-13 | 1.70E-14 | -0.91 | 1.94E-13 | 4.18E-15 | -1.07 | 1.03E-09 | 2.90E-11 | -0.88 |
| 212535_at | MEF2A | 1.26E-17 | 1.59E-19 | -0.91 | 8.63E-14 | 1.73E-15 | -0.78 | 1.21E-15 | 5.23E-18 | -0.75 |
| 205934_at | PLCL1 | 4.30E-13 | 1.70E-14 | -0.91 | 1.24E-15 | 1.65E-17 | -1.24 | 1.08E-07 | 5.53E-09 | -0.91 |
| 219820_at | SLC6A16 | 2.57E-16 | 4.71E-18 | -0.91 | 4.54E-12 | 1.34E-13 | -0.89 | 7.70E-06 | 7.15E-07 | -1.12 |
| 204007_at | FCGR3B | 1.37E-07 | 1.69E-08 | -0.90 | 9.27E-12 | 2.94E-13 | -1.63 | 8.08E-11 | 1.58E-12 | -1.95 |
| 241365_at | SATB1 | 6.28E-14 | 2.05E-15 | -0.90 | 1.52E-07 | 1.22E-08 | -0.89 | 1.93E-05 | 2.02E-06 | -0.96 |
| 226390_at | STARD4 | 1.59E-10 | 1.08E-11 | -0.90 | 9.67E-11 | 3.88E-12 | -0.77 | 4.62E-10 | 1.17E-11 | -0.89 |
| 213221_s_at | SIK2 | 4.54E-16 | 8.75E-18 | -0.90 | 1.21E-15 | 1.60E-17 | -1.01 | 4.12E-14 | 2.82E-16 | -1.02 |
| 228977_at | LOC729680 | 7.47E-09 | 7.16E-10 | -0.90 | 5.28E-13 | 1.25E-14 | -1.32 | 8.40E-09 | 3.07E-10 | -1.29 |
| 205404_at | HSD11B1 | 4.17E-07 | 5.67E-08 | -0.90 | 3.19E-07 | 2.76E-08 | -0.99 | 5.15E-05 | 6.16E-06 | -0.83 |
| 226038_at | LONRF1 | 5.14E-13 | 2.08E-14 | -0.90 | 6.21E-10 | 2.98E-11 | -0.90 | 4.67E-12 | 6.08E-14 | -1.01 |
| 205824_at | HSPB2 | 2.85E-14 | 8.64E-16 | -0.90 | 6.94E-16 | 8.85E-18 | -0.85 | 7.55E-08 | 3.68E-09 | -1.55 |
| 225972_at | TMEM64 | 7.43E-14 | 2.46E-15 | -0.89 | 1.22E-10 | 5.05E-12 | -0.89 | 1.15E-09 | 3.27E-11 | -0.98 |
| 229487_at | EBF1 | 1.85E-09 | 1.57E-10 | -0.89 | 5.76E-09 | 3.42E-10 | -0.88 | 4.46E-12 | 5.73E-14 | -1.37 |
| 213960_at | NTRK3 | 1.21E-11 | 6.54E-13 | -0.89 | 2.90E-09 | 1.62E-10 | -0.89 | 1.09E-07 | 5.58E-09 | -1.86 |
| 229699_at | LOC100129550 | 1.81E-10 | 1.24E-11 | -0.89 | 2.69E-11 | 9.53E-13 | -0.79 | 4.72E-12 | 6.16E-14 | -0.77 |
| 209867_s_at | LPHN3 | 6.81E-10 | 5.28E-11 | -0.89 | 9.47E-13 | 2.40E-14 | -0.93 | 5.86E-10 | 1.52E-11 | -2.39 |
| 238861_at | SSBP2 | 2.78E-13 | 1.05E-14 | -0.89 | 1.06E-09 | 5.36E-11 | -0.77 | 3.69E-06 | 3.08E-07 | -1.00 |
| 213413_at | STON1 | 1.06E-08 | 1.05E-09 | -0.89 | 1.27E-07 | 1.01E-08 | -1.09 | 7.68E-08 | 3.75E-09 | -1.08 |
| 226638_at | ARHGAP23 | 8.64E-13 | 3.65E-14 | -0.89 | 2.66E-11 | 9.39E-13 | -0.75 | 2.34E-08 | 9.81E-10 | -0.74 |
| 212225_at | EIF1 | 7.00E-11 | 4.39E-12 | -0.89 | 6.65E-11 | 2.57E-12 | -1.15 | 2.58E-05 | 2.81E-06 | -1.04 |
| 203710_at | ITPR1 | 2.81E-13 | 1.06E-14 | -0.89 | 9.23E-12 | 2.93E-13 | -0.89 | 4.58E-07 | 2.87E-08 | -0.81 |
| 211355_x_at | LEPR | 3.60E-05 | 7.35E-06 | -0.89 | 5.03E-08 | 3.65E-09 | -1.34 | 1.74E-08 | 6.98E-10 | -1.23 |
| 222407_s_at | ZNF106 | 2.86E-17 | 3.97E-19 | -0.89 | 9.69E-12 | 3.09E-13 | -0.89 | 2.52E-10 | 5.79E-12 | -0.83 |
| 211379_x_at | B3GALNT1 | 1.47E-06 | 2.23E-07 | -0.89 | 7.67E-09 | 4.68E-10 | -1.47 | 3.39E-07 | 2.02E-08 | -1.04 |
| 235483_at | STX3 | 1.81E-15 | 4.14E-17 | -0.89 | 6.86E-13 | 1.67E-14 | -0.78 | 2.00E-09 | 6.14E-11 | -0.85 |
| 1554012_at | RSPO2 | 2.87E-09 | 2.53E-10 | -0.88 | 1.03E-09 | 5.24E-11 | -1.01 | 6.11E-06 | 5.49E-07 | -1.79 |
| 221885_at | DENND2A | 1.94E-12 | 8.78E-14 | -0.88 | 2.20E-16 | 2.47E-18 | -0.88 | 3.89E-12 | 4.91E-14 | -1.55 |
| 202732_at | PKIG | 1.75E-11 | 9.75E-13 | -0.88 | 1.83E-17 | 1.59E-19 | -1.30 | 3.78E-12 | 4.74E-14 | -1.14 |
| 201811_x_at | SH3BP5 | 1.55E-17 | 2.01E-19 | -0.88 | 1.28E-16 | 1.35E-18 | -0.99 | 5.79E-12 | 7.74E-14 | -1.16 |
| 226646_at | KLF2 | 4.36E-14 | 1.37E-15 | -0.88 | 8.01E-10 | 3.94E-11 | -0.81 | 3.60E-05 | 4.11E-06 | -0.96 |
| 203063_at | PPM1F | 1.03E-12 | 4.41E-14 | -0.88 | 6.46E-15 | 1.02E-16 | -1.08 | 7.07E-18 | 1.38E-20 | -1.52 |
| 230800_at | ADCY4 | 5.65E-16 | 1.12E-17 | -0.88 | 3.34E-19 | 1.76E-21 | -0.95 | 2.48E-12 | 2.86E-14 | -1.09 |
| 212747_at | ANKS1A | 2.50E-14 | 7.47E-16 | -0.88 | 2.71E-14 | 4.86E-16 | -0.86 | 4.98E-19 | 6.28E-22 | -1.00 |
| 201565_s_at | ID2 | 3.41E-10 | 2.48E-11 | -0.88 | 1.95E-09 | 1.04E-10 | -0.85 | 9.49E-10 | 2.63E-11 | -0.96 |
| 218825_at | EGFL7 | 1.18E-09 | 9.61E-11 | -0.88 | 1.07E-10 | 4.33E-12 | -0.87 | 6.18E-09 | 2.19E-10 | -1.21 |
| 201787_at | FBLN1 | 1.35E-05 | 2.50E-06 | -0.88 | 3.55E-07 | 3.12E-08 | -0.98 | 1.27E-07 | 6.64E-09 | -1.57 |
| 242363_at |  | 3.90E-06 | 6.45E-07 | -0.88 | 9.36E-07 | 9.12E-08 | -0.75 | 5.24E-05 | 6.27E-06 | -0.83 |
| 228067_at | KIAA1211L | 1.46E-06 | 2.21E-07 | -0.88 | 3.71E-11 | 1.35E-12 | -0.99 | 5.88E-11 | 1.11E-12 | -1.30 |
| 229670_at |  | 8.38E-09 | 8.10E-10 | -0.88 | 1.44E-12 | 3.80E-14 | -1.17 | 2.45E-09 | 7.76E-11 | -1.09 |
| 221870_at | EHD2 | 3.62E-10 | 2.64E-11 | -0.88 | 8.30E-11 | 3.28E-12 | -0.75 | 5.43E-09 | 1.90E-10 | -1.09 |
| 231887_s_at | PALD1 | 8.85E-12 | 4.67E-13 | -0.88 | 3.82E-11 | 1.39E-12 | -0.90 | 3.46E-12 | 4.26E-14 | -1.20 |
| 213075_at | OLFML2A | 9.06E-07 | 1.31E-07 | -0.88 | 2.30E-11 | 7.97E-13 | -0.92 | 3.96E-11 | 7.11E-13 | -1.36 |
| 226186_at | TMOD2 | 1.11E-13 | 3.85E-15 | -0.88 | 3.11E-13 | 7.02E-15 | -0.89 | 9.10E-09 | 3.37E-10 | -1.04 |
| 218353_at | RGS5 | 1.20E-06 | 1.79E-07 | -0.87 | 4.34E-07 | 3.88E-08 | -0.89 | 4.13E-06 | 3.50E-07 | -1.05 |
| 213306_at | MPDZ | 3.54E-10 | 2.58E-11 | -0.87 | 2.19E-13 | 4.77E-15 | -1.07 | 3.24E-09 | 1.07E-10 | -0.85 |
| 229634_at | TMEM139 | 5.33E-07 | 7.39E-08 | -0.87 | 5.93E-14 | 1.14E-15 | -1.11 | 2.46E-05 | 2.65E-06 | -1.48 |
| 223504_at | DNAJC27 | 9.53E-11 | 6.14E-12 | -0.87 | 6.52E-09 | 3.92E-10 | -0.88 | 3.32E-12 | 4.05E-14 | -0.92 |
| 203680_at | PRKAR2B | 4.01E-08 | 4.45E-09 | -0.87 | 1.72E-05 | 2.28E-06 | -0.93 | 4.80E-05 | 5.68E-06 | -0.84 |
| 218864_at | TNS1 | 8.73E-13 | 3.69E-14 | -0.87 | 1.56E-13 | 3.27E-15 | -0.77 | 5.74E-06 | 5.11E-07 | -0.70 |
| 219970_at | GIPC2 | 3.52E-07 | 4.72E-08 | -0.87 | 1.98E-08 | 1.31E-09 | -1.13 | 9.98E-10 | 2.78E-11 | -1.79 |
| 224780_at | RBM17 | 2.46E-15 | 5.83E-17 | -0.87 | 3.65E-16 | 4.29E-18 | -0.89 | 2.03E-13 | 1.69E-15 | -0.80 |
| 213348_at | CDKN1C | 5.60E-07 | 7.79E-08 | -0.87 | 1.27E-11 | 4.15E-13 | -1.10 | 3.71E-08 | 1.65E-09 | -1.10 |
| 203071_at | SEMA3B | 5.44E-08 | 6.20E-09 | -0.87 | 1.66E-15 | 2.29E-17 | -1.12 | 2.24E-09 | 7.00E-11 | -1.38 |
| 229277_at | ADRB1 | 8.74E-12 | 4.59E-13 | -0.87 | 5.35E-18 | 3.96E-20 | -1.23 | 1.69E-11 | 2.73E-13 | -1.78 |
| 236344_at | PDE1C | 1.68E-11 | 9.32E-13 | -0.87 | 3.80E-14 | 7.04E-16 | -1.04 | 2.96E-07 | 1.74E-08 | -1.48 |
| 209822_s_at | VLDLR | 9.49E-09 | 9.26E-10 | -0.87 | 8.17E-06 | 1.00E-06 | -0.82 | 3.40E-05 | 3.84E-06 | -0.91 |
| 210772_at | FPR2 | 5.71E-06 | 9.80E-07 | -0.87 | 1.21E-12 | 3.13E-14 | -1.83 | 2.04E-11 | 3.37E-13 | -1.41 |
| 221928_at | ACACB | 3.42E-14 | 1.06E-15 | -0.87 | 5.28E-10 | 2.50E-11 | -0.73 | 7.58E-12 | 1.06E-13 | -1.34 |
| 201627_s_at | INSIG1 | 2.13E-07 | 2.73E-08 | -0.86 | 1.29E-06 | 1.30E-07 | -0.88 | 7.67E-07 | 5.17E-08 | -0.86 |
| 230511_at | CREM | 2.23E-06 | 3.50E-07 | -0.86 | 5.02E-06 | 5.86E-07 | -0.89 | 2.05E-13 | 1.71E-15 | -1.33 |
| 214370_at | S100A8 | 4.49E-05 | 9.37E-06 | -0.86 | 6.30E-08 | 4.69E-09 | -1.10 | 3.33E-05 | 3.75E-06 | -2.05 |
| 205932_s_at | MSX1 | 1.56E-05 | 2.93E-06 | -0.86 | 1.23E-08 | 7.84E-10 | -1.00 | 2.60E-12 | 3.02E-14 | -1.32 |
| 218764_at | PRKCH | 6.54E-15 | 1.71E-16 | -0.86 | 4.06E-17 | 3.76E-19 | -1.05 | 1.11E-11 | 1.68E-13 | -0.87 |
| 243730_at |  | 3.81E-10 | 2.80E-11 | -0.86 | 2.05E-15 | 2.88E-17 | -0.80 | 2.66E-08 | 1.13E-09 | -1.36 |
| 205082_s_at | AOX1 | 7.55E-09 | 7.24E-10 | -0.86 | 4.30E-11 | 1.59E-12 | -0.87 | 1.09E-10 | 2.25E-12 | -1.33 |
| 228962_at | PDE4D | 8.46E-06 | 1.50E-06 | -0.86 | 1.46E-09 | 7.59E-11 | -0.98 | 3.82E-09 | 1.29E-10 | -1.35 |
| 206834_at | HBD | 2.53E-09 | 2.21E-10 | -0.86 | 3.70E-10 | 1.69E-11 | -0.93 | 1.61E-06 | 1.20E-07 | -1.59 |
| 242541_at | ABCA9 | 5.49E-10 | 4.17E-11 | -0.86 | 9.86E-17 | 1.01E-18 | -1.12 | 3.02E-06 | 2.46E-07 | -1.44 |
| 235209_at | SBSPON | 4.39E-10 | 3.28E-11 | -0.86 | 5.77E-08 | 4.26E-09 | -1.04 | 1.97E-06 | 1.51E-07 | -1.45 |
| 204773_at | IL11RA | 1.83E-14 | 5.30E-16 | -0.85 | 9.89E-12 | 3.16E-13 | -0.75 | 7.75E-07 | 5.22E-08 | -0.80 |
| 223749_at | C1QTNF2 | 3.37E-10 | 2.45E-11 | -0.85 | 1.36E-15 | 1.83E-17 | -0.89 | 2.46E-10 | 5.62E-12 | -1.66 |
| 241801_at | PGAP1 | 3.78E-15 | 9.29E-17 | -0.85 | 1.19E-10 | 4.90E-12 | -0.93 | 8.10E-15 | 4.34E-17 | -1.42 |
| 225778_at | RBMS2 | 1.29E-14 | 3.61E-16 | -0.85 | 1.88E-19 | 9.44E-22 | -0.81 | 6.62E-07 | 4.36E-08 | -0.75 |
| 213891_s_at | TCF4 | 4.17E-10 | 3.10E-11 | -0.85 | 4.33E-09 | 2.50E-10 | -0.87 | 9.66E-08 | 4.87E-09 | -0.90 |
| 235759_at |  | 5.31E-10 | 4.03E-11 | -0.85 | 2.86E-11 | 1.01E-12 | -1.16 | 2.45E-09 | 7.73E-11 | -1.11 |
| 218610_s_at | CPPED1 | 1.30E-15 | 2.86E-17 | -0.85 | 6.77E-08 | 5.06E-09 | -0.81 | 6.94E-06 | 6.35E-07 | -0.71 |
| 57588_at | SLC24A3 | 5.64E-08 | 6.46E-09 | -0.85 | 1.19E-06 | 1.19E-07 | -0.72 | 1.55E-05 | 1.57E-06 | -0.87 |
| 205304_s_at | KCNJ8 | 2.68E-09 | 2.35E-10 | -0.84 | 7.00E-18 | 5.42E-20 | -1.23 | 6.85E-12 | 9.44E-14 | -1.50 |
| 225919_s_at | C9orf72 | 3.34E-12 | 1.59E-13 | -0.84 | 1.87E-06 | 1.96E-07 | -0.78 | 3.66E-10 | 8.94E-12 | -0.79 |
| 227503_at |  | 5.65E-10 | 4.30E-11 | -0.84 | 1.82E-06 | 1.91E-07 | -0.74 | 4.19E-05 | 4.88E-06 | -0.97 |
| 243917_at | CLIC5 | 2.57E-20 | 1.30E-22 | -0.84 | 6.45E-18 | 4.92E-20 | -0.78 | 1.18E-05 | 1.15E-06 | -1.98 |
| 236456_at | PTPN5 | 2.93E-11 | 1.71E-12 | -0.84 | 2.46E-09 | 1.35E-10 | -0.82 | 3.64E-14 | 2.44E-16 | -2.61 |
| 230673_at | PKHD1L1 | 4.65E-06 | 7.82E-07 | -0.84 | 8.12E-08 | 6.18E-09 | -0.83 | 2.68E-06 | 2.14E-07 | -2.29 |
| 236081_at | SNCA | 1.07E-14 | 2.91E-16 | -0.84 | 7.32E-21 | 2.55E-23 | -1.06 | 1.84E-15 | 8.38E-18 | -2.09 |
| 218486_at | KLF11 | 4.00E-11 | 2.39E-12 | -0.83 | 4.19E-13 | 9.84E-15 | -0.89 | 7.52E-14 | 5.54E-16 | -0.95 |
| 231980_at | DOK6 | 1.08E-10 | 7.03E-12 | -0.83 | 8.66E-15 | 1.40E-16 | -1.26 | 3.47E-11 | 6.12E-13 | -1.54 |
| 233882_s_at | SEMA6D | 1.04E-08 | 1.03E-09 | -0.83 | 1.64E-10 | 6.96E-12 | -0.72 | 1.17E-09 | 3.36E-11 | -1.61 |
| 1557961_s_at | C8orf88 | 1.62E-08 | 1.66E-09 | -0.83 | 2.03E-11 | 6.96E-13 | -1.09 | 6.07E-11 | 1.15E-12 | -1.54 |
| 215222_x_at | MACF1 | 2.48E-11 | 1.43E-12 | -0.83 | 1.28E-11 | 4.21E-13 | -0.76 | 9.97E-11 | 2.03E-12 | -0.83 |
| 211813_x_at | DCN | 7.20E-07 | 1.02E-07 | -0.83 | 1.08E-06 | 1.07E-07 | -0.86 | 8.28E-05 | 1.06E-05 | -0.90 |
| 206747_at | GPRIN2 | 7.48E-14 | 2.48E-15 | -0.82 | 9.16E-12 | 2.90E-13 | -0.78 | 1.19E-08 | 4.55E-10 | -1.10 |
| 45288_at | ABHD6 | 1.17E-10 | 7.64E-12 | -0.82 | 7.93E-13 | 1.95E-14 | -0.80 | 4.20E-15 | 2.10E-17 | -1.31 |
| 235764_at | PRDM5 | 1.95E-12 | 8.85E-14 | -0.82 | 2.16E-12 | 5.90E-14 | -0.79 | 6.80E-09 | 2.44E-10 | -1.12 |
| 221541_at | CRISPLD2 | 5.41E-07 | 7.52E-08 | -0.82 | 4.85E-06 | 5.63E-07 | -1.02 | 1.07E-08 | 4.03E-10 | -1.10 |
| 204904_at | GJA4 | 7.16E-11 | 4.50E-12 | -0.82 | 8.91E-15 | 1.44E-16 | -0.88 | 2.28E-12 | 2.57E-14 | -1.44 |
| 1554789_a_at | PDE8B | 9.01E-06 | 1.61E-06 | -0.82 | 1.46E-10 | 6.12E-12 | -1.26 | 5.35E-10 | 1.37E-11 | -1.37 |
| 225167_at | FRMD4A | 1.15E-11 | 6.19E-13 | -0.82 | 1.35E-11 | 4.45E-13 | -0.82 | 3.21E-11 | 5.61E-13 | -0.83 |
| 203661_s_at | TMOD1 | 6.29E-12 | 3.22E-13 | -0.82 | 3.31E-16 | 3.85E-18 | -0.83 | 1.67E-09 | 5.02E-11 | -1.06 |
| 212387_at | TCF4 | 1.33E-10 | 8.84E-12 | -0.82 | 1.47E-08 | 9.46E-10 | -0.94 | 2.55E-09 | 8.11E-11 | -1.00 |
| 204742_s_at | PDS5B | 1.79E-13 | 6.47E-15 | -0.82 | 2.20E-09 | 1.19E-10 | -0.82 | 3.66E-12 | 4.54E-14 | -0.93 |
| 211776_s_at | EPB41L3 | 3.91E-06 | 6.47E-07 | -0.82 | 4.34E-08 | 3.09E-09 | -1.13 | 8.41E-08 | 4.17E-09 | -0.95 |
| 203542_s_at | KLF9 | 4.72E-05 | 9.90E-06 | -0.82 | 3.20E-13 | 7.25E-15 | -1.43 | 2.64E-13 | 2.24E-15 | -1.46 |
| 242828_at | FIGN | 1.80E-06 | 2.78E-07 | -0.82 | 6.61E-06 | 7.96E-07 | -0.78 | 3.68E-10 | 9.02E-12 | -1.28 |
| 206995_x_at | SCARF1 | 5.21E-15 | 1.32E-16 | -0.81 | 1.10E-10 | 4.48E-12 | -0.84 | 5.06E-06 | 4.41E-07 | -0.89 |
| 204395_s_at | GRK5 | 5.82E-08 | 6.69E-09 | -0.81 | 3.17E-12 | 9.00E-14 | -1.25 | 3.11E-11 | 5.42E-13 | -1.69 |
| 203810_at | DNAJB4 | 3.53E-09 | 3.19E-10 | -0.81 | 1.60E-13 | 3.36E-15 | -1.30 | 2.58E-12 | 2.99E-14 | -1.18 |
| 238488_at | LRRC70///IPO11 | 1.21E-08 | 1.21E-09 | -0.81 | 1.72E-08 | 1.13E-09 | -1.17 | 4.85E-05 | 5.75E-06 | -1.14 |
| 210640_s_at | GPER1 | 5.93E-08 | 6.83E-09 | -0.81 | 8.10E-12 | 2.54E-13 | -0.75 | 5.00E-14 | 3.47E-16 | -2.14 |
| 205782_at | FGF7 | 6.89E-09 | 6.58E-10 | -0.80 | 3.32E-05 | 4.74E-06 | -1.01 | 9.71E-05 | 1.27E-05 | -1.01 |
| 229116_at | CNKSR2 | 1.53E-11 | 8.41E-13 | -0.80 | 3.90E-11 | 1.43E-12 | -1.12 | 5.76E-05 | 6.98E-06 | -1.23 |
| 207075_at | NLRP3 | 6.23E-06 | 1.08E-06 | -0.80 | 5.13E-06 | 6.00E-07 | -0.75 | 3.83E-06 | 3.22E-07 | -0.76 |
| 212923_s_at | PXDC1 | 3.81E-12 | 1.84E-13 | -0.80 | 1.51E-13 | 3.13E-15 | -0.90 | 1.19E-11 | 1.84E-13 | -0.90 |
| 212451_at | SECISBP2L | 3.64E-05 | 7.45E-06 | -0.80 | 5.44E-06 | 6.42E-07 | -0.87 | 6.79E-08 | 3.28E-09 | -0.75 |
| 229121_at | CMKLR1 | 1.17E-07 | 1.42E-08 | -0.80 | 1.98E-08 | 1.32E-09 | -0.74 | 5.64E-06 | 5.00E-07 | -1.18 |
| 235051_at | CCDC50 | 7.71E-11 | 4.87E-12 | -0.80 | 2.41E-13 | 5.34E-15 | -1.18 | 1.50E-10 | 3.22E-12 | -0.94 |
| 45749_at | FAM65A | 4.83E-16 | 9.40E-18 | -0.80 | 1.16E-17 | 9.52E-20 | -0.77 | 4.32E-11 | 7.78E-13 | -0.85 |
| 207330_at | PZP | 9.74E-12 | 5.18E-13 | -0.80 | 3.87E-13 | 8.97E-15 | -0.75 | 8.07E-08 | 3.98E-09 | -1.40 |
| 228693_at | CCDC50 | 1.61E-07 | 2.01E-08 | -0.80 | 1.78E-09 | 9.49E-11 | -1.18 | 4.83E-10 | 1.23E-11 | -1.12 |
| 240214_at | RWDD1 | 7.56E-13 | 3.17E-14 | -0.80 | 9.92E-10 | 5.00E-11 | -0.78 | 3.17E-10 | 7.57E-12 | -0.80 |
| 209209_s_at | FERMT2 | 1.42E-05 | 2.65E-06 | -0.80 | 3.20E-07 | 2.78E-08 | -1.31 | 4.05E-12 | 5.16E-14 | -0.97 |
| 213094_at | GPR126 | 7.54E-06 | 1.32E-06 | -0.80 | 2.17E-08 | 1.46E-09 | -1.25 | 8.97E-08 | 4.49E-09 | -1.35 |
| 1561754_at |  | 1.29E-07 | 1.58E-08 | -0.80 | 1.68E-11 | 5.65E-13 | -0.76 | 7.67E-09 | 2.77E-10 | -2.58 |
| 202794_at | INPP1 | 9.24E-13 | 3.94E-14 | -0.79 | 1.82E-11 | 6.15E-13 | -0.80 | 1.19E-08 | 4.56E-10 | -0.81 |
| 232204_at | EBF1 | 9.39E-08 | 1.12E-08 | -0.79 | 2.40E-11 | 8.37E-13 | -1.07 | 2.11E-09 | 6.54E-11 | -1.47 |
| 222033_s_at | FLT1 | 9.62E-10 | 7.69E-11 | -0.79 | 2.75E-12 | 7.65E-14 | -0.97 | 1.01E-14 | 5.69E-17 | -1.31 |
| 1552315_at | GIMAP1 | 9.68E-10 | 7.75E-11 | -0.79 | 1.14E-11 | 3.68E-13 | -0.97 | 1.12E-05 | 1.09E-06 | -0.77 |
| 226342_at | SPTBN1 | 1.56E-10 | 1.05E-11 | -0.79 | 3.80E-14 | 7.05E-16 | -1.17 | 1.67E-07 | 9.00E-09 | -0.88 |
| 223075_s_at | AIF1L | 6.16E-06 | 1.06E-06 | -0.79 | 7.85E-11 | 3.10E-12 | -1.07 | 1.07E-05 | 1.03E-06 | -1.10 |
| 213386_at | TMEM246 | 9.82E-09 | 9.63E-10 | -0.79 | 1.99E-11 | 6.81E-13 | -1.05 | 4.44E-08 | 2.03E-09 | -1.37 |
| 202133_at | WWTR1 | 1.23E-11 | 6.66E-13 | -0.79 | 1.25E-11 | 4.07E-13 | -0.87 | 5.35E-09 | 1.87E-10 | -0.71 |
| 213183_s_at | CDKN1C | 2.01E-05 | 3.88E-06 | -0.79 | 3.13E-06 | 3.45E-07 | -0.87 | 2.53E-06 | 2.01E-07 | -0.71 |
| 241679_at |  | 4.71E-06 | 7.93E-07 | -0.79 | 3.71E-06 | 4.17E-07 | -0.74 | 3.12E-12 | 3.76E-14 | -2.06 |
| 209789_at | CORO2B | 6.07E-09 | 5.74E-10 | -0.79 | 1.26E-23 | 2.05E-26 | -1.57 | 4.84E-10 | 1.23E-11 | -1.85 |
| 226795_at | LRCH1 | 4.65E-13 | 1.86E-14 | -0.78 | 2.88E-11 | 1.02E-12 | -0.88 | 7.77E-12 | 1.09E-13 | -0.87 |
| 241986_at | BMPER | 2.54E-12 | 1.18E-13 | -0.78 | 4.53E-20 | 1.90E-22 | -1.33 | 1.09E-11 | 1.64E-13 | -2.74 |
| 232095_at | LOC100509683 | 1.02E-09 | 8.22E-11 | -0.78 | 2.37E-13 | 5.21E-15 | -1.11 | 1.04E-15 | 4.38E-18 | -1.07 |
| 209087_x_at | MCAM | 1.95E-07 | 2.48E-08 | -0.78 | 4.34E-07 | 3.88E-08 | -0.81 | 2.42E-14 | 1.54E-16 | -1.56 |
| 201566_x_at | ID2 | 2.29E-09 | 1.97E-10 | -0.78 | 9.98E-10 | 5.04E-11 | -0.83 | 3.14E-10 | 7.47E-12 | -1.28 |
| 229125_at | KANK4 | 1.37E-05 | 2.55E-06 | -0.78 | 2.64E-07 | 2.25E-08 | -0.97 | 1.05E-05 | 1.02E-06 | -1.66 |
| 220016_at | AHNAK | 1.13E-08 | 1.12E-09 | -0.78 | 2.41E-13 | 5.33E-15 | -0.85 | 1.30E-05 | 1.29E-06 | -0.91 |
| 236548_at | GIPC2 | 9.48E-07 | 1.38E-07 | -0.78 | 8.98E-07 | 8.70E-08 | -1.01 | 3.06E-07 | 1.81E-08 | -1.63 |
| 1552715_a_at | RXFP1 | 1.61E-08 | 1.65E-09 | -0.78 | 6.47E-14 | 1.26E-15 | -1.44 | 2.96E-07 | 1.74E-08 | -1.97 |
| 224833_at | ETS1 | 1.13E-11 | 6.04E-13 | -0.78 | 4.34E-14 | 8.18E-16 | -1.01 | 2.94E-10 | 6.89E-12 | -0.77 |
| 1560879_a_at | SYT15 | 5.25E-16 | 1.04E-17 | -0.77 | 2.27E-17 | 1.99E-19 | -0.85 | 2.87E-07 | 1.68E-08 | -1.12 |
| 230081_at | PLCXD3 | 1.90E-08 | 1.97E-09 | -0.77 | 2.21E-14 | 3.91E-16 | -1.33 | 6.57E-07 | 4.33E-08 | -2.04 |
| 230403_at | RFX3 | 1.07E-05 | 1.94E-06 | -0.77 | 1.94E-07 | 1.60E-08 | -0.70 | 2.76E-05 | 3.02E-06 | -0.86 |
| 244422_at |  | 1.54E-09 | 1.28E-10 | -0.77 | 4.93E-19 | 2.73E-21 | -1.38 | 2.31E-07 | 1.30E-08 | -1.54 |
| 214920_at | THSD7A | 5.32E-05 | 1.13E-05 | -0.77 | 4.37E-08 | 3.12E-09 | -1.03 | 3.02E-06 | 2.46E-07 | -1.09 |
| 222146_s_at | TCF4 | 8.38E-09 | 8.11E-10 | -0.77 | 4.72E-07 | 4.26E-08 | -0.80 | 3.79E-10 | 9.32E-12 | -1.15 |
| 204688_at | SGCE | 9.24E-07 | 1.34E-07 | -0.77 | 1.21E-09 | 6.21E-11 | -1.17 | 1.42E-06 | 1.04E-07 | -1.04 |
| 208633_s_at | MACF1 | 6.74E-08 | 7.85E-09 | -0.77 | 2.27E-08 | 1.53E-09 | -0.78 | 2.70E-11 | 4.62E-13 | -0.97 |
| 226828_s_at | HEYL | 2.45E-07 | 3.18E-08 | -0.77 | 7.39E-09 | 4.49E-10 | -0.75 | 1.01E-14 | 5.67E-17 | -1.24 |
| 227326_at | MXRA7 | 9.93E-10 | 7.96E-11 | -0.77 | 4.53E-11 | 1.68E-12 | -0.76 | 7.08E-08 | 3.43E-09 | -1.00 |
| 229497_at | ANKDD1A | 7.30E-11 | 4.60E-12 | -0.76 | 2.64E-10 | 1.17E-11 | -0.82 | 1.77E-10 | 3.87E-12 | -1.38 |
| 230351_at | FGF14-AS2 | 7.05E-13 | 2.93E-14 | -0.76 | 4.03E-10 | 1.86E-11 | -0.81 | 7.76E-10 | 2.10E-11 | -1.39 |
| 228837_at | TCF4 | 3.48E-09 | 3.13E-10 | -0.76 | 2.74E-08 | 1.88E-09 | -0.97 | 3.32E-12 | 4.05E-14 | -1.09 |
| 228185_at | ZNF25 | 3.77E-13 | 1.47E-14 | -0.76 | 1.23E-10 | 5.07E-12 | -0.71 | 4.09E-10 | 1.02E-11 | -0.76 |
| 212698_s_at | SEPT10 | 4.05E-08 | 4.49E-09 | -0.76 | 8.27E-10 | 4.08E-11 | -0.95 | 1.39E-11 | 2.20E-13 | -0.82 |
| 232378_at | SLC5A9 | 3.32E-06 | 5.41E-07 | -0.76 | 3.02E-17 | 2.70E-19 | -1.34 | 3.49E-08 | 1.54E-09 | -2.87 |
| 205440_s_at | NPY1R | 5.38E-05 | 1.14E-05 | -0.76 | 6.21E-09 | 3.72E-10 | -1.14 | 7.99E-06 | 7.44E-07 | -1.90 |
| 212099_at | RHOB | 5.12E-06 | 8.69E-07 | -0.76 | 4.93E-11 | 1.85E-12 | -1.08 | 8.78E-05 | 1.13E-05 | -0.86 |
| 229084_at | CNTN4 | 1.83E-06 | 2.82E-07 | -0.76 | 6.86E-09 | 4.15E-10 | -0.90 | 2.87E-05 | 3.16E-06 | -0.89 |
| 213029_at | NFIB | 7.40E-07 | 1.05E-07 | -0.76 | 5.88E-11 | 2.24E-12 | -0.75 | 2.88E-05 | 3.18E-06 | -0.72 |
| 212647_at | RRAS | 2.00E-09 | 1.70E-10 | -0.76 | 5.04E-18 | 3.73E-20 | -1.11 | 1.33E-09 | 3.86E-11 | -0.86 |
| 239146_at | CLDND1 | 2.85E-14 | 8.62E-16 | -0.76 | 1.38E-10 | 5.77E-12 | -1.00 | 4.93E-07 | 3.13E-08 | -0.86 |
| 223279_s_at | UACA | 2.31E-09 | 1.99E-10 | -0.76 | 6.27E-12 | 1.92E-13 | -0.91 | 2.21E-08 | 9.20E-10 | -0.95 |
| 1565577_s_at |  | 2.70E-10 | 1.93E-11 | -0.76 | 6.65E-08 | 4.97E-09 | -0.71 | 2.74E-09 | 8.81E-11 | -0.84 |
| 220676_at | ADAMTS8 | 3.88E-13 | 1.52E-14 | -0.75 | 3.72E-16 | 4.39E-18 | -0.75 | 6.33E-06 | 5.72E-07 | -1.34 |
| 202422_s_at | ACSL4 | 2.62E-07 | 3.42E-08 | -0.75 | 2.12E-06 | 2.25E-07 | -0.84 | 1.62E-05 | 1.65E-06 | -0.72 |
| 221675_s_at | CHPT1 | 5.99E-10 | 4.58E-11 | -0.75 | 7.50E-10 | 3.67E-11 | -0.80 | 3.09E-05 | 3.44E-06 | -0.76 |
| 223380_s_at | LATS2 | 4.14E-13 | 1.63E-14 | -0.75 | 1.05E-08 | 6.57E-10 | -0.79 | 2.95E-14 | 1.95E-16 | -1.08 |
| 236996_at |  | 8.88E-12 | 4.69E-13 | -0.75 | 1.87E-14 | 3.24E-16 | -0.95 | 2.85E-09 | 9.22E-11 | -1.47 |
| 210155_at | MYOC | 6.44E-06 | 1.12E-06 | -0.75 | 3.65E-16 | 4.29E-18 | -1.07 | 4.26E-17 | 1.08E-19 | -1.79 |
| 209496_at | RARRES2 | 1.20E-05 | 2.20E-06 | -0.74 | 1.12E-11 | 3.63E-13 | -1.14 | 9.76E-05 | 1.28E-05 | -0.88 |
| 229163_at | CAMK2N1 | 7.82E-05 | 1.73E-05 | -0.74 | 1.35E-05 | 1.74E-06 | -0.79 | 1.49E-06 | 1.10E-07 | -1.47 |
| 232205_at |  | 1.46E-07 | 1.81E-08 | -0.74 | 4.46E-07 | 4.01E-08 | -0.90 | 1.00E-05 | 9.60E-07 | -0.74 |
| 244825_at | SHROOM4 | 1.06E-08 | 1.04E-09 | -0.74 | 2.33E-09 | 1.27E-10 | -0.80 | 4.61E-06 | 3.96E-07 | -1.14 |
| 236290_at | DOK6 | 8.67E-10 | 6.85E-11 | -0.74 | 5.44E-16 | 6.78E-18 | -0.94 | 1.38E-09 | 4.04E-11 | -1.68 |
| 227911_at | ARHGAP28 | 3.70E-05 | 7.58E-06 | -0.74 | 1.54E-05 | 2.02E-06 | -0.74 | 1.44E-06 | 1.06E-07 | -1.10 |
| 216336_x_at | LOC100505584///MT1E | 1.87E-05 | 3.59E-06 | -0.74 | 1.23E-05 | 1.57E-06 | -0.71 | 2.82E-08 | 1.21E-09 | -1.09 |
| 212262_at | QKI | 2.55E-12 | 1.19E-13 | -0.74 | 7.19E-10 | 3.50E-11 | -0.97 | 1.70E-10 | 3.69E-12 | -0.78 |
| 225662_at | ZAK | 5.95E-06 | 1.02E-06 | -0.74 | 7.05E-10 | 3.43E-11 | -0.79 | 5.42E-08 | 2.54E-09 | -0.82 |
| 202724_s_at | FOXO1 | 3.50E-10 | 2.55E-11 | -0.74 | 2.97E-09 | 1.66E-10 | -0.87 | 2.57E-08 | 1.09E-09 | -0.83 |
| 239345_at | SLC19A3 | 5.61E-12 | 2.83E-13 | -0.74 | 1.17E-19 | 5.44E-22 | -1.36 | 1.14E-12 | 1.16E-14 | -2.06 |
| 225665_at | ZAK | 3.14E-09 | 2.80E-10 | -0.74 | 1.44E-07 | 1.16E-08 | -0.81 | 1.80E-08 | 7.27E-10 | -0.82 |
| 1558103_a_at |  | 1.50E-07 | 1.86E-08 | -0.73 | 1.88E-10 | 8.06E-12 | -0.84 | 4.03E-09 | 1.37E-10 | -1.23 |
| 1558404_at | LINC00622 | 2.79E-09 | 2.45E-10 | -0.73 | 1.21E-11 | 3.94E-13 | -1.19 | 1.75E-16 | 5.68E-19 | -1.94 |
| 219046_s_at | PKNOX2 | 5.30E-14 | 1.69E-15 | -0.73 | 3.46E-12 | 9.94E-14 | -0.73 | 2.90E-10 | 6.77E-12 | -1.51 |
| 211458_s_at | GABARAPL3///GABARAPL1 | 1.62E-11 | 9.00E-13 | -0.73 | 4.60E-17 | 4.35E-19 | -1.02 | 6.67E-12 | 9.16E-14 | -1.02 |
| 224663_s_at | CFL2 | 7.36E-08 | 8.64E-09 | -0.73 | 4.57E-11 | 1.70E-12 | -1.04 | 2.35E-08 | 9.87E-10 | -0.88 |
| 219228_at | ZNF331 | 9.98E-07 | 1.46E-07 | -0.72 | 1.15E-10 | 4.73E-12 | -1.18 | 8.46E-11 | 1.67E-12 | -1.44 |
| 202073_at | OPTN | 2.50E-10 | 1.77E-11 | -0.72 | 1.47E-10 | 6.18E-12 | -0.86 | 5.86E-08 | 2.78E-09 | -0.73 |
| 227283_at | EFR3B | 3.34E-11 | 1.97E-12 | -0.72 | 1.97E-10 | 8.53E-12 | -0.70 | 1.06E-06 | 7.46E-08 | -1.04 |
| 214660_at | ITGA1 | 1.33E-06 | 2.00E-07 | -0.72 | 6.23E-06 | 7.45E-07 | -0.86 | 3.08E-10 | 7.28E-12 | -1.11 |
| 221552_at | ABHD6 | 2.61E-10 | 1.86E-11 | -0.72 | 1.59E-15 | 2.19E-17 | -0.78 | 1.43E-16 | 4.41E-19 | -1.39 |
| 1553844_a_at | C10orf67 | 2.24E-13 | 8.29E-15 | -0.71 | 7.24E-21 | 2.50E-23 | -0.97 | 2.14E-17 | 4.94E-20 | -2.68 |
| 238694_at | DGKE | 1.97E-08 | 2.05E-09 | -0.71 | 1.52E-13 | 3.17E-15 | -0.92 | 4.11E-10 | 1.03E-11 | -1.06 |
| 1558102_at |  | 1.29E-06 | 1.93E-07 | -0.71 | 9.06E-09 | 5.61E-10 | -0.90 | 5.57E-07 | 3.59E-08 | -0.95 |
| 224458_at | TMEM246 | 2.19E-06 | 3.44E-07 | -0.71 | 7.99E-08 | 6.07E-09 | -0.88 | 4.56E-05 | 5.37E-06 | -1.30 |
| 41577_at | PPP1R16B | 3.04E-07 | 4.01E-08 | -0.71 | 2.28E-14 | 4.03E-16 | -1.21 | 6.36E-07 | 4.16E-08 | -1.22 |
| 225380_at | PKDCC | 2.61E-08 | 2.79E-09 | -0.71 | 1.21E-12 | 3.13E-14 | -0.92 | 6.51E-05 | 8.05E-06 | -1.13 |
| 1569136_at | MGAT4A | 5.47E-08 | 6.24E-09 | -0.71 | 1.82E-13 | 3.88E-15 | -0.92 | 9.93E-08 | 5.03E-09 | -1.03 |
| 222762_x_at | LIMD1 | 4.76E-08 | 5.35E-09 | -0.71 | 1.01E-14 | 1.64E-16 | -0.85 | 3.59E-10 | 8.74E-12 | -1.16 |
| 212386_at | TCF4 | 1.73E-08 | 1.78E-09 | -0.71 | 1.82E-10 | 7.78E-12 | -0.95 | 4.70E-07 | 2.96E-08 | -0.74 |
| 211340_s_at | MCAM | 1.14E-06 | 1.69E-07 | -0.71 | 6.85E-07 | 6.48E-08 | -0.86 | 1.47E-13 | 1.19E-15 | -1.60 |
| 210090_at | ARC | 1.74E-06 | 2.68E-07 | -0.71 | 3.06E-15 | 4.47E-17 | -1.34 | 1.71E-08 | 6.87E-10 | -2.62 |
| 226080_at | SSH2 | 8.73E-14 | 2.95E-15 | -0.71 | 2.51E-15 | 3.59E-17 | -0.77 | 1.04E-15 | 4.41E-18 | -0.77 |
| 214212_x_at | FERMT2 | 5.93E-07 | 8.29E-08 | -0.70 | 6.35E-12 | 1.94E-13 | -0.94 | 6.27E-12 | 8.50E-14 | -0.98 |
| 243806_at |  | 8.17E-07 | 1.17E-07 | -0.70 | 7.64E-12 | 2.39E-13 | -0.74 | 3.77E-11 | 6.73E-13 | -2.54 |
| 209293_x_at | ID4 | 1.59E-07 | 1.98E-08 | -0.70 | 6.09E-09 | 3.64E-10 | -0.76 | 3.02E-09 | 9.89E-11 | -1.04 |
| 203753_at | TCF4 | 5.52E-08 | 6.31E-09 | -0.70 | 8.56E-07 | 8.26E-08 | -0.82 | 6.49E-07 | 4.26E-08 | -0.83 |
| 226565_at | TMEM99 | 1.53E-08 | 1.56E-09 | 0.70 | 6.67E-13 | 1.62E-14 | 0.93 | 4.98E-09 | 1.72E-10 | 1.03 |
| 223249_at | CLDN12 | 3.55E-09 | 3.22E-10 | 0.71 | 1.06E-11 | 3.41E-13 | 0.81 | 1.12E-06 | 8.01E-08 | 0.80 |
| 218756_s_at | DHRS11 | 2.72E-09 | 2.39E-10 | 0.71 | 1.03E-08 | 6.43E-10 | 0.72 | 2.36E-06 | 1.86E-07 | 0.78 |
| 223037_at | PDZD11 | 1.12E-17 | 1.38E-19 | 0.71 | 6.53E-19 | 3.83E-21 | 0.70 | 3.55E-10 | 8.63E-12 | 0.84 |
| 221923_s_at | NPM1 | 1.72E-12 | 7.74E-14 | 0.71 | 2.16E-12 | 5.91E-14 | 0.75 | 3.18E-12 | 3.85E-14 | 0.94 |
| 223245_at | STRBP | 1.45E-09 | 1.20E-10 | 0.71 | 1.32E-13 | 2.73E-15 | 0.80 | 6.55E-13 | 6.27E-15 | 0.84 |
| 213164_at | SLC5A3 | 5.84E-08 | 6.72E-09 | 0.72 | 3.28E-11 | 1.18E-12 | 1.01 | 2.79E-08 | 1.20E-09 | 0.83 |
| 205286_at | TFAP2C | 3.52E-05 | 7.18E-06 | 0.72 | 4.43E-08 | 3.17E-09 | 0.92 | 5.42E-08 | 2.54E-09 | 1.29 |
| 1554438_at | KIAA1217 | 2.28E-17 | 3.10E-19 | 0.73 | 6.67E-13 | 1.62E-14 | 0.92 | 2.32E-19 | 2.42E-22 | 1.25 |
| 214106_s_at | GMDS | 3.78E-09 | 3.44E-10 | 0.73 | 1.89E-08 | 1.25E-09 | 0.88 | 1.32E-05 | 1.31E-06 | 0.94 |
| 230682_x_at | ABCC3 | 9.67E-08 | 1.16E-08 | 0.73 | 4.36E-08 | 3.11E-09 | 0.98 | 3.68E-13 | 3.32E-15 | 2.04 |
| 222029_x_at | PFDN6 | 2.71E-13 | 1.02E-14 | 0.73 | 2.36E-17 | 2.08E-19 | 0.78 | 4.34E-09 | 1.48E-10 | 0.71 |
| 214404_x_at | SPDEF | 1.86E-07 | 2.36E-08 | 0.73 | 8.87E-11 | 3.54E-12 | 0.81 | 4.71E-12 | 6.13E-14 | 1.75 |
| 209626_s_at | OSBPL3 | 2.75E-06 | 4.42E-07 | 0.74 | 8.09E-07 | 7.76E-08 | 0.71 | 6.30E-08 | 3.01E-09 | 1.03 |
| 224444_s_at | LINC00467 | 1.28E-06 | 1.92E-07 | 0.74 | 4.76E-12 | 1.41E-13 | 1.10 | 6.72E-13 | 6.48E-15 | 1.11 |
| 226276_at | TMEM167A | 9.28E-11 | 5.97E-12 | 0.74 | 1.66E-07 | 1.35E-08 | 0.73 | 4.60E-09 | 1.58E-10 | 0.86 |
| 207264_at | KDELR3 | 2.29E-08 | 2.42E-09 | 0.74 | 3.73E-15 | 5.56E-17 | 0.80 | 1.39E-14 | 8.22E-17 | 2.03 |
| 212729_at | DLG3 | 1.94E-15 | 4.49E-17 | 0.74 | 2.09E-10 | 9.10E-12 | 0.82 | 5.07E-10 | 1.30E-11 | 0.88 |
| 226129_at | FAM83H | 1.86E-06 | 2.88E-07 | 0.74 | 2.13E-07 | 1.77E-08 | 0.72 | 1.58E-06 | 1.17E-07 | 0.95 |
| 206482_at | PTK6 | 5.82E-06 | 1.00E-06 | 0.74 | 7.14E-09 | 4.33E-10 | 0.82 | 1.41E-05 | 1.41E-06 | 1.23 |
| 201231_s_at | ENO1 | 1.28E-10 | 8.44E-12 | 0.74 | 2.69E-16 | 3.08E-18 | 0.96 | 3.05E-10 | 7.20E-12 | 0.94 |
| 240616_at | LOC100507636 | 5.73E-10 | 4.37E-11 | 0.74 | 1.77E-15 | 2.46E-17 | 1.31 | 2.14E-10 | 4.78E-12 | 1.05 |
| 1558508_a_at | C1orf53 | 2.41E-05 | 4.73E-06 | 0.74 | 2.00E-17 | 1.74E-19 | 1.86 | 2.19E-11 | 3.65E-13 | 1.60 |
| 218800_at | SRD5A3 | 6.29E-05 | 1.36E-05 | 0.74 | 5.97E-06 | 7.12E-07 | 0.77 | 2.51E-06 | 2.00E-07 | 1.02 |
| 218681_s_at | SDF2L1 | 3.85E-08 | 4.26E-09 | 0.74 | 9.29E-14 | 1.87E-15 | 0.81 | 2.44E-06 | 1.93E-07 | 0.85 |
| 223246_s_at | STRBP | 1.07E-14 | 2.92E-16 | 0.74 | 5.11E-12 | 1.53E-13 | 0.70 | 2.05E-08 | 8.43E-10 | 0.75 |
| 216641_s_at | LAD1 | 1.02E-09 | 8.19E-11 | 0.74 | 1.51E-10 | 6.33E-12 | 1.03 | 2.26E-11 | 3.80E-13 | 1.57 |
| 218321_x_at | STYXL1 | 1.73E-12 | 7.80E-14 | 0.75 | 4.54E-13 | 1.07E-14 | 0.78 | 4.25E-09 | 1.45E-10 | 0.87 |
| 200903_s_at | AHCY | 1.65E-12 | 7.38E-14 | 0.75 | 9.54E-17 | 9.73E-19 | 0.72 | 3.53E-11 | 6.25E-13 | 0.85 |
| 232602_at | WFDC3 | 2.31E-09 | 1.99E-10 | 0.75 | 3.78E-06 | 4.27E-07 | 0.95 | 7.70E-08 | 3.77E-09 | 2.45 |
| 204476_s_at | PC | 1.34E-11 | 7.31E-13 | 0.75 | 7.63E-13 | 1.88E-14 | 0.97 | 1.28E-10 | 2.68E-12 | 1.44 |
| 209507_at | RPA3 | 1.94E-11 | 1.09E-12 | 0.75 | 1.81E-09 | 9.67E-11 | 0.70 | 7.07E-07 | 4.72E-08 | 0.83 |
| 202220_at | KIAA0907 | 1.49E-11 | 8.18E-13 | 0.75 | 1.65E-08 | 1.07E-09 | 0.76 | 2.70E-11 | 4.61E-13 | 0.72 |
| 200698_at | KDELR2 | 1.53E-13 | 5.40E-15 | 0.75 | 7.42E-11 | 2.90E-12 | 0.81 | 2.54E-09 | 8.07E-11 | 0.81 |
| 221734_at | PRRC1 | 1.05E-10 | 6.83E-12 | 0.76 | 1.93E-13 | 4.14E-15 | 0.77 | 4.16E-14 | 2.86E-16 | 0.81 |
| 240861_at |  | 8.09E-05 | 1.79E-05 | 0.76 | 6.28E-07 | 5.89E-08 | 1.20 | 1.26E-05 | 1.24E-06 | 1.10 |
| 222723_at | VWA1 | 1.83E-09 | 1.55E-10 | 0.76 | 9.91E-12 | 3.17E-13 | 0.76 | 2.92E-05 | 3.23E-06 | 0.86 |
| 229271_x_at | COL11A1 | 2.36E-07 | 3.06E-08 | 0.76 | 2.35E-09 | 1.28E-10 | 1.61 | 7.02E-07 | 4.67E-08 | 3.96 |
| 218704_at | RNF43 | 6.47E-07 | 9.10E-08 | 0.76 | 1.91E-09 | 1.03E-10 | 0.95 | 8.15E-09 | 2.97E-10 | 1.37 |
| 201341_at | ENC1 | 3.37E-07 | 4.49E-08 | 0.76 | 2.16E-20 | 8.36E-23 | 1.20 | 3.46E-12 | 4.25E-14 | 1.08 |
| 209589_s_at | EPHB2 | 6.30E-09 | 5.98E-10 | 0.76 | 1.12E-10 | 4.56E-12 | 0.78 | 4.48E-08 | 2.05E-09 | 1.51 |
| 204729_s_at | STX1A | 2.46E-15 | 5.80E-17 | 0.76 | 7.47E-11 | 2.93E-12 | 0.78 | 2.11E-08 | 8.67E-10 | 1.23 |
| 223531_x_at | GPR89A///GPR89B | 7.89E-19 | 6.72E-21 | 0.77 | 1.64E-21 | 4.92E-24 | 0.76 | 1.13E-11 | 1.72E-13 | 0.74 |
| 230370_x_at | STYXL1 | 6.28E-12 | 3.21E-13 | 0.77 | 4.81E-12 | 1.43E-13 | 0.81 | 5.82E-11 | 1.10E-12 | 1.07 |
| 225836_s_at | RHNO1 | 2.02E-10 | 1.40E-11 | 0.77 | 7.97E-14 | 1.58E-15 | 0.91 | 4.71E-09 | 1.62E-10 | 0.97 |
| 207732_s_at | DLG3 | 1.93E-15 | 4.44E-17 | 0.77 | 5.10E-08 | 3.70E-09 | 0.80 | 9.07E-23 | 1.99E-26 | 2.39 |
| 210959_s_at | SRD5A1 | 4.70E-09 | 4.35E-10 | 0.77 | 5.86E-06 | 6.96E-07 | 0.92 | 6.01E-09 | 2.13E-10 | 1.20 |
| 233252_s_at | STRBP | 6.23E-16 | 1.26E-17 | 0.77 | 7.98E-14 | 1.59E-15 | 0.81 | 7.99E-08 | 3.93E-09 | 0.72 |
| 209610_s_at | SLC1A4 | 3.71E-06 | 6.12E-07 | 0.77 | 8.44E-19 | 5.03E-21 | 1.11 | 2.70E-06 | 2.16E-07 | 0.73 |
| 220441_at | DNAJC22 | 1.27E-12 | 5.55E-14 | 0.78 | 2.53E-10 | 1.12E-11 | 0.94 | 6.75E-08 | 3.25E-09 | 2.01 |
| 227046_at | SLC39A11 | 2.98E-10 | 2.14E-11 | 0.78 | 4.18E-17 | 3.89E-19 | 1.06 | 1.22E-13 | 9.65E-16 | 1.18 |
| 225792_at | HOOK1 | 1.87E-07 | 2.37E-08 | 0.78 | 4.37E-08 | 3.12E-09 | 1.02 | 1.48E-12 | 1.56E-14 | 1.22 |
| 217809_at | BZW2 | 4.42E-11 | 2.67E-12 | 0.78 | 2.23E-18 | 1.48E-20 | 1.04 | 9.13E-12 | 1.34E-13 | 1.02 |
| 227094_at | DHTKD1 | 3.91E-12 | 1.90E-13 | 0.79 | 6.47E-17 | 6.33E-19 | 0.92 | 7.48E-10 | 2.02E-11 | 0.81 |
| 204667_at | FOXA1 | 3.66E-05 | 7.50E-06 | 0.79 | 7.44E-08 | 5.62E-09 | 0.85 | 7.07E-05 | 8.84E-06 | 1.05 |
| 209326_at | SLC35A2 | 1.50E-12 | 6.62E-14 | 0.79 | 2.79E-14 | 5.04E-16 | 0.85 | 1.09E-07 | 5.58E-09 | 0.83 |
| 208837_at | TMED3 | 4.18E-10 | 3.11E-11 | 0.79 | 2.45E-22 | 5.56E-25 | 1.06 | 6.75E-18 | 1.29E-20 | 1.32 |
| 229866_at | STK32A | 4.51E-09 | 4.15E-10 | 0.79 | 4.02E-15 | 6.05E-17 | 0.99 | 7.93E-12 | 1.12E-13 | 2.14 |
| 1554894_a_at | PCBD2 | 7.27E-13 | 3.04E-14 | 0.79 | 5.14E-10 | 2.43E-11 | 0.70 | 4.60E-13 | 4.26E-15 | 0.97 |
| 217736_s_at | EIF2AK1 | 2.27E-17 | 3.07E-19 | 0.80 | 2.33E-20 | 9.16E-23 | 0.91 | 1.06E-11 | 1.59E-13 | 0.92 |
| 1554050_at | SMPDL3B | 6.50E-16 | 1.32E-17 | 0.80 | 3.52E-10 | 1.60E-11 | 0.71 | 1.06E-11 | 1.58E-13 | 1.20 |
| 229103_at | WNT3 | 4.46E-11 | 2.70E-12 | 0.80 | 5.05E-09 | 2.96E-10 | 0.79 | 3.87E-09 | 1.31E-10 | 1.35 |
| 219463_at | LAMP5 | 2.61E-06 | 4.16E-07 | 0.80 | 2.77E-10 | 1.23E-11 | 1.02 | 8.68E-06 | 8.15E-07 | 1.28 |
| 201644_at | TSTA3 | 8.36E-12 | 4.38E-13 | 0.80 | 7.37E-14 | 1.46E-15 | 0.84 | 5.99E-07 | 3.89E-08 | 0.99 |
| 224917_at | MIR21 | 1.24E-05 | 2.30E-06 | 0.80 | 1.37E-08 | 8.78E-10 | 1.18 | 3.60E-13 | 3.24E-15 | 1.17 |
| 218952_at | PCSK1N | 9.70E-09 | 9.49E-10 | 0.80 | 4.20E-11 | 1.55E-12 | 1.43 | 9.09E-05 | 1.18E-05 | 1.34 |
| 218795_at | ACP6 | 1.34E-09 | 1.10E-10 | 0.80 | 8.78E-14 | 1.76E-15 | 0.91 | 1.14E-09 | 3.23E-11 | 1.00 |
| 224443_at | LINC00467 | 9.65E-08 | 1.16E-08 | 0.80 | 6.35E-16 | 8.05E-18 | 1.17 | 4.53E-12 | 5.85E-14 | 1.30 |
| 1568838_at | WASIR2///WASIR1 | 2.22E-07 | 2.86E-08 | 0.80 | 1.48E-12 | 3.89E-14 | 1.23 | 7.62E-12 | 1.07E-13 | 2.09 |
| 224233_s_at | MSTO2P///MSTO1 | 6.59E-21 | 2.58E-23 | 0.80 | 4.73E-18 | 3.44E-20 | 0.77 | 1.22E-06 | 8.80E-08 | 0.93 |
| 210021_s_at | CCNO | 2.90E-07 | 3.82E-08 | 0.81 | 2.66E-09 | 1.47E-10 | 0.75 | 4.23E-05 | 4.93E-06 | 1.35 |
| 226615_at | XPR1 | 3.03E-06 | 4.91E-07 | 0.81 | 1.95E-07 | 1.61E-08 | 0.82 | 7.82E-07 | 5.28E-08 | 0.94 |
| 222891_s_at | BCL11A | 2.40E-05 | 4.72E-06 | 0.81 | 3.95E-06 | 4.48E-07 | 0.98 | 6.41E-05 | 7.90E-06 | 1.15 |
| 232105_at | LOC100506918 | 9.33E-10 | 7.43E-11 | 0.81 | 2.99E-15 | 4.36E-17 | 1.28 | 1.75E-14 | 1.07E-16 | 2.76 |
| 226132_s_at | MANEAL | 1.89E-07 | 2.39E-08 | 0.81 | 3.17E-18 | 2.20E-20 | 1.43 | 2.18E-10 | 4.89E-12 | 1.37 |
| 232067_at | FAXC | 1.25E-05 | 2.31E-06 | 0.81 | 3.15E-07 | 2.73E-08 | 1.09 | 3.47E-06 | 2.87E-07 | 1.47 |
| 204636_at | COL17A1 | 1.55E-06 | 2.37E-07 | 0.81 | 1.24E-08 | 7.91E-10 | 0.91 | 4.45E-05 | 5.21E-06 | 2.17 |
| 222449_at | PMEPA1 | 5.82E-07 | 8.13E-08 | 0.81 | 2.00E-08 | 1.34E-09 | 0.83 | 4.24E-09 | 1.44E-10 | 1.12 |
| 230789_at | ZNF280B | 2.08E-08 | 2.18E-09 | 0.82 | 1.34E-06 | 1.35E-07 | 0.85 | 1.13E-05 | 1.10E-06 | 1.21 |
| 218493_at | SNRNP25 | 2.05E-14 | 5.97E-16 | 0.82 | 6.48E-17 | 6.35E-19 | 0.81 | 3.14E-13 | 2.74E-15 | 0.95 |
| 210964_s_at | GYG2 | 2.07E-11 | 1.17E-12 | 0.82 | 1.29E-13 | 2.66E-15 | 0.95 | 8.13E-06 | 7.60E-07 | 1.02 |
| 243444_at | SRD5A3 | 3.24E-05 | 6.57E-06 | 0.82 | 1.13E-07 | 8.90E-09 | 0.79 | 1.25E-06 | 9.03E-08 | 0.92 |
| 219768_at | VTCN1 | 6.44E-06 | 1.11E-06 | 0.82 | 1.30E-06 | 1.31E-07 | 0.81 | 9.59E-05 | 1.26E-05 | 1.84 |
| 215465_at | ABCA12 | 6.02E-09 | 5.69E-10 | 0.82 | 1.38E-09 | 7.19E-11 | 1.01 | 2.20E-06 | 1.72E-07 | 2.21 |
| 211165_x_at | EPHB2 | 1.95E-12 | 8.83E-14 | 0.83 | 2.03E-06 | 2.15E-07 | 0.73 | 5.89E-09 | 2.08E-10 | 2.12 |
| 1554930_a_at | FUT8 | 3.02E-06 | 4.88E-07 | 0.83 | 3.01E-07 | 2.60E-08 | 0.99 | 4.25E-07 | 2.63E-08 | 0.82 |
| 204485_s_at | TOM1L1 | 7.39E-08 | 8.67E-09 | 0.83 | 2.38E-06 | 2.56E-07 | 0.78 | 2.28E-09 | 7.14E-11 | 0.98 |
| 201549_x_at | KDM5B | 1.81E-15 | 4.16E-17 | 0.83 | 4.95E-13 | 1.17E-14 | 0.74 | 2.78E-12 | 3.26E-14 | 0.77 |
| 232271_at | HNF4G | 1.96E-08 | 2.05E-09 | 0.83 | 2.22E-09 | 1.21E-10 | 0.99 | 5.10E-15 | 2.60E-17 | 3.25 |
| 213506_at | F2RL1 | 7.05E-06 | 1.23E-06 | 0.83 | 1.01E-14 | 1.66E-16 | 1.49 | 6.75E-10 | 1.80E-11 | 1.40 |
| 200966_x_at | ALDOA | 8.81E-13 | 3.73E-14 | 0.84 | 8.13E-15 | 1.30E-16 | 0.70 | 6.20E-08 | 2.95E-09 | 0.77 |
| 210715_s_at | SPINT2 | 5.45E-12 | 2.74E-13 | 0.84 | 1.29E-10 | 5.35E-12 | 0.73 | 2.36E-10 | 5.35E-12 | 0.97 |
| 209014_at | MAGED1 | 9.72E-17 | 1.55E-18 | 0.84 | 4.06E-17 | 3.76E-19 | 0.93 | 2.18E-12 | 2.43E-14 | 0.72 |
| 229860_x_at | C4orf48 | 3.31E-10 | 2.40E-11 | 0.84 | 8.87E-12 | 2.80E-13 | 1.03 | 5.55E-09 | 1.95E-10 | 1.06 |
| 229599_at | SMIM22 | 1.20E-08 | 1.20E-09 | 0.85 | 3.51E-12 | 1.01E-13 | 1.01 | 8.74E-12 | 1.27E-13 | 2.40 |
| 218982_s_at | ZNF713///MRPS17 | 2.15E-12 | 9.83E-14 | 0.85 | 7.60E-10 | 3.72E-11 | 0.83 | 3.78E-05 | 4.33E-06 | 0.72 |
| 231148_at | IGFL2 | 1.78E-11 | 9.91E-13 | 0.85 | 3.85E-12 | 1.12E-13 | 1.08 | 5.62E-08 | 2.65E-09 | 2.68 |
| 219288_at | C3orf14 | 4.79E-08 | 5.40E-09 | 0.85 | 1.81E-13 | 3.86E-15 | 1.12 | 4.09E-10 | 1.02E-11 | 1.14 |
| 227512_at | MEX3A | 3.14E-16 | 5.87E-18 | 0.85 | 7.76E-17 | 7.75E-19 | 0.74 | 1.24E-10 | 2.61E-12 | 0.96 |
| 227247_at | PLEKHA8 | 4.09E-10 | 3.02E-11 | 0.85 | 2.28E-10 | 9.98E-12 | 0.92 | 4.55E-10 | 1.15E-11 | 0.76 |
| 239657_x_at | FOXO6 | 3.14E-12 | 1.49E-13 | 0.85 | 4.35E-11 | 1.61E-12 | 0.78 | 6.93E-05 | 8.64E-06 | 1.19 |
| 223363_at | PSMG3 | 2.18E-13 | 8.06E-15 | 0.85 | 1.60E-17 | 1.36E-19 | 0.94 | 2.93E-08 | 1.27E-09 | 0.81 |
| 201666_at | TIMP1 | 2.16E-06 | 3.38E-07 | 0.85 | 4.24E-10 | 1.96E-11 | 0.85 | 1.16E-10 | 2.42E-12 | 1.36 |
| 231810_at | BRI3BP | 4.79E-09 | 4.44E-10 | 0.85 | 3.24E-12 | 9.21E-14 | 0.82 | 4.26E-08 | 1.94E-09 | 0.73 |
| 212944_at | SLC5A3 | 8.47E-10 | 6.67E-11 | 0.85 | 3.54E-12 | 1.02E-13 | 1.06 | 8.19E-11 | 1.61E-12 | 0.96 |
| 208051_s_at | PAIP1 | 5.24E-10 | 3.97E-11 | 0.85 | 1.95E-12 | 5.26E-14 | 0.91 | 6.61E-08 | 3.18E-09 | 0.82 |
| 219976_at | HOOK1 | 2.73E-09 | 2.40E-10 | 0.86 | 1.32E-11 | 4.35E-13 | 0.99 | 5.37E-16 | 2.10E-18 | 1.44 |
| 204679_at | KCNK1 | 1.43E-05 | 2.68E-06 | 0.86 | 1.49E-08 | 9.67E-10 | 1.11 | 1.63E-06 | 1.21E-07 | 1.26 |
| 213273_at | TENM4 | 1.68E-09 | 1.41E-10 | 0.86 | 5.88E-13 | 1.42E-14 | 1.02 | 3.04E-06 | 2.48E-07 | 1.14 |
| 216228_s_at | WDHD1 | 5.04E-12 | 2.51E-13 | 0.86 | 6.17E-08 | 4.57E-09 | 0.82 | 5.63E-06 | 4.99E-07 | 0.99 |
| 231008_at | UNC5CL | 1.22E-06 | 1.82E-07 | 0.86 | 3.30E-12 | 9.43E-14 | 1.45 | 1.92E-13 | 1.59E-15 | 2.29 |
| 209635_at | AP1S1 | 1.93E-13 | 7.03E-15 | 0.86 | 3.28E-14 | 5.98E-16 | 0.82 | 1.52E-07 | 8.08E-09 | 0.71 |
| 224467_s_at | PDCD2L | 7.24E-16 | 1.49E-17 | 0.86 | 1.04E-10 | 4.19E-12 | 0.71 | 6.29E-06 | 5.68E-07 | 0.71 |
| 202487_s_at | H2AFV | 3.54E-16 | 6.67E-18 | 0.86 | 4.80E-17 | 4.55E-19 | 0.88 | 6.69E-11 | 1.28E-12 | 0.98 |
| 209064_x_at | PAIP1 | 3.61E-11 | 2.14E-12 | 0.87 | 2.96E-13 | 6.67E-15 | 0.88 | 9.26E-07 | 6.40E-08 | 0.74 |
| 228427_at | FBXO16 | 1.64E-12 | 7.34E-14 | 0.87 | 1.11E-08 | 7.01E-10 | 0.73 | 2.94E-11 | 5.08E-13 | 1.15 |
| 1553928_at | ELMOD2 | 3.77E-11 | 2.25E-12 | 0.87 | 6.92E-05 | 1.06E-05 | 0.72 | 2.23E-11 | 3.73E-13 | 1.08 |
| 212902_at | SEC24A | 2.36E-08 | 2.50E-09 | 0.87 | 9.03E-10 | 4.50E-11 | 0.81 | 5.82E-11 | 1.10E-12 | 1.06 |
| 201418_s_at | SOX4 | 1.61E-08 | 1.64E-09 | 0.87 | 1.12E-08 | 7.07E-10 | 0.88 | 2.21E-09 | 6.90E-11 | 1.02 |
| 209836_x_at | BOLA2B///BOLA2 | 4.84E-14 | 1.53E-15 | 0.87 | 2.40E-13 | 5.31E-15 | 0.75 | 2.95E-09 | 9.61E-11 | 1.06 |
| 212909_at | LYPD1 | 2.72E-09 | 2.39E-10 | 0.87 | 3.50E-08 | 2.45E-09 | 0.89 | 1.67E-06 | 1.25E-07 | 2.90 |
| 228933_at | NHS | 4.79E-07 | 6.59E-08 | 0.88 | 1.71E-19 | 8.37E-22 | 1.89 | 5.54E-09 | 1.94E-10 | 1.55 |
| 235148_at | KRTCAP3 | 2.63E-09 | 2.29E-10 | 0.88 | 7.97E-12 | 2.50E-13 | 0.96 | 2.19E-06 | 1.71E-07 | 1.09 |
| 203876_s_at | MMP11 | 1.17E-10 | 7.63E-12 | 0.88 | 4.40E-12 | 1.30E-13 | 1.55 | 1.58E-05 | 1.60E-06 | 2.18 |
| 220318_at | EPN3 | 1.60E-14 | 4.58E-16 | 0.88 | 2.36E-13 | 5.18E-15 | 0.79 | 2.32E-20 | 1.57E-23 | 2.71 |
| 36936_at | TSTA3 | 5.39E-12 | 2.71E-13 | 0.88 | 2.15E-14 | 3.78E-16 | 0.97 | 9.16E-09 | 3.39E-10 | 1.00 |
| 227996_at | FARP1 | 1.25E-09 | 1.02E-10 | 0.89 | 1.09E-14 | 1.79E-16 | 1.16 | 4.09E-07 | 2.51E-08 | 1.05 |
| 202005_at | ST14 | 3.90E-11 | 2.33E-12 | 0.89 | 1.10E-19 | 5.06E-22 | 1.12 | 1.92E-12 | 2.10E-14 | 1.81 |
| 202200_s_at | SRPK1 | 6.28E-18 | 7.14E-20 | 0.89 | 1.03E-17 | 8.27E-20 | 0.91 | 2.23E-11 | 3.74E-13 | 0.86 |
| 213237_at | KNOP1 | 6.90E-14 | 2.27E-15 | 0.89 | 9.59E-16 | 1.25E-17 | 0.90 | 2.85E-10 | 6.65E-12 | 0.86 |
| 214039_s_at | LAPTM4B | 1.47E-11 | 8.06E-13 | 0.89 | 1.13E-15 | 1.49E-17 | 1.11 | 2.28E-09 | 7.14E-11 | 0.83 |
| 230650_at | SLCO5A1 | 1.15E-08 | 1.15E-09 | 0.89 | 3.64E-08 | 2.55E-09 | 0.92 | 4.57E-12 | 5.92E-14 | 1.90 |
| 211603_s_at | ETV4 | 1.03E-14 | 2.80E-16 | 0.89 | 6.03E-18 | 4.54E-20 | 1.11 | 8.80E-17 | 2.49E-19 | 3.04 |
| 222155_s_at | SLC52A2 | 1.18E-10 | 7.75E-12 | 0.89 | 2.67E-07 | 2.27E-08 | 0.73 | 9.41E-07 | 6.52E-08 | 0.77 |
| 200656_s_at | P4HB | 1.63E-13 | 5.80E-15 | 0.89 | 4.44E-06 | 5.12E-07 | 0.76 | 6.97E-07 | 4.63E-08 | 0.74 |
| 218720_x_at | SEZ6L2 | 3.53E-09 | 3.19E-10 | 0.90 | 3.20E-14 | 5.83E-16 | 1.51 | 1.77E-09 | 5.35E-11 | 1.88 |
| 206576_s_at | CEACAM1 | 1.89E-10 | 1.31E-11 | 0.90 | 1.81E-10 | 7.75E-12 | 1.07 | 3.21E-09 | 1.06E-10 | 1.45 |
| 214070_s_at | ATP10B | 5.87E-07 | 8.20E-08 | 0.90 | 6.89E-13 | 1.68E-14 | 1.50 | 1.18E-14 | 6.84E-17 | 3.61 |
| 235463_s_at | CERS6 | 3.65E-09 | 3.32E-10 | 0.90 | 3.95E-08 | 2.79E-09 | 0.84 | 4.42E-10 | 1.12E-11 | 1.08 |
| 208967_s_at | AK2 | 3.17E-17 | 4.44E-19 | 0.90 | 1.14E-12 | 2.92E-14 | 0.77 | 1.95E-10 | 4.31E-12 | 0.93 |
| 224950_at | PTGFRN | 3.24E-10 | 2.35E-11 | 0.91 | 1.80E-19 | 8.95E-22 | 1.23 | 6.30E-09 | 2.24E-10 | 1.25 |
| 226597_at | REEP6 | 5.60E-10 | 4.25E-11 | 0.91 | 2.05E-09 | 1.11E-10 | 0.90 | 3.26E-06 | 2.67E-07 | 1.23 |
| 202786_at | STK39 | 7.18E-12 | 3.71E-13 | 0.91 | 1.70E-19 | 8.32E-22 | 1.38 | 6.04E-10 | 1.58E-11 | 0.97 |
| 1554897_s_at | RHBDL2 | 1.23E-14 | 3.40E-16 | 0.91 | 2.13E-13 | 4.62E-15 | 0.99 | 6.19E-05 | 7.60E-06 | 1.15 |
| 238417_at | PGM2L1 | 3.34E-12 | 1.59E-13 | 0.91 | 1.32E-12 | 3.45E-14 | 0.90 | 1.12E-08 | 4.25E-10 | 0.90 |
| 224937_at | PTGFRN | 2.95E-09 | 2.61E-10 | 0.91 | 5.91E-19 | 3.41E-21 | 1.36 | 1.33E-07 | 7.00E-09 | 0.88 |
| 238996_x_at | ALDOA | 3.18E-10 | 2.30E-11 | 0.91 | 7.42E-11 | 2.90E-12 | 0.86 | 1.91E-05 | 1.99E-06 | 0.82 |
| 222532_at | SRPRB | 6.67E-09 | 6.34E-10 | 0.91 | 8.39E-12 | 2.64E-13 | 0.79 | 2.54E-08 | 1.07E-09 | 0.91 |
| 238567_at | SGPP2 | 3.58E-05 | 7.33E-06 | 0.91 | 2.67E-10 | 1.18E-11 | 1.39 | 8.17E-18 | 1.66E-20 | 2.56 |
| 228463_at | FOXA3 | 1.28E-07 | 1.56E-08 | 0.92 | 4.23E-07 | 3.77E-08 | 0.94 | 7.92E-07 | 5.36E-08 | 2.37 |
| 216905_s_at | ST14 | 5.24E-10 | 3.97E-11 | 0.92 | 1.24E-15 | 1.66E-17 | 1.15 | 6.64E-11 | 1.27E-12 | 1.69 |
| 225485_at | CEP41 | 2.54E-08 | 2.71E-09 | 0.92 | 7.57E-09 | 4.61E-10 | 0.84 | 2.54E-07 | 1.46E-08 | 0.82 |
| 208658_at | PDIA4 | 2.49E-14 | 7.41E-16 | 0.92 | 1.37E-15 | 1.86E-17 | 1.15 | 5.75E-16 | 2.32E-18 | 1.31 |
| 220013_at | EPHX3 | 4.18E-07 | 5.69E-08 | 0.92 | 1.12E-12 | 2.88E-14 | 1.11 | 6.55E-08 | 3.14E-09 | 1.76 |
| 225944_at | NLN | 3.63E-10 | 2.65E-11 | 0.92 | 6.96E-18 | 5.37E-20 | 1.28 | 8.19E-11 | 1.61E-12 | 0.89 |
| 218257_s_at | UGGT1 | 6.52E-15 | 1.70E-16 | 0.92 | 1.05E-14 | 1.72E-16 | 0.88 | 2.11E-12 | 2.34E-14 | 0.99 |
| 243894_at | SLC41A2 | 6.84E-12 | 3.52E-13 | 0.92 | 1.00E-07 | 7.81E-09 | 0.75 | 4.98E-10 | 1.27E-11 | 1.40 |
| 218163_at | MCTS1 | 2.85E-15 | 6.82E-17 | 0.92 | 4.16E-16 | 4.99E-18 | 0.86 | 2.73E-12 | 3.19E-14 | 0.97 |
| 208639_x_at | PDIA6 | 4.87E-16 | 9.52E-18 | 0.93 | 1.88E-13 | 4.02E-15 | 0.70 | 3.20E-11 | 5.59E-13 | 0.83 |
| 208308_s_at | GPI | 1.25E-12 | 5.44E-14 | 0.93 | 2.00E-12 | 5.42E-14 | 0.75 | 1.44E-05 | 1.44E-06 | 0.73 |
| 1558217_at | SLFN13 | 5.54E-08 | 6.34E-09 | 0.93 | 6.21E-11 | 2.38E-12 | 1.12 | 4.17E-08 | 1.89E-09 | 1.26 |
| 236979_at | BCL2L15 | 1.10E-10 | 7.17E-12 | 0.93 | 5.49E-14 | 1.05E-15 | 1.33 | 1.90E-06 | 1.45E-07 | 1.47 |
| 203287_at | LAD1 | 6.97E-12 | 3.59E-13 | 0.93 | 8.63E-16 | 1.12E-17 | 1.39 | 4.73E-12 | 6.18E-14 | 1.51 |
| 202710_at | BET1 | 4.67E-12 | 2.31E-13 | 0.94 | 2.76E-09 | 1.53E-10 | 0.72 | 1.15E-07 | 5.94E-09 | 0.73 |
| 1554576_a_at | ETV4 | 2.13E-15 | 4.97E-17 | 0.94 | 4.32E-16 | 5.23E-18 | 1.26 | 1.10E-11 | 1.67E-13 | 1.84 |
| 233337_s_at | SEZ6L2 | 1.44E-09 | 1.20E-10 | 0.94 | 6.56E-14 | 1.28E-15 | 1.44 | 1.10E-09 | 3.12E-11 | 1.87 |
| 225723_at | CCDC167 | 1.97E-14 | 5.73E-16 | 0.94 | 3.08E-12 | 8.71E-14 | 0.76 | 3.30E-06 | 2.71E-07 | 0.80 |
| 214463_x_at | HIST1H4J | 3.40E-12 | 1.63E-13 | 0.94 | 3.64E-13 | 8.40E-15 | 0.87 | 4.65E-06 | 4.00E-07 | 1.09 |
| 201287_s_at | SDC1 | 5.67E-16 | 1.12E-17 | 0.94 | 5.08E-12 | 1.52E-13 | 1.03 | 6.68E-14 | 4.85E-16 | 1.43 |
| 209873_s_at | PKP3 | 4.99E-16 | 9.77E-18 | 0.94 | 1.25E-12 | 3.26E-14 | 0.76 | 2.58E-08 | 1.09E-09 | 0.96 |
| 1552502_s_at | RHBDL2 | 3.15E-13 | 1.21E-14 | 0.95 | 3.90E-13 | 9.06E-15 | 1.35 | 6.23E-10 | 1.64E-11 | 1.56 |
| 205479_s_at | PLAU | 2.15E-05 | 4.18E-06 | 0.95 | 1.35E-09 | 7.03E-11 | 1.26 | 1.21E-05 | 1.19E-06 | 1.48 |
| 219105_x_at | ORC6 | 4.28E-17 | 6.16E-19 | 0.95 | 2.29E-10 | 1.00E-11 | 1.01 | 6.47E-10 | 1.71E-11 | 1.66 |
| 243010_at | MSI2 | 2.19E-16 | 3.94E-18 | 0.95 | 2.65E-15 | 3.82E-17 | 0.73 | 3.77E-14 | 2.56E-16 | 1.36 |
| 229360_at | ZNF280B | 2.58E-09 | 2.25E-10 | 0.95 | 1.73E-07 | 1.41E-08 | 0.79 | 3.45E-05 | 3.90E-06 | 1.04 |
| 215695_s_at | GYG2 | 4.18E-11 | 2.52E-12 | 0.96 | 1.22E-16 | 1.27E-18 | 0.93 | 2.08E-09 | 6.44E-11 | 1.89 |
| 223540_at | PVRL4 | 7.21E-13 | 3.00E-14 | 0.96 | 1.89E-14 | 3.28E-16 | 0.97 | 1.24E-11 | 1.94E-13 | 1.18 |
| 226488_at | RCCD1 | 8.12E-20 | 4.94E-22 | 0.96 | 2.47E-16 | 2.81E-18 | 0.78 | 2.32E-14 | 1.46E-16 | 1.06 |
| 210046_s_at | IDH2 | 3.10E-15 | 7.46E-17 | 0.96 | 2.15E-16 | 2.39E-18 | 0.91 | 1.27E-08 | 4.88E-10 | 0.99 |
| 227804_at | TLCD1 | 4.90E-11 | 2.99E-12 | 0.96 | 1.35E-15 | 1.81E-17 | 1.20 | 2.60E-12 | 3.01E-14 | 1.63 |
| 202721_s_at | GFPT1 | 3.09E-12 | 1.47E-13 | 0.96 | 1.50E-09 | 7.88E-11 | 0.89 | 1.48E-11 | 2.36E-13 | 1.18 |
| 219061_s_at | LAGE3 | 2.21E-16 | 3.99E-18 | 0.97 | 3.86E-17 | 3.56E-19 | 0.91 | 3.44E-07 | 2.06E-08 | 0.83 |
| 226346_at | MEX3A | 1.40E-17 | 1.79E-19 | 0.97 | 1.33E-12 | 3.46E-14 | 0.77 | 2.70E-07 | 1.56E-08 | 0.74 |
| 203276_at | LMNB1 | 3.27E-08 | 3.58E-09 | 0.97 | 1.98E-07 | 1.63E-08 | 0.92 | 4.87E-08 | 2.25E-09 | 0.95 |
| 208029_s_at | LAPTM4B | 1.54E-10 | 1.04E-11 | 0.97 | 2.05E-11 | 7.04E-13 | 1.14 | 2.97E-08 | 1.29E-09 | 0.96 |
| 243579_at | MSI2 | 8.91E-16 | 1.87E-17 | 0.97 | 9.20E-13 | 2.32E-14 | 0.71 | 3.10E-08 | 1.35E-09 | 0.77 |
| 214469_at | HIST1H2AE | 2.26E-08 | 2.38E-09 | 0.97 | 3.44E-06 | 3.83E-07 | 0.83 | 6.46E-08 | 3.09E-09 | 2.30 |
| 226444_at |  | 3.63E-07 | 4.88E-08 | 0.97 | 8.11E-09 | 4.97E-10 | 1.24 | 8.08E-09 | 2.94E-10 | 1.09 |
| 204678_s_at | KCNK1 | 4.59E-07 | 6.29E-08 | 0.98 | 5.94E-07 | 5.52E-08 | 1.02 | 3.90E-05 | 4.50E-06 | 1.23 |
| 222899_at | ITGA11 | 4.14E-13 | 1.63E-14 | 0.98 | 1.94E-15 | 2.72E-17 | 1.10 | 3.47E-07 | 2.08E-08 | 1.55 |
| 218392_x_at | SFXN1 | 2.40E-15 | 5.66E-17 | 0.98 | 4.30E-17 | 4.03E-19 | 1.04 | 1.15E-13 | 9.03E-16 | 1.09 |
| 228072_at | SYT12 | 5.29E-10 | 4.00E-11 | 0.98 | 6.41E-09 | 3.84E-10 | 0.85 | 2.63E-06 | 2.09E-07 | 1.11 |
| 226548_at | SBK1 | 5.77E-14 | 1.86E-15 | 0.98 | 4.96E-13 | 1.18E-14 | 0.96 | 4.13E-15 | 2.06E-17 | 2.58 |
| 225846_at | ESRP1 | 3.03E-09 | 2.68E-10 | 0.98 | 1.73E-08 | 1.13E-09 | 0.97 | 2.40E-06 | 1.89E-07 | 0.97 |
| 209186_at | ATP2A2 | 5.84E-16 | 1.16E-17 | 0.98 | 1.65E-18 | 1.07E-20 | 0.89 | 8.19E-14 | 6.17E-16 | 1.03 |
| 1553423_a_at | SLFN13 | 4.99E-08 | 5.63E-09 | 0.99 | 4.32E-08 | 3.08E-09 | 0.90 | 5.47E-09 | 1.91E-10 | 1.29 |
| 204734_at | KRT15 | 1.01E-05 | 1.83E-06 | 0.99 | 5.67E-08 | 4.17E-09 | 1.16 | 2.60E-07 | 1.50E-08 | 2.64 |
| 208358_s_at | UGT8 | 6.48E-10 | 4.99E-11 | 0.99 | 6.75E-08 | 5.05E-09 | 0.98 | 1.28E-05 | 1.27E-06 | 1.82 |
| 201004_at | SSR4 | 4.33E-13 | 1.72E-14 | 0.99 | 3.25E-24 | 4.69E-27 | 0.93 | 2.59E-16 | 8.83E-19 | 1.19 |
| 224623_at | LOC728554///THOC3 | 2.75E-13 | 1.04E-14 | 0.99 | 2.48E-14 | 4.43E-16 | 0.83 | 1.02E-11 | 1.52E-13 | 0.84 |
| 217787_s_at | GALNT2 | 1.59E-12 | 7.10E-14 | 0.99 | 4.32E-10 | 2.00E-11 | 0.74 | 2.67E-09 | 8.54E-11 | 0.93 |
| 205817_at | SIX1 | 7.95E-08 | 9.40E-09 | 0.99 | 7.31E-09 | 4.44E-10 | 1.17 | 6.42E-06 | 5.81E-07 | 2.34 |
| 241031_at | C2CD4A | 9.23E-05 | 2.07E-05 | 0.99 | 1.11E-06 | 1.10E-07 | 1.26 | 2.13E-11 | 3.54E-13 | 3.31 |
| 218858_at | DEPTOR | 1.02E-05 | 1.84E-06 | 1.00 | 4.24E-16 | 5.10E-18 | 1.60 | 8.85E-12 | 1.29E-13 | 1.50 |
| 208527_x_at | HIST1H2BE | 4.59E-13 | 1.84E-14 | 1.00 | 8.31E-10 | 4.11E-11 | 0.70 | 8.56E-07 | 5.85E-08 | 1.25 |
| 209771_x_at | CD24 | 3.96E-07 | 5.36E-08 | 1.00 | 4.00E-13 | 9.32E-15 | 1.54 | 6.24E-14 | 4.51E-16 | 1.84 |
| 228033_at | E2F7 | 1.89E-09 | 1.61E-10 | 1.00 | 7.32E-05 | 1.14E-05 | 0.77 | 9.04E-05 | 1.17E-05 | 1.09 |
| 204875_s_at | GMDS | 6.22E-10 | 4.78E-11 | 1.00 | 6.65E-11 | 2.57E-12 | 1.10 | 1.55E-05 | 1.58E-06 | 0.88 |
| 225943_at | NLN | 4.33E-11 | 2.61E-12 | 1.00 | 3.06E-16 | 3.53E-18 | 0.98 | 7.07E-16 | 2.91E-18 | 1.34 |
| 204856_at | B3GNT3 | 4.29E-16 | 8.23E-18 | 1.00 | 4.04E-14 | 7.54E-16 | 0.97 | 6.50E-17 | 1.82E-19 | 2.42 |
| 214282_at |  | 1.07E-08 | 1.06E-09 | 1.00 | 2.09E-09 | 1.13E-10 | 1.13 | 2.16E-09 | 6.73E-11 | 2.81 |
| 229256_at | PGM2L1 | 2.93E-11 | 1.71E-12 | 1.01 | 4.82E-10 | 2.27E-11 | 1.05 | 5.86E-07 | 3.80E-08 | 0.94 |
| 209641_s_at | ABCC3 | 1.24E-07 | 1.51E-08 | 1.01 | 1.44E-19 | 6.82E-22 | 1.75 | 1.32E-16 | 3.92E-19 | 2.73 |
| 209108_at | TSPAN6 | 2.00E-11 | 1.13E-12 | 1.01 | 1.69E-09 | 8.97E-11 | 1.04 | 9.71E-10 | 2.70E-11 | 1.07 |
| 227943_at |  | 3.22E-10 | 2.33E-11 | 1.01 | 1.44E-07 | 1.16E-08 | 0.78 | 2.18E-05 | 2.32E-06 | 0.84 |
| 209172_s_at | CENPF | 1.61E-14 | 4.61E-16 | 1.01 | 1.56E-08 | 1.02E-09 | 0.84 | 3.05E-06 | 2.48E-07 | 1.16 |
| 214096_s_at | SHMT2 | 1.35E-15 | 2.99E-17 | 1.01 | 1.46E-11 | 4.83E-13 | 0.84 | 3.33E-09 | 1.10E-10 | 0.81 |
| 208651_x_at | CD24 | 6.04E-07 | 8.45E-08 | 1.01 | 2.42E-12 | 6.65E-14 | 1.52 | 2.59E-09 | 8.27E-11 | 1.82 |
| 1552289_a_at | CILP2 | 4.01E-09 | 3.66E-10 | 1.01 | 1.79E-10 | 7.64E-12 | 1.15 | 1.13E-06 | 8.10E-08 | 1.34 |
| 226549_at | SBK1 | 8.57E-13 | 3.62E-14 | 1.01 | 6.88E-13 | 1.68E-14 | 1.13 | 1.77E-08 | 7.13E-10 | 1.67 |
| 208579_x_at | H2BFS | 1.45E-10 | 9.75E-12 | 1.01 | 3.51E-09 | 1.99E-10 | 0.83 | 5.41E-05 | 6.50E-06 | 0.94 |
| 216379_x_at | CD24 | 7.52E-07 | 1.07E-07 | 1.01 | 3.06E-13 | 6.90E-15 | 1.62 | 7.52E-14 | 5.56E-16 | 1.87 |
| 231233_at | PCAT6 | 1.25E-16 | 2.10E-18 | 1.02 | 2.40E-18 | 1.62E-20 | 1.09 | 8.82E-15 | 4.82E-17 | 1.53 |
| 224511_s_at | TXNDC17 | 8.28E-17 | 1.29E-18 | 1.02 | 3.88E-19 | 2.08E-21 | 1.16 | 2.80E-13 | 2.41E-15 | 1.24 |
| 222640_at | DNMT3A | 3.71E-17 | 5.28E-19 | 1.02 | 5.84E-11 | 2.23E-12 | 0.75 | 3.92E-10 | 9.72E-12 | 0.79 |
| 212322_at | SGPL1 | 1.79E-16 | 3.16E-18 | 1.02 | 1.85E-11 | 6.27E-13 | 0.73 | 3.63E-12 | 4.49E-14 | 0.99 |
| 203954_x_at | CLDN3 | 3.81E-08 | 4.20E-09 | 1.02 | 1.61E-10 | 6.81E-12 | 1.17 | 1.34E-08 | 5.20E-10 | 1.69 |
| 203988_s_at | FUT8 | 6.57E-10 | 5.08E-11 | 1.04 | 9.47E-18 | 7.54E-20 | 1.15 | 3.44E-11 | 6.06E-13 | 1.34 |
| 225240_s_at | MSI2 | 6.98E-14 | 2.30E-15 | 1.04 | 6.63E-12 | 2.04E-13 | 1.16 | 9.84E-15 | 5.45E-17 | 0.98 |
| 223798_at | SLC41A2 | 4.07E-12 | 1.99E-13 | 1.04 | 1.87E-09 | 9.99E-11 | 0.91 | 1.97E-14 | 1.23E-16 | 1.91 |
| 205894_at | ARSE | 3.32E-05 | 6.75E-06 | 1.04 | 3.86E-11 | 1.41E-12 | 1.64 | 1.13E-06 | 8.04E-08 | 1.92 |
| 208767_s_at | LAPTM4B | 9.93E-10 | 7.95E-11 | 1.04 | 1.22E-07 | 9.66E-09 | 1.07 | 1.73E-05 | 1.78E-06 | 1.10 |
| 212115_at | HN1L | 4.43E-19 | 3.41E-21 | 1.05 | 5.44E-25 | 5.67E-28 | 1.10 | 8.71E-13 | 8.56E-15 | 1.15 |
| 220011_at | AUNIP | 1.57E-17 | 2.04E-19 | 1.05 | 4.02E-13 | 9.40E-15 | 0.79 | 4.48E-08 | 2.05E-09 | 1.03 |
| 219655_at | SUGCT | 1.07E-08 | 1.06E-09 | 1.05 | 1.10E-19 | 5.07E-22 | 1.40 | 2.88E-12 | 3.41E-14 | 2.62 |
| 214373_at |  | 1.35E-07 | 1.66E-08 | 1.06 | 1.66E-05 | 2.20E-06 | 0.70 | 5.94E-05 | 7.24E-06 | 0.90 |
| 218073_s_at | NDC1 | 1.22E-14 | 3.37E-16 | 1.06 | 9.90E-06 | 1.24E-06 | 0.74 | 3.72E-06 | 3.11E-07 | 0.73 |
| 218741_at | CENPM | 1.35E-12 | 5.93E-14 | 1.06 | 3.47E-08 | 2.42E-09 | 0.71 | 2.54E-08 | 1.07E-09 | 1.77 |
| 218313_s_at | GALNT7 | 6.44E-12 | 3.30E-13 | 1.06 | 9.00E-18 | 7.11E-20 | 1.47 | 1.83E-17 | 4.03E-20 | 1.54 |
| 227055_at | METTL7B | 5.48E-08 | 6.26E-09 | 1.06 | 1.60E-16 | 1.71E-18 | 1.56 | 3.14E-13 | 2.74E-15 | 2.36 |
| 212680_x_at | PPP1R14B | 6.02E-18 | 6.80E-20 | 1.06 | 3.93E-18 | 2.78E-20 | 0.91 | 1.71E-08 | 6.87E-10 | 1.05 |
| 229377_at | GRTP1 | 8.18E-09 | 7.89E-10 | 1.06 | 8.93E-13 | 2.23E-14 | 1.27 | 6.32E-07 | 4.13E-08 | 1.11 |
| 230966_at | IL4I1 | 1.56E-07 | 1.95E-08 | 1.06 | 1.50E-06 | 1.54E-07 | 0.84 | 1.09E-07 | 5.58E-09 | 1.73 |
| 226455_at | CREB3L4 | 1.67E-10 | 1.14E-11 | 1.07 | 1.19E-17 | 9.72E-20 | 1.09 | 5.42E-08 | 2.54E-09 | 0.88 |
| 230349_at | XKRX | 1.77E-08 | 1.83E-09 | 1.07 | 2.79E-07 | 2.39E-08 | 0.98 | 5.05E-08 | 2.34E-09 | 1.91 |
| 230323_s_at | TMEM45B | 5.69E-06 | 9.76E-07 | 1.07 | 4.89E-08 | 3.53E-09 | 1.20 | 6.32E-09 | 2.25E-10 | 1.70 |
| 226622_at | MUC20 | 2.66E-05 | 5.29E-06 | 1.07 | 1.06E-16 | 1.10E-18 | 1.78 | 3.54E-09 | 1.18E-10 | 1.98 |
| 266_s_at | CD24 | 1.32E-06 | 1.99E-07 | 1.07 | 9.46E-13 | 2.39E-14 | 1.69 | 1.15E-11 | 1.75E-13 | 1.90 |
| 208161_s_at | ABCC3 | 2.35E-07 | 3.04E-08 | 1.07 | 4.25E-21 | 1.36E-23 | 2.13 | 9.08E-20 | 8.30E-23 | 2.79 |
| 205534_at | PCDH7 | 1.55E-07 | 1.93E-08 | 1.07 | 1.14E-11 | 3.71E-13 | 1.48 | 3.99E-05 | 4.61E-06 | 1.51 |
| 205195_at | AP1S1 | 1.27E-11 | 6.86E-13 | 1.07 | 8.15E-09 | 5.00E-10 | 0.84 | 5.04E-06 | 4.39E-07 | 1.42 |
| 227271_at | FGF11 | 6.54E-16 | 1.33E-17 | 1.07 | 3.15E-10 | 1.41E-11 | 0.78 | 6.36E-09 | 2.27E-10 | 1.40 |
| 1568763_s_at | LOC728613///PDCD6 | 5.70E-10 | 4.34E-11 | 1.07 | 1.76E-08 | 1.16E-09 | 0.90 | 4.98E-07 | 3.17E-08 | 1.09 |
| 215380_s_at | GGCT | 4.28E-16 | 8.21E-18 | 1.07 | 5.35E-21 | 1.75E-23 | 1.17 | 8.30E-15 | 4.48E-17 | 1.03 |
| 215942_s_at | GTSE1 | 4.30E-15 | 1.07E-16 | 1.08 | 3.18E-08 | 2.21E-09 | 0.88 | 9.89E-06 | 9.43E-07 | 1.33 |
| 230831_at | FRMD5 | 4.41E-11 | 2.66E-12 | 1.08 | 9.40E-15 | 1.53E-16 | 1.51 | 8.35E-19 | 1.16E-21 | 4.09 |
| 211048_s_at | PDIA4 | 1.93E-11 | 1.08E-12 | 1.08 | 8.28E-16 | 1.07E-17 | 1.08 | 7.39E-10 | 1.99E-11 | 1.05 |
| 218930_s_at | TMEM106B | 1.15E-18 | 1.04E-20 | 1.08 | 5.68E-16 | 7.12E-18 | 1.37 | 3.42E-14 | 2.29E-16 | 1.29 |
| 204922_at | C11orf80 | 1.08E-15 | 2.32E-17 | 1.08 | 9.11E-13 | 2.29E-14 | 0.80 | 8.37E-09 | 3.06E-10 | 0.83 |
| 220192_x_at | SPDEF | 4.20E-09 | 3.85E-10 | 1.09 | 7.49E-14 | 1.48E-15 | 1.10 | 1.57E-14 | 9.60E-17 | 2.36 |
| 226803_at | CHMP4C | 6.48E-11 | 4.04E-12 | 1.09 | 1.98E-06 | 2.09E-07 | 0.76 | 1.60E-05 | 1.63E-06 | 0.75 |
| 219773_at | NOX4 | 4.69E-08 | 5.27E-09 | 1.09 | 1.79E-05 | 2.38E-06 | 0.92 | 1.89E-05 | 1.97E-06 | 1.42 |
| 218856_at | TNFRSF21 | 1.26E-13 | 4.41E-15 | 1.09 | 6.27E-12 | 1.91E-13 | 0.98 | 1.69E-09 | 5.08E-11 | 1.25 |
| 1558290_a_at | PVT1 | 1.92E-12 | 8.68E-14 | 1.09 | 5.27E-13 | 1.25E-14 | 1.06 | 2.29E-12 | 2.61E-14 | 1.46 |
| 227103_s_at | ECE2 | 1.30E-17 | 1.65E-19 | 1.10 | 2.28E-12 | 6.26E-14 | 0.81 | 8.26E-10 | 2.25E-11 | 1.08 |
| 226670_s_at | PABPC1L | 5.15E-11 | 3.16E-12 | 1.10 | 1.79E-05 | 2.38E-06 | 0.98 | 5.25E-09 | 1.83E-10 | 1.42 |
| 208190_s_at | LSR | 7.56E-12 | 3.92E-13 | 1.10 | 4.88E-16 | 6.02E-18 | 1.04 | 3.14E-07 | 1.86E-08 | 1.01 |
| 1568574_x_at | SPP1 | 1.71E-13 | 6.15E-15 | 1.10 | 9.96E-09 | 6.23E-10 | 1.06 | 1.01E-07 | 5.11E-09 | 2.40 |
| 203476_at | TPBG | 9.94E-12 | 5.29E-13 | 1.10 | 3.44E-18 | 2.40E-20 | 1.29 | 3.10E-08 | 1.35E-09 | 1.17 |
| 205450_at | PHKA1 | 3.73E-20 | 2.00E-22 | 1.10 | 2.26E-14 | 4.00E-16 | 0.72 | 2.78E-12 | 3.25E-14 | 1.31 |
| 236044_at | PPAPDC1A | 1.57E-10 | 1.06E-11 | 1.10 | 1.81E-09 | 9.68E-11 | 1.15 | 3.35E-05 | 3.77E-06 | 1.59 |
| 202722_s_at | GFPT1 | 1.41E-17 | 1.81E-19 | 1.10 | 3.46E-13 | 7.91E-15 | 1.19 | 4.99E-14 | 3.46E-16 | 1.18 |
| 206499_s_at | RCC1 | 1.80E-15 | 4.11E-17 | 1.11 | 5.16E-19 | 2.90E-21 | 1.24 | 4.63E-16 | 1.73E-18 | 1.15 |
| 227350_at | HELLS | 2.63E-12 | 1.23E-13 | 1.11 | 2.31E-07 | 1.94E-08 | 0.90 | 1.57E-06 | 1.17E-07 | 0.84 |
| 219215_s_at | SLC39A4 | 3.26E-12 | 1.55E-13 | 1.11 | 6.58E-09 | 3.96E-10 | 0.92 | 2.07E-06 | 1.60E-07 | 1.16 |
| 217791_s_at | ALDH18A1 | 1.70E-16 | 2.99E-18 | 1.11 | 3.53E-14 | 6.50E-16 | 0.87 | 1.84E-07 | 1.01E-08 | 0.79 |
| 205167_s_at | CDC25C | 1.11E-17 | 1.37E-19 | 1.11 | 3.36E-10 | 1.51E-11 | 0.76 | 7.48E-09 | 2.70E-10 | 2.10 |
| 209008_x_at | KRT8 | 1.69E-10 | 1.15E-11 | 1.11 | 5.26E-05 | 7.86E-06 | 0.71 | 1.01E-08 | 3.79E-10 | 1.40 |
| 205780_at | BIK | 2.10E-08 | 2.21E-09 | 1.12 | 7.96E-10 | 3.91E-11 | 1.03 | 3.64E-13 | 3.28E-15 | 2.23 |
| 220638_s_at | CBLC | 6.23E-15 | 1.62E-16 | 1.12 | 3.30E-11 | 1.18E-12 | 0.82 | 3.04E-05 | 3.38E-06 | 1.88 |
| 229276_at | IGSF9 | 1.13E-15 | 2.44E-17 | 1.12 | 1.40E-15 | 1.91E-17 | 1.13 | 5.13E-16 | 1.97E-18 | 2.80 |
| 227240_at | NGEF | 1.10E-13 | 3.78E-15 | 1.12 | 2.77E-15 | 4.02E-17 | 1.08 | 2.83E-06 | 2.28E-07 | 1.72 |
| 201690_s_at | TPD52 | 1.52E-16 | 2.61E-18 | 1.12 | 5.43E-10 | 2.58E-11 | 0.76 | 3.13E-07 | 1.86E-08 | 0.75 |
| 209631_s_at | GPR37 | 1.26E-07 | 1.54E-08 | 1.12 | 1.00E-07 | 7.82E-09 | 1.23 | 7.60E-06 | 7.04E-07 | 2.47 |
| 214088_s_at | FUT3 | 3.80E-09 | 3.46E-10 | 1.12 | 1.26E-18 | 7.87E-21 | 1.33 | 2.01E-09 | 6.18E-11 | 1.50 |
| 224448_s_at | UQCC2 | 1.56E-17 | 2.03E-19 | 1.12 | 6.99E-21 | 2.38E-23 | 1.22 | 5.09E-08 | 2.36E-09 | 1.10 |
| 205774_at | F12 | 1.22E-15 | 2.67E-17 | 1.12 | 4.13E-11 | 1.52E-12 | 0.93 | 2.24E-07 | 1.26E-08 | 1.37 |
| 226150_at | PPAPDC1B | 2.40E-11 | 1.38E-12 | 1.12 | 1.24E-14 | 2.07E-16 | 1.04 | 3.15E-10 | 7.50E-12 | 1.11 |
| 228262_at | MAP7D2 | 2.80E-07 | 3.67E-08 | 1.12 | 5.19E-12 | 1.56E-13 | 1.68 | 8.42E-08 | 4.18E-09 | 2.74 |
| 223700_at | MND1 | 5.88E-16 | 1.17E-17 | 1.12 | 1.48E-11 | 4.91E-13 | 1.10 | 9.10E-12 | 1.33E-13 | 1.69 |
| 218897_at | TMEM177 | 3.57E-16 | 6.73E-18 | 1.13 | 7.10E-18 | 5.51E-20 | 1.07 | 6.11E-15 | 3.24E-17 | 1.19 |
| 218826_at | SLC35F2 | 4.89E-13 | 1.97E-14 | 1.13 | 2.13E-12 | 5.80E-14 | 1.05 | 1.02E-08 | 3.84E-10 | 1.07 |
| 1554008_at | OSMR | 1.93E-08 | 2.01E-09 | 1.13 | 5.13E-07 | 4.69E-08 | 0.82 | 1.63E-08 | 6.50E-10 | 1.39 |
| 226226_at | TMEM45B | 8.86E-09 | 8.61E-10 | 1.14 | 8.02E-10 | 3.95E-11 | 0.86 | 2.87E-09 | 9.29E-11 | 1.57 |
| 225766_s_at | TNPO1 | 1.01E-17 | 1.23E-19 | 1.15 | 9.88E-10 | 4.98E-11 | 0.94 | 9.38E-12 | 1.38E-13 | 0.99 |
| 211056_s_at | SRD5A1 | 2.45E-20 | 1.21E-22 | 1.15 | 4.46E-15 | 6.81E-17 | 1.15 | 3.81E-12 | 4.79E-14 | 1.69 |
| 231941_s_at | MUC20 | 1.90E-05 | 3.64E-06 | 1.15 | 2.07E-14 | 3.63E-16 | 1.77 | 3.10E-07 | 1.84E-08 | 1.77 |
| 201417_at | SOX4 | 5.39E-14 | 1.72E-15 | 1.15 | 3.63E-11 | 1.32E-12 | 1.03 | 5.56E-15 | 2.88E-17 | 0.80 |
| 222416_at | ALDH18A1 | 8.17E-19 | 7.00E-21 | 1.15 | 1.93E-26 | 1.41E-29 | 1.09 | 3.58E-17 | 8.96E-20 | 1.07 |
| 235911_at | MFI2 | 4.96E-09 | 4.61E-10 | 1.15 | 4.60E-10 | 2.15E-11 | 0.80 | 8.20E-06 | 7.67E-07 | 1.33 |
| 204702_s_at | NFE2L3 | 3.28E-12 | 1.56E-13 | 1.15 | 1.26E-07 | 1.00E-08 | 0.88 | 1.71E-06 | 1.29E-07 | 0.96 |
| 234725_s_at | SEMA4B | 2.54E-12 | 1.18E-13 | 1.16 | 7.70E-10 | 3.78E-11 | 0.83 | 4.24E-10 | 1.07E-11 | 1.57 |
| 209803_s_at | PHLDA2 | 1.26E-05 | 2.32E-06 | 1.16 | 1.40E-09 | 7.30E-11 | 1.37 | 7.04E-11 | 1.36E-12 | 1.73 |
| 241453_at | PTK2 | 5.52E-12 | 2.78E-13 | 1.16 | 1.16E-08 | 7.36E-10 | 0.78 | 9.99E-10 | 2.79E-11 | 1.36 |
| 201340_s_at | ENC1 | 1.51E-09 | 1.26E-10 | 1.17 | 6.32E-11 | 2.43E-12 | 1.31 | 8.76E-10 | 2.42E-11 | 1.03 |
| 221648_s_at |  | 1.47E-15 | 3.28E-17 | 1.17 | 2.94E-06 | 3.21E-07 | 0.83 | 5.59E-08 | 2.63E-09 | 1.55 |
| 228868_x_at | CDT1 | 7.70E-14 | 2.56E-15 | 1.17 | 5.93E-06 | 7.06E-07 | 0.82 | 7.80E-05 | 9.90E-06 | 1.26 |
| 225750_at | ERO1L | 1.10E-13 | 3.80E-15 | 1.18 | 9.32E-19 | 5.68E-21 | 1.07 | 7.24E-11 | 1.41E-12 | 1.15 |
| 223311_s_at | MTA3 | 1.73E-20 | 7.90E-23 | 1.18 | 1.04E-21 | 2.96E-24 | 0.82 | 2.80E-16 | 9.66E-19 | 1.05 |
| 220520_s_at | NUP62CL | 1.71E-10 | 1.16E-11 | 1.19 | 2.70E-09 | 1.50E-10 | 1.10 | 1.64E-05 | 1.68E-06 | 1.08 |
| 218115_at | ASF1B | 7.52E-17 | 1.17E-18 | 1.19 | 1.49E-09 | 7.77E-11 | 0.84 | 3.99E-08 | 1.80E-09 | 1.03 |
| 206074_s_at | HMGA1 | 3.69E-17 | 5.23E-19 | 1.19 | 5.69E-09 | 3.37E-10 | 0.89 | 1.76E-06 | 1.33E-07 | 1.05 |
| 222843_at | FIGNL1 | 5.08E-13 | 2.06E-14 | 1.20 | 1.34E-11 | 4.41E-13 | 1.26 | 1.86E-08 | 7.55E-10 | 0.96 |
| 205309_at | SMPDL3B | 1.05E-10 | 6.80E-12 | 1.20 | 7.06E-10 | 3.43E-11 | 1.29 | 5.46E-14 | 3.88E-16 | 2.26 |
| 220030_at | STYK1 | 1.88E-09 | 1.60E-10 | 1.20 | 5.23E-08 | 3.81E-09 | 1.11 | 4.15E-10 | 1.04E-11 | 1.85 |
| 214068_at | BEAN1 | 8.77E-08 | 1.04E-08 | 1.21 | 3.05E-06 | 3.36E-07 | 0.96 | 1.39E-06 | 1.02E-07 | 1.47 |
| 221436_s_at | CDCA3 | 6.71E-19 | 5.53E-21 | 1.21 | 1.91E-09 | 1.02E-10 | 0.72 | 2.00E-06 | 1.54E-07 | 1.16 |
| 201468_s_at | NQO1 | 1.71E-06 | 2.62E-07 | 1.21 | 2.14E-14 | 3.75E-16 | 1.64 | 7.97E-07 | 5.40E-08 | 1.52 |
| 209434_s_at | PPAT | 1.67E-13 | 5.98E-15 | 1.22 | 4.89E-11 | 1.83E-12 | 0.75 | 1.46E-09 | 4.30E-11 | 0.88 |
| 201563_at | SORD | 8.16E-14 | 2.74E-15 | 1.22 | 1.31E-19 | 6.18E-22 | 1.29 | 7.27E-10 | 1.95E-11 | 1.22 |
| 242546_at | LINC01296 | 4.48E-13 | 1.79E-14 | 1.23 | 3.86E-07 | 3.41E-08 | 0.87 | 2.76E-05 | 3.02E-06 | 1.35 |
| 201923_at | PRDX4 | 1.21E-16 | 2.02E-18 | 1.23 | 5.60E-18 | 4.16E-20 | 1.04 | 2.81E-09 | 9.07E-11 | 0.98 |
| 1552766_at | HS6ST2 | 4.54E-09 | 4.19E-10 | 1.23 | 4.73E-08 | 3.40E-09 | 1.27 | 8.35E-11 | 1.65E-12 | 3.00 |
| 209596_at | MXRA5 | 2.01E-08 | 2.10E-09 | 1.23 | 1.46E-11 | 4.85E-13 | 1.46 | 7.03E-05 | 8.78E-06 | 1.03 |
| 201548_s_at | KDM5B | 4.43E-19 | 3.42E-21 | 1.24 | 1.58E-11 | 5.26E-13 | 0.74 | 2.51E-15 | 1.18E-17 | 0.98 |
| 222750_s_at | SRD5A3 | 3.24E-09 | 2.90E-10 | 1.24 | 4.10E-17 | 3.81E-19 | 1.37 | 3.38E-12 | 4.14E-14 | 1.59 |
| 220840_s_at | C1orf112 | 5.68E-15 | 1.45E-16 | 1.24 | 8.47E-11 | 3.36E-12 | 0.98 | 3.42E-09 | 1.14E-10 | 1.00 |
| 219117_s_at | FKBP11 | 3.37E-09 | 3.02E-10 | 1.24 | 2.13E-13 | 4.63E-15 | 1.14 | 6.69E-06 | 6.10E-07 | 0.98 |
| 203046_s_at | TIMELESS | 4.99E-17 | 7.31E-19 | 1.24 | 1.13E-15 | 1.50E-17 | 0.86 | 9.14E-08 | 4.59E-09 | 0.87 |
| 1560916_a_at | DPY19L1 | 6.37E-13 | 2.62E-14 | 1.24 | 1.75E-10 | 7.43E-12 | 1.22 | 6.13E-10 | 1.61E-11 | 1.58 |
| 205909_at | POLE2 | 1.49E-14 | 4.20E-16 | 1.24 | 1.21E-09 | 6.24E-11 | 0.96 | 1.98E-05 | 2.08E-06 | 0.85 |
| 204767_s_at | FEN1 | 1.09E-15 | 2.36E-17 | 1.25 | 2.50E-12 | 6.91E-14 | 1.00 | 3.39E-07 | 2.03E-08 | 1.03 |
| 222037_at | MCM4 | 1.77E-15 | 4.04E-17 | 1.26 | 3.38E-08 | 2.35E-09 | 0.89 | 1.24E-05 | 1.22E-06 | 1.18 |
| 211762_s_at | KPNA2 | 4.10E-16 | 7.85E-18 | 1.26 | 5.98E-10 | 2.87E-11 | 0.86 | 7.12E-07 | 4.76E-08 | 0.91 |
| 201689_s_at | TPD52 | 1.33E-13 | 4.66E-15 | 1.26 | 4.97E-05 | 7.39E-06 | 0.76 | 1.53E-06 | 1.14E-07 | 0.81 |
| 223530_at | TDRKH | 1.42E-13 | 5.01E-15 | 1.27 | 4.03E-14 | 7.51E-16 | 1.18 | 2.29E-12 | 2.59E-14 | 1.49 |
| 200700_s_at | KDELR2 | 7.20E-18 | 8.34E-20 | 1.27 | 5.26E-17 | 5.03E-19 | 1.14 | 7.18E-11 | 1.39E-12 | 1.30 |
| 202952_s_at | ADAM12 | 5.28E-12 | 2.65E-13 | 1.27 | 2.49E-07 | 2.10E-08 | 0.87 | 5.31E-06 | 4.66E-07 | 1.89 |
| 209891_at | SPC25 | 1.90E-13 | 6.89E-15 | 1.27 | 9.23E-08 | 7.13E-09 | 0.91 | 3.00E-05 | 3.33E-06 | 1.44 |
| 204768_s_at | FEN1 | 5.16E-15 | 1.31E-16 | 1.28 | 6.88E-09 | 4.16E-10 | 0.82 | 2.37E-05 | 2.55E-06 | 0.75 |
| 226980_at | DEPDC1B | 6.42E-17 | 9.66E-19 | 1.28 | 6.68E-08 | 4.99E-09 | 1.04 | 2.87E-09 | 9.28E-11 | 2.05 |
| 203022_at | RNASEH2A | 1.52E-19 | 9.98E-22 | 1.28 | 7.59E-11 | 2.98E-12 | 0.85 | 1.26E-06 | 9.12E-08 | 0.84 |
| 239148_at | MARVELD3 | 1.28E-18 | 1.18E-20 | 1.28 | 5.46E-15 | 8.49E-17 | 1.00 | 2.57E-10 | 5.91E-12 | 1.09 |
| 208103_s_at | ANP32E | 5.99E-13 | 2.45E-14 | 1.28 | 4.44E-13 | 1.05E-14 | 1.20 | 9.28E-07 | 6.42E-08 | 1.11 |
| 212023_s_at | MKI67 | 1.50E-17 | 1.93E-19 | 1.28 | 2.72E-10 | 1.21E-11 | 0.81 | 4.09E-08 | 1.85E-09 | 1.28 |
| 217871_s_at | MIF | 1.10E-19 | 6.93E-22 | 1.29 | 5.89E-17 | 5.68E-19 | 0.87 | 4.39E-11 | 7.92E-13 | 1.13 |
| 210608_s_at | FUT2 | 1.44E-12 | 6.37E-14 | 1.29 | 3.75E-25 | 3.65E-28 | 1.87 | 1.56E-14 | 9.43E-17 | 1.84 |
| 202094_at | BIRC5 | 6.03E-18 | 6.83E-20 | 1.30 | 3.00E-09 | 1.68E-10 | 0.85 | 8.86E-07 | 6.09E-08 | 1.74 |
| 218350_s_at | GMNN | 4.30E-16 | 8.27E-18 | 1.30 | 3.50E-17 | 3.18E-19 | 1.08 | 2.87E-07 | 1.68E-08 | 0.80 |
| 219874_at | SLC12A8 | 7.43E-15 | 1.96E-16 | 1.30 | 1.13E-09 | 5.75E-11 | 0.97 | 1.10E-05 | 1.06E-06 | 1.31 |
| 203417_at | MFAP2 | 1.27E-09 | 1.04E-10 | 1.30 | 8.31E-11 | 3.28E-12 | 1.10 | 6.97E-06 | 6.39E-07 | 0.98 |
| 223895_s_at | EPN3 | 1.41E-15 | 3.13E-17 | 1.31 | 6.59E-11 | 2.54E-12 | 0.88 | 1.44E-11 | 2.29E-13 | 1.80 |
| 239196_at | ANKRD22 | 2.13E-09 | 1.82E-10 | 1.31 | 3.34E-13 | 7.62E-15 | 1.45 | 1.69E-15 | 7.56E-18 | 2.17 |
| 229796_at | SIX4 | 9.76E-10 | 7.82E-11 | 1.32 | 7.08E-13 | 1.73E-14 | 1.24 | 5.80E-14 | 4.18E-16 | 2.28 |
| 222581_at | XPR1 | 2.18E-14 | 6.42E-16 | 1.32 | 1.70E-18 | 1.11E-20 | 1.26 | 1.91E-10 | 4.22E-12 | 1.27 |
| 1553413_at | FLJ13744 | 5.24E-07 | 7.26E-08 | 1.32 | 7.37E-10 | 3.60E-11 | 1.65 | 2.64E-07 | 1.52E-08 | 3.81 |
| 219429_at | FA2H | 1.73E-10 | 1.18E-11 | 1.32 | 2.01E-13 | 4.35E-15 | 1.38 | 1.65E-06 | 1.23E-07 | 1.31 |
| 205828_at | MMP3 | 3.76E-06 | 6.21E-07 | 1.32 | 2.42E-06 | 2.60E-07 | 0.80 | 1.42E-07 | 7.55E-09 | 2.48 |
| 209109_s_at | TSPAN6 | 2.11E-15 | 4.91E-17 | 1.32 | 1.98E-12 | 5.34E-14 | 1.22 | 2.53E-11 | 4.30E-13 | 1.20 |
| 231849_at | KRT80 | 2.71E-14 | 8.16E-16 | 1.33 | 1.88E-12 | 5.06E-14 | 1.22 | 9.06E-11 | 1.82E-12 | 2.07 |
| 219121_s_at | ESRP1 | 2.53E-12 | 1.18E-13 | 1.33 | 4.75E-07 | 4.29E-08 | 0.91 | 2.00E-07 | 1.10E-08 | 1.18 |
| 224762_at | SERINC2 | 3.49E-14 | 1.08E-15 | 1.33 | 1.90E-22 | 4.13E-25 | 1.70 | 1.24E-18 | 1.79E-21 | 2.12 |
| 204603_at | EXO1 | 2.59E-16 | 4.75E-18 | 1.34 | 2.80E-09 | 1.56E-10 | 0.78 | 3.62E-07 | 2.18E-08 | 2.39 |
| 205107_s_at | EFNA4 | 1.88E-22 | 3.54E-25 | 1.34 | 8.10E-28 | 3.70E-31 | 1.61 | 1.15E-17 | 2.39E-20 | 1.26 |
| 205687_at | UBFD1 | 9.54E-18 | 1.15E-19 | 1.34 | 3.86E-11 | 1.41E-12 | 0.86 | 1.32E-11 | 2.06E-13 | 1.05 |
| 201013_s_at | PAICS | 2.60E-19 | 1.87E-21 | 1.35 | 1.81E-18 | 1.18E-20 | 1.17 | 8.23E-10 | 2.24E-11 | 1.01 |
| 227371_at | BAIAP2L1 | 2.34E-13 | 8.70E-15 | 1.35 | 8.43E-12 | 2.66E-13 | 0.94 | 5.94E-10 | 1.54E-11 | 1.69 |
| 201416_at | SOX4 | 9.77E-13 | 4.18E-14 | 1.35 | 2.63E-13 | 5.86E-15 | 1.18 | 1.58E-16 | 4.99E-19 | 1.61 |
| 227801_at | TRIM59 | 2.25E-14 | 6.65E-16 | 1.35 | 8.39E-11 | 3.33E-12 | 1.15 | 5.57E-09 | 1.96E-10 | 1.32 |
| 213008_at | FANCI | 1.52E-16 | 2.63E-18 | 1.36 | 6.28E-09 | 3.76E-10 | 0.78 | 6.31E-05 | 7.75E-06 | 1.06 |
| 203968_s_at | CDC6 | 7.26E-16 | 1.50E-17 | 1.36 | 1.09E-08 | 6.86E-10 | 0.84 | 6.49E-08 | 3.11E-09 | 1.36 |
| 212949_at | NCAPH | 5.70E-16 | 1.13E-17 | 1.36 | 6.25E-10 | 3.00E-11 | 0.72 | 2.55E-09 | 8.10E-11 | 1.60 |
| 229538_s_at | IQGAP3 | 1.86E-17 | 2.45E-19 | 1.36 | 9.35E-11 | 3.74E-12 | 1.10 | 8.86E-09 | 3.26E-10 | 1.77 |
| 226452_at | PDK1 | 5.09E-11 | 3.12E-12 | 1.37 | 6.44E-10 | 3.10E-11 | 1.03 | 8.69E-05 | 1.12E-05 | 0.93 |
| 204401_at | KCNN4 | 2.66E-11 | 1.54E-12 | 1.37 | 2.75E-12 | 7.67E-14 | 1.36 | 2.19E-07 | 1.22E-08 | 1.96 |
| 223556_at | HELLS | 6.05E-15 | 1.56E-16 | 1.37 | 8.19E-08 | 6.24E-09 | 0.92 | 8.16E-08 | 4.03E-09 | 1.26 |
| 225237_s_at | MSI2 | 3.95E-12 | 1.92E-13 | 1.37 | 4.06E-08 | 2.87E-09 | 1.07 | 7.24E-13 | 7.00E-15 | 1.08 |
| 227133_at | FAM199X | 3.72E-15 | 9.13E-17 | 1.37 | 2.57E-11 | 9.05E-13 | 1.12 | 1.26E-14 | 7.39E-17 | 1.38 |
| 242283_at | DNAH14 | 1.69E-20 | 7.68E-23 | 1.39 | 1.29E-14 | 2.16E-16 | 1.21 | 1.04E-14 | 5.89E-17 | 1.65 |
| 205499_at | SRPX2 | 7.96E-10 | 6.23E-11 | 1.40 | 5.89E-13 | 1.42E-14 | 1.44 | 8.92E-05 | 1.16E-05 | 1.47 |
| 217901_at | DSG2 | 8.20E-15 | 2.18E-16 | 1.40 | 5.11E-07 | 4.66E-08 | 0.97 | 1.78E-07 | 9.66E-09 | 1.19 |
| 204017_at | KDELR3 | 3.27E-15 | 7.93E-17 | 1.40 | 4.58E-22 | 1.17E-24 | 1.69 | 3.45E-18 | 5.94E-21 | 1.78 |
| 213668_s_at | SOX4 | 3.37E-11 | 1.99E-12 | 1.41 | 1.58E-09 | 8.34E-11 | 1.06 | 1.77E-12 | 1.91E-14 | 1.25 |
| 205393_s_at | CHEK1 | 2.78E-16 | 5.13E-18 | 1.41 | 4.63E-08 | 3.32E-09 | 0.86 | 5.79E-05 | 7.02E-06 | 1.02 |
| 206316_s_at | KNTC1 | 1.60E-17 | 2.09E-19 | 1.41 | 3.90E-08 | 2.75E-09 | 0.73 | 5.46E-05 | 6.57E-06 | 0.71 |
| 236641_at | KIF14 | 1.79E-17 | 2.36E-19 | 1.42 | 9.41E-09 | 5.86E-10 | 1.18 | 1.30E-09 | 3.76E-11 | 1.72 |
| 225765_at | TNPO1 | 2.84E-21 | 9.36E-24 | 1.42 | 6.51E-19 | 3.81E-21 | 1.24 | 5.76E-19 | 7.58E-22 | 1.19 |
| 210115_at | RPL39L | 7.18E-13 | 2.99E-14 | 1.42 | 5.60E-11 | 2.12E-12 | 0.98 | 3.76E-05 | 4.32E-06 | 1.15 |
| 204078_at | LEPREL4 | 1.31E-18 | 1.22E-20 | 1.43 | 3.11E-15 | 4.56E-17 | 1.04 | 7.95E-07 | 5.39E-08 | 1.07 |
| 201897_s_at | CKS1B | 9.28E-19 | 8.10E-21 | 1.43 | 2.17E-10 | 9.45E-12 | 0.84 | 2.05E-06 | 1.58E-07 | 0.94 |
| 227224_at | RALGPS2 | 1.02E-14 | 2.74E-16 | 1.44 | 4.02E-09 | 2.32E-10 | 0.89 | 1.09E-13 | 8.43E-16 | 1.76 |
| 214073_at | CTTN | 4.64E-14 | 1.46E-15 | 1.44 | 7.41E-15 | 1.18E-16 | 1.13 | 1.22E-10 | 2.54E-12 | 1.47 |
| 201830_s_at | NET1 | 7.72E-14 | 2.57E-15 | 1.44 | 8.26E-05 | 1.30E-05 | 0.83 | 3.50E-08 | 1.55E-09 | 0.98 |
| 209772_s_at | CD24 | 3.46E-10 | 2.52E-11 | 1.45 | 5.52E-11 | 2.09E-12 | 1.57 | 4.68E-10 | 1.19E-11 | 1.85 |
| 218280_x_at | HIST2H2AA4///HIST2H2AA3 | 1.56E-14 | 4.42E-16 | 1.45 | 6.01E-07 | 5.60E-08 | 0.81 | 2.04E-06 | 1.57E-07 | 1.31 |
| 220334_at | RGS17 | 7.49E-12 | 3.89E-13 | 1.45 | 4.02E-14 | 7.49E-16 | 1.91 | 3.75E-09 | 1.26E-10 | 2.71 |
| 237625_s_at |  | 2.41E-07 | 3.13E-08 | 1.45 | 1.32E-05 | 1.71E-06 | 0.77 | 4.60E-05 | 5.41E-06 | 1.97 |
| 212020_s_at | MKI67 | 8.57E-18 | 1.01E-19 | 1.46 | 2.61E-09 | 1.44E-10 | 0.80 | 4.09E-07 | 2.51E-08 | 0.91 |
| 228143_at | CP | 5.30E-06 | 9.02E-07 | 1.46 | 9.78E-12 | 3.12E-13 | 2.24 | 3.82E-07 | 2.32E-08 | 2.51 |
| 207265_s_at | KDELR3 | 9.09E-14 | 3.08E-15 | 1.46 | 3.50E-10 | 1.59E-11 | 1.38 | 1.07E-06 | 7.52E-08 | 0.91 |
| 204126_s_at | CDC45 | 2.83E-16 | 5.24E-18 | 1.47 | 1.47E-09 | 7.66E-11 | 0.79 | 2.01E-05 | 2.12E-06 | 2.33 |
| 222848_at | CENPK | 9.38E-16 | 1.99E-17 | 1.47 | 1.56E-08 | 1.01E-09 | 1.18 | 1.44E-06 | 1.06E-07 | 1.05 |
| 212488_at | COL5A1 | 2.31E-11 | 1.32E-12 | 1.47 | 1.83E-13 | 3.90E-15 | 1.56 | 9.52E-06 | 9.05E-07 | 1.16 |
| 212141_at | MCM4 | 3.38E-19 | 2.50E-21 | 1.47 | 1.87E-08 | 1.24E-09 | 0.80 | 2.07E-06 | 1.59E-07 | 1.24 |
| 229553_at | PGM2L1 | 3.97E-15 | 9.79E-17 | 1.47 | 1.57E-14 | 2.68E-16 | 1.49 | 1.52E-10 | 3.26E-12 | 1.33 |
| 226661_at | CDCA2 | 9.26E-19 | 8.06E-21 | 1.47 | 9.22E-09 | 5.73E-10 | 0.84 | 3.85E-07 | 2.35E-08 | 1.31 |
| 218644_at | PLEK2 | 1.07E-16 | 1.74E-18 | 1.49 | 7.69E-14 | 1.52E-15 | 1.11 | 7.64E-10 | 2.07E-11 | 1.48 |
| 225314_at | OCIAD2 | 1.91E-20 | 8.89E-23 | 1.49 | 1.18E-25 | 9.70E-29 | 1.79 | 5.59E-23 | 9.01E-27 | 2.08 |
| 205046_at | CENPE | 2.99E-16 | 5.56E-18 | 1.49 | 5.93E-07 | 5.50E-08 | 0.77 | 1.20E-06 | 8.63E-08 | 1.44 |
| 238103_at | LINC01207 | 3.38E-06 | 5.52E-07 | 1.49 | 3.16E-09 | 1.78E-10 | 1.63 | 3.75E-05 | 4.30E-06 | 2.33 |
| 209433_s_at | PPAT | 5.21E-12 | 2.61E-13 | 1.49 | 1.09E-08 | 6.86E-10 | 1.01 | 6.00E-10 | 1.56E-11 | 1.06 |
| 202338_at | TK1 | 3.99E-18 | 4.28E-20 | 1.50 | 4.66E-12 | 1.38E-13 | 0.98 | 2.04E-09 | 6.30E-11 | 1.66 |
| 222587_s_at | GALNT7 | 7.15E-15 | 1.88E-16 | 1.50 | 8.44E-15 | 1.36E-16 | 1.46 | 7.70E-14 | 5.73E-16 | 1.30 |
| 214290_s_at | HIST2H2AA4///HIST2H2AA3 | 3.27E-15 | 7.93E-17 | 1.50 | 3.46E-06 | 3.86E-07 | 0.83 | 3.72E-06 | 3.11E-07 | 1.28 |
| 204179_at | MB | 5.60E-12 | 2.82E-13 | 1.50 | 3.88E-07 | 3.44E-08 | 1.00 | 2.07E-06 | 1.59E-07 | 2.08 |
| 228323_at | CASC5 | 1.13E-16 | 1.87E-18 | 1.51 | 5.67E-09 | 3.36E-10 | 1.03 | 1.68E-07 | 9.08E-09 | 1.28 |
| 217755_at | HN1 | 4.61E-22 | 1.07E-24 | 1.52 | 4.00E-13 | 9.32E-15 | 1.06 | 7.00E-07 | 4.65E-08 | 1.41 |
| 205997_at | ADAM28 | 2.87E-11 | 1.67E-12 | 1.52 | 1.79E-09 | 9.53E-11 | 1.35 | 3.74E-08 | 1.67E-09 | 1.56 |
| 224753_at | CDCA5 | 6.42E-18 | 7.30E-20 | 1.53 | 9.89E-12 | 3.16E-13 | 0.96 | 7.88E-09 | 2.86E-10 | 1.17 |
| 227372_s_at | BAIAP2L1 | 9.48E-15 | 2.55E-16 | 1.53 | 1.64E-13 | 3.45E-15 | 1.21 | 3.89E-10 | 9.62E-12 | 1.48 |
| 232278_s_at | DEPDC1 | 5.17E-14 | 1.65E-15 | 1.54 | 5.98E-08 | 4.42E-09 | 0.86 | 9.28E-08 | 4.66E-09 | 2.46 |
| 214455_at | HIST1H2BC | 1.49E-11 | 8.17E-13 | 1.55 | 5.42E-05 | 8.11E-06 | 0.92 | 9.18E-05 | 1.19E-05 | 1.42 |
| 201467_s_at | NQO1 | 3.46E-10 | 2.52E-11 | 1.55 | 1.76E-10 | 7.50E-12 | 1.57 | 3.40E-07 | 2.03E-08 | 1.94 |
| 202949_s_at | FHL2 | 1.18E-13 | 4.11E-15 | 1.56 | 1.23E-10 | 5.07E-12 | 1.16 | 5.70E-06 | 5.07E-07 | 1.37 |
| 1552797_s_at | PROM2 | 4.28E-12 | 2.10E-13 | 1.56 | 1.03E-24 | 1.19E-27 | 2.59 | 8.58E-14 | 6.48E-16 | 2.48 |
| 203145_at | SPAG5 | 2.61E-18 | 2.68E-20 | 1.56 | 2.85E-11 | 1.01E-12 | 0.97 | 1.02E-08 | 3.83E-10 | 1.31 |
| 203953_s_at | CLDN3 | 2.49E-08 | 2.64E-09 | 1.56 | 6.80E-06 | 8.23E-07 | 1.40 | 2.60E-06 | 2.07E-07 | 1.63 |
| 202148_s_at | PYCR1 | 1.16E-25 | 4.44E-29 | 1.56 | 3.84E-24 | 5.63E-27 | 1.31 | 5.07E-18 | 9.19E-21 | 1.47 |
| 221520_s_at | CDCA8 | 7.86E-21 | 3.22E-23 | 1.57 | 1.31E-11 | 4.29E-13 | 0.79 | 1.19E-13 | 9.31E-16 | 2.50 |
| 211519_s_at | KIF2C | 2.08E-18 | 2.06E-20 | 1.57 | 5.19E-10 | 2.45E-11 | 0.93 | 5.18E-07 | 3.32E-08 | 1.15 |
| 222039_at | KIF18B | 2.24E-17 | 3.02E-19 | 1.57 | 1.10E-08 | 6.91E-10 | 0.82 | 1.80E-08 | 7.27E-10 | 1.38 |
| 235609_at | BRIP1 | 7.37E-16 | 1.52E-17 | 1.58 | 9.98E-11 | 4.02E-12 | 1.07 | 2.16E-10 | 4.85E-12 | 1.46 |
| 241994_at | XDH | 1.88E-08 | 1.95E-09 | 1.58 | 6.33E-18 | 4.80E-20 | 2.27 | 2.14E-15 | 1.00E-17 | 3.32 |
| 204023_at | RFC4 | 1.21E-15 | 2.64E-17 | 1.59 | 1.69E-09 | 8.97E-11 | 0.88 | 2.84E-05 | 3.13E-06 | 0.71 |
| 203554_x_at | PTTG1 | 1.47E-15 | 3.29E-17 | 1.59 | 3.17E-11 | 1.14E-12 | 1.35 | 1.01E-06 | 7.10E-08 | 1.28 |
| 226287_at | CCDC34 | 3.53E-17 | 4.98E-19 | 1.59 | 5.13E-11 | 1.93E-12 | 1.00 | 1.86E-06 | 1.42E-07 | 0.89 |
| 205959_at | MMP13 | 7.07E-08 | 8.27E-09 | 1.60 | 1.16E-10 | 4.77E-12 | 1.84 | 6.90E-09 | 2.48E-10 | 4.07 |
| 231311_at |  | 4.90E-17 | 7.17E-19 | 1.61 | 2.78E-16 | 3.19E-18 | 1.37 | 9.30E-15 | 5.10E-17 | 1.68 |
| 228956_at | UGT8 | 1.48E-10 | 9.95E-12 | 1.61 | 8.33E-11 | 3.29E-12 | 1.77 | 6.02E-05 | 7.35E-06 | 1.69 |
| 223484_at | C15orf48 | 1.54E-06 | 2.34E-07 | 1.62 | 3.32E-06 | 3.68E-07 | 1.27 | 2.52E-06 | 2.00E-07 | 1.83 |
| 201014_s_at | PAICS | 3.21E-17 | 4.49E-19 | 1.62 | 1.92E-10 | 8.28E-12 | 1.08 | 2.32E-09 | 7.28E-11 | 1.09 |
| 203878_s_at | MMP11 | 1.59E-15 | 3.59E-17 | 1.63 | 3.61E-17 | 3.31E-19 | 2.28 | 3.61E-12 | 4.46E-14 | 3.21 |
| 203967_at | CDC6 | 1.61E-16 | 2.81E-18 | 1.63 | 1.49E-08 | 9.66E-10 | 1.08 | 5.84E-05 | 7.08E-06 | 1.57 |
| 205339_at | STIL | 3.85E-20 | 2.10E-22 | 1.63 | 3.70E-14 | 6.84E-16 | 1.22 | 9.10E-13 | 9.00E-15 | 1.60 |
| 1554696_s_at | TYMS | 3.66E-14 | 1.14E-15 | 1.64 | 3.10E-09 | 1.74E-10 | 1.08 | 2.47E-06 | 1.95E-07 | 1.00 |
| 218498_s_at | ERO1L | 1.62E-16 | 2.83E-18 | 1.64 | 1.53E-13 | 3.20E-15 | 1.20 | 4.64E-09 | 1.60E-10 | 1.39 |
| 204430_s_at | SLC2A5 | 2.78E-13 | 1.05E-14 | 1.64 | 9.45E-10 | 4.73E-11 | 1.35 | 1.17E-08 | 4.48E-10 | 1.80 |
| 203228_at | PAFAH1B3 | 1.50E-26 | 2.46E-30 | 1.65 | 6.20E-19 | 3.60E-21 | 1.08 | 7.48E-10 | 2.02E-11 | 1.05 |
| 206224_at | CST1 | 2.57E-08 | 2.74E-09 | 1.65 | 6.93E-20 | 3.08E-22 | 2.99 | 5.09E-10 | 1.30E-11 | 3.01 |
| 204146_at | RAD51AP1 | 3.59E-16 | 6.80E-18 | 1.65 | 5.78E-08 | 4.26E-09 | 0.96 | 1.57E-05 | 1.60E-06 | 1.10 |
| 204709_s_at | KIF23 | 1.01E-14 | 2.73E-16 | 1.66 | 1.45E-07 | 1.17E-08 | 0.84 | 6.01E-05 | 7.34E-06 | 1.20 |
| 218663_at | NCAPG | 1.17E-17 | 1.46E-19 | 1.66 | 4.33E-10 | 2.01E-11 | 1.26 | 2.85E-08 | 1.23E-09 | 1.75 |
| 201577_at | NME1 | 1.55E-21 | 4.55E-24 | 1.67 | 1.94E-16 | 2.14E-18 | 1.15 | 2.12E-08 | 8.76E-10 | 1.08 |
| 231647_s_at | FCRL5 | 2.18E-08 | 2.29E-09 | 1.67 | 7.91E-06 | 9.70E-07 | 0.87 | 3.84E-05 | 4.42E-06 | 1.97 |
| 203256_at | CDH3 | 4.08E-11 | 2.44E-12 | 1.67 | 5.92E-20 | 2.60E-22 | 1.98 | 7.21E-14 | 5.29E-16 | 2.53 |
| 203559_s_at | AOC1 | 4.59E-08 | 5.15E-09 | 1.68 | 2.65E-07 | 2.26E-08 | 1.38 | 1.95E-06 | 1.49E-07 | 2.18 |
| 203358_s_at | EZH2 | 6.98E-16 | 1.43E-17 | 1.69 | 7.43E-10 | 3.63E-11 | 1.20 | 1.26E-06 | 9.08E-08 | 1.13 |
| 219010_at | C1orf106 | 2.25E-17 | 3.03E-19 | 1.69 | 1.53E-13 | 3.20E-15 | 1.65 | 1.73E-12 | 1.87E-14 | 1.92 |
| 208650_s_at | CD24 | 6.03E-11 | 3.75E-12 | 1.70 | 2.51E-11 | 8.82E-13 | 1.90 | 9.67E-15 | 5.32E-17 | 2.85 |
| 224839_s_at | GPT2 | 1.47E-17 | 1.89E-19 | 1.70 | 3.16E-15 | 4.64E-17 | 1.38 | 4.91E-13 | 4.59E-15 | 1.46 |
| 213909_at | LRRC15 | 1.37E-08 | 1.38E-09 | 1.70 | 1.13E-11 | 3.65E-13 | 1.76 | 2.73E-05 | 2.99E-06 | 2.08 |
| 210519_s_at | NQO1 | 7.47E-11 | 4.71E-12 | 1.70 | 1.56E-13 | 3.28E-15 | 1.73 | 4.06E-07 | 2.49E-08 | 1.69 |
| 1554408_a_at | TK1 | 4.52E-17 | 6.55E-19 | 1.70 | 3.17E-11 | 1.13E-12 | 1.14 | 1.73E-06 | 1.30E-07 | 0.99 |
| 213523_at | CCNE1 | 1.46E-16 | 2.49E-18 | 1.71 | 1.02E-09 | 5.14E-11 | 1.00 | 1.45E-05 | 1.45E-06 | 1.17 |
| 60474_at | FERMT1 | 1.94E-13 | 7.08E-15 | 1.71 | 2.54E-12 | 7.04E-14 | 1.54 | 1.41E-10 | 3.00E-12 | 1.74 |
| 219232_s_at | EGLN3 | 1.16E-11 | 6.29E-13 | 1.71 | 4.34E-07 | 3.89E-08 | 1.06 | 3.48E-05 | 3.94E-06 | 1.18 |
| 218883_s_at | CENPU | 1.35E-14 | 3.78E-16 | 1.71 | 3.29E-12 | 9.38E-14 | 1.49 | 5.05E-07 | 3.22E-08 | 1.30 |
| 215108_x_at | TOX3 | 2.07E-08 | 2.17E-09 | 1.71 | 1.40E-18 | 8.82E-21 | 2.75 | 7.93E-12 | 1.12E-13 | 3.10 |
| 202107_s_at | MCM2 | 5.07E-17 | 7.44E-19 | 1.71 | 1.37E-14 | 2.33E-16 | 1.11 | 1.60E-07 | 8.62E-09 | 1.06 |
| 228347_at | SIX1 | 2.24E-07 | 2.89E-08 | 1.72 | 1.48E-11 | 4.93E-13 | 2.06 | 2.20E-09 | 6.85E-11 | 2.69 |
| 219306_at | KIF15 | 6.93E-18 | 7.96E-20 | 1.73 | 1.94E-08 | 1.29E-09 | 1.04 | 3.24E-06 | 2.66E-07 | 1.11 |
| 222740_at | ATAD2 | 2.62E-15 | 6.24E-17 | 1.73 | 3.88E-09 | 2.23E-10 | 0.96 | 1.97E-07 | 1.08E-08 | 1.09 |
| 203213_at | CDK1 | 3.90E-17 | 5.57E-19 | 1.74 | 1.22E-10 | 5.04E-12 | 1.17 | 2.42E-06 | 1.91E-07 | 1.21 |
| 204675_at | SRD5A1 | 1.13E-17 | 1.41E-19 | 1.74 | 4.84E-18 | 3.54E-20 | 1.85 | 4.35E-15 | 2.18E-17 | 1.85 |
| 205394_at | CHEK1 | 1.17E-16 | 1.94E-18 | 1.75 | 1.59E-08 | 1.04E-09 | 1.01 | 3.10E-07 | 1.84E-08 | 1.54 |
| 209911_x_at | HIST1H2BD | 1.45E-17 | 1.86E-19 | 1.75 | 1.84E-14 | 3.20E-16 | 1.44 | 5.45E-08 | 2.56E-09 | 1.43 |
| 213007_at | FANCI | 3.99E-17 | 5.72E-19 | 1.75 | 3.58E-11 | 1.30E-12 | 1.04 | 4.44E-07 | 2.77E-08 | 1.16 |
| 238593_at | C11orf80 | 1.12E-16 | 1.83E-18 | 1.75 | 4.42E-07 | 3.96E-08 | 1.20 | 3.26E-10 | 7.82E-12 | 1.40 |
| 226456_at | RMI2 | 6.87E-17 | 1.05E-18 | 1.75 | 7.24E-21 | 2.50E-23 | 1.53 | 6.17E-17 | 1.67E-19 | 1.99 |
| 222067_x_at | HIST1H2BD | 2.57E-20 | 1.30E-22 | 1.75 | 1.08E-15 | 1.42E-17 | 1.24 | 2.82E-08 | 1.21E-09 | 1.77 |
| 209709_s_at | HMMR | 4.75E-17 | 6.93E-19 | 1.76 | 1.71E-12 | 4.55E-14 | 1.25 | 3.14E-10 | 7.46E-12 | 1.78 |
| 209529_at | PPAP2C | 8.89E-16 | 1.87E-17 | 1.77 | 4.72E-20 | 2.00E-22 | 1.90 | 5.53E-17 | 1.48E-19 | 2.21 |
| 238439_at | ANKRD22 | 8.04E-10 | 6.31E-11 | 1.77 | 1.09E-14 | 1.79E-16 | 2.00 | 1.17E-15 | 4.97E-18 | 2.44 |
| 209955_s_at | FAP | 2.29E-13 | 8.50E-15 | 1.78 | 6.05E-10 | 2.90E-11 | 1.36 | 4.27E-07 | 2.64E-08 | 1.32 |
| 229802_at | WISP1 | 2.31E-09 | 1.99E-10 | 1.79 | 9.36E-07 | 9.12E-08 | 1.38 | 9.98E-06 | 9.53E-07 | 1.36 |
| 218796_at | FERMT1 | 8.44E-14 | 2.84E-15 | 1.79 | 2.06E-14 | 3.60E-16 | 1.64 | 4.89E-11 | 8.97E-13 | 2.05 |
| 218326_s_at | LGR4 | 5.87E-16 | 1.17E-17 | 1.81 | 2.32E-15 | 3.29E-17 | 1.82 | 3.80E-06 | 3.19E-07 | 1.25 |
| 218782_s_at | ATAD2 | 1.46E-16 | 2.50E-18 | 1.81 | 5.78E-06 | 6.86E-07 | 0.75 | 1.60E-07 | 8.59E-09 | 1.09 |
| 218662_s_at | NCAPG | 1.25E-17 | 1.56E-19 | 1.82 | 3.42E-12 | 9.79E-14 | 1.30 | 1.01E-06 | 7.08E-08 | 1.41 |
| 203936_s_at | MMP9 | 5.92E-10 | 4.52E-11 | 1.83 | 4.13E-06 | 4.71E-07 | 1.44 | 3.82E-08 | 1.71E-09 | 2.34 |
| 203214_x_at | CDK1 | 6.63E-16 | 1.35E-17 | 1.83 | 2.72E-09 | 1.51E-10 | 1.13 | 5.66E-07 | 3.66E-08 | 1.23 |
| 223307_at | CDCA3 | 4.50E-19 | 3.49E-21 | 1.84 | 1.68E-13 | 3.56E-15 | 1.13 | 2.31E-07 | 1.30E-08 | 1.48 |
| 204092_s_at | AURKA | 4.10E-20 | 2.26E-22 | 1.86 | 8.98E-13 | 2.25E-14 | 1.21 | 4.54E-08 | 2.08E-09 | 1.40 |
| 221521_s_at | GINS2 | 2.57E-20 | 1.30E-22 | 1.86 | 1.74E-12 | 4.63E-14 | 1.12 | 9.73E-09 | 3.63E-10 | 1.50 |
| 219787_s_at | ECT2 | 1.45E-16 | 2.46E-18 | 1.86 | 5.43E-09 | 3.21E-10 | 1.28 | 7.31E-07 | 4.90E-08 | 1.08 |
| 238741_at | FAM83A | 4.67E-16 | 9.07E-18 | 1.87 | 3.11E-14 | 5.65E-16 | 0.80 | 2.28E-05 | 2.44E-06 | 1.39 |
| 223779_at | AFAP1-AS1 | 2.08E-11 | 1.18E-12 | 1.88 | 4.44E-18 | 3.19E-20 | 2.50 | 2.97E-13 | 2.57E-15 | 3.99 |
| 204444_at | KIF11 | 2.53E-17 | 3.48E-19 | 1.88 | 1.97E-12 | 5.31E-14 | 1.48 | 1.03E-06 | 7.26E-08 | 1.25 |
| 219990_at | E2F8 | 8.32E-18 | 9.76E-20 | 1.89 | 1.85E-12 | 4.98E-14 | 1.36 | 3.19E-12 | 3.86E-14 | 2.33 |
| 202404_s_at | COL1A2 | 4.59E-13 | 1.84E-14 | 1.90 | 4.44E-15 | 6.75E-17 | 1.83 | 2.07E-07 | 1.14E-08 | 0.84 |
| 204653_at | TFAP2A | 2.84E-11 | 1.65E-12 | 1.90 | 1.99E-16 | 2.20E-18 | 1.78 | 7.22E-13 | 6.97E-15 | 2.64 |
| 212021_s_at | MKI67 | 1.83E-18 | 1.79E-20 | 1.90 | 4.45E-08 | 3.19E-09 | 0.89 | 1.14E-07 | 5.91E-09 | 1.30 |
| 214774_x_at | TOX3 | 5.09E-08 | 5.77E-09 | 1.91 | 5.80E-20 | 2.54E-22 | 3.20 | 2.19E-12 | 2.45E-14 | 3.22 |
| 219888_at | SPAG4 | 2.98E-13 | 1.14E-14 | 1.92 | 5.11E-16 | 6.33E-18 | 1.52 | 5.80E-07 | 3.75E-08 | 1.49 |
| 203418_at | CCNA2 | 1.01E-17 | 1.23E-19 | 1.92 | 1.81E-10 | 7.77E-12 | 1.17 | 5.61E-12 | 7.48E-14 | 2.12 |
| 227253_at | CP | 7.92E-08 | 9.35E-09 | 1.92 | 1.21E-10 | 4.98E-12 | 2.37 | 1.27E-08 | 4.89E-10 | 2.91 |
| 216623_x_at | TOX3 | 4.22E-08 | 4.69E-09 | 1.93 | 2.13E-19 | 1.09E-21 | 3.11 | 3.12E-13 | 2.71E-15 | 3.10 |
| 202580_x_at | FOXM1 | 8.69E-18 | 1.03E-19 | 1.93 | 2.57E-10 | 1.13E-11 | 1.18 | 1.56E-06 | 1.16E-07 | 1.63 |
| 218726_at | HJURP | 2.92E-21 | 9.84E-24 | 1.94 | 1.28E-10 | 5.32E-12 | 1.00 | 2.25E-07 | 1.26E-08 | 1.57 |
| 211161_s_at | COL3A1 | 1.35E-12 | 5.94E-14 | 1.95 | 4.16E-18 | 2.96E-20 | 1.98 | 9.19E-09 | 3.41E-10 | 0.80 |
| 1554768_a_at | MAD2L1 | 4.04E-15 | 1.00E-16 | 1.95 | 1.02E-05 | 1.28E-06 | 1.00 | 1.00E-07 | 5.10E-09 | 1.51 |
| 235456_at |  | 2.48E-13 | 9.25E-15 | 1.95 | 6.37E-10 | 3.07E-11 | 1.72 | 1.11E-06 | 7.89E-08 | 1.74 |
| 225803_at | FBXO32 | 2.97E-22 | 6.36E-25 | 1.97 | 7.31E-19 | 4.32E-21 | 1.39 | 6.46E-08 | 3.10E-09 | 1.37 |
| 218755_at | KIF20A | 1.27E-17 | 1.61E-19 | 1.97 | 3.21E-15 | 4.73E-17 | 1.68 | 2.79E-08 | 1.20E-09 | 1.53 |
| 216548_x_at | HMGB3P1 | 4.04E-18 | 4.35E-20 | 1.98 | 2.12E-15 | 3.00E-17 | 1.31 | 2.32E-06 | 1.82E-07 | 1.07 |
| 201852_x_at | COL3A1 | 1.05E-11 | 5.63E-13 | 1.98 | 3.14E-13 | 7.10E-15 | 1.75 | 6.51E-06 | 5.91E-07 | 1.02 |
| 229479_at |  | 1.61E-15 | 3.64E-17 | 1.99 | 5.88E-12 | 1.78E-13 | 1.89 | 4.95E-06 | 4.30E-07 | 1.99 |
| 202589_at | TYMS | 5.04E-15 | 1.28E-16 | 1.99 | 8.06E-13 | 1.99E-14 | 1.53 | 3.69E-05 | 4.22E-06 | 1.06 |
| 222847_s_at | EGLN3 | 4.50E-16 | 8.68E-18 | 1.99 | 1.50E-08 | 9.74E-10 | 0.97 | 4.40E-05 | 5.15E-06 | 1.60 |
| 204026_s_at | ZWINT | 6.02E-17 | 9.04E-19 | 2.00 | 4.97E-18 | 3.66E-20 | 1.77 | 3.64E-08 | 1.62E-09 | 1.54 |
| 203083_at | THBS2 | 7.17E-13 | 2.98E-14 | 2.00 | 1.25E-16 | 1.31E-18 | 2.13 | 4.29E-10 | 1.08E-11 | 1.91 |
| 206364_at | KIF14 | 6.39E-19 | 5.19E-21 | 2.01 | 8.24E-13 | 2.04E-14 | 1.17 | 1.11E-06 | 7.86E-08 | 1.25 |
| 212022_s_at | MKI67 | 3.65E-18 | 3.87E-20 | 2.03 | 7.75E-11 | 3.05E-12 | 1.10 | 6.69E-10 | 1.77E-11 | 1.88 |
| 204846_at | CP | 1.50E-08 | 1.52E-09 | 2.03 | 4.12E-10 | 1.91E-11 | 2.37 | 4.64E-09 | 1.60E-10 | 3.13 |
| 228729_at | CCNB1 | 1.54E-18 | 1.47E-20 | 2.04 | 7.59E-13 | 1.86E-14 | 1.65 | 2.72E-08 | 1.16E-09 | 1.49 |
| 1552619_a_at | ANLN | 1.20E-16 | 2.01E-18 | 2.05 | 5.10E-10 | 2.41E-11 | 1.37 | 4.09E-08 | 1.85E-09 | 1.97 |
| 209642_at | BUB1 | 5.22E-17 | 7.71E-19 | 2.06 | 1.19E-12 | 3.07E-14 | 1.44 | 1.08E-07 | 5.51E-09 | 1.41 |
| 222958_s_at | DEPDC1 | 7.89E-19 | 6.74E-21 | 2.06 | 1.85E-10 | 7.94E-12 | 1.16 | 1.67E-11 | 2.68E-13 | 2.30 |
| 222646_s_at | ERO1L | 2.07E-17 | 2.76E-19 | 2.06 | 6.00E-14 | 1.16E-15 | 1.58 | 3.55E-08 | 1.57E-09 | 1.47 |
| 219978_s_at | NUSAP1 | 8.15E-18 | 9.52E-20 | 2.07 | 4.58E-05 | 6.75E-06 | 0.75 | 4.02E-10 | 1.00E-11 | 1.49 |
| 208079_s_at | AURKA | 1.55E-19 | 1.02E-21 | 2.08 | 1.28E-11 | 4.20E-13 | 1.32 | 5.24E-08 | 2.45E-09 | 1.56 |
| 219508_at | GCNT3 | 6.99E-13 | 2.90E-14 | 2.10 | 4.25E-22 | 1.06E-24 | 2.47 | 4.92E-16 | 1.85E-18 | 4.11 |
| 227452_at | LINC00673 | 3.57E-16 | 6.75E-18 | 2.11 | 1.48E-22 | 3.18E-25 | 2.25 | 1.57E-14 | 9.60E-17 | 2.54 |
| 238617_at |  | 1.14E-15 | 2.47E-17 | 2.11 | 5.11E-19 | 2.84E-21 | 2.10 | 4.21E-14 | 2.90E-16 | 2.37 |
| 201884_at | CEACAM5 | 1.28E-05 | 2.36E-06 | 2.12 | 3.26E-09 | 1.84E-10 | 2.59 | 8.11E-06 | 7.57E-07 | 3.20 |
| 226777_at | ADAM12 | 1.75E-14 | 5.04E-16 | 2.12 | 2.33E-11 | 8.10E-13 | 1.98 | 1.19E-05 | 1.17E-06 | 1.74 |
| 218039_at | NUSAP1 | 1.31E-17 | 1.66E-19 | 2.15 | 2.86E-15 | 4.16E-17 | 1.68 | 5.05E-09 | 1.75E-10 | 1.51 |
| 239586_at | FAM83A | 1.47E-18 | 1.39E-20 | 2.17 | 7.19E-14 | 1.42E-15 | 0.87 | 4.21E-08 | 1.91E-09 | 1.88 |
| 209408_at | KIF2C | 2.12E-20 | 1.01E-22 | 2.17 | 2.75E-12 | 7.67E-14 | 1.37 | 1.50E-10 | 3.20E-12 | 1.77 |
| 242517_at | KISS1R | 8.97E-13 | 3.81E-14 | 2.20 | 4.99E-08 | 3.62E-09 | 1.27 | 1.01E-09 | 2.82E-11 | 2.24 |
| 204962_s_at | CENPA | 4.74E-18 | 5.18E-20 | 2.20 | 7.16E-12 | 2.22E-13 | 1.29 | 5.57E-08 | 2.63E-09 | 1.79 |
| 218960_at | TMPRSS4 | 3.99E-17 | 5.72E-19 | 2.21 | 8.74E-17 | 8.79E-19 | 2.32 | 1.90E-22 | 4.52E-26 | 5.18 |
| 209035_at | MDK | 5.29E-20 | 3.05E-22 | 2.22 | 2.27E-17 | 2.00E-19 | 1.55 | 1.01E-09 | 2.82E-11 | 1.55 |
| 212354_at | SULF1 | 4.48E-18 | 4.86E-20 | 2.22 | 6.41E-14 | 1.25E-15 | 1.80 | 9.17E-07 | 6.32E-08 | 1.83 |
| 212344_at | SULF1 | 5.00E-19 | 3.93E-21 | 2.24 | 8.78E-13 | 2.19E-14 | 1.55 | 4.10E-07 | 2.52E-08 | 1.95 |
| 207165_at | HMMR | 1.17E-17 | 1.45E-19 | 2.25 | 1.47E-15 | 2.01E-17 | 1.90 | 2.24E-12 | 2.52E-14 | 2.35 |
| 203755_at | BUB1B | 1.08E-19 | 6.74E-22 | 2.25 | 6.17E-15 | 9.70E-17 | 1.77 | 2.21E-09 | 6.90E-11 | 1.81 |
| 201250_s_at | SLC2A1 | 3.44E-20 | 1.81E-22 | 2.28 | 5.65E-12 | 1.71E-13 | 1.46 | 6.55E-08 | 3.15E-09 | 1.49 |
| 209714_s_at | CDKN3 | 4.75E-18 | 5.19E-20 | 2.28 | 8.87E-12 | 2.80E-13 | 1.47 | 3.40E-06 | 2.80E-07 | 1.37 |
| 217771_at | GOLM1 | 1.91E-24 | 1.32E-27 | 2.29 | 7.18E-31 | 1.31E-34 | 2.07 | 1.36E-19 | 1.36E-22 | 2.21 |
| 203362_s_at | MAD2L1 | 1.87E-19 | 1.26E-21 | 2.30 | 5.44E-10 | 2.58E-11 | 1.45 | 3.94E-07 | 2.41E-08 | 1.36 |
| 242881_x_at |  | 1.14E-15 | 2.46E-17 | 2.31 | 3.29E-08 | 2.29E-09 | 1.48 | 7.48E-06 | 6.92E-07 | 1.54 |
| 1558034_s_at | CP | 1.40E-10 | 9.36E-12 | 2.32 | 2.87E-08 | 1.98E-09 | 2.08 | 3.28E-09 | 1.09E-10 | 3.26 |
| 225601_at | HMGB3 | 2.54E-15 | 6.03E-17 | 2.32 | 2.13E-13 | 4.62E-15 | 1.81 | 2.20E-07 | 1.22E-08 | 1.61 |
| 229490_s_at |  | 7.37E-19 | 6.19E-21 | 2.33 | 3.24E-12 | 9.22E-14 | 1.22 | 2.35E-07 | 1.33E-08 | 1.73 |
| 207828_s_at | CENPF | 5.88E-21 | 2.27E-23 | 2.33 | 2.30E-14 | 4.08E-16 | 1.62 | 5.78E-09 | 2.04E-10 | 1.75 |
| 202575_at | CRABP2 | 6.84E-17 | 1.04E-18 | 2.33 | 7.44E-19 | 4.41E-21 | 2.30 | 1.27E-08 | 4.89E-10 | 2.79 |
| 205941_s_at | COL10A1 | 1.26E-17 | 1.59E-19 | 2.36 | 1.27E-30 | 3.01E-34 | 3.80 | 5.59E-23 | 8.77E-27 | 4.13 |
| 202705_at | CCNB2 | 1.08E-20 | 4.62E-23 | 2.36 | 1.78E-14 | 3.09E-16 | 1.60 | 2.55E-09 | 8.12E-11 | 1.98 |
| 218355_at | KIF4A | 2.37E-21 | 7.55E-24 | 2.36 | 2.11E-12 | 5.74E-14 | 1.52 | 2.42E-07 | 1.38E-08 | 1.73 |
| 218542_at | CEP55 | 4.71E-19 | 3.68E-21 | 2.37 | 3.69E-11 | 1.34E-12 | 1.62 | 1.25E-07 | 6.54E-09 | 1.77 |
| 225655_at | UHRF1 | 8.85E-22 | 2.22E-24 | 2.37 | 3.38E-15 | 5.02E-17 | 1.87 | 1.38E-06 | 1.01E-07 | 1.47 |
| 203889_at | SCG5 | 2.10E-15 | 4.89E-17 | 2.39 | 1.45E-12 | 3.82E-14 | 1.77 | 1.61E-06 | 1.20E-07 | 1.84 |
| 1555758_a_at | CDKN3 | 4.06E-18 | 4.37E-20 | 2.39 | 6.84E-09 | 4.13E-10 | 1.46 | 2.85E-05 | 3.14E-06 | 1.35 |
| 223381_at | NUF2 | 3.48E-20 | 1.84E-22 | 2.41 | 1.06E-10 | 4.31E-12 | 1.44 | 1.21E-07 | 6.31E-09 | 1.69 |
| 238460_at | FAM83A | 9.11E-16 | 1.92E-17 | 2.43 | 2.19E-10 | 9.54E-12 | 1.09 | 2.55E-10 | 5.86E-12 | 3.31 |
| 202095_s_at | BIRC5 | 1.35E-19 | 8.67E-22 | 2.43 | 9.91E-14 | 2.00E-15 | 1.66 | 1.72E-08 | 6.90E-10 | 1.90 |
| 223229_at | UBE2T | 1.81E-23 | 2.32E-26 | 2.43 | 8.85E-18 | 6.98E-20 | 1.75 | 1.87E-15 | 8.56E-18 | 2.46 |
| 206023_at | NMU | 2.70E-12 | 1.26E-13 | 2.45 | 1.30E-14 | 2.18E-16 | 1.54 | 8.20E-07 | 5.59E-08 | 2.19 |
| 206134_at | ADAMDEC1 | 1.87E-09 | 1.59E-10 | 2.46 | 2.68E-05 | 3.75E-06 | 1.70 | 1.30E-07 | 6.84E-09 | 2.39 |
| 223062_s_at | PSAT1 | 2.03E-17 | 2.71E-19 | 2.46 | 5.79E-19 | 3.32E-21 | 2.29 | 3.34E-10 | 8.07E-12 | 1.87 |
| 219148_at | PBK | 1.31E-17 | 1.67E-19 | 2.47 | 3.27E-08 | 2.28E-09 | 1.35 | 6.60E-05 | 8.16E-06 | 1.38 |
| 202310_s_at | COL1A1 | 2.42E-14 | 7.18E-16 | 2.48 | 4.29E-15 | 6.48E-17 | 2.26 | 9.68E-10 | 2.69E-11 | 2.16 |
| 204825_at | MELK | 2.42E-20 | 1.18E-22 | 2.48 | 1.80E-13 | 3.84E-15 | 1.62 | 3.17E-08 | 1.39E-09 | 1.97 |
| 204822_at | TTK | 5.20E-17 | 7.65E-19 | 2.49 | 1.75E-11 | 5.91E-13 | 1.67 | 8.86E-09 | 3.26E-10 | 1.57 |
| 205549_at | PCP4 | 3.91E-10 | 2.88E-11 | 2.49 | 1.52E-09 | 7.97E-11 | 1.31 | 2.49E-06 | 1.98E-07 | 3.14 |
| 212353_at | SULF1 | 1.95E-19 | 1.33E-21 | 2.51 | 6.00E-16 | 7.55E-18 | 2.14 | 6.11E-07 | 3.98E-08 | 1.70 |
| 230030_at | HS6ST2 | 1.03E-12 | 4.45E-14 | 2.52 | 3.37E-20 | 1.37E-22 | 3.39 | 7.88E-15 | 4.21E-17 | 3.71 |
| 206102_at | GINS1 | 1.02E-21 | 2.64E-24 | 2.53 | 6.21E-16 | 7.85E-18 | 1.96 | 1.38E-09 | 4.05E-11 | 1.61 |
| 204641_at | NEK2 | 4.65E-23 | 6.81E-26 | 2.54 | 3.79E-13 | 8.77E-15 | 1.76 | 2.36E-07 | 1.33E-08 | 1.58 |
| 202503_s_at | KIAA0101 | 6.97E-19 | 5.81E-21 | 2.56 | 1.51E-19 | 7.18E-22 | 2.07 | 1.10E-13 | 8.52E-16 | 2.11 |
| 203744_at | HMGB3 | 2.21E-20 | 1.06E-22 | 2.59 | 3.44E-17 | 3.11E-19 | 1.84 | 6.71E-12 | 9.22E-14 | 2.16 |
| 219918_s_at | ASPM | 5.08E-19 | 4.01E-21 | 2.60 | 3.83E-11 | 1.40E-12 | 1.71 | 2.08E-08 | 8.52E-10 | 2.00 |
| 224428_s_at | CDCA7 | 1.29E-19 | 8.20E-22 | 2.60 | 1.73E-17 | 1.48E-19 | 2.39 | 3.03E-15 | 1.45E-17 | 2.56 |
| 201890_at | RRM2 | 2.75E-18 | 2.86E-20 | 2.61 | 5.86E-13 | 1.41E-14 | 1.89 | 3.58E-09 | 1.19E-10 | 1.92 |
| 202954_at | UBE2C | 1.15E-20 | 5.05E-23 | 2.62 | 1.55E-14 | 2.65E-16 | 1.52 | 1.35E-06 | 9.84E-08 | 1.34 |
| 214710_s_at | CCNB1 | 3.69E-20 | 1.97E-22 | 2.64 | 4.58E-14 | 8.68E-16 | 1.72 | 1.54E-08 | 6.07E-10 | 1.56 |
| 1552767_a_at | HS6ST2 | 4.41E-12 | 2.17E-13 | 2.67 | 2.12E-20 | 8.13E-23 | 3.43 | 3.91E-15 | 1.92E-17 | 3.51 |
| 210052_s_at | TPX2 | 4.54E-21 | 1.65E-23 | 2.69 | 9.30E-13 | 2.34E-14 | 1.54 | 1.77E-08 | 7.13E-10 | 1.85 |
| 223278_at | GJB2 | 1.83E-18 | 1.78E-20 | 2.71 | 2.67E-15 | 3.86E-17 | 2.57 | 1.99E-11 | 3.28E-13 | 3.53 |
| 202870_s_at | CDC20 | 4.73E-21 | 1.76E-23 | 2.73 | 1.37E-15 | 1.84E-17 | 1.79 | 1.39E-07 | 7.33E-09 | 1.63 |
| 203764_at | DLGAP5 | 2.33E-19 | 1.63E-21 | 2.77 | 7.80E-14 | 1.55E-15 | 1.91 | 5.68E-09 | 2.00E-10 | 2.08 |
| 202311_s_at | COL1A1 | 1.00E-15 | 2.13E-17 | 2.78 | 1.60E-18 | 1.03E-20 | 3.00 | 2.89E-08 | 1.25E-09 | 1.92 |
| 209773_s_at | RRM2 | 9.39E-17 | 1.49E-18 | 2.86 | 2.07E-14 | 3.62E-16 | 1.94 | 4.68E-10 | 1.19E-11 | 2.10 |
| 206239_s_at | SPINK1 | 2.40E-09 | 2.08E-10 | 3.06 | 1.89E-11 | 6.44E-13 | 3.34 | 5.49E-10 | 1.41E-11 | 3.75 |
| 217428_s_at | COL10A1 | 9.86E-19 | 8.73E-21 | 3.06 | 3.70E-28 | 1.49E-31 | 4.26 | 3.68E-31 | 6.74E-36 | 4.89 |
| 222608_s_at | ANLN | 9.94E-21 | 4.24E-23 | 3.08 | 2.69E-16 | 3.07E-18 | 2.49 | 4.17E-10 | 1.05E-11 | 2.34 |
| 201292_at | TOP2A | 6.35E-21 | 2.46E-23 | 3.10 | 5.36E-20 | 2.30E-22 | 2.64 | 3.22E-12 | 3.91E-14 | 2.43 |
| 225681_at | CTHRC1 | 4.15E-19 | 3.16E-21 | 3.35 | 2.40E-24 | 3.16E-27 | 3.19 | 1.10E-14 | 6.36E-17 | 2.31 |
| 204475_at | MMP1 | 1.34E-11 | 7.29E-13 | 3.37 | 1.25E-10 | 5.19E-12 | 3.04 | 1.82E-05 | 1.88E-06 | 2.95 |
| 201291_s_at | TOP2A | 1.70E-21 | 5.07E-24 | 3.58 | 1.40E-15 | 1.89E-17 | 2.25 | 1.51E-13 | 1.25E-15 | 2.62 |
| 205242_at | CXCL13 | 1.25E-12 | 5.48E-14 | 3.62 | 1.03E-06 | 1.02E-07 | 2.14 | 3.81E-10 | 9.39E-12 | 3.81 |
| 218468_s_at | GREM1 | 1.01E-18 | 9.02E-21 | 3.65 | 6.22E-12 | 1.89E-13 | 2.71 | 6.32E-06 | 5.71E-07 | 2.99 |
| 218469_at | GREM1 | 1.30E-18 | 1.21E-20 | 3.80 | 2.91E-13 | 6.54E-15 | 3.03 | 7.04E-07 | 4.68E-08 | 3.04 |
| 204320_at | COL11A1 | 2.47E-21 | 7.89E-24 | 3.89 | 6.68E-18 | 5.12E-20 | 3.32 | 9.90E-10 | 2.76E-11 | 4.64 |
| 209875_s_at | SPP1 | 1.15E-21 | 3.14E-24 | 4.05 | 1.55E-26 | 1.10E-29 | 4.50 | 1.48E-16 | 4.64E-19 | 3.94 |
| 204580_at | MMP12 | 1.47E-18 | 1.39E-20 | 4.10 | 1.13E-14 | 1.87E-16 | 3.18 | 6.34E-10 | 1.67E-11 | 3.72 |
| 37892_at | COL11A1 | 1.73E-22 | 3.16E-25 | 4.33 | 1.55E-18 | 9.89E-21 | 3.98 | 1.01E-10 | 2.07E-12 | 5.20 |
