# Supplementary material for: A robust gene expression-based prognostic risk score predicts overall survival of lung adenocarcinoma patients
Source: Oncotarget. 2017 Dec 15;9(6):6862–71. doi: 10.18632/oncotarget.23490 (PMC5805521; doi:10.18632/oncotarget.23490)
Supplement: Supplementary file 3 [file oncotarget-09-6862-s003.doc]

| **Supplementary Table 2.** The impact of deregulated genes on overall survival (OS). The genes that are significantly associated with OS are highlighted in yellow. | | | |
| --- | --- | --- | --- |
| **Gene name** | **Affymetrix microarray** | | |
|  | HR (95% CI) | p-value | Adjusted p-value |
| TPX2 | 2.80 (2.18 − 3.60) | < 1.00E-16 | < 1.00E-16 |
| MAMDC2 | 0.34 (0.26 − 0.44) | < 1.00E-16 | < 1.00E-16 |
| BIRC5 | 2.84 (2.21 − 3.65) | < 1.00E-16 | < 1.00E-16 |
| FEN1 | 2.85 (2.21 − 3.67) | < 1.00E-16 | < 1.00E-16 |
| ITPR1 | 0.36 (0.28 − 0.46) | 1.1E-16 | 2.2E-13 |
| CDCA3 | 2.74 (2.13 − 3.52) | 2.2E-16 | 4.4E-13 |
| PLAU | 2.78 (2.16 − 3.58) | 2.2E-16 | 4.4E-13 |
| TK1 | 2.85 (2.19 − 3.70) | 3.3E-16 | 6.5E-13 |
| TTC28 | 0.36 (0.28 − 0.47) | 6.7E-16 | 1.3E-12 |
| HN1 | 2.74 (2.12 − 3.54) | 1.0E-15 | 2.0E-12 |
| INPP5A | 0.37 (0.29 − 0.48) | 1.2E-15 | 2.4E-12 |
| KIF2C | 2.61 (2.04 − 3.34) | 1.9E-15 | 3.8E-12 |
| ANKS1A | 0.37 (0.29 − 0.48) | 2.6E-15 | 5.2E-12 |
| MARC2 | 0.36 (0.28 − 0.47) | 3.2E-15 | 6.3E-12 |
| H3F3B | 0.38 (0.30 − 0.49) | 3.7E-15 | 7.3E-12 |
| NME1 | 2.65 (2.06 − 3.42) | 3.9E-15 | 7.7E-12 |
| CDC6 | 2.59 (2.02 − 3.32) | 5.1E-15 | 1.0E-11 |
| LIMCH1 | 0.39 (0.31 − 0.50) | 6.8E-15 | 1.3E-11 |
| NUSAP1 | 2.54 (1.99 − 3.25) | 1.0E-14 | 2.0E-11 |
| TYMS | 2.68 (2.06 − 3.48) | 1.5E-14 | 3.0E-11 |
| ADARB1 | 0.39 (0.30 − 0.50) | 2.0E-14 | 4.0E-11 |
| GPRASP1 | 0.38 (0.29 − 0.49) | 2.3E-14 | 4.6E-11 |
| FGF2 | 0.39 (0.31 − 0.51) | 2.8E-14 | 5.5E-11 |
| UBE2C | 2.53 (1.97 − 3.23) | 2.8E-14 | 5.5E-11 |
| ASF1B | 2.49 (1.95 − 3.18) | 3.4E-14 | 6.7E-11 |
| PDIA6 | 0.41 (0.32 − 0.52) | 5.9E-14 | 1.2E-10 |
| LONRF1 | 0.38 (0.29 − 0.50) | 6.3E-14 | 1.2E-10 |
| NFIA | 0.38 (0.29 − 0.49) | 6.8E-14 | 1.3E-10 |
| MACF1 | 0.40 (0.31 − 0.51) | 6.9E-14 | 1.4E-10 |
| PIK3R1 | 0.39 (0.31 − 0.51) | 7.9E-14 | 1.6E-10 |
| ABCA8 | 0.40 (0.31 − 0.51) | 1.1E-13 | 2.2E-10 |
| RERG | 0.39 (0.30 − 0.51) | 1.2E-13 | 2.4E-10 |
| CDC25C | 2.42 (1.90 − 3.08) | 1.4E-13 | 2.8E-10 |
| FRY | 0.41 (0.32 − 0.52) | 2.1E-13 | 4.2E-10 |
| MYH11 | 0.40 (0.31 − 0.52) | 2.6E-13 | 5.2E-10 |
| AP1S1 | 2.48 (1.93 − 3.19) | 2.8E-13 | 5.5E-10 |
| RORA | 0.39 (0.30 − 0.51) | 2.9E-13 | 5.7E-10 |
| MYH10 | 0.41 (0.32 − 0.52) | 3.1E-13 | 6.1E-10 |
| DSTN | 0.42 (0.33 − 0.53) | 3.6E-13 | 7.1E-10 |
| HJURP | 2.42 (1.89 − 3.09) | 3.9E-13 | 7.7E-10 |
| KLF6 | 2.47 (1.92 − 3.18) | 4.1E-13 | 8.1E-10 |
| FOXM1 | 2.40 (1.88 − 3.06) | 4.2E-13 | 8.3E-10 |
| CDT1 | 2.39 (1.87 − 3.04) | 4.5E-13 | 8.9E-10 |
| CDKN3 | 2.57 (1.97 − 3.35) | 5.1E-13 | 1.0E-09 |
| RBMS2 | 0.40 (0.31 − 0.51) | 5.6E-13 | 1.1E-09 |
| MYLIP | 0.40 (0.31 − 0.52) | 6.1E-13 | 1.2E-09 |
| PCM1 | 0.40 (0.31 − 0.52) | 7.4E-13 | 1.5E-09 |
| SCAI | 0.39 (0.30 − 0.51) | 7.4E-13 | 1.5E-09 |
| SHC3 | 0.40 (0.31 − 0.52) | 7.9E-13 | 1.6E-09 |
| KIF18B | 2.38 (1.86 − 3.03) | 8.0E-13 | 1.6E-09 |
| CDC20 | 2.39 (1.87 − 3.05) | 8.6E-13 | 1.7E-09 |
| COL1A1 | 2.38 (1.86 − 3.04) | 8.7E-13 | 1.7E-09 |
| SPG20 | 0.40 (0.31 − 0.52) | 1.1E-12 | 2.2E-09 |
| SYNE1 | 0.42 (0.32 − 0.53) | 1.2E-12 | 2.4E-09 |
| CHRDL1 | 0.42 (0.33 − 0.54) | 1.3E-12 | 2.6E-09 |
| ARAP2 | 0.41 (0.32 − 0.53) | 1.4E-12 | 2.8E-09 |
| CGNL1 | 0.40 (0.31 − 0.52) | 1.7E-12 | 3.4E-09 |
| PTTG1 | 2.36 (1.85 − 3.02) | 1.9E-12 | 3.8E-09 |
| PCDH7 | 2.31 (1.82 − 2.94) | 2.0E-12 | 4.0E-09 |
| SYNPO2 | 0.40 (0.31 − 0.52) | 2.0E-12 | 4.0E-09 |
| HIST2H2AA4 | 2.37 (1.85 − 3.04) | 2.2E-12 | 4.4E-09 |
| SCN7A | 0.41 (0.32 − 0.53) | 2.3E-12 | 4.6E-09 |
| TNS1 | 0.41 (0.32 − 0.53) | 2.6E-12 | 5.2E-09 |
| ADRB2 | 0.42 (0.33 − 0.54) | 2.9E-12 | 5.7E-09 |
| CCNB2 | 2.36 (1.84 − 3.02) | 2.9E-12 | 5.7E-09 |
| RECK | 0.43 (0.34 − 0.55) | 3.0E-12 | 5.9E-09 |
| TBX5-AS1 | 0.41 (0.32 − 0.53) | 3.0E-12 | 5.9E-09 |
| MCM4 | 2.37 (1.85 − 3.05) | 3.2E-12 | 6.3E-09 |
| ARHGEF6 | 0.43 (0.33 − 0.55) | 3.7E-12 | 7.3E-09 |
| SSBP2 | 0.43 (0.34 − 0.55) | 3.9E-12 | 7.7E-09 |
| NEK2 | 2.33 (1.82 − 2.98) | 4.1E-12 | 8.1E-09 |
| TCF4 | 0.43 (0.33 − 0.55) | 5.2E-12 | 1.0E-08 |
| GUCY1A2 | 0.41 (0.32 − 0.54) | 5.8E-12 | 1.1E-08 |
| CLASP2 | 0.44 (0.34 − 0.56) | 6.1E-12 | 1.2E-08 |
| PTPN21 | 0.41 (0.32 − 0.54) | 6.7E-12 | 1.3E-08 |
| SYNC | 0.43 (0.34 − 0.55) | 6.7E-12 | 1.3E-08 |
| ZNF25 | 0.42 (0.32 − 0.54) | 6.9E-12 | 1.4E-08 |
| SASH1 | 0.41 (0.32 − 0.54) | 7.0E-12 | 1.4E-08 |
| JAM2 | 0.41 (0.31 − 0.53) | 7.1E-12 | 1.4E-08 |
| USP53 | 0.41 (0.32 − 0.54) | 7.4E-12 | 1.5E-08 |
| CPED1 | 0.42 (0.32 − 0.54) | 7.5E-12 | 1.5E-08 |
| RGS13 | 0.44 (0.34 − 0.56) | 7.5E-12 | 1.5E-08 |
| TBX5 | 0.42 (0.32 − 0.54) | 8.6E-12 | 1.7E-08 |
| AURKA | 2.02 (1.64 − 2.48) | 9.2E-12 | 1.8E-08 |
| PTPRM | 0.41 (0.32 − 0.54) | 1.0E-11 | 2.0E-08 |
| CNTN4 | 0.42 (0.32 − 0.54) | 1.2E-11 | 2.4E-08 |
| LOC100129550 | 0.41 (0.32 − 0.54) | 1.2E-11 | 2.4E-08 |
| SLC2A1 | 2.25 (1.77 − 2.86) | 1.2E-11 | 2.4E-08 |
| MAD2L1 | 2.41 (1.86 − 3.14) | 1.3E-11 | 2.6E-08 |
| MKI67 | 2.27 (1.78 − 2.90) | 1.3E-11 | 2.6E-08 |
| TXNL1 | 0.42 (0.33 − 0.55) | 1.3E-11 | 2.6E-08 |
| CFLAR | 0.43 (0.33 − 0.55) | 1.4E-11 | 2.8E-08 |
| HMGA1 | 2.29 (1.79 − 2.92) | 1.4E-11 | 2.8E-08 |
| LIFR | 0.43 (0.33 − 0.55) | 1.4E-11 | 2.8E-08 |
| STRBP | 0.41 (0.32 − 0.54) | 1.4E-11 | 2.8E-08 |
| FGF14-AS2 | 0.42 (0.32 − 0.54) | 1.5E-11 | 3.0E-08 |
| SPC25 | 2.26 (1.77 − 2.88) | 1.5E-11 | 3.0E-08 |
| EDNRB | 0.44 (0.35 − 0.57) | 1.7E-11 | 3.4E-08 |
| WIF1 | 0.44 (0.35 − 0.57) | 1.7E-11 | 3.4E-08 |
| CNKSR2 | 0.42 (0.33 − 0.55) | 1.8E-11 | 3.6E-08 |
| TMEM237 | 0.42 (0.33 − 0.55) | 1.8E-11 | 3.6E-08 |
| KIF14 | 2.27 (1.78 − 2.91) | 2.0E-11 | 4.0E-08 |
| DOCK4 | 0.44 (0.35 − 0.57) | 2.2E-11 | 4.4E-08 |
| PLSCR4 | 0.44 (0.34 − 0.56) | 2.3E-11 | 4.6E-08 |
| RNF125 | 0.43 (0.33 − 0.55) | 2.4E-11 | 4.8E-08 |
| ANKRD29 | 2.26 (1.77 − 2.89) | 2.5E-11 | 5.0E-08 |
| PDK1 | 0.44 (0.34 − 0.56) | 2.5E-11 | 5.0E-08 |
| RAP1A | 0.42 (0.33 − 0.55) | 2.7E-11 | 5.4E-08 |
| MIF | 2.30 (1.79 − 2.96) | 2.8E-11 | 5.5E-08 |
| PRX | 0.45 (0.35 − 0.57) | 2.8E-11 | 5.5E-08 |
| SLIT2 | 0.42 (0.32 − 0.55) | 2.8E-11 | 5.5E-08 |
| FILIP1 | 0.43 (0.33 − 0.55) | 2.9E-11 | 5.7E-08 |
| CRIM1 | 0.45 (0.35 − 0.57) | 3.1E-11 | 6.1E-08 |
| MSRB3 | 2.33 (1.80 − 3.01) | 3.1E-11 | 6.1E-08 |
| PID1 | 0.45 (0.35 − 0.57) | 3.1E-11 | 6.1E-08 |
| RNASEH2A | 2.24 (1.75 − 2.85) | 3.1E-11 | 6.1E-08 |
| SH3BP5 | 0.45 (0.35 − 0.57) | 3.1E-11 | 6.1E-08 |
| UBE2T | 2.35 (1.81 − 3.05) | 3.5E-11 | 6.9E-08 |
| GNAQ | 2.25 (1.76 − 2.88) | 3.6E-11 | 7.1E-08 |
| GNG2 | 0.43 (0.34 − 0.56) | 3.8E-11 | 7.5E-08 |
| PSAT1 | 2.23 (1.74 − 2.84) | 3.9E-11 | 7.7E-08 |
| PPAP2B | 0.45 (0.35 − 0.57) | 4.0E-11 | 7.9E-08 |
| EXO1 | 2.21 (1.74 − 2.81) | 4.1E-11 | 8.1E-08 |
| SETBP1 | 0.43 (0.33 − 0.56) | 4.2E-11 | 8.3E-08 |
| MICU3 | 0.43 (0.33 − 0.55) | 4.3E-11 | 8.5E-08 |
| P4HB | 2.27 (1.77 − 2.91) | 4.4E-11 | 8.7E-08 |
| ALDOA | 2.24 (1.75 − 2.86) | 4.5E-11 | 8.9E-08 |
| C1orf21 | 0.44 (0.34 − 0.56) | 4.7E-11 | 9.3E-08 |
| LSAMP | 0.42 (0.32 − 0.55) | 4.7E-11 | 9.3E-08 |
| GPI | 0.45 (0.35 − 0.57) | 4.8E-11 | 9.5E-08 |
| ACACB | 0.44 (0.34 − 0.57) | 5.0E-11 | 9.9E-08 |
| BOLA2B | 2.25 (1.76 − 2.89) | 5.1E-11 | 1.0E-07 |
| FAM13C | 0.44 (0.34 − 0.56) | 5.2E-11 | 1.0E-07 |
| GALNT2 | 0.45 (0.35 − 0.58) | 5.4E-11 | 1.1E-07 |
| GTSE1 | 2.21 (1.73 − 2.81) | 5.4E-11 | 1.1E-07 |
| SDPR | 0.44 (0.34 − 0.57) | 5.5E-11 | 1.1E-07 |
| RNF180 | 0.44 (0.34 − 0.56) | 5.6E-11 | 1.1E-07 |
| PDE5A | 0.44 (0.34 − 0.56) | 5.8E-11 | 1.1E-07 |
| S100A12 | 2.18 (1.72 − 2.77) | 5.8E-11 | 1.1E-07 |
| SAP18 | 0.46 (0.36 − 0.58) | 5.8E-11 | 1.1E-07 |
| PTGIS | 2.20 (1.72 − 2.80) | 6.2E-11 | 1.2E-07 |
| FCGR3B | 2.25 (1.75 − 2.89) | 6.4E-11 | 1.3E-07 |
| TMEM64 | 0.44 (0.34 − 0.57) | 9.1E-11 | 1.8E-07 |
| PRICKLE2 | 0.43 (0.34 − 0.56) | 9.4E-11 | 1.9E-07 |
| KIF20A | 2.18 (1.71 − 2.78) | 9.7E-11 | 1.9E-07 |
| CDCA5 | 2.26 (1.75 − 2.92) | 1.0E-10 | 2.0E-07 |
| FGFR2 | 0.46 (0.36 − 0.58) | 1.0E-10 | 2.0E-07 |
| ID4 | 0.46 (0.36 − 0.58) | 1.0E-10 | 2.0E-07 |
| GPM6B | 0.46 (0.36 − 0.58) | 1.2E-10 | 2.4E-07 |
| S100A8 | 2.24 (1.74 − 2.87) | 1.2E-10 | 2.4E-07 |
| BDH2 | 0.44 (0.34 − 0.57) | 1.3E-10 | 2.6E-07 |
| CHEK1 | 2.19 (1.71 − 2.80) | 1.3E-10 | 2.6E-07 |
| DOCK9 | 0.46 (0.37 − 0.59) | 1.3E-10 | 2.6E-07 |
| ECM2 | 0.46 (0.36 − 0.59) | 1.3E-10 | 2.6E-07 |
| TACC1 | 0.44 (0.34 − 0.57) | 1.3E-10 | 2.6E-07 |
| PLEKHH2 | 0.44 (0.34 − 0.57) | 1.4E-10 | 2.8E-07 |
| PROM2 | 2.26 (1.75 − 2.92) | 1.4E-10 | 2.8E-07 |
| PDZD2 | 0.46 (0.36 − 0.59) | 1.5E-10 | 3.0E-07 |
| PFDN6 | 2.24 (1.74 − 2.89) | 1.7E-10 | 3.4E-07 |
| ETS2 | 0.46 (0.36 − 0.59) | 1.9E-10 | 3.8E-07 |
| SCARA5 | 0.45 (0.35 − 0.58) | 2.0E-10 | 4.0E-07 |
| FAM49A | 0.44 (0.34 − 0.57) | 2.2E-10 | 4.4E-07 |
| GIMAP6 | 0.46 (0.36 − 0.59) | 2.2E-10 | 4.4E-07 |
| ARGLU1 | 0.45 (0.35 − 0.58) | 2.3E-10 | 4.6E-07 |
| PKP3 | 2.17 (1.70 − 2.77) | 2.3E-10 | 4.6E-07 |
| RPL39L | 2.15 (1.69 − 2.74) | 2.3E-10 | 4.6E-07 |
| PRDM5 | 0.45 (0.34 − 0.58) | 2.4E-10 | 4.8E-07 |
| FLI1 | 0.47 (0.37 − 0.59) | 2.6E-10 | 5.2E-07 |
| NEDD9 | 0.47 (0.36 − 0.59) | 2.6E-10 | 5.2E-07 |
| ADH1B | 0.47 (0.37 − 0.60) | 3.0E-10 | 5.9E-07 |
| LPL | 0.47 (0.37 − 0.60) | 3.0E-10 | 5.9E-07 |
| SLC2A5 | 2.13 (1.67 − 2.71) | 3.0E-10 | 5.9E-07 |
| KIAA0101 | 1.91 (1.56 − 2.35) | 3.1E-10 | 6.1E-07 |
| METTL7A | 0.47 (0.36 − 0.59) | 3.1E-10 | 6.1E-07 |
| RCC1 | 2.12 (1.67 − 2.70) | 3.3E-10 | 6.5E-07 |
| LHFP | 0.47 (0.36 − 0.59) | 3.4E-10 | 6.7E-07 |
| RGS5 | 2.28 (1.75 − 2.97) | 3.7E-10 | 7.3E-07 |
| TMEM178A | 0.45 (0.35 − 0.58) | 3.8E-10 | 7.5E-07 |
| MCC | 0.45 (0.35 − 0.58) | 4.0E-10 | 7.9E-07 |
| MAGED1 | 0.47 (0.37 − 0.60) | 4.1E-10 | 8.1E-07 |
| TGFBR3 | 0.46 (0.36 − 0.59) | 4.1E-10 | 8.1E-07 |
| ZEB1 | 0.46 (0.36 − 0.59) | 4.1E-10 | 8.1E-07 |
| SLC39A8 | 0.48 (0.37 − 0.60) | 4.3E-10 | 8.5E-07 |
| TIMP1 | 2.16 (1.69 − 2.76) | 4.3E-10 | 8.5E-07 |
| QKI | 0.47 (0.37 − 0.60) | 5.2E-10 | 1.0E-06 |
| ROR1 | 0.46 (0.35 − 0.59) | 5.2E-10 | 1.0E-06 |
| KLF13 | 0.45 (0.35 − 0.59) | 5.4E-10 | 1.1E-06 |
| SNX1 | 0.47 (0.37 − 0.60) | 5.4E-10 | 1.1E-06 |
| EHD2 | 2.11 (1.66 − 2.69) | 5.6E-10 | 1.1E-06 |
| KANK2 | 0.47 (0.36 − 0.60) | 5.6E-10 | 1.1E-06 |
| RBMS3 | 0.45 (0.35 − 0.59) | 5.9E-10 | 1.2E-06 |
| PPP1R16B | 0.47 (0.37 − 0.60) | 6.2E-10 | 1.2E-06 |
| PDK4 | 0.46 (0.36 − 0.59) | 7.0E-10 | 1.4E-06 |
| SLC12A8 | 2.09 (1.64 − 2.66) | 7.4E-10 | 1.5E-06 |
| C2orf40 | 0.46 (0.36 − 0.59) | 7.6E-10 | 1.5E-06 |
| NET1 | 0.48 (0.37 − 0.61) | 8.8E-10 | 1.7E-06 |
| TEK | 0.47 (0.37 − 0.60) | 8.9E-10 | 1.8E-06 |
| PTK2 | 0.48 (0.38 − 0.61) | 9.0E-10 | 1.8E-06 |
| SLC11A1 | 2.08 (1.64 − 2.65) | 9.0E-10 | 1.8E-06 |
| FHL1 | 0.47 (0.37 − 0.60) | 9.4E-10 | 1.9E-06 |
| SOBP | 0.47 (0.36 − 0.60) | 9.5E-10 | 1.9E-06 |
| AOC3 | 0.47 (0.37 − 0.61) | 9.6E-10 | 1.9E-06 |
| CD59 | 0.48 (0.37 − 0.61) | 9.6E-10 | 1.9E-06 |
| HGF | 0.48 (0.37 − 0.61) | 1.0E-09 | 2.0E-06 |
| DENND2A | 0.47 (0.37 − 0.60) | 1.1E-09 | 2.2E-06 |
| EIF5 | 0.47 (0.36 − 0.60) | 1.1E-09 | 2.2E-06 |
| ETS1 | 0.45 (0.35 − 0.59) | 1.1E-09 | 2.2E-06 |
| MAOB | 0.48 (0.38 − 0.61) | 1.1E-09 | 2.2E-06 |
| MATN2 | 0.48 (0.38 − 0.61) | 1.1E-09 | 2.2E-06 |
| RWDD1 | 0.48 (0.38 − 0.61) | 1.2E-09 | 2.4E-06 |
| SEC63 | 0.48 (0.37 − 0.61) | 1.2E-09 | 2.4E-06 |
| FOXP1 | 0.46 (0.36 − 0.60) | 1.3E-09 | 2.6E-06 |
| LOC100288911 | 0.46 (0.36 − 0.60) | 1.3E-09 | 2.6E-06 |
| PRRC1 | 0.47 (0.37 − 0.60) | 1.3E-09 | 2.6E-06 |
| PDE4D | 0.47 (0.37 − 0.60) | 1.4E-09 | 2.8E-06 |
| SEMA6D | 0.47 (0.36 − 0.60) | 1.5E-09 | 3.0E-06 |
| CENPA | 2.07 (1.63 − 2.64) | 1.6E-09 | 3.2E-06 |
| KRT8 | 2.08 (1.63 − 2.66) | 1.6E-09 | 3.2E-06 |
| MEIS2 | 0.48 (0.37 − 0.61) | 1.6E-09 | 3.2E-06 |
| TFAP2A | 2.06 (1.62 − 2.62) | 1.6E-09 | 3.2E-06 |
| RTN1 | 0.48 (0.37 − 0.61) | 1.7E-09 | 3.4E-06 |
| SPAG5 | 2.07 (1.63 − 2.64) | 1.7E-09 | 3.4E-06 |
| GIMAP7 | 0.47 (0.37 − 0.61) | 1.8E-09 | 3.6E-06 |
| FAM162B | 0.47 (0.36 − 0.60) | 1.9E-09 | 3.8E-06 |
| INMT | 0.47 (0.37 − 0.61) | 1.9E-09 | 3.8E-06 |
| ECE2 | 2.11 (1.65 − 2.72) | 2.1E-09 | 4.2E-06 |
| H2AFJ | 0.46 (0.36 − 0.60) | 2.1E-09 | 4.2E-06 |
| PDE3B | 0.49 (0.38 − 0.62) | 2.2E-09 | 4.4E-06 |
| PTPRD | 0.48 (0.38 − 0.62) | 2.3E-09 | 4.6E-06 |
| RHOB | 2.15 (1.66 − 2.77) | 2.3E-09 | 4.6E-06 |
| BMPER | 0.47 (0.36 − 0.61) | 2.4E-09 | 4.8E-06 |
| SLC35A2 | 2.06 (1.61 − 2.62) | 2.5E-09 | 5.0E-06 |
| NHS | 0.47 (0.37 − 0.61) | 2.9E-09 | 5.7E-06 |
| TMEM204 | 0.48 (0.37 − 0.61) | 2.9E-09 | 5.7E-06 |
| DOK6 | 0.47 (0.37 − 0.61) | 3.0E-09 | 5.9E-06 |
| C1orf112 | 2.08 (1.62 − 2.66) | 3.1E-09 | 6.1E-06 |
| KIF4A | 2.04 (1.60 − 2.59) | 3.2E-09 | 6.3E-06 |
| NFIB | 0.49 (0.39 − 0.62) | 3.2E-09 | 6.3E-06 |
| DSG2 | 2.17 (1.67 − 2.82) | 3.3E-09 | 6.5E-06 |
| KLF11 | 2.11 (1.64 − 2.72) | 3.4E-09 | 6.7E-06 |
| RPA3 | 2.09 (1.63 − 2.68) | 3.4E-09 | 6.7E-06 |
| SLC24A3 | 0.49 (0.38 − 0.62) | 3.5E-09 | 6.9E-06 |
| TPD52 | 0.48 (0.37 − 0.61) | 3.8E-09 | 7.5E-06 |
| ARHGEF10 | 0.49 (0.39 − 0.63) | 3.9E-09 | 7.7E-06 |
| C1QTNF7 | 0.47 (0.37 − 0.61) | 4.1E-09 | 8.1E-06 |
| GIMAP1 | 0.47 (0.36 − 0.61) | 4.2E-09 | 8.3E-06 |
| FRAS1 | 0.48 (0.37 − 0.61) | 4.4E-09 | 8.7E-06 |
| MCM2 | 2.05 (1.60 − 2.62) | 4.8E-09 | 9.5E-06 |
| CCNB1 | 2.04 (1.60 − 2.61) | 5.4E-09 | 1.1E-05 |
| ADAMTSL3 | 0.49 (0.38 − 0.62) | 5.5E-09 | 1.1E-05 |
| LMNB1 | 2.02 (1.58 − 2.56) | 5.7E-09 | 1.1E-05 |
| RFX3 | 0.48 (0.37 − 0.61) | 5.7E-09 | 1.1E-05 |
| PRKG1 | 0.48 (0.37 − 0.62) | 6.1E-09 | 1.2E-05 |
| FERMT2 | 0.50 (0.39 − 0.63) | 6.4E-09 | 1.3E-05 |
| TPPP | 0.48 (0.37 − 0.62) | 6.9E-09 | 1.4E-05 |
| EFEMP1 | 0.50 (0.40 − 0.64) | 7.5E-09 | 1.5E-05 |
| LAMA4 | 1.99 (1.57 − 2.53) | 7.5E-09 | 1.5E-05 |
| PBK | 2.02 (1.58 − 2.57) | 8.2E-09 | 1.6E-05 |
| SATB1 | 0.48 (0.37 − 0.62) | 8.6E-09 | 1.7E-05 |
| CCNE1 | 1.99 (1.57 − 2.53) | 8.8E-09 | 1.7E-05 |
| NCAPH | 2.00 (1.57 − 2.55) | 8.9E-09 | 1.8E-05 |
| UHRF1 | 2.05 (1.60 − 2.64) | 8.9E-09 | 1.8E-05 |
| TNFRSF21 | 2.03 (1.59 − 2.60) | 9.0E-09 | 1.8E-05 |
| LATS2 | 0.48 (0.37 − 0.62) | 9.5E-09 | 1.9E-05 |
| AFF3 | 0.49 (0.38 − 0.63) | 1.0E-08 | 2.0E-05 |
| MYADM | 2.04 (1.59 − 2.62) | 1.0E-08 | 2.0E-05 |
| CBX7 | 0.50 (0.39 − 0.64) | 1.1E-08 | 2.2E-05 |
| PGM5 | 0.48 (0.38 − 0.62) | 1.1E-08 | 2.2E-05 |
| PGR | 0.48 (0.37 − 0.62) | 1.1E-08 | 2.2E-05 |
| PTPRG | 0.50 (0.40 − 0.64) | 1.1E-08 | 2.2E-05 |
| C14orf132 | 0.50 (0.39 − 0.64) | 1.2E-08 | 2.4E-05 |
| FRMD4A | 0.48 (0.37 − 0.62) | 1.2E-08 | 2.4E-05 |
| MAL | 1.96 (1.55 − 2.48) | 1.3E-08 | 2.6E-05 |
| LINC00968 | 0.49 (0.38 − 0.63) | 1.4E-08 | 2.8E-05 |
| UTRN | 0.49 (0.38 − 0.63) | 1.4E-08 | 2.8E-05 |
| BRIP1 | 1.98 (1.56 − 2.53) | 1.5E-08 | 3.0E-05 |
| CAMK2N1 | 2.02 (1.58 − 2.60) | 1.5E-08 | 3.0E-05 |
| PDS5B | 0.50 (0.39 − 0.64) | 1.5E-08 | 3.0E-05 |
| LRCH2 | 0.49 (0.38 − 0.63) | 1.6E-08 | 3.2E-05 |
| TRHDE | 0.50 (0.40 − 0.64) | 1.6E-08 | 3.2E-05 |
| IQGAP3 | 2.01 (1.57 − 2.57) | 1.7E-08 | 3.4E-05 |
| PAIP1 | 0.51 (0.40 − 0.65) | 1.7E-08 | 3.4E-05 |
| PC | 0.51 (0.40 − 0.64) | 1.7E-08 | 3.4E-05 |
| PODXL | 0.51 (0.40 − 0.64) | 1.7E-08 | 3.4E-05 |
| SSH2 | 2.01 (1.57 − 2.57) | 1.8E-08 | 3.6E-05 |
| LEPR | 0.51 (0.40 − 0.65) | 1.9E-08 | 3.8E-05 |
| RAPGEF5 | 0.51 (0.40 − 0.65) | 1.9E-08 | 3.8E-05 |
| SMAD9 | 0.49 (0.38 − 0.63) | 1.9E-08 | 3.8E-05 |
| ENG | 1.97 (1.55 − 2.51) | 2.0E-08 | 4.0E-05 |
| FYN | 0.51 (0.40 − 0.65) | 2.0E-08 | 4.0E-05 |
| MND1 | 2.04 (1.58 − 2.63) | 2.0E-08 | 4.0E-05 |
| SLK | 0.51 (0.40 − 0.65) | 2.0E-08 | 4.0E-05 |
| STX3 | 1.99 (1.56 − 2.53) | 2.0E-08 | 4.0E-05 |
| HEG1 | 0.50 (0.40 − 0.64) | 2.1E-08 | 4.2E-05 |
| MPDZ | 0.51 (0.40 − 0.65) | 2.2E-08 | 4.4E-05 |
| PAPSS2 | 0.51 (0.40 − 0.65) | 2.3E-08 | 4.6E-05 |
| PRKCH | 0.50 (0.39 − 0.64) | 2.3E-08 | 4.6E-05 |
| FMO2 | 0.50 (0.39 − 0.64) | 2.4E-08 | 4.8E-05 |
| NTN4 | 0.50 (0.39 − 0.64) | 2.4E-08 | 4.8E-05 |
| ULK2 | 0.51 (0.40 − 0.65) | 2.4E-08 | 4.8E-05 |
| CYBRD1 | 0.50 (0.39 − 0.64) | 2.5E-08 | 5.0E-05 |
| PELO | 0.51 (0.41 − 0.65) | 2.6E-08 | 5.2E-05 |
| SRPK1 | 1.96 (1.54 − 2.50) | 2.6E-08 | 5.2E-05 |
| CDS2 | 0.51 (0.40 − 0.65) | 2.8E-08 | 5.5E-05 |
| H2BFS | 1.99 (1.55 − 2.54) | 2.8E-08 | 5.5E-05 |
| FOXO1 | 0.50 (0.39 − 0.64) | 2.9E-08 | 5.7E-05 |
| LDB2 | 0.51 (0.40 − 0.65) | 3.0E-08 | 5.9E-05 |
| MDK | 1.96 (1.54 − 2.50) | 3.0E-08 | 5.9E-05 |
| MGAT4A | 0.50 (0.39 − 0.64) | 3.0E-08 | 5.9E-05 |
| RAB8B | 0.50 (0.39 − 0.64) | 3.0E-08 | 5.9E-05 |
| UGGT1 | 0.49 (0.37 − 0.63) | 3.1E-08 | 6.1E-05 |
| RAI2 | 0.50 (0.39 − 0.64) | 3.2E-08 | 6.3E-05 |
| RRM2 | 1.95 (1.53 − 2.49) | 3.2E-08 | 6.3E-05 |
| HMMR | 1.95 (1.53 − 2.48) | 3.3E-08 | 6.5E-05 |
| TCF21 | 0.51 (0.40 − 0.65) | 3.3E-08 | 6.5E-05 |
| PDZRN3 | 0.51 (0.40 − 0.65) | 3.4E-08 | 6.7E-05 |
| CENPU | 1.98 (1.55 − 2.54) | 3.6E-08 | 7.1E-05 |
| PCBD2 | 0.50 (0.39 − 0.64) | 3.6E-08 | 7.1E-05 |
| FAM199X | 0.50 (0.39 − 0.64) | 3.8E-08 | 7.5E-05 |
| LRRN3 | 0.51 (0.40 − 0.65) | 3.9E-08 | 7.7E-05 |
| SHROOM4 | 0.50 (0.39 − 0.64) | 3.9E-08 | 7.7E-05 |
| ENO1 | 1.98 (1.55 − 2.54) | 4.0E-08 | 7.9E-05 |
| NAP1L5 | 0.50 (0.39 − 0.64) | 4.1E-08 | 8.1E-05 |
| WWTR1 | 1.93 (1.52 − 2.46) | 4.1E-08 | 8.1E-05 |
| PGAP1 | 0.49 (0.38 − 0.64) | 4.3E-08 | 8.5E-05 |
| HN1L | 1.98 (1.54 − 2.54) | 4.4E-08 | 8.7E-05 |
| TNPO1 | 0.51 (0.40 − 0.66) | 4.6E-08 | 9.1E-05 |
| MBNL1-AS1 | 0.50 (0.39 − 0.65) | 4.7E-08 | 9.3E-05 |
| SESN1 | 0.51 (0.40 − 0.65) | 4.7E-08 | 9.3E-05 |
| STXBP6 | 1.91 (1.51 − 2.42) | 4.8E-08 | 9.5E-05 |
| H2AFV | 0.52 (0.41 − 0.66) | 4.9E-08 | 9.7E-05 |
| SHMT2 | 1.92 (1.51 − 2.44) | 4.9E-08 | 9.7E-05 |
| DNAJC27 | 0.50 (0.38 − 0.64) | 5.1E-08 | 1.0E-04 |
| FIGF | 0.52 (0.41 − 0.66) | 5.1E-08 | 1.0E-04 |
| SAMD5 | 0.50 (0.39 − 0.65) | 5.1E-08 | 1.0E-04 |
| SEC24A | 0.52 (0.41 − 0.66) | 5.4E-08 | 1.1E-04 |
| LRRFIP1 | 0.51 (0.40 − 0.65) | 5.5E-08 | 1.1E-04 |
| STX1A | 1.90 (1.50 − 2.41) | 5.6E-08 | 1.1E-04 |
| MAP3K8 | 0.52 (0.41 − 0.66) | 5.7E-08 | 1.1E-04 |
| RHBDL2 | 2.00 (1.55 − 2.58) | 5.7E-08 | 1.1E-04 |
| F12 | 1.88 (1.49 − 2.37) | 5.8E-08 | 1.1E-04 |
| KCNAB1 | 0.52 (0.41 − 0.66) | 7.0E-08 | 1.4E-04 |
| GINS2 | 1.92 (1.51 − 2.44) | 7.3E-08 | 1.4E-04 |
| HSPB8 | 0.52 (0.41 − 0.67) | 7.3E-08 | 1.4E-04 |
| PECAM1 | 0.52 (0.41 − 0.66) | 7.8E-08 | 1.5E-04 |
| MYLK | 0.51 (0.39 − 0.65) | 7.9E-08 | 1.6E-04 |
| P2RY14 | 0.52 (0.41 − 0.67) | 7.9E-08 | 1.6E-04 |
| MEX3A | 1.96 (1.52 − 2.51) | 8.1E-08 | 1.6E-04 |
| PTRF | 1.97 (1.53 − 2.53) | 8.3E-08 | 1.6E-04 |
| SNRK | 0.52 (0.41 − 0.67) | 8.4E-08 | 1.7E-04 |
| PIK3C3 | 0.53 (0.42 − 0.67) | 9.4E-08 | 1.9E-04 |
| RSPO2 | 0.52 (0.41 − 0.67) | 9.5E-08 | 1.9E-04 |
| RUNX1T1 | 0.52 (0.41 − 0.67) | 9.5E-08 | 1.9E-04 |
| NLRP3 | 0.52 (0.41 − 0.67) | 1.2E-07 | 2.4E-04 |
| MAGI2-AS3 | 0.51 (0.40 − 0.66) | 1.0E-07 | 2.0E-04 |
| NEBL | 1.88 (1.48 − 2.38) | 1.0E-07 | 2.0E-04 |
| AGTPBP1 | 0.53 (0.42 − 0.67) | 1.1E-07 | 2.2E-04 |
| DEPDC1 | 1.97 (1.53 − 2.54) | 1.1E-07 | 2.2E-04 |
| EXT1 | 0.52 (0.41 − 0.66) | 1.1E-07 | 2.2E-04 |
| GRK5 | 0.52 (0.41 − 0.67) | 1.2E-07 | 2.4E-04 |
| ACVRL1 | 1.88 (1.48 − 2.38) | 1.3E-07 | 2.6E-04 |
| C4orf32 | 0.51 (0.40 − 0.66) | 1.3E-07 | 2.6E-04 |
| DLC1 | 1.93 (1.51 − 2.47) | 1.3E-07 | 2.6E-04 |
| TMEM106B | 0.52 (0.41 − 0.67) | 1.3E-07 | 2.6E-04 |
| LILRA2 | 1.88 (1.48 − 2.39) | 1.4E-07 | 2.8E-04 |
| MAGI1 | 0.50 (0.39 − 0.65) | 1.4E-07 | 2.8E-04 |
| PARVA | 0.53 (0.42 − 0.68) | 1.4E-07 | 2.8E-04 |
| PMP22 | 0.53 (0.42 − 0.67) | 1.4E-07 | 2.8E-04 |
| PTPRB | 0.52 (0.40 − 0.66) | 1.4E-07 | 2.8E-04 |
| TSPAN7 | 0.53 (0.41 − 0.67) | 1.4E-07 | 2.8E-04 |
| GPM6A | 0.53 (0.41 − 0.67) | 1.5E-07 | 3.0E-04 |
| HS6ST2 | 0.52 (0.41 − 0.67) | 1.5E-07 | 3.0E-04 |
| MCTS1 | 0.51 (0.40 − 0.66) | 1.5E-07 | 3.0E-04 |
| SH3GL3 | 1.87 (1.47 − 2.36) | 1.5E-07 | 3.0E-04 |
| VAPA | 0.52 (0.41 − 0.67) | 1.5E-07 | 3.0E-04 |
| S100A10 | 1.89 (1.48 − 2.40) | 1.6E-07 | 3.2E-04 |
| SOX17 | 0.51 (0.39 − 0.66) | 1.6E-07 | 3.2E-04 |
| NACC2 | 1.92 (1.50 − 2.47) | 1.7E-07 | 3.4E-04 |
| SCN4B | 0.52 (0.40 − 0.66) | 1.7E-07 | 3.4E-04 |
| TIMELESS | 1.87 (1.47 − 2.37) | 1.7E-07 | 3.4E-04 |
| TMEM99 | 0.52 (0.40 − 0.67) | 1.7E-07 | 3.4E-04 |
| UACA | 0.51 (0.40 − 0.66) | 1.7E-07 | 3.4E-04 |
| ZAK | 0.52 (0.41 − 0.67) | 1.7E-07 | 3.4E-04 |
| MSI2 | 1.96 (1.52 − 2.54) | 1.8E-07 | 3.6E-04 |
| RBM17 | 0.51 (0.40 − 0.66) | 1.8E-07 | 3.6E-04 |
| VGLL3 | 0.53 (0.42 − 0.68) | 1.8E-07 | 3.6E-04 |
| ARHGAP29 | 0.54 (0.42 − 0.68) | 1.9E-07 | 3.8E-04 |
| CD93 | 0.53 (0.42 − 0.68) | 1.9E-07 | 3.8E-04 |
| CDH13 | 0.53 (0.42 − 0.68) | 1.9E-07 | 3.8E-04 |
| STARD13 | 0.52 (0.41 − 0.67) | 2.0E-07 | 4.0E-04 |
| NEDD4L | 0.54 (0.43 − 0.68) | 2.2E-07 | 4.4E-04 |
| PPAPDC1B | 0.53 (0.41 − 0.68) | 2.5E-07 | 5.0E-04 |
| SYNM | 0.53 (0.42 − 0.68) | 2.6E-07 | 5.2E-04 |
| ADRB1 | 0.53 (0.41 − 0.67) | 2.7E-07 | 5.4E-04 |
| CCDC68 | 0.55 (0.43 − 0.69) | 2.8E-07 | 5.5E-04 |
| LRCH1 | 0.52 (0.40 − 0.67) | 2.8E-07 | 5.5E-04 |
| AQP9 | 1.85 (1.46 − 2.35) | 2.9E-07 | 5.7E-04 |
| LAPTM4B | 0.54 (0.43 − 0.69) | 2.9E-07 | 5.7E-04 |
| IDH2 | 1.86 (1.46 − 2.38) | 3.0E-07 | 5.9E-04 |
| SH3D19 | 0.53 (0.41 − 0.68) | 3.0E-07 | 5.9E-04 |
| MEF2A | 0.54 (0.43 − 0.69) | 3.1E-07 | 6.1E-04 |
| FGF11 | 1.88 (1.47 − 2.40) | 3.2E-07 | 6.3E-04 |
| GATA6 | 0.54 (0.43 − 0.69) | 3.2E-07 | 6.3E-04 |
| MEIS1 | 0.54 (0.42 − 0.68) | 3.3E-07 | 6.5E-04 |
| SAMHD1 | 1.89 (1.48 − 2.43) | 3.4E-07 | 6.7E-04 |
| TMEM47 | 0.54 (0.42 − 0.69) | 3.4E-07 | 6.7E-04 |
| TNXB | 1.86 (1.46 − 2.36) | 3.4E-07 | 6.7E-04 |
| WASF3 | 0.54 (0.42 − 0.69) | 3.5E-07 | 6.9E-04 |
| WWC2 | 0.52 (0.40 − 0.67) | 3.6E-07 | 7.1E-04 |
| MASP1 | 1.82 (1.44 − 2.31) | 3.8E-07 | 7.5E-04 |
| FAR2 | 0.53 (0.41 − 0.68) | 3.9E-07 | 7.7E-04 |
| IL4I1 | 1.83 (1.44 − 2.31) | 3.9E-07 | 7.7E-04 |
| RAB11A | 0.53 (0.41 − 0.68) | 4.0E-07 | 7.9E-04 |
| PEG3 | 0.54 (0.42 − 0.69) | 4.1E-07 | 8.1E-04 |
| CILP2 | 1.88 (1.47 − 2.42) | 4.2E-07 | 8.3E-04 |
| ACSS3 | 0.53 (0.41 − 0.68) | 4.4E-07 | 8.7E-04 |
| RNF182 | 0.53 (0.41 − 0.68) | 4.4E-07 | 8.7E-04 |
| PIP5K1B | 0.55 (0.43 − 0.69) | 4.5E-07 | 8.9E-04 |
| PRICKLE1 | 0.53 (0.41 − 0.68) | 4.5E-07 | 8.9E-04 |
| GABARAPL3 | 0.54 (0.43 − 0.69) | 5.0E-07 | 9.9E-04 |
| EP300-AS1 | 0.53 (0.41 − 0.68) | 5.1E-07 | 1.0E-03 |
| MTURN | 0.52 (0.40 − 0.67) | 5.2E-07 | 1.0E-03 |
| GLDN | 0.53 (0.41 − 0.68) | 5.5E-07 | 1.1E-03 |
| HOOK1 | 0.54 (0.42 − 0.69) | 5.6E-07 | 1.1E-03 |
| PLEKHA8 | 0.53 (0.41 − 0.68) | 5.6E-07 | 1.1E-03 |
| IL11RA | 0.54 (0.42 − 0.69) | 5.7E-07 | 1.1E-03 |
| KANK3 | 0.54 (0.43 − 0.69) | 5.8E-07 | 1.1E-03 |
| CREM | 0.55 (0.43 − 0.70) | 5.9E-07 | 1.2E-03 |
| VEPH1 | 0.53 (0.41 − 0.68) | 6.0E-07 | 1.2E-03 |
| LMO7 | 0.53 (0.41 − 0.68) | 6.1E-07 | 1.2E-03 |
| HECW2 | 0.53 (0.41 − 0.69) | 6.3E-07 | 1.2E-03 |
| PLCE1 | 0.55 (0.43 − 0.70) | 6.4E-07 | 1.3E-03 |
| ZEB2 | 0.55 (0.43 − 0.70) | 6.4E-07 | 1.3E-03 |
| SPARCL1 | 0.55 (0.44 − 0.70) | 6.5E-07 | 1.3E-03 |
| LAD1 | 1.82 (1.43 − 2.31) | 6.6E-07 | 1.3E-03 |
| FGD5 | 0.53 (0.41 − 0.68) | 6.7E-07 | 1.3E-03 |
| GCOM1 | 0.55 (0.43 − 0.70) | 6.7E-07 | 1.3E-03 |
| PCDH9 | 0.55 (0.43 − 0.70) | 6.7E-07 | 1.3E-03 |
| PEAK1 | 0.53 (0.41 − 0.68) | 6.7E-07 | 1.3E-03 |
| TMEM100 | 0.55 (0.43 − 0.70) | 6.7E-07 | 1.3E-03 |
| EPAS1 | 1.82 (1.43 − 2.32) | 7.2E-07 | 1.4E-03 |
| FRMD4B | 0.55 (0.43 − 0.70) | 7.5E-07 | 1.5E-03 |
| BTNL9 | 0.54 (0.42 − 0.69) | 7.6E-07 | 1.5E-03 |
| PGM2L1 | 1.85 (1.44 − 2.37) | 7.6E-07 | 1.5E-03 |
| ITGA8 | 0.54 (0.43 − 0.70) | 7.9E-07 | 1.6E-03 |
| MTA3 | 1.87 (1.45 − 2.41) | 7.9E-07 | 1.6E-03 |
| PDZD11 | 1.85 (1.45 − 2.38) | 7.9E-07 | 1.6E-03 |
| FUT3 | 1.81 (1.42 − 2.29) | 8.3E-07 | 1.6E-03 |
| TSTA3 | 1.81 (1.43 − 2.30) | 8.4E-07 | 1.7E-03 |
| RNF144B | 0.54 (0.42 − 0.69) | 9.3E-07 | 1.8E-03 |
| MMRN1 | 0.55 (0.44 − 0.70) | 9.4E-07 | 1.9E-03 |
| NCAPG | 1.81 (1.42 − 2.30) | 1.0E-06 | 2.0E-03 |
| FAM150B | 0.54 (0.42 − 0.70) | 1.1E-06 | 2.2E-03 |
| ID2 | 0.56 (0.44 − 0.71) | 1.1E-06 | 2.2E-03 |
| LMCD1 | 0.55 (0.43 − 0.70) | 1.1E-06 | 2.2E-03 |
| LMOD1 | 0.55 (0.44 − 0.71) | 1.1E-06 | 2.2E-03 |
| OLFML1 | 0.55 (0.43 − 0.70) | 1.1E-06 | 2.2E-03 |
| TTK | 1.80 (1.42 − 2.30) | 1.1E-06 | 2.2E-03 |
| VLDLR | 0.56 (0.44 − 0.71) | 1.1E-06 | 2.2E-03 |
| CACNA1D | 0.56 (0.44 − 0.71) | 1.2E-06 | 2.4E-03 |
| GIMAP5 | 0.55 (0.43 − 0.70) | 1.2E-06 | 2.4E-03 |
| NCKAP5 | 0.54 (0.42 − 0.70) | 1.2E-06 | 2.4E-03 |
| C5AR1 | 1.78 (1.40 − 2.25) | 1.3E-06 | 2.6E-03 |
| GHR | 0.55 (0.43 − 0.71) | 1.3E-06 | 2.6E-03 |
| NES | 0.53 (0.41 − 0.69) | 1.3E-06 | 2.6E-03 |
| SLC2A3 | 1.78 (1.40 − 2.26) | 1.3E-06 | 2.6E-03 |
| UBFD1 | 0.54 (0.42 − 0.70) | 1.4E-06 | 2.8E-03 |
| SULF1 | 1.80 (1.41 − 2.29) | 1.5E-06 | 3.0E-03 |
| DEFA1B | 1.76 (1.39 − 2.23) | 1.6E-06 | 3.2E-03 |
| FANCI | 1.83 (1.42 − 2.34) | 1.6E-06 | 3.2E-03 |
| TOX2 | 0.55 (0.42 − 0.70) | 1.6E-06 | 3.2E-03 |
| CDO1 | 0.56 (0.44 − 0.71) | 1.7E-06 | 3.4E-03 |
| EMP1 | 0.56 (0.45 − 0.72) | 1.7E-06 | 3.4E-03 |
| IFT57 | 0.55 (0.43 − 0.70) | 1.7E-06 | 3.4E-03 |
| PPP1R14B | 1.79 (1.40 − 2.27) | 1.7E-06 | 3.4E-03 |
| GATA2 | 1.76 (1.39 − 2.23) | 1.8E-06 | 3.6E-03 |
| PNPLA6 | 1.76 (1.39 − 2.22) | 1.8E-06 | 3.6E-03 |
| SIK2 | 0.56 (0.44 − 0.71) | 1.8E-06 | 3.6E-03 |
| CNRIP1 | 0.55 (0.43 − 0.70) | 1.9E-06 | 3.8E-03 |
| KL | 0.56 (0.44 − 0.72) | 1.9E-06 | 3.8E-03 |
| PHACTR2 | 0.55 (0.43 − 0.70) | 1.9E-06 | 3.8E-03 |
| PPM1F | 0.56 (0.44 − 0.71) | 1.9E-06 | 3.8E-03 |
| TRPC6 | 0.57 (0.45 − 0.72) | 1.9E-06 | 3.8E-03 |
| ACE | 1.76 (1.39 − 2.22) | 2.0E-06 | 4.0E-03 |
| RPGR | 0.56 (0.44 − 0.72) | 2.0E-06 | 4.0E-03 |
| WISP1 | 1.76 (1.39 − 2.24) | 2.0E-06 | 4.0E-03 |
| ZC3H12C | 0.55 (0.43 − 0.71) | 2.1E-06 | 4.2E-03 |
| JDP2 | 1.80 (1.41 − 2.31) | 2.2E-06 | 4.4E-03 |
| KLF9 | 0.57 (0.45 − 0.72) | 2.2E-06 | 4.4E-03 |
| RAPGEF4 | 0.56 (0.44 − 0.72) | 2.2E-06 | 4.4E-03 |
| RFC4 | 1.78 (1.40 − 2.26) | 2.2E-06 | 4.4E-03 |
| SECISBP2L | 0.56 (0.44 − 0.72) | 2.3E-06 | 4.6E-03 |
| SMAD6 | 0.56 (0.44 − 0.72) | 2.3E-06 | 4.6E-03 |
| PPAPDC1A | 1.79 (1.40 − 2.29) | 2.4E-06 | 4.8E-03 |
| CDCA8 | 1.76 (1.39 − 2.24) | 2.5E-06 | 5.0E-03 |
| FOXF2 | 0.56 (0.44 − 0.72) | 2.6E-06 | 5.2E-03 |
| PPP1R15A | 1.75 (1.38 − 2.21) | 2.6E-06 | 5.2E-03 |
| PALMD | 0.57 (0.45 − 0.72) | 2.7E-06 | 5.4E-03 |
| SCARA3 | 0.56 (0.43 − 0.71) | 2.7E-06 | 5.4E-03 |
| SLC44A1 | 0.55 (0.43 − 0.71) | 2.7E-06 | 5.4E-03 |
| SEPT8 | 0.56 (0.44 − 0.72) | 2.8E-06 | 5.5E-03 |
| DAPK2 | 0.57 (0.45 − 0.72) | 2.8E-06 | 5.5E-03 |
| GNL1 | 0.55 (0.43 − 0.71) | 2.8E-06 | 5.5E-03 |
| RCCD1 | 1.78 (1.39 − 2.28) | 2.8E-06 | 5.5E-03 |
| IGSF9 | 1.79 (1.40 − 2.30) | 2.9E-06 | 5.7E-03 |
| SLC1A1 | 0.57 (0.45 − 0.72) | 2.9E-06 | 5.7E-03 |
| LINC00622 | 0.55 (0.43 − 0.71) | 3.0E-06 | 5.9E-03 |
| TOP2A | 1.76 (1.38 − 2.23) | 3.0E-06 | 5.9E-03 |
| PRKAR2B | 0.58 (0.46 − 0.73) | 3.2E-06 | 6.3E-03 |
| MMP11 | 1.74 (1.37 − 2.20) | 3.3E-06 | 6.5E-03 |
| MMRN2 | 0.55 (0.43 − 0.71) | 3.3E-06 | 6.5E-03 |
| MFI2 | 1.73 (1.37 − 2.18) | 3.5E-06 | 6.9E-03 |
| OTUD1 | 0.56 (0.44 − 0.72) | 3.6E-06 | 7.1E-03 |
| NPNT | 0.56 (0.44 − 0.72) | 3.7E-06 | 7.3E-03 |
| RHOJ | 0.56 (0.43 − 0.72) | 3.7E-06 | 7.3E-03 |
| ANLN | 1.78 (1.39 − 2.29) | 3.8E-06 | 7.5E-03 |
| MFNG | 1.74 (1.37 − 2.21) | 3.9E-06 | 7.7E-03 |
| ABCA6 | 0.57 (0.45 − 0.73) | 4.0E-06 | 7.9E-03 |
| SMAD7 | 0.57 (0.45 − 0.73) | 4.0E-06 | 7.9E-03 |
| SRPRB | 1.78 (1.39 − 2.28) | 4.0E-06 | 7.9E-03 |
| HIST1H2BE | 1.75 (1.38 − 2.24) | 4.1E-06 | 8.1E-03 |
| NFASC | 0.56 (0.44 − 0.72) | 4.1E-06 | 8.1E-03 |
| RMI2 | 1.77 (1.38 − 2.27) | 4.1E-06 | 8.1E-03 |
| PAICS | 1.73 (1.36 − 2.19) | 4.3E-06 | 8.5E-03 |
| TMOD1 | 0.58 (0.46 − 0.73) | 4.4E-06 | 8.7E-03 |
| SLC1A4 | 1.73 (1.36 − 2.19) | 4.5E-06 | 8.9E-03 |
| DSEL | 0.57 (0.44 − 0.72) | 4.6E-06 | 9.1E-03 |
| FEZ1 | 1.73 (1.37 − 2.20) | 4.6E-06 | 9.1E-03 |
| JAM3 | 0.57 (0.45 − 0.73) | 4.7E-06 | 9.3E-03 |
| TMEM167A | 0.57 (0.45 − 0.73) | 4.9E-06 | 9.7E-03 |
| TBX3 | 0.56 (0.44 − 0.72) | 5.1E-06 | 1.0E-02 |
| ADAM12 | 1.76 (1.37 − 2.25) | 5.2E-06 | 1.0E-02 |
| C8orf88 | 1.76 (1.38 − 2.26) | 5.2E-06 | 1.0E-02 |
| IGSF10 | 0.56 (0.44 − 0.72) | 5.3E-06 | 1.1E-02 |
| PLCL1 | 0.58 (0.45 − 0.73) | 5.4E-06 | 1.1E-02 |
| S1PR1 | 0.57 (0.45 − 0.73) | 5.9E-06 | 1.2E-02 |
| MSTO2P | 1.71 (1.35 − 2.17) | 6.3E-06 | 1.2E-02 |
| PLAGL1 | 0.58 (0.46 − 0.74) | 6.3E-06 | 1.2E-02 |
| SDF2L1 | 1.72 (1.36 − 2.19) | 6.6E-06 | 1.3E-02 |
| STK32A | 0.57 (0.44 − 0.73) | 6.7E-06 | 1.3E-02 |
| C4orf48 | 1.75 (1.37 − 2.24) | 6.8E-06 | 1.3E-02 |
| LDB3 | 1.70 (1.34 − 2.14) | 6.8E-06 | 1.3E-02 |
| GYPC | 1.71 (1.35 − 2.17) | 6.9E-06 | 1.4E-02 |
| IL33 | 0.59 (0.46 − 0.74) | 6.9E-06 | 1.4E-02 |
| UQCC2 | 1.76 (1.37 − 2.26) | 7.2E-06 | 1.4E-02 |
| FAM83A | 1.76 (1.37 − 2.26) | 7.7E-06 | 1.5E-02 |
| SOX7 | 0.57 (0.44 − 0.73) | 7.9E-06 | 1.6E-02 |
| TMOD2 | 0.57 (0.44 − 0.73) | 8.4E-06 | 1.7E-02 |
| TOX3 | 0.59 (0.47 − 0.75) | 8.7E-06 | 1.7E-02 |
| FKBP11 | 1.72 (1.35 − 2.19) | 9.2E-06 | 1.8E-02 |
| CYYR1 | 0.57 (0.45 − 0.74) | 9.3E-06 | 1.8E-02 |
| SEMA3B | 1.69 (1.34 − 2.14) | 9.3E-06 | 1.8E-02 |
| ITM2A | 0.59 (0.47 − 0.75) | 9.4E-06 | 1.9E-02 |
| NEXN | 0.58 (0.45 − 0.74) | 9.4E-06 | 1.9E-02 |
| TIMP3 | 1.71 (1.34 − 2.17) | 9.6E-06 | 1.9E-02 |
| KIAA0040 | 0.63 (0.52 − 0.78) | 9.7E-06 | 1.9E-02 |
| PPARGC1A | 0.59 (0.46 − 0.75) | 9.7E-06 | 1.9E-02 |
| NLRC4 | 1.64 (1.28 − 2.10) | 6.3E-05 | 1.2E-01 |
| GALNT18 | 0.57 (0.45 − 0.74) | 1.0E-05 | 2.0E-02 |
| GJB2 | 1.73 (1.35 − 2.21) | 1.0E-05 | 2.0E-02 |
| IL1RL1 | 1.69 (1.33 − 2.13) | 1.0E-05 | 2.0E-02 |
| PMEPA1 | 0.57 (0.45 − 0.74) | 1.1E-05 | 2.2E-02 |
| SGPL1 | 0.59 (0.46 − 0.75) | 1.1E-05 | 2.2E-02 |
| UBASH3B | 0.58 (0.45 − 0.74) | 1.1E-05 | 2.2E-02 |
| CALM1 | 1.73 (1.35 − 2.22) | 1.2E-05 | 2.4E-02 |
| IL6 | 1.68 (1.33 − 2.13) | 1.2E-05 | 2.4E-02 |
| LPHN2 | 0.59 (0.47 − 0.75) | 1.2E-05 | 2.4E-02 |
| MFAP4 | 0.59 (0.47 − 0.75) | 1.2E-05 | 2.4E-02 |
| SLC14A1 | 0.58 (0.45 − 0.74) | 1.2E-05 | 2.4E-02 |
| TTN | 0.57 (0.44 − 0.74) | 1.2E-05 | 2.4E-02 |
| ZBTB16 | 0.59 (0.46 − 0.75) | 1.2E-05 | 2.4E-02 |
| ENC1 | 0.59 (0.46 − 0.75) | 1.3E-05 | 2.6E-02 |
| GIMAP8 | 0.58 (0.45 − 0.74) | 1.3E-05 | 2.6E-02 |
| NMU | 1.69 (1.33 − 2.14) | 1.3E-05 | 2.6E-02 |
| PKNOX2 | 1.68 (1.33 − 2.12) | 1.3E-05 | 2.6E-02 |
| SYNPO | 1.71 (1.34 − 2.19) | 1.3E-05 | 2.6E-02 |
| MCEMP1 | 0.58 (0.45 − 0.75) | 1.5E-05 | 3.0E-02 |
| LINC01128 | 0.58 (0.45 − 0.75) | 1.6E-05 | 3.2E-02 |
| MRC1 | 0.60 (0.47 − 0.76) | 1.6E-05 | 3.2E-02 |
| RHNO1 | 1.74 (1.35 − 2.24) | 1.6E-05 | 3.2E-02 |
| STARD4 | 0.58 (0.45 − 0.75) | 1.6E-05 | 3.2E-02 |
| SEPT7 | 0.60 (0.47 − 0.76) | 1.7E-05 | 3.4E-02 |
| NCALD | 0.59 (0.46 − 0.75) | 1.7E-05 | 3.4E-02 |
| PALD1 | 0.57 (0.44 − 0.74) | 1.7E-05 | 3.4E-02 |
| TPPP3 | 0.60 (0.47 − 0.76) | 1.7E-05 | 3.4E-02 |
| GIPC2 | 0.58 (0.45 − 0.75) | 1.8E-05 | 3.6E-02 |
| IL18R1 | 0.60 (0.47 − 0.76) | 1.8E-05 | 3.6E-02 |
| MMP12 | 1.68 (1.32 − 2.13) | 1.8E-05 | 3.6E-02 |
| ARRB1 | 1.69 (1.32 − 2.15) | 1.9E-05 | 3.8E-02 |
| CPB2 | 0.60 (0.47 − 0.76) | 1.9E-05 | 3.8E-02 |
| LGALSL | 0.58 (0.45 − 0.75) | 1.9E-05 | 3.8E-02 |
| ASPA | 0.60 (0.47 − 0.76) | 2.0E-05 | 4.0E-02 |
| PKHD1L1 | 0.59 (0.46 − 0.76) | 2.2E-05 | 4.4E-02 |
| PHLDB2 | 0.58 (0.45 − 0.75) | 2.3E-05 | 4.6E-02 |
| C1QTNF2 | 0.59 (0.46 − 0.75) | 2.4E-05 | 4.8E-02 |
| KAL1 | 0.60 (0.47 − 0.76) | 2.4E-05 | 4.8E-02 |
| LGR4 | 0.61 (0.48 − 0.77) | 2.4E-05 | 4.8E-02 |
| LILRB2 | 1.65 (1.30 − 2.08) | 2.4E-05 | 4.8E-02 |
| IRAK3 | 0.61 (0.48 − 0.77) | 2.5E-05 | 5.0E-02 |
| VSIG10 | 0.58 (0.45 − 0.75) | 2.5E-05 | 5.0E-02 |
| BIK | 1.65 (1.30 − 2.10) | 2.8E-05 | 5.5E-02 |
| OGN | 0.60 (0.47 − 0.76) | 2.8E-05 | 5.5E-02 |
| ADPRH | 0.59 (0.46 − 0.76) | 2.9E-05 | 5.7E-02 |
| TAL1 | 0.60 (0.47 − 0.76) | 3.0E-05 | 5.9E-02 |
| FHL5 | 0.59 (0.46 − 0.76) | 3.2E-05 | 6.3E-02 |
| PYCR1 | 1.64 (1.30 − 2.08) | 3.2E-05 | 6.3E-02 |
| CCDC167 | 1.68 (1.31 − 2.15) | 3.3E-05 | 6.5E-02 |
| DNAJC22 | 1.63 (1.29 − 2.06) | 3.4E-05 | 6.7E-02 |
| SORD | 1.64 (1.30 − 2.08) | 3.4E-05 | 6.7E-02 |
| HIST1H2AE | 1.64 (1.29 − 2.07) | 3.5E-05 | 6.9E-02 |
| MAP2 | 0.60 (0.47 − 0.76) | 3.5E-05 | 6.9E-02 |
| CLIC3 | 1.64 (1.29 − 2.07) | 3.6E-05 | 7.1E-02 |
| CLDN11 | 0.60 (0.47 − 0.77) | 3.7E-05 | 7.3E-02 |
| NPR1 | 0.61 (0.48 − 0.77) | 3.8E-05 | 7.5E-02 |
| EPB41L3 | 0.61 (0.48 − 0.78) | 3.9E-05 | 7.7E-02 |
| PROS1 | 0.61 (0.48 − 0.78) | 3.9E-05 | 7.7E-02 |
| HIST1H2BD | 1.64 (1.29 − 2.08) | 4.0E-05 | 7.9E-02 |
| MS4A7 | 0.60 (0.47 − 0.77) | 4.0E-05 | 7.9E-02 |
| FGF7 | 0.61 (0.48 − 0.78) | 4.2E-05 | 8.3E-02 |
| LRRK2 | 0.60 (0.47 − 0.77) | 4.2E-05 | 8.3E-02 |
| STYXL1 | 1.66 (1.30 − 2.12) | 4.2E-05 | 8.3E-02 |
| ANGPT1 | 0.60 (0.47 − 0.77) | 4.3E-05 | 8.5E-02 |
| SLC5A3 | 1.63 (1.29 − 2.07) | 4.3E-05 | 8.5E-02 |
| DLGAP5 | 1.63 (1.29 − 2.07) | 4.7E-05 | 9.3E-02 |
| LRRC70 | 0.60 (0.47 − 0.77) | 4.7E-05 | 9.3E-02 |
| FGF14 | 0.60 (0.47 − 0.77) | 4.8E-05 | 9.5E-02 |
| LRRC36 | 0.61 (0.48 − 0.78) | 4.8E-05 | 9.5E-02 |
| SNCA | 0.60 (0.46 − 0.77) | 4.8E-05 | 9.5E-02 |
| CEP55 | 1.63 (1.28 − 2.07) | 4.9E-05 | 9.7E-02 |
| COL6A6 | 0.60 (0.47 − 0.77) | 4.9E-05 | 9.7E-02 |
| MT1E | 1.62 (1.28 − 2.05) | 4.9E-05 | 9.7E-02 |
| MT1M | 1.62 (1.28 − 2.05) | 4.9E-05 | 9.7E-02 |
| SCG5 | 1.63 (1.28 − 2.06) | 4.9E-05 | 9.7E-02 |
| FAT3 | 0.60 (0.47 − 0.77) | 5.0E-05 | 9.9E-02 |
| SYT12 | 1.65 (1.29 − 2.11) | 5.2E-05 | 1.0E-01 |
| LAGE3 | 1.61 (1.28 − 2.04) | 5.3E-05 | 1.1E-01 |
| LTBP4 | 0.61 (0.48 − 0.78) | 5.3E-05 | 1.1E-01 |
| GREM1 | 1.63 (1.28 − 2.06) | 5.4E-05 | 1.1E-01 |
| NR4A3 | 1.61 (1.27 − 2.03) | 5.5E-05 | 1.1E-01 |
| SOCS2 | 0.62 (0.49 − 0.78) | 5.6E-05 | 1.1E-01 |
| KANK4 | 0.60 (0.47 − 0.77) | 5.8E-05 | 1.1E-01 |
| THBD | 0.62 (0.49 − 0.78) | 5.9E-05 | 1.2E-01 |
| ANXA3 | 0.62 (0.49 − 0.79) | 6.2E-05 | 1.2E-01 |
| FIGNL1 | 0.61 (0.48 − 0.78) | 6.3E-05 | 1.2E-01 |
| PVT1 | 1.60 (1.27 − 2.03) | 6.7E-05 | 1.3E-01 |
| ADAMTS8 | 0.62 (0.49 − 0.79) | 6.9E-05 | 1.4E-01 |
| CCDC102B | 0.62 (0.49 − 0.79) | 6.9E-05 | 1.4E-01 |
| ELTD1 | 0.62 (0.49 − 0.79) | 7.0E-05 | 1.4E-01 |
| COL10A1 | 0.63 (0.49 − 0.79) | 7.1E-05 | 1.4E-01 |
| GAS6 | 0.61 (0.48 − 0.78) | 7.2E-05 | 1.4E-01 |
| DEPDC1B | 1.64 (1.28 − 2.09) | 7.5E-05 | 1.5E-01 |
| SOX4 | 1.61 (1.27 − 2.04) | 7.5E-05 | 1.5E-01 |
| LSR | 1.60 (1.26 − 2.04) | 9.0E-05 | 1.8E-01 |
| SVEP1 | 0.62 (0.49 − 0.79) | 9.0E-05 | 1.8E-01 |
| ALDH18A1 | 1.60 (1.26 − 2.03) | 9.1E-05 | 1.8E-01 |
| ABCA12 | 1.60 (1.26 − 2.02) | 9.5E-05 | 1.9E-01 |
| MCOLN3 | 0.62 (0.48 − 0.79) | 9.7E-05 | 1.9E-01 |
| RASL12 | 0.62 (0.49 − 0.79) | 9.7E-05 | 1.9E-01 |
| TBX2 | 0.62 (0.49 − 0.79) | 9.7E-05 | 1.9E-01 |
| E2F7 | 1.61 (1.27 − 2.06) | 9.8E-05 | 1.9E-01 |
| PPARGC1B | 0.61 (0.48 − 0.78) | 9.8E-05 | 1.9E-01 |
| ZNF423 | 0.62 (0.49 − 0.79) | 9.8E-05 | 1.9E-01 |
| EML1 | 0.63 (0.49 − 0.80) | 1.0E-04 | 2.0E-01 |
| KIAA1324L | 0.62 (0.48 − 0.79) | 1.0E-04 | 2.0E-01 |
| AKT3 | 0.61 (0.48 − 0.79) | 1.1E-04 | 2.2E-01 |
| XPR1 | 0.62 (0.48 − 0.79) | 1.1E-04 | 2.2E-01 |
| BET1 | 0.63 (0.50 − 0.80) | 1.2E-04 | 2.4E-01 |
| GFOD1 | 0.61 (0.47 − 0.79) | 1.2E-04 | 2.4E-01 |
| ICAM2 | 1.58 (1.25 − 2.00) | 1.2E-04 | 2.4E-01 |
| MNDA | 0.63 (0.50 − 0.80) | 1.2E-04 | 2.4E-01 |
| PHEX | 1.57 (1.25 − 1.99) | 1.2E-04 | 2.4E-01 |
| CENPM | 1.58 (1.25 − 1.99) | 1.3E-04 | 2.6E-01 |
| FPR2 | 1.57 (1.24 − 1.99) | 1.3E-04 | 2.6E-01 |
| HEYL | 1.57 (1.24 − 1.98) | 1.3E-04 | 2.6E-01 |
| LIMD1 | 1.58 (1.25 − 2.00) | 1.3E-04 | 2.6E-01 |
| MRGPRF | 0.62 (0.48 − 0.79) | 1.3E-04 | 2.6E-01 |
| STK39 | 0.64 (0.50 − 0.80) | 1.3E-04 | 2.6E-01 |
| ARL13B | 0.62 (0.49 − 0.80) | 1.4E-04 | 2.8E-01 |
| BUB1B | 1.57 (1.24 − 1.99) | 1.4E-04 | 2.8E-01 |
| DPT | 0.63 (0.50 − 0.80) | 1.4E-04 | 2.8E-01 |
| GJA5 | 1.57 (1.24 − 1.99) | 1.4E-04 | 2.8E-01 |
| PCDH17 | 0.62 (0.49 − 0.80) | 1.4E-04 | 2.8E-01 |
| COL11A1 | 1.57 (1.24 − 1.99) | 1.5E-04 | 3.0E-01 |
| FLT1 | 1.57 (1.24 − 1.98) | 1.5E-04 | 3.0E-01 |
| FRMD3 | 0.62 (0.48 − 0.79) | 1.5E-04 | 3.0E-01 |
| IFRD1 | 0.62 (0.49 − 0.80) | 1.5E-04 | 3.0E-01 |
| LDLR | 1.57 (1.24 − 1.98) | 1.5E-04 | 3.0E-01 |
| NDRG2 | 1.57 (1.24 − 1.99) | 1.5E-04 | 3.0E-01 |
| SLC6A13 | 0.62 (0.48 − 0.80) | 1.5E-04 | 3.0E-01 |
| LAMP3 | 0.64 (0.50 − 0.81) | 1.6E-04 | 3.2E-01 |
| THSD7A | 0.62 (0.49 − 0.80) | 1.6E-04 | 3.2E-01 |
| FA2H | 1.59 (1.25 − 2.03) | 1.7E-04 | 3.4E-01 |
| FIBIN | 0.63 (0.49 − 0.80) | 1.7E-04 | 3.4E-01 |
| FUT8 | 1.61 (1.25 − 2.07) | 1.7E-04 | 3.4E-01 |
| GPR126 | 0.64 (0.51 − 0.81) | 1.7E-04 | 3.4E-01 |
| NCK1 | 0.64 (0.51 − 0.81) | 1.7E-04 | 3.4E-01 |
| TM6SF1 | 0.64 (0.50 − 0.81) | 1.7E-04 | 3.4E-01 |
| ANP32E | 0.64 (0.51 − 0.81) | 1.8E-04 | 3.6E-01 |
| ASPM | 1.57 (1.24 − 2.00) | 1.8E-04 | 3.6E-01 |
| DLG3 | 1.56 (1.24 − 1.98) | 1.8E-04 | 3.6E-01 |
| PALM2-AKAP2 | 0.64 (0.50 − 0.81) | 1.8E-04 | 3.6E-01 |
| FAXC | 0.63 (0.49 − 0.81) | 1.9E-04 | 3.8E-01 |
| MELK | 1.56 (1.23 − 1.97) | 1.9E-04 | 3.8E-01 |
| OSR1 | 1.58 (1.24 − 2.01) | 1.9E-04 | 3.8E-01 |
| ATOH8 | 0.63 (0.49 − 0.80) | 2.0E-04 | 4.0E-01 |
| OR7E47P | 0.63 (0.49 − 0.81) | 2.0E-04 | 4.0E-01 |
| TNNC1 | 1.55 (1.23 − 1.96) | 2.0E-04 | 4.0E-01 |
| CFD | 0.64 (0.51 − 0.81) | 2.1E-04 | 4.2E-01 |
| DIXDC1 | 0.62 (0.49 − 0.80) | 2.1E-04 | 4.2E-01 |
| EGLN3 | 1.58 (1.24 − 2.02) | 2.1E-04 | 4.2E-01 |
| F2RL1 | 0.64 (0.51 − 0.81) | 2.1E-04 | 4.2E-01 |
| S100A4 | 1.57 (1.23 − 1.99) | 2.1E-04 | 4.2E-01 |
| CXorf57 | 0.64 (0.51 − 0.81) | 2.2E-04 | 4.4E-01 |
| KLRF1 | 0.64 (0.51 − 0.81) | 2.2E-04 | 4.4E-01 |
| OPTN | 0.64 (0.51 − 0.82) | 2.2E-04 | 4.4E-01 |
| PKIA | 0.63 (0.50 − 0.81) | 2.3E-04 | 4.6E-01 |
| CASKIN2 | 1.54 (1.22 − 1.95) | 2.4E-04 | 4.8E-01 |
| COL5A1 | 1.54 (1.22 − 1.95) | 2.4E-04 | 4.8E-01 |
| COLGALT2 | 1.56 (1.23 − 1.99) | 2.4E-04 | 4.8E-01 |
| HMBOX1 | 0.63 (0.49 − 0.81) | 2.4E-04 | 4.8E-01 |
| SPAG4 | 1.55 (1.22 − 1.96) | 2.4E-04 | 4.8E-01 |
| DHRS11 | 1.55 (1.22 − 1.96) | 2.5E-04 | 5.0E-01 |
| LINC00467 | 0.64 (0.50 − 0.81) | 2.5E-04 | 5.0E-01 |
| PCAT19 | 0.63 (0.49 − 0.81) | 2.5E-04 | 5.0E-01 |
| PDE1C | 0.63 (0.49 − 0.81) | 2.5E-04 | 5.0E-01 |
| CCM2L | 0.63 (0.49 − 0.81) | 2.6E-04 | 5.2E-01 |
| RASSF2 | 0.64 (0.51 − 0.82) | 2.6E-04 | 5.2E-01 |
| EIF4E3 | 1.58 (1.23 − 2.03) | 2.7E-04 | 5.4E-01 |
| FGFBP2 | 0.63 (0.49 − 0.81) | 2.7E-04 | 5.4E-01 |
| TRPV2 | 1.56 (1.23 − 1.99) | 2.7E-04 | 5.4E-01 |
| CCDC50 | 0.63 (0.49 − 0.81) | 2.8E-04 | 5.5E-01 |
| OSCAR | 1.56 (1.22 − 1.99) | 2.8E-04 | 5.5E-01 |
| BUB1 | 1.53 (1.22 − 1.94) | 2.9E-04 | 5.7E-01 |
| NDC1 | 0.64 (0.50 − 0.81) | 2.9E-04 | 5.7E-01 |
| CBFA2T3 | 0.64 (0.50 − 0.82) | 3.0E-04 | 5.9E-01 |
| FAM107A | 0.64 (0.50 − 0.82) | 3.0E-04 | 5.9E-01 |
| NOSTRIN | 0.64 (0.50 − 0.82) | 3.1E-04 | 6.1E-01 |
| AGTR2 | 1.53 (1.21 − 1.93) | 3.2E-04 | 6.3E-01 |
| NTNG1 | 0.64 (0.50 − 0.82) | 3.2E-04 | 6.3E-01 |
| PLCB4 | 0.65 (0.52 − 0.82) | 3.3E-04 | 6.5E-01 |
| SLIT3 | 0.65 (0.51 − 0.82) | 3.3E-04 | 6.5E-01 |
| XKRX | 0.64 (0.50 − 0.82) | 3.3E-04 | 6.5E-01 |
| BMPR2 | 1.56 (1.22 − 2.00) | 3.4E-04 | 6.7E-01 |
| C15orf48 | 1.56 (1.22 − 1.99) | 3.4E-04 | 6.7E-01 |
| CLDN12 | 0.64 (0.50 − 0.82) | 3.4E-04 | 6.7E-01 |
| EIF1 | 1.53 (1.21 − 1.94) | 3.4E-04 | 6.7E-01 |
| KLF2 | 0.65 (0.51 − 0.82) | 3.4E-04 | 6.7E-01 |
| ABCB1 | 0.65 (0.52 − 0.83) | 3.5E-04 | 6.9E-01 |
| CDH3 | 1.53 (1.21 − 1.93) | 3.5E-04 | 6.9E-01 |
| NFATC1 | 0.65 (0.51 − 0.82) | 3.5E-04 | 6.9E-01 |
| AOC1 | 1.53 (1.21 − 1.93) | 3.6E-04 | 7.1E-01 |
| CASQ2 | 0.65 (0.51 − 0.82) | 3.6E-04 | 7.1E-01 |
| EPB41L5 | 0.65 (0.51 − 0.82) | 3.8E-04 | 7.5E-01 |
| S100A3 | 1.52 (1.21 − 1.92) | 3.8E-04 | 7.5E-01 |
| AK2 | 1.54 (1.21 − 1.95) | 3.9E-04 | 7.7E-01 |
| MFAP2 | 1.53 (1.21 − 1.94) | 3.9E-04 | 7.7E-01 |
| MMP1 | 1.53 (1.21 − 1.94) | 3.9E-04 | 7.7E-01 |
| RAB11FIP1 | 0.66 (0.52 − 0.83) | 3.9E-04 | 7.7E-01 |
| PRKCE | 0.64 (0.50 − 0.82) | 4.0E-04 | 7.9E-01 |
| ZNF713 | 1.53 (1.21 − 1.95) | 4.0E-04 | 7.9E-01 |
| NUP62CL | 1.53 (1.20 − 1.93) | 4.2E-04 | 8.3E-01 |
| PHKA1 | 0.65 (0.51 − 0.83) | 4.2E-04 | 8.3E-01 |
| PPAT | 0.66 (0.52 − 0.83) | 4.2E-04 | 8.3E-01 |
| TSPAN6 | 0.66 (0.52 − 0.83) | 4.2E-04 | 8.3E-01 |
| XDH | 1.52 (1.20 − 1.91) | 4.2E-04 | 8.3E-01 |
| ACADL | 0.66 (0.52 − 0.83) | 4.4E-04 | 8.7E-01 |
| TMEM139 | 0.65 (0.51 − 0.83) | 4.5E-04 | 8.9E-01 |
| CCND2 | 1.51 (1.20 − 1.91) | 4.6E-04 | 9.1E-01 |
| SEMA5A | 0.65 (0.51 − 0.83) | 4.6E-04 | 9.1E-01 |
| INPP1 | 0.66 (0.52 − 0.83) | 4.7E-04 | 9.3E-01 |
| ITIH5 | 0.65 (0.51 − 0.83) | 4.7E-04 | 9.3E-01 |
| ABCA3 | 0.66 (0.52 − 0.83) | 4.8E-04 | 9.5E-01 |
| EMP2 | 0.65 (0.51 − 0.83) | 4.8E-04 | 9.5E-01 |
| LPHN3 | 0.66 (0.52 − 0.83) | 4.8E-04 | 9.5E-01 |
| ABLIM1 | 0.65 (0.51 − 0.83) | 5.0E-04 | 9.9E-01 |
| ARHGAP31 | 0.65 (0.50 − 0.83) | 5.0E-04 | 9.9E-01 |
| ADAMTSL4 | 1.54 (1.20 − 1.96) | 5.1E-04 | 1.0E+00 |
| SPTBN1 | 0.65 (0.50 − 0.83) | 5.2E-04 | 1.0E+00 |
| DPEP2 | 0.66 (0.52 − 0.84) | 5.4E-04 | 1.1E+00 |
| NUF2 | 1.54 (1.20 − 1.96) | 5.4E-04 | 1.1E+00 |
| GFPT1 | 0.66 (0.53 − 0.84) | 5.5E-04 | 1.1E+00 |
| NQO1 | 0.66 (0.53 − 0.84) | 5.6E-04 | 1.1E+00 |
| SRD5A1 | 0.66 (0.52 − 0.84) | 5.6E-04 | 1.1E+00 |
| CAV2 | 1.51 (1.19 − 1.91) | 5.7E-04 | 1.1E+00 |
| ABCG1 | 0.66 (0.52 − 0.84) | 5.8E-04 | 1.1E+00 |
| TXNDC17 | 1.54 (1.20 − 1.98) | 5.9E-04 | 1.2E+00 |
| ARHGAP23 | 1.52 (1.19 − 1.94) | 6.1E-04 | 1.2E+00 |
| C1orf53 | 1.55 (1.21 − 2.00) | 6.1E-04 | 1.2E+00 |
| MARVELD3 | 0.66 (0.51 − 0.84) | 6.6E-04 | 1.3E+00 |
| SLC6A16 | 0.66 (0.52 − 0.84) | 6.6E-04 | 1.3E+00 |
| TCEAL7 | 0.66 (0.51 − 0.84) | 6.7E-04 | 1.3E+00 |
| CLDN3 | 1.51 (1.19 − 1.91) | 6.8E-04 | 1.3E+00 |
| NPY1R | 0.67 (0.53 − 0.84) | 6.8E-04 | 1.3E+00 |
| ACTR3 | 0.67 (0.53 − 0.85) | 6.9E-04 | 1.4E+00 |
| ST7L | 0.66 (0.51 − 0.84) | 7.0E-04 | 1.4E+00 |
| AQP4 | 0.66 (0.52 − 0.84) | 7.1E-04 | 1.4E+00 |
| HLA-E | 0.67 (0.53 − 0.85) | 7.1E-04 | 1.4E+00 |
| STARD9 | 0.65 (0.51 − 0.84) | 7.2E-04 | 1.4E+00 |
| EPB41L2 | 1.49 (1.18 − 1.89) | 7.7E-04 | 1.5E+00 |
| APOLD1 | 0.67 (0.52 − 0.85) | 7.8E-04 | 1.5E+00 |
| FHL2 | 1.49 (1.18 − 1.88) | 7.8E-04 | 1.5E+00 |
| MS4A2 | 0.67 (0.53 − 0.85) | 7.8E-04 | 1.5E+00 |
| SEZ6L2 | 1.51 (1.18 − 1.93) | 8.1E-04 | 1.6E+00 |
| RFX2 | 0.66 (0.51 − 0.84) | 8.2E-04 | 1.6E+00 |
| PTPLA | 0.67 (0.53 − 0.85) | 8.4E-04 | 1.7E+00 |
| ATAD2 | 1.50 (1.18 − 1.90) | 8.7E-04 | 1.7E+00 |
| ABCA9 | 0.65 (0.51 − 0.84) | 8.8E-04 | 1.7E+00 |
| CNTN6 | 0.67 (0.53 − 0.85) | 8.9E-04 | 1.8E+00 |
| C3orf14 | 0.66 (0.52 − 0.85) | 9.0E-04 | 1.8E+00 |
| BNIP2 | 0.68 (0.54 − 0.85) | 9.1E-04 | 1.8E+00 |
| CTTN | 1.48 (1.17 − 1.88) | 9.2E-04 | 1.8E+00 |
| ROBO4 | 1.48 (1.17 − 1.87) | 9.2E-04 | 1.8E+00 |
| SCN1A | 0.67 (0.53 − 0.85) | 9.3E-04 | 1.8E+00 |
| CAB39L | 0.67 (0.53 − 0.85) | 9.5E-04 | 1.9E+00 |
| CLEC2B | 0.68 (0.54 − 0.85) | 9.8E-04 | 1.9E+00 |
| DENND3 | 0.67 (0.52 − 0.85) | 1.0E-03 | 2.0E+00 |
| INSIG1 | 0.68 (0.53 − 0.86) | 1.1E-03 | 2.2E+00 |
| KIAA1462 | 0.66 (0.52 − 0.85) | 1.1E-03 | 2.2E+00 |
| OSMR | 1.48 (1.17 − 1.88) | 1.1E-03 | 2.2E+00 |
| TMPRSS4 | 1.48 (1.17 − 1.87) | 1.1E-03 | 2.2E+00 |
| ABI3BP | 0.67 (0.52 − 0.85) | 1.2E-03 | 2.4E+00 |
| AMICA1 | 0.67 (0.52 − 0.85) | 1.2E-03 | 2.4E+00 |
| AOX1 | 0.68 (0.54 − 0.86) | 1.2E-03 | 2.4E+00 |
| CLIC5 | 0.67 (0.52 − 0.85) | 1.2E-03 | 2.4E+00 |
| CMKLR1 | 1.46 (1.16 − 1.85) | 1.2E-03 | 2.4E+00 |
| ERO1L | 1.50 (1.17 − 1.91) | 1.2E-03 | 2.4E+00 |
| FOXF1 | 0.68 (0.53 − 0.86) | 1.2E-03 | 2.4E+00 |
| GALNT7 | 0.68 (0.54 − 0.86) | 1.2E-03 | 2.4E+00 |
| IL18RAP | 0.68 (0.53 − 0.86) | 1.2E-03 | 2.4E+00 |
| TMTC1 | 0.67 (0.53 − 0.86) | 1.2E-03 | 2.4E+00 |
| ARHGAP18 | 0.67 (0.53 − 0.86) | 1.3E-03 | 2.6E+00 |
| DES | 0.68 (0.54 − 0.86) | 1.3E-03 | 2.6E+00 |
| GYG2 | 0.68 (0.54 − 0.86) | 1.3E-03 | 2.6E+00 |
| KIF15 | 1.47 (1.16 − 1.86) | 1.3E-03 | 2.6E+00 |
| FAM167A | 0.67 (0.53 − 0.86) | 1.4E-03 | 2.8E+00 |
| SSTR1 | 0.67 (0.53 − 0.86) | 1.4E-03 | 2.8E+00 |
| C9orf72 | 0.67 (0.53 − 0.86) | 1.5E-03 | 3.0E+00 |
| KRT4 | 0.68 (0.53 − 0.86) | 1.5E-03 | 3.0E+00 |
| SPP1 | 1.46 (1.16 − 1.85) | 1.5E-03 | 3.0E+00 |
| CENPE | 1.46 (1.15 − 1.86) | 1.7E-03 | 3.4E+00 |
| FAM43A | 1.47 (1.15 − 1.87) | 1.7E-03 | 3.4E+00 |
| GPIHBP1 | 0.67 (0.53 − 0.86) | 1.7E-03 | 3.4E+00 |
| GRIA1 | 0.68 (0.54 − 0.87) | 1.7E-03 | 3.4E+00 |
| LYPD1 | 1.45 (1.15 − 1.83) | 1.7E-03 | 3.4E+00 |
| RXFP1 | 0.68 (0.53 − 0.87) | 1.7E-03 | 3.4E+00 |
| CFL2 | 1.48 (1.16 − 1.90) | 1.8E-03 | 3.6E+00 |
| COL3A1 | 1.45 (1.15 − 1.84) | 1.8E-03 | 3.6E+00 |
| DPY19L1 | 0.69 (0.55 − 0.87) | 1.8E-03 | 3.6E+00 |
| PDE8B | 0.68 (0.53 − 0.87) | 1.8E-03 | 3.6E+00 |
| PREX1 | 0.67 (0.53 − 0.86) | 1.8E-03 | 3.6E+00 |
| ADAM28 | 0.69 (0.54 − 0.87) | 1.9E-03 | 3.8E+00 |
| ADAMTS9 | 1.46 (1.15 − 1.87) | 1.9E-03 | 3.8E+00 |
| CD274 | 1.47 (1.15 − 1.87) | 1.9E-03 | 3.8E+00 |
| CEACAM5 | 1.45 (1.15 − 1.83) | 1.9E-03 | 3.8E+00 |
| DHTKD1 | 0.68 (0.54 − 0.87) | 1.9E-03 | 3.8E+00 |
| FPR1 | 1.44 (1.14 − 1.82) | 1.9E-03 | 3.8E+00 |
| NOS1 | 1.44 (1.14 − 1.82) | 1.9E-03 | 3.8E+00 |
| NR5A2 | 0.69 (0.54 − 0.87) | 1.9E-03 | 3.8E+00 |
| ALOX5 | 0.69 (0.55 − 0.87) | 2.0E-03 | 4.0E+00 |
| C10orf54 | 0.67 (0.53 − 0.87) | 2.0E-03 | 4.0E+00 |
| COL1A2 | 1.44 (1.14 − 1.82) | 2.0E-03 | 4.0E+00 |
| MAFF | 0.69 (0.55 − 0.87) | 2.0E-03 | 4.0E+00 |
| NHSL1 | 1.46 (1.15 − 1.87) | 2.0E-03 | 4.0E+00 |
| STON1 | 0.69 (0.55 − 0.88) | 2.0E-03 | 4.0E+00 |
| ARHGEF26 | 0.68 (0.54 − 0.87) | 2.1E-03 | 4.2E+00 |
| B3GALNT1 | 1.37 (1.12 − 1.68) | 2.1E-03 | 4.2E+00 |
| GRTP1 | 0.68 (0.54 − 0.87) | 2.1E-03 | 4.2E+00 |
| TIE1 | 1.44 (1.14 − 1.82) | 2.1E-03 | 4.2E+00 |
| ABHD6 | 0.69 (0.55 − 0.88) | 2.2E-03 | 4.4E+00 |
| CARD16 | 0.69 (0.54 − 0.87) | 2.2E-03 | 4.4E+00 |
| CD36 | 0.70 (0.55 − 0.88) | 2.3E-03 | 4.6E+00 |
| FAM101B | 0.68 (0.53 − 0.87) | 2.3E-03 | 4.6E+00 |
| FBLN5 | 0.70 (0.55 − 0.88) | 2.3E-03 | 4.6E+00 |
| RAMP2 | 1.44 (1.14 − 1.82) | 2.3E-03 | 4.6E+00 |
| VWF | 1.45 (1.14 − 1.85) | 2.3E-03 | 4.6E+00 |
| CORO1C | 1.43 (1.14 − 1.81) | 2.4E-03 | 4.8E+00 |
| PTPN5 | 0.68 (0.53 − 0.87) | 2.4E-03 | 4.8E+00 |
| SDC1 | 1.44 (1.14 − 1.83) | 2.4E-03 | 4.8E+00 |
| SEMA4B | 1.45 (1.14 − 1.85) | 2.5E-03 | 5.0E+00 |
| HIST1H2BC | 1.43 (1.13 − 1.81) | 2.6E-03 | 5.2E+00 |
| PCP4 | 1.43 (1.13 − 1.81) | 2.6E-03 | 5.2E+00 |
| CELF2 | 1.45 (1.13 − 1.84) | 2.7E-03 | 5.4E+00 |
| LMO2 | 0.70 (0.55 − 0.88) | 2.7E-03 | 5.4E+00 |
| OSBPL3 | 0.70 (0.55 − 0.88) | 2.7E-03 | 5.4E+00 |
| PTGFRN | 1.45 (1.14 − 1.84) | 2.7E-03 | 5.4E+00 |
| TLR8 | 0.69 (0.54 − 0.88) | 2.7E-03 | 5.4E+00 |
| FXYD6 | 0.70 (0.55 − 0.88) | 2.8E-03 | 5.5E+00 |
| ABCG2 | 0.70 (0.55 − 0.88) | 2.9E-03 | 5.7E+00 |
| CD24 | 0.70 (0.56 − 0.89) | 2.9E-03 | 5.7E+00 |
| PVRL4 | 1.44 (1.13 − 1.84) | 2.9E-03 | 5.7E+00 |
| NR4A2 | 0.70 (0.56 − 0.89) | 3.0E-03 | 5.9E+00 |
| ATP2A2 | 1.42 (1.12 − 1.79) | 3.1E-03 | 6.1E+00 |
| TCEAL2 | 0.70 (0.55 − 0.89) | 3.2E-03 | 6.3E+00 |
| DKK2 | 0.70 (0.56 − 0.89) | 3.3E-03 | 6.5E+00 |
| CDH5 | 0.70 (0.56 − 0.89) | 3.4E-03 | 6.7E+00 |
| RALGPS2 | 0.70 (0.55 − 0.89) | 3.4E-03 | 6.7E+00 |
| KLF4 | 0.71 (0.56 − 0.89) | 3.5E-03 | 6.9E+00 |
| SRPX2 | 1.41 (1.12 − 1.78) | 3.5E-03 | 6.9E+00 |
| ERG | 0.70 (0.55 − 0.89) | 3.6E-03 | 7.1E+00 |
| WNT3 | 0.70 (0.55 − 0.89) | 3.6E-03 | 7.1E+00 |
| EPN3 | 1.41 (1.12 − 1.78) | 3.8E-03 | 7.5E+00 |
| CD52 | 1.41 (1.12 − 1.78) | 3.9E-03 | 7.7E+00 |
| HBA2 | 1.41 (1.12 − 1.79) | 3.9E-03 | 7.7E+00 |
| FGFR4 | 0.71 (0.56 − 0.90) | 4.0E-03 | 7.9E+00 |
| KISS1R | 1.43 (1.12 − 1.82) | 4.0E-03 | 7.9E+00 |
| PEAR1 | 0.70 (0.54 − 0.89) | 4.0E-03 | 7.9E+00 |
| SBK1 | 0.70 (0.55 − 0.89) | 4.0E-03 | 7.9E+00 |
| CCDC85A | 0.70 (0.55 − 0.90) | 4.1E-03 | 8.1E+00 |
| FAM189A2 | 0.71 (0.56 − 0.90) | 4.1E-03 | 8.1E+00 |
| CAV1 | 0.71 (0.56 − 0.90) | 4.2E-03 | 8.3E+00 |
| CD300LG | 0.70 (0.55 − 0.90) | 4.3E-03 | 8.5E+00 |
| ARHGAP28 | 1.40 (1.11 − 1.77) | 4.4E-03 | 8.7E+00 |
| REEP1 | 0.71 (0.56 − 0.90) | 4.4E-03 | 8.7E+00 |
| SERINC2 | 1.42 (1.11 − 1.81) | 4.4E-03 | 8.7E+00 |
| NTRK3 | 0.71 (0.55 − 0.90) | 4.7E-03 | 9.3E+00 |
| VWA1 | 1.40 (1.11 − 1.77) | 4.7E-03 | 9.3E+00 |
| DACH1 | 0.70 (0.55 − 0.90) | 4.8E-03 | 9.5E+00 |
| RARRES2 | 1.40 (1.11 − 1.77) | 4.8E-03 | 9.5E+00 |
| CSF3R | 1.39 (1.10 − 1.76) | 4.9E-03 | 9.7E+00 |
| CTHRC1 | 1.41 (1.11 − 1.80) | 4.9E-03 | 9.7E+00 |
| CALCRL | 0.72 (0.57 − 0.91) | 5.1E-03 | 1.0E+01 |
| COL17A1 | 1.39 (1.10 − 1.76) | 5.1E-03 | 1.0E+01 |
| CYP2U1 | 1.39 (1.10 − 1.76) | 5.1E-03 | 1.0E+01 |
| DGKE | 0.71 (0.55 − 0.90) | 5.2E-03 | 1.0E+01 |
| SLC6A4 | 0.71 (0.55 − 0.90) | 5.2E-03 | 1.0E+01 |
| ANGPTL1 | 0.71 (0.56 − 0.90) | 5.3E-03 | 1.1E+01 |
| CEACAM1 | 0.71 (0.56 − 0.90) | 5.3E-03 | 1.1E+01 |
| SPATS2L | 0.72 (0.57 − 0.91) | 5.3E-03 | 1.1E+01 |
| TDRKH | 0.72 (0.57 − 0.91) | 5.3E-03 | 1.1E+01 |
| CDCA2 | 1.41 (1.10 − 1.79) | 5.5E-03 | 1.1E+01 |
| ADAMDEC1 | 1.39 (1.10 − 1.76) | 5.6E-03 | 1.1E+01 |
| EGFL7 | 1.39 (1.10 − 1.75) | 5.6E-03 | 1.1E+01 |
| MSR1 | 1.39 (1.10 − 1.75) | 5.9E-03 | 1.2E+01 |
| ANKDD1A | 0.71 (0.56 − 0.91) | 6.0E-03 | 1.2E+01 |
| NR4A1 | 1.38 (1.10 − 1.75) | 6.0E-03 | 1.2E+01 |
| RGS2 | 0.72 (0.57 − 0.91) | 6.1E-03 | 1.2E+01 |
| PPP1R14A | 0.71 (0.56 − 0.91) | 6.3E-03 | 1.2E+01 |
| EBF1 | 0.71 (0.56 − 0.91) | 6.4E-03 | 1.3E+01 |
| PAFAH1B3 | 1.38 (1.09 − 1.75) | 6.6E-03 | 1.3E+01 |
| AFAP1-AS1 | 0.72 (0.56 − 0.91) | 6.7E-03 | 1.3E+01 |
| FBLN1 | 1.38 (1.09 − 1.74) | 6.9E-03 | 1.4E+01 |
| FAM65A | 1.39 (1.09 − 1.77) | 7.0E-03 | 1.4E+01 |
| FAM83H | 1.39 (1.09 − 1.77) | 7.0E-03 | 1.4E+01 |
| SLC39A4 | 1.38 (1.09 − 1.74) | 7.0E-03 | 1.4E+01 |
| ZBED2 | 1.38 (1.09 − 1.74) | 7.0E-03 | 1.4E+01 |
| CCBE1 | 0.72 (0.56 − 0.91) | 7.2E-03 | 1.4E+01 |
| CCDC34 | 1.40 (1.09 − 1.78) | 7.2E-03 | 1.4E+01 |
| E2F8 | 1.38 (1.09 − 1.74) | 7.2E-03 | 1.4E+01 |
| SBSPON | 0.72 (0.56 − 0.92) | 7.5E-03 | 1.5E+01 |
| TEX14 | 0.73 (0.58 − 0.92) | 7.7E-03 | 1.5E+01 |
| KPNA2 | 1.38 (1.09 − 1.74) | 7.9E-03 | 1.6E+01 |
| ARHGAP6 | 1.37 (1.08 − 1.72) | 8.2E-03 | 1.6E+01 |
| EMCN | 0.73 (0.58 − 0.92) | 8.3E-03 | 1.6E+01 |
| HLX | 0.72 (0.57 − 0.92) | 8.3E-03 | 1.6E+01 |
| AHNAK | 0.72 (0.56 − 0.92) | 8.4E-03 | 1.7E+01 |
| GINS1 | 1.37 (1.08 − 1.73) | 8.5E-03 | 1.7E+01 |
| TMEM177 | 1.37 (1.08 − 1.72) | 8.6E-03 | 1.7E+01 |
| CP | 0.73 (0.57 − 0.92) | 8.8E-03 | 1.7E+01 |
| DDR2 | 0.72 (0.57 − 0.92) | 8.8E-03 | 1.7E+01 |
| HIGD1B | 0.73 (0.58 − 0.93) | 8.8E-03 | 1.7E+01 |
| MCAM | 1.37 (1.08 − 1.73) | 8.9E-03 | 1.8E+01 |
| SLC41A2 | 0.73 (0.57 − 0.92) | 9.1E-03 | 1.8E+01 |
| ANXA8L1 | 1.36 (1.08 − 1.72) | 9.2E-03 | 1.8E+01 |
| C10orf128 | 0.62 (0.43 − 0.89) | 9.2E-03 | 1.8E+01 |
| SCARF1 | 0.73 (0.58 − 0.93) | 9.2E-03 | 1.8E+01 |
| WFS1 | 0.73 (0.57 − 0.93) | 9.2E-03 | 1.8E+01 |
| CLEC12A | 0.73 (0.57 − 0.93) | 9.4E-03 | 1.9E+01 |
| GDF10 | 0.73 (0.58 − 0.93) | 9.4E-03 | 1.9E+01 |
| C10orf67 | 1.38 (1.08 − 1.76) | 9.5E-03 | 1.9E+01 |
| CAT | 0.73 (0.58 − 0.93) | 9.7E-03 | 1.9E+01 |
| CDH19 | 0.73 (0.58 − 0.93) | 9.8E-03 | 1.9E+01 |
| PDLIM2 | 0.74 (0.58 − 0.93) | 9.8E-03 | 1.9E+01 |
| CEBPD | 0.73 (0.58 − 0.93) | 1.0E-02 | 2.0E+01 |
| GGCT | 0.74 (0.58 − 0.93) | 1.0E-02 | 2.0E+01 |
| GLIPR2 | 0.73 (0.57 − 0.93) | 1.0E-02 | 2.0E+01 |
| MMP9 | 1.36 (1.07 − 1.71) | 1.0E-02 | 2.0E+01 |
| AKAP12 | 0.74 (0.58 − 0.93) | 1.1E-02 | 2.2E+01 |
| GSTM5 | 0.73 (0.58 − 0.93) | 1.1E-02 | 2.2E+01 |
| LOC100506725 | 0.73 (0.57 − 0.93) | 1.1E-02 | 2.2E+01 |
| MFAP3L | 0.74 (0.58 − 0.93) | 1.1E-02 | 2.2E+01 |
| SGCG | 0.73 (0.58 − 0.93) | 1.1E-02 | 2.2E+01 |
| SMIM22 | 1.37 (1.07 − 1.75) | 1.1E-02 | 2.2E+01 |
| ZWINT | 1.35 (1.07 − 1.71) | 1.1E-02 | 2.2E+01 |
| CRABP2 | 1.35 (1.07 − 1.70) | 1.2E-02 | 2.4E+01 |
| HHIP | 0.73 (0.57 − 0.94) | 1.2E-02 | 2.4E+01 |
| RRAS | 1.35 (1.07 − 1.70) | 1.2E-02 | 2.4E+01 |
| TFAP2C | 0.74 (0.59 − 0.94) | 1.2E-02 | 2.4E+01 |
| GMDS | 0.74 (0.59 − 0.94) | 1.3E-02 | 2.6E+01 |
| HIST1H4J | 1.35 (1.06 − 1.70) | 1.3E-02 | 2.6E+01 |
| ITGA11 | 1.36 (1.07 − 1.73) | 1.3E-02 | 2.6E+01 |
| CCNA2 | 1.34 (1.06 − 1.69) | 1.4E-02 | 2.8E+01 |
| CLU | 0.75 (0.59 − 0.94) | 1.4E-02 | 2.8E+01 |
| CPPED1 | 0.74 (0.58 − 0.94) | 1.4E-02 | 2.8E+01 |
| KIF23 | 1.34 (1.06 − 1.70) | 1.4E-02 | 2.8E+01 |
| NPM1 | 1.35 (1.06 − 1.72) | 1.4E-02 | 2.8E+01 |
| RNF43 | 0.74 (0.59 − 0.94) | 1.4E-02 | 2.8E+01 |
| SGCE | 0.75 (0.59 − 0.94) | 1.4E-02 | 2.8E+01 |
| SIX4 | 0.74 (0.58 − 0.94) | 1.4E-02 | 2.8E+01 |
| PDCD2L | 1.35 (1.06 − 1.72) | 1.5E-02 | 3.0E+01 |
| DCN | 0.75 (0.60 − 0.95) | 1.6E-02 | 3.2E+01 |
| GMNN | 0.75 (0.60 − 0.95) | 1.6E-02 | 3.2E+01 |
| GPR146 | 0.74 (0.58 − 0.95) | 1.6E-02 | 3.2E+01 |
| KDELR2 | 1.28 (1.05 − 1.56) | 1.6E-02 | 3.2E+01 |
| MVB12B | 1.35 (1.06 − 1.72) | 1.6E-02 | 3.2E+01 |
| PDIA4 | 1.33 (1.05 − 1.68) | 1.6E-02 | 3.2E+01 |
| PEBP4 | 0.74 (0.58 − 0.95) | 1.6E-02 | 3.2E+01 |
| TSPAN18 | 0.74 (0.58 − 0.95) | 1.6E-02 | 3.2E+01 |
| CLDN18 | 0.75 (0.60 − 0.95) | 1.7E-02 | 3.4E+01 |
| LOC643733 | 0.75 (0.58 − 0.95) | 1.7E-02 | 3.4E+01 |
| MGAM | 0.75 (0.59 − 0.95) | 1.7E-02 | 3.4E+01 |
| SFXN1 | 1.35 (1.05 − 1.72) | 1.7E-02 | 3.4E+01 |
| ST6GALNAC5 | 0.74 (0.58 − 0.95) | 1.7E-02 | 3.4E+01 |
| AIF1L | 0.74 (0.58 − 0.95) | 1.8E-02 | 3.6E+01 |
| CCDC69 | 1.34 (1.05 − 1.71) | 1.8E-02 | 3.6E+01 |
| CDKN2B | 1.32 (1.05 − 1.67) | 1.8E-02 | 3.6E+01 |
| CLDND1 | 0.75 (0.59 − 0.95) | 1.8E-02 | 3.6E+01 |
| EDN1 | 0.74 (0.58 − 0.95) | 1.8E-02 | 3.6E+01 |
| MYL9 | 1.32 (1.05 − 1.67) | 1.9E-02 | 3.8E+01 |
| B3GNT3 | 1.27 (1.04 − 1.55) | 2.0E-02 | 4.0E+01 |
| FOXO6 | 0.75 (0.59 − 0.96) | 2.0E-02 | 4.0E+01 |
| WDHD1 | 1.32 (1.04 − 1.66) | 2.0E-02 | 4.0E+01 |
| ADAMTS1 | 0.75 (0.59 − 0.96) | 2.1E-02 | 4.2E+01 |
| ECT2 | 1.33 (1.04 − 1.69) | 2.1E-02 | 4.2E+01 |
| FBXO32 | 0.75 (0.59 − 0.96) | 2.1E-02 | 4.2E+01 |
| SSR4 | 1.32 (1.04 − 1.68) | 2.1E-02 | 4.2E+01 |
| PLCXD3 | 0.75 (0.59 − 0.96) | 2.2E-02 | 4.4E+01 |
| LINC00312 | 0.75 (0.59 − 0.96) | 2.3E-02 | 4.6E+01 |
| EPHB2 | 1.31 (1.03 − 1.65) | 2.4E-02 | 4.8E+01 |
| CENPF | 1.31 (1.03 − 1.65) | 2.5E-02 | 5.0E+01 |
| ECSCR | 0.76 (0.59 − 0.97) | 2.5E-02 | 5.0E+01 |
| GPR37 | 0.77 (0.61 − 0.97) | 2.5E-02 | 5.0E+01 |
| HELLS | 1.32 (1.03 − 1.68) | 2.5E-02 | 5.0E+01 |
| CD97 | 0.74 (0.57 − 0.97) | 2.6E-02 | 5.2E+01 |
| COX7A1 | 1.30 (1.03 − 1.64) | 2.6E-02 | 5.2E+01 |
| FZD4 | 0.76 (0.60 − 0.97) | 2.6E-02 | 5.2E+01 |
| MB | 1.30 (1.03 − 1.64) | 2.6E-02 | 5.2E+01 |
| SPDEF | 1.30 (1.03 − 1.65) | 2.6E-02 | 5.2E+01 |
| SRPX | 0.77 (0.61 − 0.97) | 2.6E-02 | 5.2E+01 |
| TLR4 | 1.30 (1.03 − 1.64) | 2.6E-02 | 5.2E+01 |
| HMGB3 | 1.32 (1.03 − 1.69) | 2.7E-02 | 5.4E+01 |
| CFP | 0.77 (0.61 − 0.97) | 2.8E-02 | 5.5E+01 |
| SH2D3C | 1.30 (1.03 − 1.64) | 2.8E-02 | 5.5E+01 |
| TOM1L1 | 0.77 (0.61 − 0.97) | 2.8E-02 | 5.5E+01 |
| CBLC | 0.77 (0.61 − 0.97) | 2.9E-02 | 5.7E+01 |
| BCL6B | 1.30 (1.02 − 1.66) | 3.0E-02 | 5.9E+01 |
| KCNK1 | 1.25 (1.02 − 1.52) | 3.0E-02 | 5.9E+01 |
| NGEF | 1.31 (1.03 − 1.66) | 3.0E-02 | 5.9E+01 |
| GPR89A | 0.78 (0.61 − 0.98) | 3.1E-02 | 6.1E+01 |
| ABCC3 | 0.78 (0.62 − 0.98) | 3.2E-02 | 6.3E+01 |
| OCIAD2 | 0.77 (0.60 − 0.98) | 3.2E-02 | 6.3E+01 |
| ABLIM3 | 1.29 (1.02 − 1.63) | 3.3E-02 | 6.5E+01 |
| CREB3L4 | 0.77 (0.60 − 0.98) | 3.3E-02 | 6.5E+01 |
| MMP3 | 1.29 (1.02 − 1.62) | 3.3E-02 | 6.5E+01 |
| CSF3 | 1.28 (1.02 − 1.62) | 3.4E-02 | 6.7E+01 |
| KDR | 0.80 (0.66 − 0.98) | 3.4E-02 | 6.7E+01 |
| LRRC32 | 0.77 (0.61 − 0.98) | 3.4E-02 | 6.7E+01 |
| MYO5B | 1.30 (1.02 − 1.66) | 3.4E-02 | 6.7E+01 |
| ESRP1 | 0.77 (0.61 − 0.98) | 3.5E-02 | 6.9E+01 |
| AMOTL1 | 1.29 (1.02 − 1.65) | 3.6E-02 | 7.1E+01 |
| GCNT3 | 0.78 (0.62 − 0.98) | 3.6E-02 | 7.1E+01 |
| LYVE1 | 1.28 (1.02 − 1.62) | 3.6E-02 | 7.1E+01 |
| PPBP | 1.28 (1.01 − 1.61) | 3.7E-02 | 7.3E+01 |
| LINC00673 | 1.29 (1.01 − 1.65) | 3.8E-02 | 7.5E+01 |
| DNAH14 | 0.77 (0.61 − 0.99) | 3.9E-02 | 7.7E+01 |
| WASIR2 | 0.77 (0.61 − 0.99) | 3.9E-02 | 7.7E+01 |
| BAIAP2L1 | 1.45 (1.02 − 2.08) | 4.0E-02 | 7.9E+01 |
| CST1 | 1.28 (1.01 − 1.61) | 4.0E-02 | 7.9E+01 |
| PF4 | 1.28 (1.01 − 1.61) | 4.0E-02 | 7.9E+01 |
| ADCY4 | 0.77 (0.60 − 0.99) | 4.1E-02 | 8.1E+01 |
| DNMT3A | 0.78 (0.61 − 0.99) | 4.1E-02 | 8.1E+01 |
| GADD45B | 0.78 (0.62 − 0.99) | 4.1E-02 | 8.1E+01 |
| GIMAP4 | 0.78 (0.62 − 0.99) | 4.1E-02 | 8.1E+01 |
| HBD | 1.27 (1.01 − 1.61) | 4.1E-02 | 8.1E+01 |
| SEMA6A | 0.78 (0.61 − 0.99) | 4.1E-02 | 8.1E+01 |
| SLC39A11 | 1.28 (1.01 − 1.63) | 4.2E-02 | 8.3E+01 |
| DNASE1L3 | 0.79 (0.62 − 0.99) | 4.4E-02 | 8.7E+01 |
| HSPA12B | 1.28 (1.01 − 1.63) | 4.4E-02 | 8.7E+01 |
| PLAC9 | 0.78 (0.61 − 0.99) | 4.4E-02 | 8.7E+01 |
| CLDN5 | 0.79 (0.62 − 1.00) | 4.5E-02 | 8.9E+01 |
| EFR3B | 0.78 (0.61 − 1.00) | 4.5E-02 | 8.9E+01 |
| EZH2 | 1.27 (1.01 − 1.60) | 4.5E-02 | 8.9E+01 |
| FCN1 | 1.27 (1.00 − 1.60) | 4.5E-02 | 8.9E+01 |
| HOXA5 | 0.79 (0.62 − 0.99) | 4.5E-02 | 8.9E+01 |
| LOC731424 | 1.28 (1.00 − 1.62) | 4.6E-02 | 9.1E+01 |
| NOX4 | 0.78 (0.61 − 1.00) | 4.6E-02 | 9.1E+01 |
| MXRA7 | 0.79 (0.63 − 1.00) | 4.7E-02 | 9.3E+01 |
| PTK6 | 1.27 (1.00 − 1.62) | 4.8E-02 | 9.5E+01 |
| CCNO | 1.26 (1.00 − 1.59) | 4.9E-02 | 9.7E+01 |
| FIGN | 0.78 (0.61 − 1.00) | 4.9E-02 | 9.7E+01 |
| HYAL1 | 0.79 (0.62 − 1.00) | 4.9E-02 | 9.7E+01 |
| DST | 0.78 (0.61 − 1.00) | 5.0E-02 | 9.9E+01 |
| SFTPC | 0.79 (0.63 − 1.00) | 5.0E-02 | 9.9E+01 |
| HNF4G | 1.26 (1.00 − 1.59) | 5.1E-02 | 1.0E+02 |
| CCDC141 | 0.78 (0.61 − 1.00) | 5.3E-02 | 1.1E+02 |
| AGTR1 | 0.80 (0.63 − 1.00) | 5.4E-02 | 1.1E+02 |
| COL13A1 | 0.80 (0.63 − 1.01) | 5.5E-02 | 1.1E+02 |
| ST6GALNAC3 | 0.79 (0.62 − 1.01) | 5.5E-02 | 1.1E+02 |
| ZNF280B | 0.79 (0.62 − 1.01) | 5.5E-02 | 1.1E+02 |
| THBS2 | 1.25 (0.99 − 1.58) | 5.7E-02 | 1.1E+02 |
| FERMT1 | 1.25 (0.99 − 1.58) | 5.8E-02 | 1.1E+02 |
| CEP41 | 0.79 (0.62 − 1.01) | 5.9E-02 | 1.2E+02 |
| RASIP1 | 1.27 (0.99 − 1.64) | 6.0E-02 | 1.2E+02 |
| APOBEC3A | 1.25 (0.99 − 1.58) | 6.1E-02 | 1.2E+02 |
| EGR3 | 0.80 (0.63 − 1.01) | 6.1E-02 | 1.2E+02 |
| GPR4 | 1.25 (0.99 − 1.58) | 6.1E-02 | 1.2E+02 |
| KDM5B | 1.21 (0.99 − 1.48) | 6.1E-02 | 1.2E+02 |
| AGER | 0.80 (0.64 − 1.01) | 6.3E-02 | 1.2E+02 |
| AGER | 0.80 (0.64 − 1.01) | 6.3E-02 | 1.2E+02 |
| PABPC1L | 0.79 (0.62 − 1.01) | 6.3E-02 | 1.2E+02 |
| PCSK1N | 1.25 (0.99 − 1.57) | 6.3E-02 | 1.2E+02 |
| ATP10B | 1.24 (0.99 − 1.57) | 6.4E-02 | 1.3E+02 |
| CA4 | 1.24 (0.98 − 1.56) | 6.9E-02 | 1.4E+02 |
| CRTAC1 | 0.80 (0.63 − 1.02) | 6.9E-02 | 1.4E+02 |
| NXF3 | 1.24 (0.98 − 1.56) | 6.9E-02 | 1.4E+02 |
| CRYAB | 1.24 (0.98 − 1.56) | 7.0E-02 | 1.4E+02 |
| STX11 | 0.80 (0.63 − 1.02) | 7.0E-02 | 1.4E+02 |
| SOSTDC1 | 0.81 (0.64 − 1.02) | 7.3E-02 | 1.4E+02 |
| KIAA1211L | 0.72 (0.50 − 1.03) | 7.4E-02 | 1.5E+02 |
| NLN | 1.25 (0.98 − 1.59) | 7.4E-02 | 1.5E+02 |
| CDKN1C | 1.24 (0.98 − 1.56) | 7.5E-02 | 1.5E+02 |
| PCOLCE2 | 0.81 (0.64 − 1.02) | 7.5E-02 | 1.5E+02 |
| ARC | 1.24 (0.98 − 1.56) | 7.6E-02 | 1.5E+02 |
| FARP1 | 0.81 (0.64 − 1.02) | 7.7E-02 | 1.5E+02 |
| BCHE | 0.83 (0.68 − 1.02) | 7.8E-02 | 1.5E+02 |
| MYCT1 | 0.81 (0.63 − 1.02) | 7.8E-02 | 1.5E+02 |
| CHMP4C | 0.81 (0.63 − 1.03) | 7.9E-02 | 1.6E+02 |
| KIF11 | 1.23 (0.98 − 1.56) | 7.9E-02 | 1.6E+02 |
| PHLDA2 | 1.23 (0.98 − 1.55) | 8.0E-02 | 1.6E+02 |
| SLC46A2 | 0.81 (0.63 − 1.03) | 8.0E-02 | 1.6E+02 |
| TRIM59 | 1.24 (0.97 − 1.58) | 8.1E-02 | 1.6E+02 |
| SGPP2 | 0.81 (0.64 − 1.03) | 8.3E-02 | 1.6E+02 |
| CLEC14A | 0.81 (0.64 − 1.03) | 8.4E-02 | 1.7E+02 |
| REEP6 | 1.23 (0.97 − 1.57) | 8.9E-02 | 1.8E+02 |
| ST14 | 1.22 (0.97 − 1.54) | 9.2E-02 | 1.8E+02 |
| FAM46B | 0.81 (0.64 − 1.04) | 9.3E-02 | 1.8E+02 |
| ITPRIP | 1.23 (0.96 − 1.56) | 9.7E-02 | 1.9E+02 |
| ANK2 | 1.22 (0.96 − 1.53) | 9.8E-02 | 1.9E+02 |
| CENPK | 1.23 (0.96 − 1.56) | 9.8E-02 | 1.9E+02 |
| IL17D | 0.81 (0.64 − 1.04) | 9.8E-02 | 1.9E+02 |
| ACSL4 | 0.82 (0.65 − 1.04) | 1.0E-01 | 2.0E+02 |
| ATP1A2 | 0.85 (0.69 − 1.03) | 1.0E-01 | 2.0E+02 |
| KCNK3 | 1.18 (0.97 − 1.45) | 1.0E-01 | 2.0E+02 |
| BDNF | 0.82 (0.65 − 1.04) | 1.1E-01 | 2.2E+02 |
| CRISPLD2 | 0.83 (0.65 − 1.04) | 1.1E-01 | 2.2E+02 |
| FOXA1 | 1.21 (0.96 − 1.52) | 1.1E-01 | 2.2E+02 |
| HBEGF | 1.21 (0.96 − 1.53) | 1.1E-01 | 2.2E+02 |
| LOC728613 | 1.22 (0.96 − 1.55) | 1.1E-01 | 2.2E+02 |
| MIR21 | 0.83 (0.66 − 1.05) | 1.1E-01 | 2.2E+02 |
| POLE2 | 1.21 (0.96 − 1.52) | 1.1E-01 | 2.2E+02 |
| SIX1 | 0.82 (0.65 − 1.05) | 1.1E-01 | 2.2E+02 |
| TUBB1 | 0.82 (0.65 − 1.05) | 1.1E-01 | 2.2E+02 |
| ARL6 | 1.21 (0.95 − 1.54) | 1.2E-01 | 2.4E+02 |
| EIF2AK1 | 1.20 (0.95 − 1.51) | 1.2E-01 | 2.4E+02 |
| FBXO16 | 1.21 (0.95 − 1.54) | 1.2E-01 | 2.4E+02 |
| GNG11 | 0.83 (0.66 − 1.05) | 1.2E-01 | 2.4E+02 |
| KDELR3 | 0.85 (0.70 − 1.04) | 1.2E-01 | 2.4E+02 |
| TUBB6 | 1.20 (0.95 − 1.52) | 1.2E-01 | 2.4E+02 |
| EPHX3 | 1.20 (0.95 − 1.51) | 1.3E-01 | 2.6E+02 |
| GJA4 | 0.84 (0.66 − 1.05) | 1.3E-01 | 2.6E+02 |
| ANKRD20A9P | 0.83 (0.66 − 1.06) | 1.4E-01 | 2.8E+02 |
| C1orf162 | 1.20 (0.94 − 1.52) | 1.4E-01 | 2.8E+02 |
| GOLM1 | 0.84 (0.67 − 1.06) | 1.4E-01 | 2.8E+02 |
| HSD11B1 | 0.84 (0.66 − 1.06) | 1.4E-01 | 2.8E+02 |
| MSX1 | 1.19 (0.94 − 1.50) | 1.4E-01 | 2.8E+02 |
| PCDH12 | 1.19 (0.94 − 1.50) | 1.4E-01 | 2.8E+02 |
| RAMP3 | 1.19 (0.95 − 1.50) | 1.4E-01 | 2.8E+02 |
| SELP | 0.84 (0.66 − 1.06) | 1.4E-01 | 2.8E+02 |
| ETV4 | 1.19 (0.94 − 1.52) | 1.5E-01 | 3.0E+02 |
| TLCD1 | 1.19 (0.94 − 1.52) | 1.5E-01 | 3.0E+02 |
| TTC7B | 0.84 (0.66 − 1.06) | 1.5E-01 | 3.0E+02 |
| BAI3 | 1.16 (0.95 − 1.41) | 1.6E-01 | 3.2E+02 |
| CKS1B | 1.18 (0.94 − 1.50) | 1.6E-01 | 3.2E+02 |
| GBP4 | 1.19 (0.93 − 1.51) | 1.6E-01 | 3.2E+02 |
| GPX3 | 0.85 (0.67 − 1.07) | 1.6E-01 | 3.2E+02 |
| MUC20 | 1.19 (0.93 − 1.51) | 1.6E-01 | 3.2E+02 |
| MXRA5 | 1.18 (0.94 − 1.49) | 1.6E-01 | 3.2E+02 |
| MYOC | 0.84 (0.67 − 1.07) | 1.6E-01 | 3.2E+02 |
| TMEM45B | 0.84 (0.66 − 1.07) | 1.6E-01 | 3.2E+02 |
| BZW2 | 1.15 (0.94 − 1.41) | 1.7E-01 | 3.4E+02 |
| C11orf80 | 1.18 (0.93 − 1.49) | 1.7E-01 | 3.4E+02 |
| C1orf115 | 1.18 (0.93 − 1.48) | 1.7E-01 | 3.4E+02 |
| FABP5 | 0.85 (0.67 − 1.07) | 1.7E-01 | 3.4E+02 |
| IGFL2 | 1.18 (0.93 − 1.51) | 1.7E-01 | 3.4E+02 |
| TMEM88 | 1.19 (0.93 − 1.51) | 1.7E-01 | 3.4E+02 |
| BCL11A | 1.15 (0.94 − 1.40) | 1.8E-01 | 3.6E+02 |
| KRT15 | 0.85 (0.68 − 1.08) | 1.8E-01 | 3.6E+02 |
| FENDRR | 1.17 (0.92 − 1.49) | 1.9E-01 | 3.8E+02 |
| GPD1 | 1.17 (0.93 − 1.47) | 1.9E-01 | 3.8E+02 |
| HBG2 | 1.17 (0.93 − 1.47) | 1.9E-01 | 3.8E+02 |
| KCNJ8 | 0.87 (0.71 − 1.07) | 1.9E-01 | 3.8E+02 |
| SLFN13 | 0.85 (0.67 − 1.08) | 1.9E-01 | 3.8E+02 |
| TENC1 | 0.86 (0.68 − 1.08) | 1.9E-01 | 3.8E+02 |
| VIPR1 | 0.85 (0.68 − 1.08) | 1.9E-01 | 3.8E+02 |
| AHCY | 1.16 (0.92 − 1.47) | 2.0E-01 | 4.0E+02 |
| LRRC15 | 1.16 (0.92 − 1.47) | 2.0E-01 | 4.0E+02 |
| NOTCH4 | 1.16 (0.92 − 1.47) | 2.0E-01 | 4.0E+02 |
| ARAP3 | 1.17 (0.92 − 1.48) | 2.1E-01 | 4.2E+02 |
| CHPT1 | 0.86 (0.68 − 1.09) | 2.1E-01 | 4.2E+02 |
| CLEC1A | 0.86 (0.68 − 1.09) | 2.1E-01 | 4.2E+02 |
| PLEK2 | 1.16 (0.92 − 1.46) | 2.1E-01 | 4.2E+02 |
| CASC5 | 1.16 (0.92 − 1.46) | 2.2E-01 | 4.4E+02 |
| FCN3 | 1.16 (0.92 − 1.46) | 2.2E-01 | 4.4E+02 |
| GPC3 | 0.87 (0.69 − 1.09) | 2.3E-01 | 4.6E+02 |
| SLCO5A1 | 1.15 (0.91 − 1.45) | 2.3E-01 | 4.6E+02 |
| VTCN1 | 1.15 (0.91 − 1.46) | 2.3E-01 | 4.6E+02 |
| EFNA4 | 1.15 (0.91 − 1.45) | 2.4E-01 | 4.8E+02 |
| ESAM | 0.87 (0.68 − 1.10) | 2.4E-01 | 4.8E+02 |
| PREX2 | 0.86 (0.68 − 1.10) | 2.4E-01 | 4.8E+02 |
| SPOCK2 | 1.15 (0.91 − 1.45) | 2.4E-01 | 4.8E+02 |
| STYK1 | 1.15 (0.91 − 1.45) | 2.4E-01 | 4.8E+02 |
| ADAMTS15 | 0.87 (0.68 − 1.10) | 2.5E-01 | 5.0E+02 |
| ADTRP | 0.87 (0.68 − 1.11) | 2.5E-01 | 5.0E+02 |
| DUOX1 | 0.87 (0.69 − 1.10) | 2.5E-01 | 5.0E+02 |
| FABP4 | 0.87 (0.69 − 1.10) | 2.5E-01 | 5.0E+02 |
| NFE2L3 | 0.87 (0.68 − 1.10) | 2.5E-01 | 5.0E+02 |
| RGS17 | 0.87 (0.69 − 1.10) | 2.5E-01 | 5.0E+02 |
| SLCO2A1 | 0.87 (0.69 − 1.10) | 2.5E-01 | 5.0E+02 |
| SPRYD7 | 0.87 (0.68 − 1.10) | 2.5E-01 | 5.0E+02 |
| CERS6 | 1.15 (0.90 − 1.46) | 2.6E-01 | 5.2E+02 |
| CES1 | 0.87 (0.69 − 1.10) | 2.6E-01 | 5.2E+02 |
| KCNT2 | 0.82 (0.57 − 1.16) | 2.6E-01 | 5.2E+02 |
| LINC01207 | 0.87 (0.68 − 1.11) | 2.6E-01 | 5.2E+02 |
| PKIG | 0.87 (0.69 − 1.10) | 2.6E-01 | 5.2E+02 |
| C1orf106 | 1.14 (0.90 − 1.44) | 2.7E-01 | 5.4E+02 |
| CCL23 | 1.14 (0.90 − 1.44) | 2.7E-01 | 5.4E+02 |
| CTNNAL1 | 0.88 (0.70 − 1.11) | 2.7E-01 | 5.4E+02 |
| PCAT6 | 1.15 (0.90 − 1.46) | 2.7E-01 | 5.4E+02 |
| TTLL7 | 0.88 (0.69 − 1.11) | 2.7E-01 | 5.4E+02 |
| AFAP1L1 | 1.14 (0.90 − 1.45) | 2.8E-01 | 5.5E+02 |
| NRN1 | 0.88 (0.70 − 1.11) | 2.9E-01 | 5.7E+02 |
| PKDCC | 1.14 (0.89 − 1.45) | 2.9E-01 | 5.7E+02 |
| RETN | 0.88 (0.70 − 1.11) | 2.9E-01 | 5.7E+02 |
| SLC19A3 | 0.88 (0.69 − 1.12) | 2.9E-01 | 5.7E+02 |
| SRD5A3 | 1.14 (0.89 − 1.45) | 3.0E-01 | 5.9E+02 |
| TPBG | 1.13 (0.90 − 1.42) | 3.0E-01 | 5.9E+02 |
| VSIG4 | 1.13 (0.90 − 1.43) | 3.0E-01 | 5.9E+02 |
| C2CD4A | 0.88 (0.69 − 1.12) | 3.1E-01 | 6.1E+02 |
| EMR1 | 1.12 (0.89 − 1.42) | 3.2E-01 | 6.3E+02 |
| ZNF331 | 1.13 (0.89 − 1.44) | 3.2E-01 | 6.3E+02 |
| ARHGEF15 | 1.12 (0.89 − 1.42) | 3.3E-01 | 6.5E+02 |
| HSPB2 | 0.89 (0.70 − 1.12) | 3.3E-01 | 6.5E+02 |
| KNTC1 | 1.12 (0.89 − 1.41) | 3.4E-01 | 6.7E+02 |
| MAP7D2 | 0.89 (0.70 − 1.13) | 3.4E-01 | 6.7E+02 |
| KIAA0907 | 0.91 (0.74 − 1.11) | 3.5E-01 | 6.9E+02 |
| SHANK3 | 0.89 (0.70 − 1.14) | 3.5E-01 | 6.9E+02 |
| STIL | 0.90 (0.71 − 1.13) | 3.7E-01 | 7.3E+02 |
| KIAA1217 | 0.85 (0.60 − 1.22) | 3.8E-01 | 7.5E+02 |
| SMPDL3B | 0.90 (0.72 − 1.14) | 3.9E-01 | 7.7E+02 |
| UGT8 | 1.11 (0.88 − 1.40) | 3.9E-01 | 7.7E+02 |
| ANKRD22 | 0.90 (0.71 − 1.15) | 4.0E-01 | 7.9E+02 |
| CDCA7 | 1.11 (0.87 − 1.41) | 4.0E-01 | 7.9E+02 |
| RTKN2 | 1.11 (0.87 − 1.41) | 4.0E-01 | 7.9E+02 |
| CORO2B | 0.90 (0.71 − 1.15) | 4.1E-01 | 8.1E+02 |
| UPK3B | 1.10 (0.87 − 1.39) | 4.1E-01 | 8.1E+02 |
| AUNIP | 1.16 (0.81 − 1.65) | 4.2E-01 | 8.3E+02 |
| ELMOD2 | 1.10 (0.87 − 1.40) | 4.3E-01 | 8.5E+02 |
| LINC01296 | 1.10 (0.87 − 1.40) | 4.3E-01 | 8.5E+02 |
| SERPING1 | 1.10 (0.87 − 1.38) | 4.3E-01 | 8.5E+02 |
| TMED3 | 0.91 (0.71 − 1.16) | 4.4E-01 | 8.7E+02 |
| NDRG4 | 1.09 (0.87 − 1.38) | 4.5E-01 | 8.9E+02 |
| SPINT2 | 1.09 (0.86 − 1.37) | 4.8E-01 | 9.5E+02 |
| FCRL5 | 0.92 (0.72 − 1.17) | 4.9E-01 | 9.7E+02 |
| GMFG | 0.92 (0.73 − 1.16) | 5.0E-01 | 9.9E+02 |
| SERTM1 | 0.92 (0.72 − 1.17) | 5.0E-01 | 9.9E+02 |
| WISP2 | 1.08 (0.86 − 1.37) | 5.0E-01 | 9.9E+02 |
| LGI3 | 0.92 (0.72 − 1.18) | 5.1E-01 | 1.0E+03 |
| PRDX4 | 0.93 (0.73 − 1.17) | 5.2E-01 | 1.0E+03 |
| PSMG3 | 1.08 (0.85 − 1.38) | 5.2E-01 | 1.0E+03 |
| CXCL13 | 0.93 (0.74 − 1.17) | 5.3E-01 | 1.1E+03 |
| LAMC3 | 1.08 (0.85 − 1.36) | 5.3E-01 | 1.1E+03 |
| KCNN4 | 1.07 (0.87 − 1.30) | 5.4E-01 | 1.1E+03 |
| PPARG | 0.93 (0.74 − 1.18) | 5.5E-01 | 1.1E+03 |
| CMTM2 | 1.07 (0.84 − 1.37) | 5.6E-01 | 1.1E+03 |
| SNRNP25 | 1.07 (0.85 − 1.35) | 5.6E-01 | 1.1E+03 |
| ERP44 | 0.94 (0.74 − 1.18) | 5.7E-01 | 1.1E+03 |
| FRMD5 | 1.07 (0.84 − 1.36) | 5.7E-01 | 1.1E+03 |
| BCL2L15 | 0.90 (0.64 − 1.29) | 5.8E-01 | 1.1E+03 |
| WFDC1 | 0.94 (0.74 − 1.18) | 5.8E-01 | 1.1E+03 |
| GPRIN2 | 1.07 (0.84 − 1.36) | 5.9E-01 | 1.2E+03 |
| MMP13 | 1.07 (0.85 − 1.34) | 5.9E-01 | 1.2E+03 |
| BRE-AS1 | 0.94 (0.74 − 1.19) | 6.0E-01 | 1.2E+03 |
| CD34 | 0.94 (0.74 − 1.19) | 6.0E-01 | 1.2E+03 |
| DNAJB4 | 0.94 (0.75 − 1.19) | 6.0E-01 | 1.2E+03 |
| GPT2 | 1.06 (0.84 − 1.35) | 6.2E-01 | 1.2E+03 |
| PPAP2C | 1.06 (0.84 − 1.33) | 6.3E-01 | 1.2E+03 |
| SLC31A2 | 1.06 (0.84 − 1.34) | 6.3E-01 | 1.2E+03 |
| TMEM246 | 0.94 (0.74 − 1.20) | 6.3E-01 | 1.2E+03 |
| CLEC3B | 0.95 (0.75 − 1.19) | 6.4E-01 | 1.3E+03 |
| GPA33 | 1.06 (0.84 − 1.33) | 6.4E-01 | 1.3E+03 |
| HBB | 0.95 (0.75 − 1.19) | 6.4E-01 | 1.3E+03 |
| LIMS2 | 1.06 (0.84 − 1.34) | 6.4E-01 | 1.3E+03 |
| ATP5S | 1.05 (0.86 − 1.28) | 6.5E-01 | 1.3E+03 |
| FOXA3 | 0.95 (0.74 − 1.20) | 6.5E-01 | 1.3E+03 |
| MME | 0.95 (0.75 − 1.19) | 6.5E-01 | 1.3E+03 |
| SFTPA2 | 0.95 (0.75 − 1.20) | 6.7E-01 | 1.3E+03 |
| PROK2 | 1.05 (0.83 − 1.34) | 6.9E-01 | 1.4E+03 |
| LINC00936 | 1.05 (0.82 − 1.34) | 7.0E-01 | 1.4E+03 |
| OLFML2A | 1.05 (0.83 − 1.32) | 7.1E-01 | 1.4E+03 |
| WFDC3 | 1.05 (0.82 − 1.33) | 7.1E-01 | 1.4E+03 |
| KRTCAP3 | 1.04 (0.82 − 1.33) | 7.3E-01 | 1.4E+03 |
| ACP6 | 1.04 (0.82 − 1.31) | 7.5E-01 | 1.5E+03 |
| OLR1 | 1.03 (0.82 − 1.30) | 7.8E-01 | 1.5E+03 |
| STARD8 | 0.97 (0.76 − 1.23) | 8.0E-01 | 1.6E+03 |
| FAP | 1.03 (0.81 − 1.30) | 8.2E-01 | 1.6E+03 |
| PZP | 1.03 (0.81 − 1.30) | 8.2E-01 | 1.6E+03 |
| CSRNP1 | 1.03 (0.81 − 1.31) | 8.3E-01 | 1.6E+03 |
| MUSTN1 | 1.03 (0.81 − 1.31) | 8.3E-01 | 1.6E+03 |
| UNC5CL | 0.98 (0.77 − 1.24) | 8.4E-01 | 1.7E+03 |
| SEPT10 | 1.02 (0.81 − 1.29) | 8.5E-01 | 1.7E+03 |
| PDE2A | 1.02 (0.81 − 1.29) | 8.5E-01 | 1.7E+03 |
| MARCO | 0.98 (0.78 − 1.24) | 8.6E-01 | 1.7E+03 |
| SLC5A9 | 0.98 (0.77 − 1.25) | 8.6E-01 | 1.7E+03 |
| GKN2 | 0.98 (0.77 − 1.25) | 8.8E-01 | 1.7E+03 |
| FUT2 | 0.98 (0.78 − 1.24) | 8.9E-01 | 1.8E+03 |
| SLC35F2 | 1.02 (0.81 − 1.28) | 8.9E-01 | 1.8E+03 |
| ZNF106 | 1.02 (0.80 − 1.29) | 8.9E-01 | 1.8E+03 |
| APOL3 | 1.01 (0.80 − 1.28) | 9.0E-01 | 1.8E+03 |
| ACTG2 | 0.99 (0.78 − 1.25) | 9.1E-01 | 1.8E+03 |
| KRT80 | 1.01 (0.80 − 1.29) | 9.2E-01 | 1.8E+03 |
| ARSE | 1.01 (0.80 − 1.27) | 9.3E-01 | 1.8E+03 |
| CNN1 | 0.99 (0.79 − 1.25) | 9.4E-01 | 1.9E+03 |
| RAD51AP1 | 0.99 (0.79 − 1.25) | 9.5E-01 | 1.9E+03 |
| SEMA3G | 0.99 (0.78 − 1.26) | 9.5E-01 | 1.9E+03 |
| FXYD1 | 1.01 (0.80 − 1.27) | 9.6E-01 | 1.9E+03 |
| METTL7B | 0.99 (0.78 − 1.26) | 9.6E-01 | 1.9E+03 |
| FGR | 1.00 (0.80 − 1.27) | 9.7E-01 | 1.9E+03 |
| SPINK1 | 1.00 (0.79 − 1.26) | 9.9E-01 | 2.0E+03 |
