# Supplementary material for: A robust gene expression-based prognostic risk score predicts overall survival of lung adenocarcinoma patients
Source: Oncotarget. 2017 Dec 15;9(6):6862–71. doi: 10.18632/oncotarget.23490 (PMC5805521; doi:10.18632/oncotarget.23490)
Supplement: Supplementary file 4 [file oncotarget-09-6862-s004.doc]

| **Supplementary Table 3:** Frequency of genes appeared in Cox regression model among 100 resampling test sets. The signature genes are highlighted in yellow. | |
| --- | --- |
| Gene name | Frequency |
| FAM83A | 75 |
| STK32A | 72 |
| TRPC6 | 70 |
| DEFA1B | 68 |
| TMEM47 | 53 |
| CDC25C | 50 |
| PRKAR2B | 49 |
| TMEM100 | 47 |
| CNTN4 | 45 |
| HOOK1 | 42 |
| INPP5A | 42 |
| TRHDE | 40 |
| RSPO2 | 39 |
| LDB3 | 36 |
| SLC24A3 | 35 |
| VEPH1 | 35 |
| SLC1A1 | 34 |
| GPM6A | 33 |
| TMEM106B | 33 |
| FOXP1 | 32 |
| NTN4 | 32 |
| PALD1 | 32 |
| F12 | 31 |
| FHL1 | 31 |
| TIMP1 | 31 |
| IGSF9 | 30 |
| KLF9 | 30 |
| GALNT18 | 29 |
| HECW2 | 29 |
| IQGAP3 | 29 |
| KRT8 | 29 |
| NEDD4L | 29 |
| CILP2 | 28 |
| DSEL | 28 |
| LPL | 28 |
| STX3 | 28 |
| NHS | 26 |
| CPB2 | 25 |
| ENO1 | 25 |
| FOXF2 | 25 |
| LGR4 | 25 |
| MACF1 | 25 |
| MFI2 | 25 |
| PAIP1 | 24 |
| RNF144B | 24 |
| GATA2 | 23 |
| GNL1 | 23 |
| MTA3 | 23 |
| PC | 23 |
| SYNPO | 23 |
| ALDOA | 22 |
| ARHGEF10 | 22 |
| BTNL9 | 22 |
| FAR2 | 22 |
| FOXO1 | 22 |
| GLDN | 22 |
| ITGA8 | 22 |
| KIF18B | 22 |
| MFNG | 22 |
| P2RY14 | 22 |
| PPM1F | 22 |
| RERG | 22 |
| SCARA3 | 22 |
| SLC12A8 | 22 |
| ZNF25 | 22 |
| ADARB1 | 21 |
| CPED1 | 21 |
| FAM49A | 21 |
| FMO2 | 21 |
| HIST1H2BE | 21 |
| JAM2 | 21 |
| PARVA | 21 |
| PLEKHH2 | 21 |
| PTGIS | 21 |
| SDPR | 21 |
| TTC28 | 21 |
| TYMS | 21 |
| ANKRD29 | 20 |
| FERMT2 | 20 |
| MAOB | 20 |
| PAPSS2 | 20 |
| PGM2L1 | 20 |
| TOP2A | 20 |
| FAM13C | 19 |
| GUCY1A2 | 19 |
| IL1RL1 | 19 |
| NES | 19 |
| RWDD1 | 19 |
| SOX17 | 19 |
| TMEM64 | 19 |
| EFEMP1 | 18 |
| GIMAP1 | 18 |
| JDP2 | 18 |
| LILRB2 | 18 |
| MMRN1 | 18 |
| NAP1L5 | 18 |
| PRICKLE2 | 18 |
| SLC14A1 | 18 |
| STARD4 | 18 |
| BRIP1 | 17 |
| GALNT2 | 17 |
| GPM6B | 17 |
| MICU3 | 17 |
| MTURN | 17 |
| PDK1 | 17 |
| PGR | 17 |
| PTTG1 | 17 |
| UBE2C | 17 |
| ARAP2 | 16 |
| C4ORF48 | 16 |
| CDT1 | 16 |
| HGF | 16 |
| HMMR | 16 |
| LAD1 | 16 |
| NEBL | 16 |
| OTUD1 | 16 |
| PCM1 | 16 |
| PDE5A | 16 |
| PEG3 | 16 |
| PKP3 | 16 |
| RBM17 | 16 |
| WASF3 | 16 |
| DOK6 | 15 |
| FILIP1 | 15 |
| IFT57 | 15 |
| MCEMP1 | 15 |
| NLRC4 | 15 |
| SECISBP2L | 15 |
| SHC3 | 15 |
| SLK | 15 |
| TMEM237 | 15 |
| TOX2 | 15 |
| ACACB | 14 |
| CACNA1D | 14 |
| EPAS1 | 14 |
| ETS1 | 14 |
| ETS2 | 14 |
| EXT1 | 14 |
| HMGA1 | 14 |
| KIF2C | 14 |
| KIF4A | 14 |
| LILRA2 | 14 |
| LONRF1 | 14 |
| MAGED1 | 14 |
| MEX3A | 14 |
| MYH11 | 14 |
| NMU | 14 |
| PFDN6 | 14 |
| PGM5 | 14 |
| PMEPA1 | 14 |
| PTK2 | 14 |
| S100A12 | 14 |
| SEMA3B | 14 |
| SYNC | 14 |
| TMOD1 | 14 |
| TNS1 | 14 |
| TPPP3 | 14 |
| ZBTB16 | 14 |
| ACVRL1 | 13 |
| ARRB1 | 13 |
| C1ORF21 | 13 |
| CAMK2N1 | 13 |
| CYBRD1 | 13 |
| FRY | 13 |
| GJB2 | 13 |
| H2AFV | 13 |
| LIFR | 13 |
| LRRN3 | 13 |
| MEF2A | 13 |
| MMP12 | 13 |
| NEDD9 | 13 |
| NFIA | 13 |
| PLCE1 | 13 |
| PPP1R15A | 13 |
| S100A8 | 13 |
| SHROOM4 | 13 |
| SIK2 | 13 |
| SPG20 | 13 |
| SRPRB | 13 |
| SSH2 | 13 |
| TMEM167A | 13 |
| VSIG10 | 13 |
| AFF3 | 12 |
| CCDC68 | 12 |
| DOCK4 | 12 |
| ECM2 | 12 |
| EMP1 | 12 |
| FGD5 | 12 |
| FGF11 | 12 |
| FGFR2 | 12 |
| FKBP11 | 12 |
| FRMD4A | 12 |
| FUT3 | 12 |
| GIPC2 | 12 |
| IL18R1 | 12 |
| KIF20A | 12 |
| LDB2 | 12 |
| LEPR | 12 |
| LMOD1 | 12 |
| MYADM | 12 |
| NCKAP5 | 12 |
| NEXN | 12 |
| NLRP3 | 12 |
| NME1 | 12 |
| PDZRN3 | 12 |
| PLAGL1 | 12 |
| PNPLA6 | 12 |
| PRICKLE1 | 12 |
| PRKCH | 12 |
| SCARA5 | 12 |
| SLC35A2 | 12 |
| STRBP | 12 |
| SYNM | 12 |
| TSTA3 | 12 |
| TTN | 12 |
| ULK2 | 12 |
| AP1S1 | 11 |
| CALM1 | 11 |
| CENPU | 11 |
| ECE2 | 11 |
| EIF5 | 11 |
| FAM150B | 11 |
| FEN1 | 11 |
| FEZ1 | 11 |
| GATA6 | 11 |
| GPRASP1 | 11 |
| HS6ST2 | 11 |
| ITPR1 | 11 |
| MAP3K8 | 11 |
| MARC2 | 11 |
| MASP1 | 11 |
| NCALD | 11 |
| NEK2 | 11 |
| PDE3B | 11 |
| PDZD2 | 11 |
| PLPP4 | 11 |
| PMP22 | 11 |
| PROM2 | 11 |
| PSAT1 | 11 |
| RAPGEF5 | 11 |
| RCC1 | 11 |
| RPGR | 11 |
| SCN4B | 11 |
| SEC24A | 11 |
| SLC2A3 | 11 |
| SLC2A5 | 11 |
| SLC44A1 | 11 |
| TACC1 | 11 |
| TBX3 | 11 |
| WWC2 | 11 |
| ARHGAP29 | 10 |
| ARHGEF6 | 10 |
| C2ORF40 | 10 |
| CDCA5 | 10 |
| CDH13 | 10 |
| CHRDL1 | 10 |
| CLASP2 | 10 |
| DSTN | 10 |
| FIGF | 10 |
| FRMD4B | 10 |
| FYN | 10 |
| GABARAPL3 | 10 |
| H3F3B | 10 |
| HN1 | 10 |
| IL6 | 10 |
| IRAK3 | 10 |
| JAM3 | 10 |
| LMO7 | 10 |
| MAMDC2 | 10 |
| MIF | 10 |
| MKI67 | 10 |
| MSRB3 | 10 |
| MYH10 | 10 |
| NFIB | 10 |
| PAICS | 10 |
| PALMD | 10 |
| PBK | 10 |
| PHACTR2 | 10 |
| PIK3R1 | 10 |
| PLCL1 | 10 |
| PTPRD | 10 |
| RHOB | 10 |
| RORA | 10 |
| SATB1 | 10 |
| SDF2L1 | 10 |
| SMAD9 | 10 |
| TCF4 | 10 |
| TEK | 10 |
| TMEM204 | 10 |
| TNFRSF21 | 10 |
| TOX3 | 10 |
| ZC3H12C | 10 |
| ADH1B | 9 |
| AGTPBP1 | 9 |
| ANOS1 | 9 |
| AQP9 | 9 |
| ASF1B | 9 |
| C1QTNF2 | 9 |
| C1QTNF7 | 9 |
| CDS2 | 9 |
| CENPA | 9 |
| CNKSR2 | 9 |
| EHD2 | 9 |
| FCGR3B | 9 |
| FLI1 | 9 |
| GCOM1 | 9 |
| HN1L | 9 |
| ITM2A | 9 |
| KL | 9 |
| MGAT4A | 9 |
| MYLIP | 9 |
| NUSAP1 | 9 |
| P4HB | 9 |
| PGAP1 | 9 |
| PID1 | 9 |
| PIK3C3 | 9 |
| PIP5K1B | 9 |
| PLEKHA8 | 9 |
| PLPP5 | 9 |
| PODXL | 9 |
| PPP1R14B | 9 |
| PTPRM | 9 |
| QKI | 9 |
| RBMS2 | 9 |
| RHOJ | 9 |
| RNF180 | 9 |
| RNF182 | 9 |
| ROR1 | 9 |
| SAMD5 | 9 |
| SCN7A | 9 |
| SNRK | 9 |
| TMEM99 | 9 |
| TMOD2 | 9 |
| TPX2 | 9 |
| UBASH3B | 9 |
| UBFD1 | 9 |
| VGLL3 | 9 |
| WWTR1 | 9 |
| ZEB1 | 9 |
| ACE | 8 |
| ADRB2 | 8 |
| ANLN | 8 |
| ARGLU1 | 8 |
| AURKA | 8 |
| BIRC5 | 8 |
| BMPER | 8 |
| C14ORF132 | 8 |
| CD59 | 8 |
| COL1A1 | 8 |
| CREM | 8 |
| EDNRB | 8 |
| EXO1 | 8 |
| GIMAP5 | 8 |
| GPI | 8 |
| H2AFJ | 8 |
| ID2 | 8 |
| IL11RA | 8 |
| IL33 | 8 |
| KCNAB1 | 8 |
| KIAA0101 | 8 |
| KLF13 | 8 |
| KLF6 | 8 |
| LAPTM4B | 8 |
| LATS2 | 8 |
| LHFP | 8 |
| LIMCH1 | 8 |
| MAD2L1 | 8 |
| MATN2 | 8 |
| MCC | 8 |
| MEIS2 | 8 |
| MSI2 | 8 |
| NACC2 | 8 |
| NCAPH | 8 |
| PDK4 | 8 |
| PELO | 8 |
| PKNOX2 | 8 |
| PLSCR4 | 8 |
| PRKG1 | 8 |
| RAP1A | 8 |
| RCCD1 | 8 |
| RPA3 | 8 |
| RPL39L | 8 |
| S100A10 | 8 |
| SGPL1 | 8 |
| SH3BP5 | 8 |
| SH3GL3 | 8 |
| SLC11A1 | 8 |
| SLC39A8 | 8 |
| SMAD6 | 8 |
| SMAD7 | 8 |
| SRPK1 | 8 |
| SSBP2 | 8 |
| STARD13 | 8 |
| SYNPO2 | 8 |
| TCF21 | 8 |
| TIMP3 | 8 |
| TNXB | 8 |
| TPD52 | 8 |
| WIF1 | 8 |
| ZEB2 | 8 |
| ABCA6 | 7 |
| ASPA | 7 |
| BDH2 | 7 |
| CCNB1 | 7 |
| CDCA8 | 7 |
| CGNL1 | 7 |
| CHEK1 | 7 |
| DENND2A | 7 |
| FAM199X | 7 |
| FANCI | 7 |
| FOXM1 | 7 |
| GIMAP7 | 7 |
| HEG1 | 7 |
| HJURP | 7 |
| INMT | 7 |
| KANK2 | 7 |
| KLF11 | 7 |
| LMNB1 | 7 |
| LRCH2 | 7 |
| MAGI1 | 7 |
| MCTS1 | 7 |
| MYLK | 7 |
| NET1 | 7 |
| PCBD2 | 7 |
| PDE4D | 7 |
| PDZD11 | 7 |
| PEAK1 | 7 |
| PKHD1L1 | 7 |
| PLAU | 7 |
| PRDM5 | 7 |
| PTPRB | 7 |
| PTRF | 7 |
| RAB8B | 7 |
| RAPGEF4 | 7 |
| RFC4 | 7 |
| RFX3 | 7 |
| RHBDL2 | 7 |
| RMI2 | 7 |
| RNF125 | 7 |
| RUNX1T1 | 7 |
| SAMHD1 | 7 |
| SEPT7 | 7 |
| SESN1 | 7 |
| SLIT2 | 7 |
| STX1A | 7 |
| STXBP6 | 7 |
| TBX5 | 7 |
| TFAP2A | 7 |
| TIMELESS | 7 |
| UQCC2 | 7 |
| VAPA | 7 |
| WISP1 | 7 |
| ADAMTSL3 | 6 |
| AOC3 | 6 |
| CCNE1 | 6 |
| CDC6 | 6 |
| CDO1 | 6 |
| CFLAR | 6 |
| DAPK2 | 6 |
| DEPDC1 | 6 |
| DNAJC27 | 6 |
| ENC1 | 6 |
| ENG | 6 |
| GYPC | 6 |
| HSPB8 | 6 |
| IDH2 | 6 |
| IGSF10 | 6 |
| KIAA0040 | 6 |
| KIF14 | 6 |
| LGALSL | 6 |
| LRRFIP1 | 6 |
| MCM4 | 6 |
| MDK | 6 |
| MEIS1 | 6 |
| METTL7A | 6 |
| MND1 | 6 |
| PCDH7 | 6 |
| PCDH9 | 6 |
| PDIA6 | 6 |
| PHLDB2 | 6 |
| PPP1R16B | 6 |
| RAI2 | 6 |
| RECK | 6 |
| RGS13 | 6 |
| RNASEH2A | 6 |
| RTN1 | 6 |
| SEC63 | 6 |
| SHMT2 | 6 |
| SLC1A4 | 6 |
| SLC2A1 | 6 |
| SPARCL1 | 6 |
| SPC25 | 6 |
| SULF1 | 6 |
| SYNE1 | 6 |
| TXNL1 | 6 |
| UGGT1 | 6 |
| UHRF1 | 6 |
| UTRN | 6 |
| VLDLR | 6 |
| ABCA8 | 5 |
| ACSS3 | 5 |
| C4ORF32 | 5 |
| C5AR1 | 5 |
| CCNB2 | 5 |
| CDCA3 | 5 |
| CDKN3 | 5 |
| DOCK9 | 5 |
| GHR | 5 |
| GIMAP6 | 5 |
| GIMAP8 | 5 |
| GNAQ | 5 |
| GRK5 | 5 |
| GTSE1 | 5 |
| LMCD1 | 5 |
| MAL | 5 |
| MCM2 | 5 |
| MFAP4 | 5 |
| MMRN2 | 5 |
| MPDZ | 5 |
| NCAPG | 5 |
| NPNT | 5 |
| OLFML1 | 5 |
| PLPP3 | 5 |
| PRRC1 | 5 |
| PTPN21 | 5 |
| RBMS3 | 5 |
| RGS5 | 5 |
| RRM2 | 5 |
| SAP18 | 5 |
| SASH1 | 5 |
| SCAI | 5 |
| SETBP1 | 5 |
| SH3D19 | 5 |
| SOBP | 5 |
| SOX7 | 5 |
| SPAG5 | 5 |
| TNPO1 | 5 |
| TPPP | 5 |
| ZAK | 5 |
| ADGRL2 | 4 |
| ADRB1 | 4 |
| C1ORF112 | 4 |
| CBX7 | 4 |
| CD93 | 4 |
| CNRIP1 | 4 |
| CYYR1 | 4 |
| DSG2 | 4 |
| FGF2 | 4 |
| ID4 | 4 |
| IL4I1 | 4 |
| KANK3 | 4 |
| LSAMP | 4 |
| NFASC | 4 |
| PDS5B | 4 |
| RAB11A | 4 |
| RHNO1 | 4 |
| S1PR1 | 4 |
| SEPT8 | 4 |
| SNX1 | 4 |
| TGFBR3 | 4 |
| TMEM178A | 4 |
| TTK | 4 |
| UACA | 4 |
| USP53 | 4 |
| ADAM12 | 3 |
| ANKS1A | 3 |
| CRIM1 | 3 |
| FAM162B | 3 |
| FRAS1 | 3 |
| GINS2 | 3 |
| GNG2 | 3 |
| LRCH1 | 3 |
| MMP11 | 3 |
| PPARGC1A | 3 |
| PRX | 3 |
| TK1 | 3 |
| UBE2T | 3 |
| DLC1 | 2 |
| LAMA4 | 2 |
| PTPRG | 2 |
| CDC20 | 1 |
| SEMA6D | 1 |
